# The Fission Yeast GATA Factor, Gaf1, Modulates Sexual Development via Direct Down-Regulation of *ste11^+^* Expression in Response to Nitrogen Starvation

**DOI:** 10.1371/journal.pone.0042409

**Published:** 2012-08-10

**Authors:** Lila Kim, Kwang-Lae Hoe, Yeong Man Yu, Ji-Hyun Yeon, Pil Jae Maeng

**Affiliations:** 1 Department of Microbiology and Molecular Biology, Chungnam National University, Daejeon, Korea; 2 Graduate School of New Drug Discovery and Development, Chungnam National University, Daejeon, Korea; Yonsei University, Korea, Republic of

## Abstract

Gaf1 is the first GATA family zinc-finger transcription factor identified in *Schizosaccharomyces pombe*. Here, we report that Gaf1 functions as a negatively acting transcription factor of *ste11^+^*, delaying the entrance of cells exposed to transient nitrogen starvation into the meiotic cycle. *gaf1Δ* strains exhibited accelerated G_1_-arrest upon nitrogen starvation. Moreover, *gaf1Δ* mutation caused increased mating and sporulation frequency under both nitrogen-starved and unstarved conditions, while overexpression of *gaf1^+^* led to a significant impairment of sporulation. By microarray analysis, we found that approximately 63% (116 genes) of the 183 genes up-regulated in unstarved *gaf1Δ* cells were nitrogen starvation-responsive genes, and furthermore that 25 genes among the genes up-regulated by *gaf1*Δ mutation are Ste11 targets (*e.g.*, *gpa1* ^+^, *ste4* ^+^, *spk1* ^+^, *ste11* ^+^, and *mei2* ^+^). The phenotype caused by *gaf1Δ* mutation was masked by *ste11Δ* mutation, indicating that *ste11^+^* is epistatic to *gaf1^+^* with respect to sporulation efficiency, and accordingly that *gaf1^+^* functions upstream of *ste11^+^* in the signaling pathway governing sexual development. *gaf1Δ* strains showed accelerated *ste11^+^* expression under nitrogen starvation and increased *ste11^+^* expression even under normal conditions. Electrophoretic mobility shift assay analysis demonstrated that Gaf1 specifically binds to the canonical GATA motif (5′-HGATAR-3′) spanning from −371 to −366 in *ste11^+^* promoter. Consequently, Gaf1 provides the prime example for negative regulation of *ste11^+^* transcription through direct binding to a *cis*-acting motif of its promoter.

## Introduction

The fission yeast *Schizosaccharomyces pombe* reproduces asexually by mitosis under favorable conditions. When haploid cells are starved of nutrients, particularly of nitrogen, they arrest the cell cycle at G_1_ and undergo sexual differentiation [1]. Cells of opposite mating types, *h^+^* and *h^−^*, fuse to form a diploid zygote, which undergoes meiosis to give four haploid ascospores that remain dormant until they encounter favorable growth conditions [2]. The transition from the mitotic cell cycle into meiosis is tightly regulated by a network of positive and negative factors that are controlled at various levels of gene expression, from transcription initiation [3]–[6] to protein modification [7]–[11].

One important regulatory component of *S. pombe* sexual development is Ste11. Ste11 positively regulates transcription of the mating type genes, *matP* and *matM,* and the *mei2^+^* gene, which is essential for commitment to meiosis, by binding to an upstream *cis*-acting element under conditions of nitrogen starvation [12]. *ste11Δ* mutants are completely defective in mating and sporulation, while ectopic expression of *ste11^+^* leads to sexual differentiation irrespective of nutritional conditions. The activity of Ste11 is regulated by two antagonistic protein kinases, Pat1 and Spk1, via the pheromone signaling pathway [13] and by the TOR protein kinase, Tor2, which is activated in the presence of nitrogen and represses sexual differentiation by directly interfering with the function of Ste11 and Mei2 [11]. Expression of *ste11^+^* is regulated by at least three different signal transduction pathways: mating pheromone signaling (RAS/MAPK pathway), cyclic AMP (cAMP)-dependent protein kinase A (PKA), and stress-activated protein kinase (SAPK) in conjunction with MAPKKKs (Wis4 and Win1), MAPKK (Wis1), and MAPK (Sty1/Spc1/Phh1) [14], [15]. So far, only positive regulatory factors of *ste11^+^* expression, such as Atf1 [6], [16], Pcr1 [5], Rst2 [4], Prr1 [17], and Ste11 itself [4] have been reported. Furthermore, only Rst2 and Ste11 are transcription factors that directly activate *ste11^+^* expression. No transcription factor that directly represses the expression of *ste11^+^* has been identified.

Here, we explore the role of Gaf1, the first GATA transcription factor identified in *S. pombe* [18], [19], in the expression of *ste11^+^*. GATA family transcription factors have a wide range of functions, from terminal differentiation in vertebrates [20]–[22] to nitrogen metabolism, siderophore biosynthesis, photoinduction, and mating type switching in fungi [23]. In *S. pombe*, Ams2 is a cell cycle-regulated GATA factor that is required for centromere function [24] and Fep1/Gaf2 occupies a central role in iron homeostasis by coordinating the reductive and non-reductive iron transport systems [25], [26]. GATA factors recognize a six base-pair consensus sequence, 5′-HGATAR-3′ (where H can be A/C/T and R can be A/G), contained in the promoters of their target genes. Although the C-terminal fragment of Gaf1 (Gaf1_565–855_) has been shown to bind specifically to the GATA motif *DAL7* UAS, a canonical GATA motif of *Saccharomyces cerevisiae* [18], little is known about the function of Gaf1 in *S. pombe*. We present evidence that Gaf1 down-regulates the transcription of *ste11^+^* via direct binding to its promoter and consequently delay the shift of nitrogen-starved cells from the vegetative cycle to the meiotic cycle.

## Materials and Methods

### *S. pombe* strains, media, and general procedures

*S. pombe* strains used in this study are listed in Table 1. Cells were maintained on complete medium (YES) containing 0.5% yeast extract, 3% glucose, 2% Bacto agar, adenine (225 µg ml^−1^), leucine (225 µg ml^−1^), and uracil (225 µg ml^−1^). Edinburgh minimal medium (EMM2) [27]–[29] was used as a minimal selective medium. EMM-N (EMM2 without NH_4_Cl) was used for starvation of nitrogen source, and EMM-G (EMM2 containing 0.5% instead of 2% glucose) for glucose-restriction experiments. All the minimal media were supplemented with required auxotrophic nutrients (adenine, leucine, and uracil) at the concentrations of 225 µg ml^−1^ each, which led to the presence of a starved amount of organic nitrogen source in EMM-N. Thiamine was added to the medium at a final concentration of 20 µM to repress expression from the thiamine-repressible *nmt42^+^* (no message in thiamine) promoter. Transformation was performed by the lithium acetate procedure [27]. Standard techniques were used for genetic manipulation and analysis [29].

**Table 1:** *S. pombe* strains used in this study.

Strain	Relevant genotype	Source or reference
972	*h^−^*	Lab stock
ED005	*h^−^ ade6-M210 leu1–32*	Lab stock
ED665	*h^−^ ade6-M210 leu1–32 ura4-D18*	Lab stock
ED668	*h^+^ ade6-M216 leu1–32 ura4-D18*	Lab stock
JY4	*h^90^ ade6-M216 leu1–32 ura4-D18*	Lab stock
JZ396	*h^90^ ade6-M216 leu1–32 ura4-D18 ste11::ura4^+^*	[12]
KL210	*h^−^ ade6-M210 leu1–32 ura4-D18 gaf1::kanMX*	This work
KL211	*h^90^ ade6-M216 leu1–32 ura4-D18 gaf1::kanMX*	This work
KL213	*h^90^ ade6-M216 leu1–32 ura4-D18 gaf1::kanMX ste11::ura4^+^*	This work
KL216	*h^+^ ade6-M216 leu1–32 ura4-D18 gaf1::kanMX*	This work
KL230	*h^−^ gaf1::kanMX*	This work
KL240	*h^−^ ade6-M210 leu1–32 ura4-D18 gaf1::hphMX*	This work
KL416	*h^+^ ade6-M216 leu1–32 ura4-D18 ste11::kanMX*	This work

### Construction of plasmids

To construct pREP-Gaf1 with a full-length open reading frame (ORF) of *gaf1^+^* downstream of the *nmt42^+^* promoter, a 2.6-kb fragment was amplified by polymerase chain reaction (PCR) with the following primers: P1 (5′AACCCGGGCCATGGATCTAAAGTTTTCC3′) and P2 (5′AACCCGGGCATAACGCTATACCAATC3′) in which underlines designate *Sma*I sites. The resulting PCR product was cloned into pGEM-T (Promega) to yield pGEM-Gaf1. After confirming the absence of PCR artifact by sequence analysis, the *gaf1^+^* ORF was excised by digestion with *Sma*I and ligated to *Sma*I-digested middle-copy expression vector pREP42 [30] to yield pREP-Gaf1. For construction of pGEX4T3-Gaf1 encoding the glutathione *S*-transferase (GST)-tagged Gaf1 (GST-Gaf1), the 2.6-kb *gaf1^+^* ORF was excised from pGEM-Gaf1 by *Sma*I-digestion and cloned in frame into the *Sma*I site of pGEX4T3 (Amersham).

A *lacZ*-based reporter plasmid used for analysis of *ste11^+^* promoter was constructed as follows. First, the replication origin of *S. pombe*, *ars1^+^*, was amplified from pESP1 (Stratagene) by PCR using primers P3 (5′CCGAATTCAGGCCTGAGTCTAACTCCTTAACCACT3′; underline designates *Stu*I site) and P4 (5′CCGATATCCAACCTTCCAATTCATTAAATC3′; underline designates *Eco*RV site). The 1.2-kb DNA fragment was cloned into pGEM-T Easy vector to yield pGEM-ars1. A 1.5-kb fragment containing the *kanMX* gene was amplified from pFA6a-kanMX6 [31] using PCR primers P5 (5′CCGATATCGGGTTAATTAAGGCGCGCCAGA3′; underline designates *Eco*RV site) and P6 (5′CCGCATGCCACTGGATGACGGCGTTAGTAT3′; underline designates *Sph*I site) and inserted into the pGEM-T vector to yield pGEM-kanMX6. The 1.5-kb *kanMX* fragment was then excised by *Eco*RV-*Sph*I double digestion and subcloned into *Eco*RV-*Sph*I-digested pGEM-ars1. From the resulting pGEM-ars1-kanMX6 vector, a 2.7-kb fragment containing both *ars1* and *kanMX* was excised with *Sph*I, Klenow enzyme, and *Stu*I. The resulting 2.7-kb *Sph*I(blunted)-*Stu*I fragment was then ligated with the 6.5-kb *Sna*BI-*Stu*I fragment of YEp353 plasmid to yield pJLC-LacZ. Then a 1.4-kb *Bam*HI fragment containing the promoter region of *ste11^+^* from −834 to +575 (the major transcription start site is assigned as position +1 for nucleotide numbering) [4] was PCR-amplified using primers P7 (5′GGATCCGCATGCCATCTCCAGGGAT3′) and P8 (5′GGATCCCAAAAGAACGTAGAGGCAA3′) in which underlines designate *Bam*HI sites. The reporter plasmid pJLC-Ste11_(p)_-LacZ was constructed by subcloning the PCR-amplified 1.4-kb *Bam*HI fragment into the *Bam*HI site of pJLC-LacZ upstream of *lacZ* in the correct orientation.

### Gene disruption

Construction of *gaf1Δ* strains was performed by direct chromosomal integration as described previously [28]. The 2.6-kb genomic regions corresponding to the entire *gaf1^+^* ORF (855 amino acids) of the wild-type strains, 972, ED665, JY4, and ED668, carrying different auxotrophic markers or mating type were replaced with the 1.5-kb PCR-amplified *gaf1::kanMX* deletion cassette derived from pFA6a-kanMX4 [32]. Stable transformants were selected by resistance to G418, and the disruptions were confirmed by PCR with appropriate primers (data not shown) yielding the *gaf1Δ* strains, KL230, KL210, KL211, and KL216, respectively. *ste11Δ* strain JZ396 [kindly donated by Dr. Yamamoto [12]] was transformed with the *gaf1::kanMX* deletion cassette to yield the *gaf1Δ ste11Δ* double-deletion mutant (KL213). To construct a *gaf1Δ* strain carrying the *hphMX* marker, the *gaf1::kanMX* allele of KL210 strain was replaced with the 1.6-kb *gaf1::hphMX* deletion cassette amplified from pFA6a-hphMX6 [33]. Stable transformants were selected by resistance to hygromycin B and sensitivity to G418 (indicating loss of *kanMX*), and the disruptions were confirmed by PCR (data not shown) to yield strain KL240.

### Growth tests

To analyze growth by plate assays, *S. pombe* cells grown to mid-exponential phase in EMM2 for 18 h were washed and resuspended in EMM2-N (for nitrogen starvation) or EMM-G (for glucose restriction) to a concentration of 5×10^6^ cells ml^−1^. The cell suspensions were serially diluted in 5-fold steps, and then a 5 µl aliquot of each dilution was spotted onto EMM, EMM-N, and EMM-G plates. The plates were incubated at 30°C and photographed after 3 d.

### Preparation of total RNA

For preparation of total RNA, wild-type (ED668) and *gaf1Δ* (KL216) strains were grown to mid-log phase in EMM2 for 18 h at 30°C. Cells were harvested from the mid-log phase cultures and used as unstarved cell preparations (+N). To prepare nitrogen-starved cell samples (−N), cells harvested from the mid-log phase cultures in EMM2 were washed with distilled water and shifted to EMM-N. Nitrogen-starved cells used for microarray analysis were harvested 4-h cultivation in EMM-N. For Northern blot analysis, aliquots of the nitrogen-starved culture were removed at intervals, *i.e.*, at 0, 3, 6, 9, and 18 h after shift. The cells were washed twice with distilled water and frozen immediately at −70°C for total RNA preparation. Total RNA samples were extracted from approximately 2×10^8^ cells using a bead beater as described previously [12].

### Microarray analysis

Thirty-microgram total RNA of each sample was further purified using RNeasy (QIAGEN) columns and submitted for microarray analysis by the SeouLin Bioscience (Korea). Probes were generated and hybridized to the GeneChip Yeast Genome 2.0 Array (Affymetrix), and the data were analyzed using GeneSpring GX software (Agilent).

### Northern blot analysis

Approximately 20 µg of total RNA was fractionated on a 1% agarose gel containing 18% formaldehyde, transferred onto a Hybond-N^+^ membrane (Amersham), and fixed by UV cross-linking (Stratagene). DNA probes for *ste11^+^*, *gaf1^+^*, and actin (*act1* ^+^) genes were prepared using the PCR fragments amplified with the following primer pairs: P11 (5′GACCTGCGATCCAGATGATT3′) and P12 (5′CCAACAGCACTCTTGACGAA3′) for *ste11^+^*; P13 (5′TTACAACTTGCGTCCAGCA3′) and P14 (5′TGAATTCAGGAGCACCTTCC3′) for *gaf1^+^*; and P15 (5′GAAGCACACCATGACGCTTA3′) and P16 (5′CCTTGATCTCACCACAAGCA3′) for *act1* ^+^. The DNA probes were labeled with [*α*-^32^p]-dCTP using a Random Primed DNA Labeling Kit (Amersham). Hybridization was carried out at 42°C overnight in Rapid-hyb solution (Amersham). The signal was visualized by exposing the membrane to X-ray film and the relative signal intensity was quantified using a shareware program (Scion Image Beta 4.0.2).

### Flow cytometric analysis

For flow cytometry, cells grown to mid-log phase (5×10^6^ cells ml^−1^) were harvested and fixed in 70% ethanol containing 50 mM sodium citrate overnight at 4°C. After brief centrifugation, cell pellets were washed twice with 1 ml of 50 mM sodium citrate buffer (pH 7) and treated with RNase A (10 µg ml^−1^) at 37°C for 2 h. The cells were stained with propidium iodide (16 µg ml^−1^) and analyzed using a BD FACScalibur Flow Cytometer as described previously [28]. Data were analyzed using WinMDI software, version 2.8.

### Mating and sporulation assay

In order to monitor the efficiency of sporulation, *S. pombe* cells grown to mid-log phase were prepared by two successive transfers of young colonies on EMM2 agar plates. The cells were then collected and washed three-times with distilled water. Suspensions of homothallic haploid cells (*h^90^*) or mixtures of mating pairs (*h^+^* and *h^−^*) were spotted in 10 µl aliquots (1.0×10^9^ cells) onto EMM2 and EMM-N agar plates. Cultures were grown at 30°C, and the cells were observed at intervals by DIC microscopy to determine sporulation frequencies. At least 400 cells from three independent experiments were evaluated, and mating and sporulation frequencies (F_M_) were calculated using the following equation [34]: F_M_ (%) = (2Z+2A+0.5S)×100/(H+2Z+2A+0.5S), where Z stands for the number of zygotes, A for the number of asci, S for the number of free spores, and H for the number of cells that failed to mate. When necessary, sporulation was visualized by iodine vapor staining.

### β-Galactosidase reporter assay

*S. pombe* cells containing β-galactosidase reporter plasmids were pre-grown to mid-log phase in liquid EMM2 medium for 18 h and shifted to EMM2 or EMM-N medium. Cells were harvested at intervals by centrifugation, washed, and resuspended at a concentration of 5×10^6^ cells ml^−1^. After the cells were permeabilized with 0.1% sodium lauroylsarcosinate, β-galactosidase activity was determined by measuring hydrolysis of the chromogenic substrate, o-nitrophenyl-β-D-galactoside, as described previously [35].

### Electrophoretic mobility shift assay

To prepare GST and GST-Gaf1 fusion proteins for electrophoretic mobility shift assay (EMSA), cells of *Escherichia coli* BL21 strains carrying pGEX4T3 or pGEX4T3-Gaf1 were cultured in LB medium with 50 µg ml^−1^ of ampicillin at 30°C to *A*_600_ of 0.5. Isopropyl β-D-thiogalactopyranoside was added to a final concentration of 1 mM and the cells were grown for 4 h at 25°C. Harvested cells were resuspended in Buffer A (20 mM Tris, pH 7.6; 137 mM NaCl) containing 0.1% Tween 20, 1 mM phenylmethylsulfonyl fluoride, and 1 µg ml^−1^ lysozyme. Resuspended cells were lysed by sonication, and the lysate was cleared by centrifugation at 24,000×g for 20 min at 4°C. The protein extracts were purified through a Glutathione Sepharose 4B column (Amersham).

To prepare EMSA probes and cold competitors, the upstream 0.60-kb *Sph*I-*Nde*I (P_A_) and downstream 0.25-kb *Nde*I-*Eco*RV (P_B_) segments of the partial *ste11^+^* promoter region [4] were amplified by PCR from the genomic DNA of *S. pombe* 972 using the following primers: P17 (5′GCATGCCATCTCCAGGGA3′) and P18 (5′ACATATGATGCGAAAGCATT3′) for P_A_; and P19 (5′CATATGTTACTTTAACCCCT3′) and P20 (5′GGATATCCTTTTAATATATGCT3′) for P_B_. Specific double-stranded oligonucleotide probes for wild-type (P_W_) and mutant (P_M_) GATA motifs corresponding to nucleotides −385 to −352 of the *ste11^+^* promoter were prepared by annealing complementary pairs of the following single-stranded oligonucleotides: P21 (5′CATTTTGCCTTGCGCTATCTCCCTCAACGAAAAG3′) and P22 (5′CTTTTCGTTGAGGGAGATAGCGCAAGGCAAAATG3′) for P_W_; and P10 and P9 for P_M_. The duplex DNAs were end-labeled with [γ-^32^P] ATP by T_4_-polynucleotide kinase and purified using a G-50 or G-25 column (Amersham). All binding reactions were carried out in 20 µl binding buffer (10 mM Tris pH 7.5, 2 mM MgCl_2_, 50 mM NaCl, 1 mM DTT, 5% glycerol) containing 2 µg of poly (dI·dC) as a non-specific competitor, 0.1–1 µg of recombinant GST-Gaf1 protein, and 5 ρmol ^32^P-end-labeled double-stranded probe, at room temperature for 20 min. For competition experiments, DNA binding reactions were allowed to reach equilibrium and a 50- or 100-fold excess of unlabeled specific competitor DNA was added to the binding reaction mixture. For detection of a specific DNA-protein complex, samples were loaded onto a 6% non-denaturation polyacrylamide gel in 0.5×Tris-glycine buffer and electrophoresed at 10 V cm^−1^ at room temperature. The gels were run for 2 h, dried on a gel dryer and autoradiographed at −70°C using Fuji X-ray film. Putative binding sites for transcription factors were searched by using TRANSFAC program (http://gene-regulation.com/pub/databases.html).

## Results

### Deletion of *gaf1^+^* causes accelerated G_1_-arrest under nitrogen-starved conditions

Previously, we suggested that *gaf1^+^* might function as a transcriptional activator, although its biological relevance was unclear as a deletion mutant showed no significant defects [18]. However, recent genome analysis revealed that the *gaf1^+^* sequence previously reported (Accession No. AAC35593) contains only a partial ORF corresponding to the C-terminal 290-amino acid segment of Gaf1, and that the complete *gaf1^+^* sequence comprises a 2,565-bp ORF encoding an 855-amino acid protein (Accession No. Q10280) [36].

**Figure 1:**
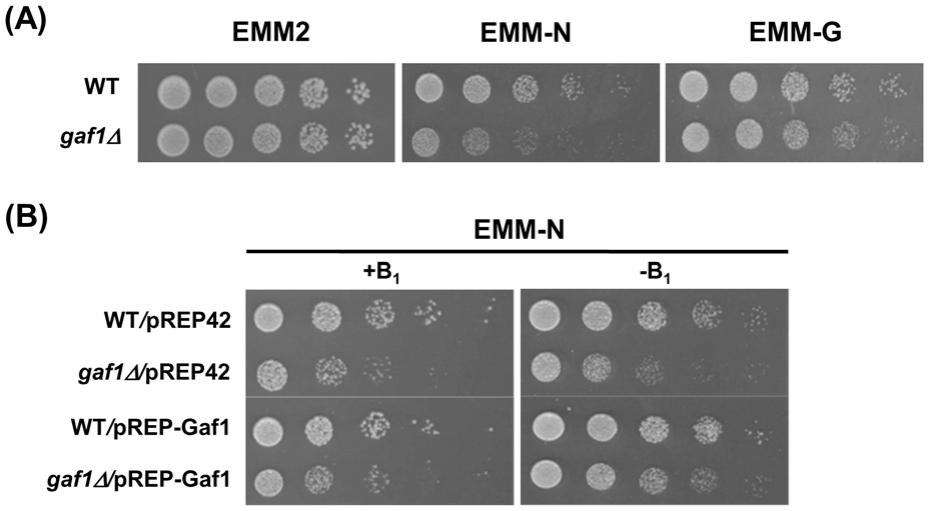
Deletion of *gaf1^+^* causes reduced growth on nitrogen-starved medium. Cells grown to mid-log phase in EMM2 for 18 h were serially diluted 5-fold and spotted on agar plates. The plates were incubated at 30°C for 3 d. (A) Cells of WT (972) and *gaf1Δ* (KL230) strains were spotted on EMM, EMM-N, and EMM-G agar plates. (B) Cells of WT(ED665)/pREP42, *gaf1Δ*(KL210)/pREP42, WT(ED665)/pREP42-Gaf1, and *gaf1Δ*(KL210)/pREP-Gaf1 strains were spotted onto EMM-N plates with (+B_1_) or without (−B_1_) 20 µM thiamine. WT denotes the wild-type (*gaf1^+^*).

In the present study, we deleted the entire *gaf1^+^* ORF from the genome of *S. pombe* (Table 1) and evaluated the response of *gaf1Δ* cells to nitrogen starvation and glucose restriction by plate assay. Cells of the *gaf1Δ* strain did not show any growth defect on EMM2 or EMM-G plate, indicating that the *gaf1^+^* gene is dispensable for mitotic growth under normal and glucose-restricted conditions (Figure 1A). However, *gaf1Δ* cells showed significantly reduced growth on EMM-N which lacked the inorganic nitrogen source, NH_4_Cl, compared to wild-type cells that did grow to a limited extent by utilizing the supplementary auxotrophic nutrients, adenine, leucine, and uracil, as organic nitrogen sources.

To confirm that the sensitivity of the *gaf1Δ* mutant to nitrogen starvation is due to the loss of Gaf1 activity rather than acquisition of abnormal activity, we constructed a system in which the production of Gaf1 could be shut off artificially using the thiamine-repressible *nmt42^+^* promoter. In the absence of thiamine, the *gaf1Δ*/pREP-Gaf1 cells carrying ectopic copies of *gaf1^+^* under the control of the *nmt42^+^* promoter exhibited growth similar to the wild-type strains (WT/pREP42 and WT/pREP-Gaf1) on EMM-N plates (Figure 1B). In the presence of thiamine, *gaf1Δ*/pREP-Gaf1 cells exhibited as poor growth on EMM-N medium as *gaf1Δ*/pREP42 cells. These results suggest that the *gaf1^+^* gene is dispensable for mitotic growth under normal conditions, but apparently plays a significant role in sustaining growth, though to a limited extent, under nitrogen-starved conditions.

**Figure 2:**
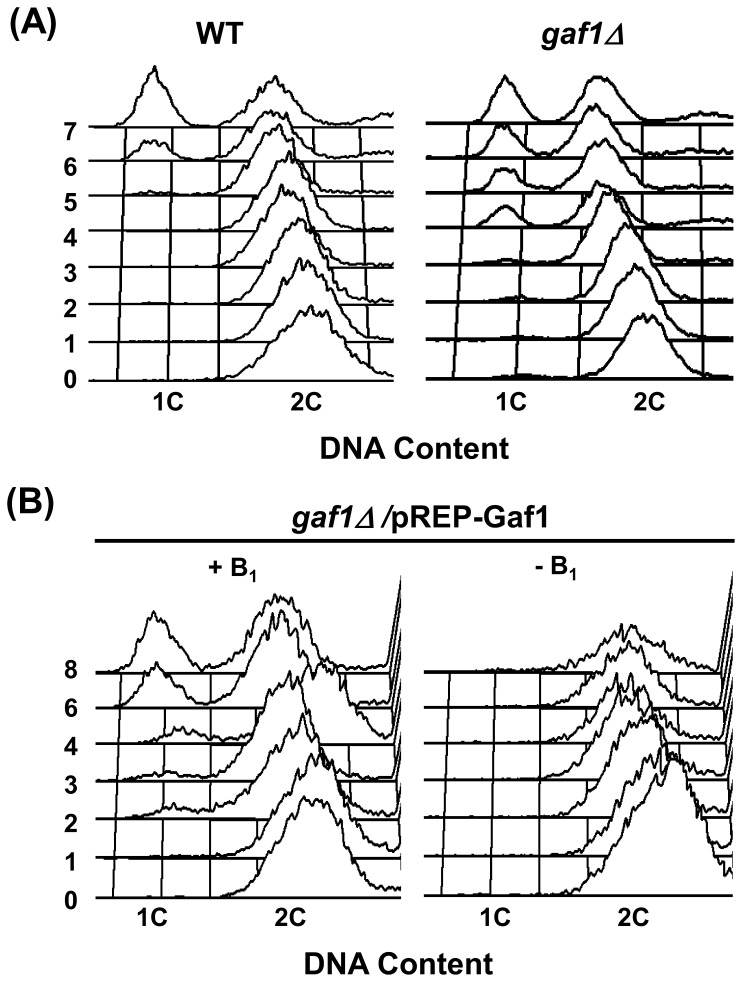
Deletion of *gaf1^+^* causes accelerated G_1_-arrest under nitrogen-starved conditions. Cells grown to mid-log phase in EMM2 for 18 h were shifted to EMM-N medium, and their DNA contents were monitored by FACS analysis at intervals. The FACS data represent typical examples of three independent experiments. (A) Cells of heterothallic (*h^−^*) WT (972) and *gaf1Δ* (KL230) strains were shifted to EMM-N medium. (B) Cells of homothallic (*h^90^*) *gaf1Δ*(KL211)/pREP-Gaf1 strain were shifted to EMM-N medium with (+B_1_) or without (−B_1_) 20 µM thiamine. WT denotes the wild-type (*gaf1^+^*).

In *S. pombe*, as key nutrients become limited, cells exit the mitotic cycle and enter either G_0_ stationary phase or a program of sexual differentiation [37]. During early nitrogen starvation, *S. pombe* cells undergo several rounds of rapid cell division and then arrest at the G_1_ phase [8], [13]. In this study, the *gaf1Δ* strain accumulated G_1_-arrested cells after 4 h of nitrogen starvation, but the wild-type strain did not accumulate any detectable amount of G_1_-arrested cells until 6 h after the nitrogen shift (Figure 2A). Correspondingly, the homothallic haploid strain *h^90^ gaf1Δ*/pREP-Gaf1 began to accumulate G_1_-arrested cells after 2 h of nitrogen starvation in the presence of thiamine, however, it did not show any signs of G_1_-arrest even after 8 h of nitrogen starvation in the absence of thiamine (Figure 2B). Therefore, the deletion of *gaf1^+^* causes accelerated entrance into G_1_ under nitrogen-starved conditions. Accordingly, the function of *gaf1^+^* might be to delay the shift of nitrogen-starved cells from the vegetative cycle to the meiotic cycle, helping to sustain the vegetative cycle upon transient nitrogen starvation.

### Sporulation efficiency is enhanced by *gaf1Δ* mutation and reduced by *gaf1^+^* overexpression

To evaluate the function of *gaf1^+^* in sporulation under nitrogen-starved conditions, we spotted suspensions of pre-grown homothallic haploid cells (*h^90^*) and mixtures of mating pairs (*h^+^* and *h^−^*) with or without *gaf1Δ* on EMM2 (+N) and EMM-N (−N) agar plates and estimated their mating and sporulation efficiencies (F_M_) after 3-d cultivation. The homothallic haploid *gaf1Δ* strain showed a significantly higher F_M_ value (24%) than the wild-type strain (7%) on EMM2 plates (Table 2). In addition, the F_M_ value of the *h^+^ gaf1Δ×h^−^ gaf1Δ* mating mixture (63%) was approximately 5-fold higher than the *h^+^×h^−^* mating pair of wild-type (13%) on EMM2 plates. The homothallic *gaf1Δ* strain (85%) and *h^+^ gaf1Δ×h^−^ gaf1Δ* mating mixture (80%) showed approximately 10–15% higher F_M_ values than the homothallic wild-type strain (69%) and *h^+^×h^−^* mating mixture of wild-type strains (71%) on EMM-N plates. These results indicate that the *gaf1Δ* mutation elevates mating and sporulation efficiency by making cells more sensitive to nitrogen starvation.

**Table 2:** Mating and sporulation frequency of *S. pombe* strains.

	Mating and sporulation frequency (%)^b^	
Strain genotype^a^	+N	−N
*h^90^*	6.5±2.1	69.2±6.1
*h^90^ gaf1Δ*	24.0±1.2	85.3±4.4
*h^+^*×*h^−^*	12.7±2.5	71.1±7.4
*h^+^ gaf1Δ*×*h^−^ gaf1Δ*	62.5±2.9	80.0±9.6
*h^+^ ste11Δ*×*h^−^ gaf1Δ*	<0.01	<0.01
*h^90^ ste11Δ*	<0.01	<0.01
*h^90^ gaf1Δ ste11Δ*	<0.01	<0.01

a For analysis of homothallic strains, pre-grown cultures of *h^90^* WT (JY4), *h^90^ gaf1Δ* (KL211), *h^90^ ste11Δ* (JZ396), and *h^90^ gaf1Δ ste11Δ* (KL213) were spotted onto EMM2 (+N) and EMM-N (−N) plates, and the cells were observed by DIC microscopy to determine sporulation frequencies after 3-d incubation at 30°C. For analysis of heterothallic strains, 1∶1 mixtures of the pre-grown mating pairs, *h^+^* (ED668)×*h ^−^* (ED665), *h^+^ gaf1Δ* (KL216)×*h* ***^−^*** *gaf1Δ* (KL210), and *h^+^ ste11Δ* (KL416)×*h^−^ gaf1Δ* (KL240) were spotted onto EMM2 and EMM-N plates.

b The values represent the average ± the standard deviation of at least three independent assays carried out in triplicate.

**Figure 3:**
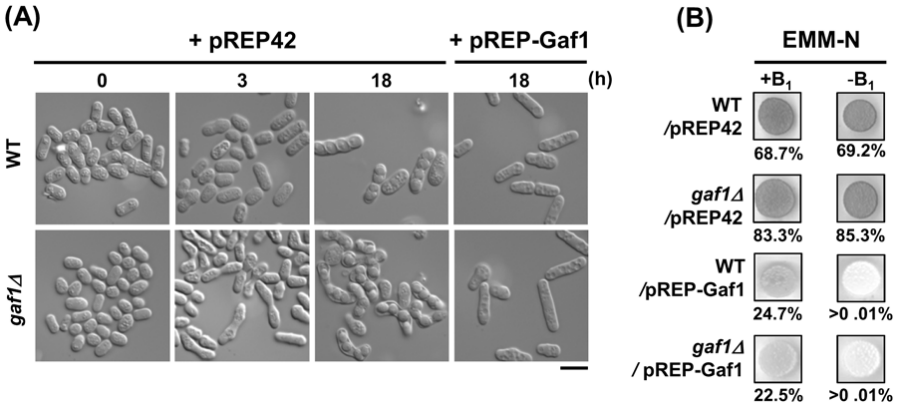
Overexpression of *gaf1^+^* results in reduction of sporulation efficiency. Cells and colonies of the homothallic (*h^90^*) strains, WT(JY4)/pREP42, *gaf1Δ*(KL211)/pREP42, WT(JY4)/pREP-Gaf1, and *gaf1Δ*(KL211)/pREP-Gaf1, were analyzed for sporulation by DIC microscopy and iodine staining, respectively. (A) Cells were grown to mid-log phase in EMM2 for 18 h and shifted to EMM-N medium. Samples were taken from the cultures at intervals, and the morphological characteristics of cells were observed by DIC microscopy. Bar, 10 µm. (B) Cells were grown to mid-log phase in EMM2 for 18 h and spotted onto EMM-N plates with (+B_1_) or without (−B_1_) 20 µM thiamine. Sporulation was monitored by iodine vapor staining of colonies after 3-d incubation at 30°C. The F_M_ value presented under each panel was determined by observing the cells under a DIC microscope. The values represent the average of at least three independent assays carried out in triplicate. WT denotes the wild-type (*gaf1^+^*).

We also evaluated the role of *gaf1^+^* in sporulation using an overexpression system of *gaf1^+^* under the control of the *nmt42^+^* promoter in homothallic strains. In liquid EMM-N medium, no sporulation was observed among *h^90^ gaf1Δ/*pREP-Gaf1 and *h^90^* WT/pREP-Gaf1 cells even after 18-h exposure to nitrogen-starved conditions, while a considerable portion of the *h^90^ gaf1Δ*/pREP42 and *h^90^* WT/pREP42 cells showed mating behavior after 3- and 18-h exposure, respectively (Figure 3A). On EMM-N plates, the *h^90^* WT/pREP-Gaf1 and *h^90^ gaf1Δ*/pREP-Gaf1 strains exhibited negligible levels of iodine staining and F_M_ value (<0.01%) in the absence of thiamine, but moderate levels of iodine staining and F_M_ values (23–25%) in the presence of thiamine (Figure 3B). On the contrary, the spots of *h^90^* WT/pREP42 and *h^90^ gaf1Δ*/pREP42 strains were stained dark brown with iodine vapor and exhibited high F_M_ values (69–85%) after 3-d cultivation regardless of the presence of thiamine, indicating luxuriant sporulation under nitrogen-starved conditions. Together, these results demonstrate that overexpression of *gaf1^+^* leads to a significant reduction of sporulation efficiency.

### Gaf1 controls both the nitrogen starvation- and pheromone-responsive genes

**Figure 4:**
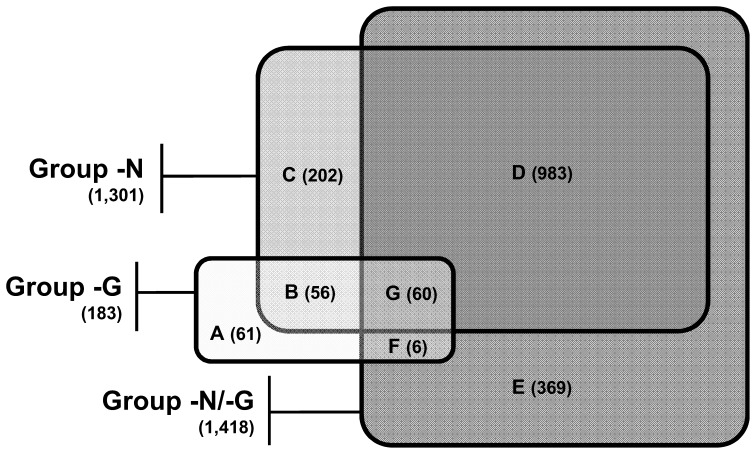
Effect of *gaf1Δ* mutation and nitrogen starvation on the global gene expression profiles of *S. pombe*. RNA samples from the nitrogen-starved (−N) and unstarved (+N) cells of wild-type (*gaf1^+^*, ED668) and *gaf1Δ* (KL216) strains were used for the transcriptome analysis with the GeneChip Yeast Genome 2.0 Array. A Venn diagram is constructed from the lists of the genes up-regulated (≥1.5-fold, p<0.05) in unstarved *gaf1Δ* cells (Group −G, Table S1), nitrogen-starved wild-type cells (Group −N, Table S2), and nitrogen-starved *gaf1Δ* cells (Group −N/−G, Table S3). The overlapping and non-overlapping portions of the three groups (Group −G, −N, and −N/−G) are designated as Subgroup A to G. The lists of the genes included in Subgroup A–G are provided in Tables S4, S5, S6, S7, S8 S9, and S10, respectively, in the Supporting Information.

To search for a set of genes whose expression is specifically altered in response to the loss of *gaf1^+^*, microarray-based transcriptome analysis was performed using the RNA samples from the nitrogen-starved (−N) and unstarved (+N) cells of wild-type (*gaf1^+^*, ED668) and *gaf1Δ* (KL216) strains. A Venn diagram (Figure 4) was constructed from the groups of the genes up-regulated (≥1.5-fold, p<0.05) in unstarved *gaf1Δ* cells (Group −G), nitrogen-starved wild-type cells (Group −N), and nitrogen-starved *gaf1Δ* cells (Group −N/−G). One hundred and eighty-three genes were up-regulated in unstarved *gaf1Δ* cells (Group −G, Table S1). A total of 1,301 and 1,418 genes were up-regulated by nitrogen starvation in wild-type (Group −N, Table S2), and *gaf1Δ* cells (Group −N/−G, Table S3).

The overlapping and non-overlapping portions of the three groups (Group −G, −N, and −N/−G) are designated as Subgroup A to G (Figure 4), which enables more detailed clustering of the genes of interest. Approximately 63% (116 genes) of the members of Group −G were up-regulated by both *gaf1Δ* mutation and nitrogen starvation (Subgroup B+G, Table S5 and S10), while most of the remainders (61 genes) were up-regulated solely by *gaf1Δ* mutation (Subgroup A, Table S4). Therefore, it is suggested that Gaf1 down-regulates the basal expression levels of the nitrogen starvation-responsive genes included in Subgroup B+G. Although majorities of the members in Group −N and −N/−G were shown to be up-regulated by nitrogen starvation in both *gaf1Δ* and wild-type cells (1,043 genes, Subgroup D+G, Table S7 and S10), a considerable portions of them were up-regulated by nitrogen starvation in either *gaf1Δ* (375 genes, Subgroup E+F, Table S8 and S9) or wild-type cells (262 genes, Subgroup B+C, Table S5 and S6).

A considerable number of the genes of Subgroup B+G are up-regulated during mating and sporulation (the Sanger Centre Database; http://www.genedb.org/genedb/pombe) [38]: genes involved in nitrogen and/or pheromone response such as *mei2* ^+^, *ste11^+^*, *spk1* ^+^, *ppk33* ^+^, *ste4* ^+^, *gpa1* ^+^, *mfm1* ^+^, *map1* ^+^, *map2* ^+^ and *map3* ^+^; genes function in double-strand break (DSB) formation, meiotic recombination, and/or nuclear segregation such as *mug8* ^+^, *mug14* ^+^, *mug24* ^+^, *mug55* ^+^, *mug112* ^+^, *mug133* ^+^, *rec24* ^+^, *bqt2* ^+^, and *moa1* ^+^ [39]; genes encoding permeases (5 genes) and transporters (15 genes) for amino acids, sugars, urea, and other nutrients; and 5 *wtf* genes belonging to Tf transposon-containing sequences that are transcribed during meiosis. Taken together, it is suggested that deletion of *gaf1^+^* may result in cellular physiology similar to one induced by nitrogen starvation and, accordingly, that Gaf1 plays an important role in both nitrogen starvation and mating response.

Furthermore, by comparing our data with the result of microarray analysis using the wild-type, Ste11-overexpressing, and *ste11Δ* strains [40], we found that 25 genes among those induced by *gaf1Δ* mutation are included in the list of the 61 Ste11 target genes (Table 3). Among the genes exhibiting increased expression in *gaf1Δ* cells, 10 genes including *mei2^+^*, *spk1^+^*, and *ste11^+^* were up-regulated even under unstarved conditions (Subgroup G), while the remainders (15 genes) including *ran1^+^*, *tht1^+^* and *ste6^+^* were induced only under nitrogen-starved conditions (Subgroup D+E). This result supports the speculation that *ste11^+^* is possibly a direct target of Gaf1 that negatively regulates its expression at the transcriptional level.

**Table 3:** List of the Ste11 target genes up-regulated by *gaf1*Δ mutation.

				Expression ratio^a^		
Subgroup	Systematic No.	Gene	Description (GeneDB)	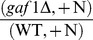	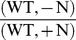	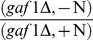
(G)^b^	SPAC27D7.03c	*mei2* ^+^	RNA-binding protein involved in meiosis	4.07	43.74	9.30
	SPAC32A11.01	*mug8* ^+^	conserved fungal protein	2.27	5.82	4.25
	SPAC31G5.09c	*spk1* ^+^	MAP kinase	1.95	36.46	23.90
	SPBC32C12.02	*ste11* ^+^	transcription factor	1.76	5.87	3.10
	SPBC359.06	*mug14* ^+^	adducing	1.73	82.76	45.98
	SPAC11E3.06	*map1* ^+^	MADS-box transcription factor	1.72	11.77	6.14
	SPBC19C2.04c	*ubp11* ^+^	ubiquitin C-terminal hydrolase	1.72	5.07	3.84
	SPBC24C6.06	*gpa1* ^+^	G-protein alpha subunit	1.70	11.54	7.32
	SPAC1565.04c	*ste4* ^+^	adaptor protein	1.69	10.87	5.41
	SPCC162.10	*ppk33* ^+^	serine/threonine protein kinase	1.53	46.35	30.03
(D+E)^c^	SPAC1F5.09c	*shk2* ^+^	PAK-related kinase	1.27	20.72	53.30
	SPCC1442.01	*ste6* ^+^	guanyl-nucleotide exchange factor	0.98	7.40	15.76
	SPAC1093.06c	*dhc1* ^+^	dynein heavy chain	0.30	1.47	15.66
	SPAC23E2.03c	*ste7* ^+^	meiotic suppressor protein	1.19	11.05	15.28
	SPBC19C2.05	*ran1* ^+^	serine/threonine protein kinase	1.18	5.38	6.88
	SPCC1393.07c	*mug4* ^+^	sequence orphan	1.17	1.97	5.57
	SPAC31G5.07		conjugation protein (predicted)	0.89	1.52	4.45
	SPBC354.08c		DUF221 family protein	0.36	0.92	4.07
	SPBC2D10.06	*rep1* ^+^	MBF transcription factor complex subunit	0.92	2.00	3.36
	SPAC13C5.03	*tht1* ^+^	nuclear membrane protein involved in karyogamy	1.04	0.62	3.01
	SPAC1F5.08c	*yam8* ^+^	calcium transport protein	1.07	1.55	2.65
	SPAC27E2.07	*pvg2* ^+^	galactose residue biosynthesis protein	1.21	1.59	2.48
	SPAPB2B4.03	*cig2* ^+^	cyclin	0.80	1.09	2.39
	SPAC31G5.10	*eta2* ^+^	Myb family protein	1.04	1.41	2.22
	SPBP4H10.11c	*lcf2* ^+^	long-chain-fatty-acid-CoA ligase	0.75	1.02	2.08

a Relative expression of the genes was measured by microarray assay in the nitrogen-starved (−N) and unstarved (+N) cells of wild-type (ED668) and *gaf1Δ* (KL216) strains. Unstarved (+N) cells were prepared from the mid-log phase cultures in EMM2. Nitrogen-starved (−N) cells were prepared by shifting the mid-log phase cells to EMM-N for 4 h as described in Materials and Methods.

b Genes up-regulated by *gaf1*Δ mutation under both nitrogen-starved and unstarved conditions (Subgroup G, *p*<0.05).

c Genes up-regulated by *gaf1*Δ mutation only under nitrogen starved conditions (Subgroup D+E, *p*<0.05).

### *ste11^+^* is epistatic to *gaf1^+^*

Epistasis analysis was performed to determine whether Gaf1 functions in the same pathway as Ste11, a key transcription factor for sexual development [12]. Pre-grown cells of the homothallic haploid strains, *h^90^ gaf1Δ*, *h^90^ ste11Δ*, and *h^90^ gaf1Δ ste11Δ*, were spotted on both EMM2 and EMM-N plates and the sporulation efficiencies were monitored after 3 d. The F_M_ value of *h^90^ gaf1Δ* reached approximately 85% on EMM-N and 24% on EMM2, but *h^90^ ste11Δ* and *h^90^ gaf1Δ ste11Δ* cells, and the *h^+^ ste11Δ*×*h^−^ gaf1Δ* mating mixture exhibited negligible levels of sporulation efficiencies even on EMM-N (Table 2). Therefore, the phenotype of the *gaf1Δ* mutation, *i.e.*, accelerated initiation of sporulation and elevated sporulation efficiency, is masked by the *ste11Δ* mutation, causing impaired sporulation. Thus, *ste11^+^* is epistatic to *gaf1^+^* with respect to sporulation efficiency, and furthermore, *gaf1^+^* functions upstream of *ste11^+^* in the signaling pathway governing sexual development.

### Expression of *ste11^+^* is accelerated and increased in *gaf1Δ* cells

**Figure 5:**
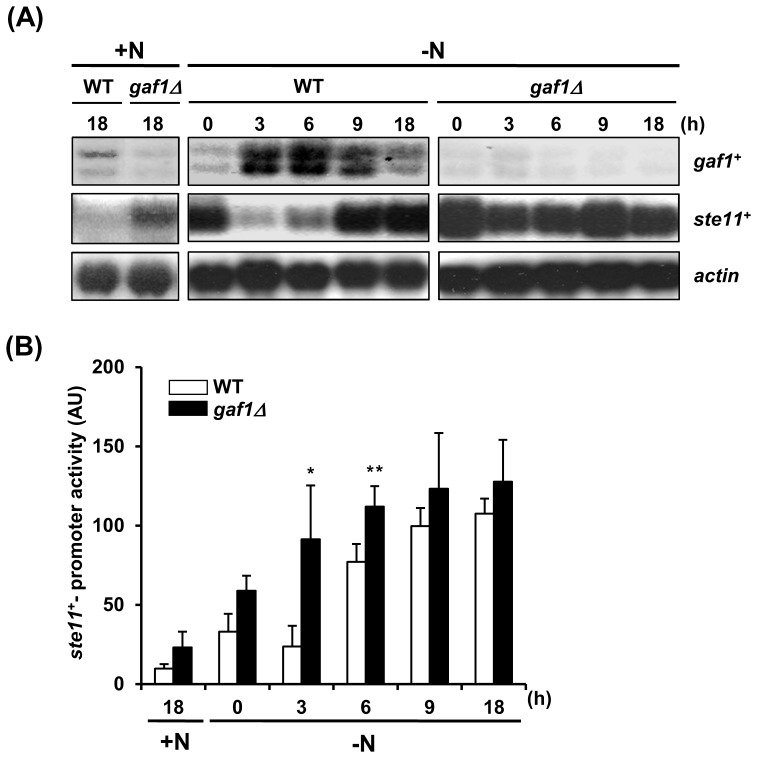
Deletion of *gaf1^+^* results in accelerated induction of *ste11^+^* expression under nitrogen-starved conditions. (A) Northern blot analysis of *ste11^+^* and *gaf1^+^* mRNA from wild-type and *gaf1Δ* cells exposed to nitrogen starvation. Cells of wild-type (972) and *gaf1Δ* (KL230) strains pre-grown in EMM2 (+N) were shifted to EMM-N (−N) and cultured with constant shaking. At indicated time points, cells were harvested and washed twice with distilled water, and total RNAs were extracted from the cells. RNA blots were hybridized with ^32^P-labeled PCR-amplified *gaf1^+^* and *ste11^+^* probes. For internal control, all blots were stripped and subsequently rehybridized with ^32^P-labeled actin-specific probe (*act1^+^*). (B) β-Galactosidase reporter assay for analysis of *ste11^+^* expression in wild-type and *gaf1Δ* cells subjected to nitrogen starvation. Cells of wild-type (ED665) and *gaf1Δ* (KL240) strains carrying pJLC-Ste11_(p)_-LacZ were cultivated to mid-log phase in EMM2 (+N) and shifted to EMM-N (−N). At indicated time points, cells were harvested and washed twice with distilled water, and the level of *ste11* ^+^ expression was estimated by measuring the activity of β-galactosidase in each sample. Values are the mean ± standard error of three independent experiments carried out in triplicate, *n* = 9. *, *p*<0.01; **, *p*<0.05 (two-tailed Student's t-test, *versus* wild-type). WT denotes the wild-type (*gaf1^+^*).

To test whether *gaf1^+^* is responsible for the transcriptional regulation of *ste11^+^*, we analyzed the expression of *ste11^+^* under both nitrogen-starved and normal conditions in *gaf1Δ* and wild-type strains by Northern blot analysis. Under normal conditions, *gaf1Δ* cells showed increased level of *ste11^+^* compared to wild-type cells (Figure 5A). When the wild-type cells were exposed to nitrogen starvation, the level of *gaf1^+^* transcript increased considerably during the first 6 h of nitrogen starvation, followed by a subsequent decline. Unexpectedly, the levels of *ste11^+^* transcript in both the wild-type and *gaf1Δ* cells harvested from the nitrogen-starved cultures at the time point of 0 h were considerably higher than those in the corresponding cells from the unstarved culture, which might be due to the very short exposure of the cells to EMM-N medium followed by washing with distilled water. This result is similar to that observed in the previous study conducted by other research group [41]. Therefore, we adopted the data from the unstarved cells of corresponding strains cultivated in EMM2 for 18 h, rather than those from the nitrogen-starved cells sampled at the time point of 0 h, as references. As shown in Figure 5A, the amount of *ste11^+^* transcript increased at a much slower rate than that of *gaf1^+^* transcript, and it did not reach its highest level until at least 9 h after the transfer. The level of *ste11^+^* transcript in *gaf1Δ* cells, however, increased steeply up to the plateau within 3 h and was maintained during the following 6 h. The expression of *ste11^+^* was also monitored by assaying the ectopic expression of *Ste11_(p)_-lacZ* hybrid gene in wild-type and *gaf1Δ* strains carrying the pJLC-Ste11_(p)_-LacZ plasmid. In accordance with the result of Northern blotting, the expression of *Ste11_(p)_-lacZ* was higher in *gaf1Δ* cells than in wild-type cells under unstarved conditions (Figure 5B). When cells were subjected to nitrogen starvation, the expression of *Ste11_(p)_-lacZ* was induced more rapidly in *gaf1Δ* cells than in wild-type cells. These results suggest that deletion of *gaf1^+^* causes accelerated induction of *ste11^+^* expression under nitrogen-starved conditions and increased *ste11^+^* expression even under unstarved conditions.

### Gaf1 protein binds to the promoter region of *ste11^+^*

**Figure 6:**
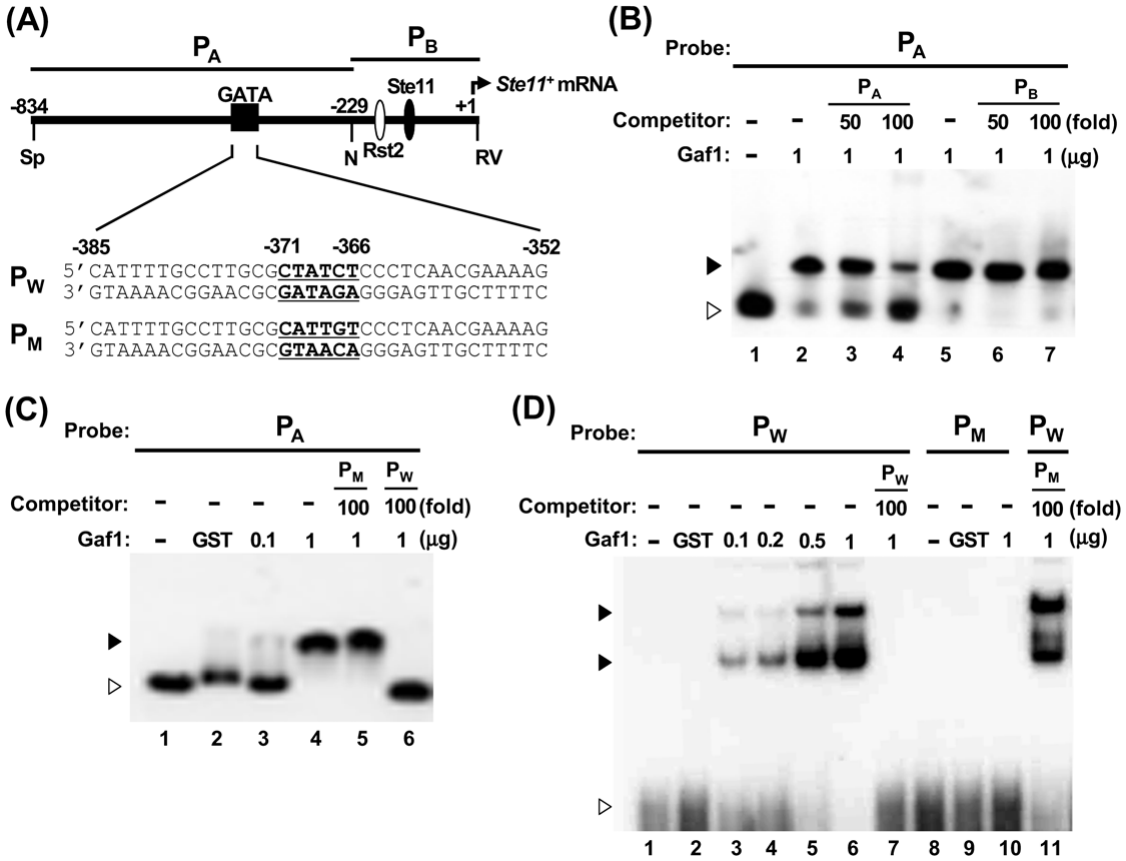
Gaf1 protein specifically binds to the GATA binding motif of *ste11^+^* promoter *in vitro*. (A) Schematic diagram of the promoter region of *ste11^+^*. The open- and closed-ovals represent binding sites for Rst2 (UASst) and Ste11 (TR1 and TR2), respectively. The major transcription start site (position number +1) is designated by a hinged arrow. The regions contained in the upstream probe P_A_ (−834∼−225) and the downstream probe P_B_ (−231∼+10) are shown as bars. The dark square represents the GATA motif spanning from −385 to −352 for which wild-type (P_W_) and mutant (P_M_) double-stranded oligonucleotide probes were designed. Restriction sites: Sp, *Sph*I; N, *Nde*I; RV, *Eco*RV. (B) Search for Gaf1-binding region in *ste11^+^* promoter by EMSA. GST-Gaf1 protein (1 µg) was incubated with labeled P_A_ probe containing the 0.6-kb upstream region of *ste11^+^* promoter in the absence (lanes 2 and 5) or presence of excess cold competitor, P_A_ (lane 3, 50-fold; lane 4, 100-fold) or P_B_ (lane 6, 50-fold; lane 7, 100-fold). (C) Analysis of Gaf1 binding to the GATA motif of *ste11^+^* promoter by EMSA. GST-Gaf1 protein (lane 3, 0.1 µg; lanes 4–6, 1 µg) was incubated with labeled P_A_ probe in the absence (lanes 3 and 4) or presence of 100-fold excess of cold competitor P_M_ (lanes 5) or P_W_ (lanes 6). (D) Mutational dissection of the Gaf1-binding GATA motif of *ste11^+^* promoter by EMSA. Varying amounts of GST-Gaf1 protein (lane 3, 0.1 µg; lane 4, 0.2 µg; lane 5, 0.5 µg; lanes 6, 7, 11, and 12, 1 µg) were incubated with labeled P_W_ oligonucleotide probe in the absence (lanes 3 and 4) or presence of 100-fold excess of cold competitor P_M_ (lanes 5) or P_W_ (lanes 6). Mock reaction mixtures without GST-Gaf1 or with GST protein were used as negative controls in (B)–(D). The closed and open arrow heads in (B)–(D) represent shifted bands and free probes, respectively.

We performed EMSA to address whether Gaf1 can directly bind to the promoter region of *ste11^+^*. The upstream probe (P_A_), encompassing the region from −835 to −227 of the *ste11^+^* promoter, was amplified by PCR and end-labeled with T4 polynucleotide kinase (Figure 6A). A DNA-protein complex was observed in the reaction mixture containing the ^32^P-labeled P_A_ probe and the recombinant GST-Gaf1 (Figure 6B, lanes 2 and 5). The DNA-protein complex was specifically reduced in the presence of 50- and 100-fold molar excess of cold P_A_ competitor probe (Figure 6B, lanes 3 and 4), but not in the presence of 50- or 100-fold molar excess of cold P_B_ competitor (Figure 6B, lanes 6 and 7). This suggests that the Gaf1 protein specifically binds to a *cis*-element contained in the upstream region (from −828 to −227) of the *ste11^+^* promoter.

We also performed competition experiments using cold oligonucleotide probes spanning base pairs −385 to −351: P_W_ containing a canonical GATA motif and P_M_ containing mutations in the GATA motif (Figure 6A). The DNA-protein complex between the ^32^P-labeled P_A_ probe and GST-Gaf1 protein was diminished by the addition of 100-fold molar excess cold P_W_ (Figure 6C, lanes 4 and 6), but not by a similar amount of cold P_M_ (Figure 6C, lanes 4 and 5). Accordingly, the amount of P_W_-GST-Gaf1 complex increased in proportion to the amount of GST-Gaf1 (Figure 6D, lanes 3–6) and was diminished to the basal level by addition of 100-fold molar excess of cold P_W_ (Figure 6C, lane 6). The P_M_ probe did not produce any detectable amount of DNA-protein complex with GST-Gaf1 (Figure 6D, lane 10) or exhibit competition against the P_W_ probe to GST-Gaf1 even at a 100-fold molar excess (Figure 6D, lane 11). These results reflect that the canonical GATA motif from −371 to −366 in the promoter of *ste11^+^* (Figure 6A) is the target sequence of the Gaf1 protein. It is thus suggested that the GATA motif in *ste11^+^* promoter functions as a *cis* element to delay and attenuate the induction of *ste11^+^* expression via the interaction with Gaf1.

## Discussion

The *S. pombe* protein Ste11, which activates a number of genes required for mating and meiosis, is a pivotal regulator of sexual differentiation induced by nutrient starvation or environmental stress [12]. In the present study, we identified Gaf1, an *S. pombe* GATA factor, as a negative regulator of *ste11^+^* expression.

Deletion of *gaf1^+^* caused no growth defects under normal conditions. However, under nitrogen-starvation, it led to reduced mitotic growth (Figure 1), accelerated entrance into G_1_ (Figure 2), and elevated mating and sporulation efficiency (Table 2). Overexpression of *gaf1^+^* resulted in a remarkable reduction of sporulation efficiency under nitrogen-starved conditions (Figure 3). It seems likely that Gaf1 functions as a modulator of the mitosis-meiosis transition, delaying the entrance of growing cells into the meiotic cycle during the initial stages of nitrogen starvation and signaling the optimal time for promoting sexual development. The delay in G_1_-arrest and subsequent sporulation may provide a safety mechanism allowing cells to revert to vegetative growth when nutrient availability again becomes favorable [6], [16], [42], [43]. In accordance with the present result, overexpression of *tor2^+^* encoding the TOR protein kinase Tor2 strongly represses mating, meiosis and sporulation efficiency, whereas its inactivation has the opposite effect, leading to cell differentiation regardless of nutritional conditions [11]. In *S. cerevisiae*, it has been shown that Tor kinase and GATA transcription factor are involved in nitrogen catabolite repression (NCR), a regulatory event in which transcription of certain genes is down-regulated by a good nitrogen source such as glutamine but up-regulated by a poor nitrogen source such as proline [44]. Therefore, the involvement of TOR kinase and GATA transcription factor in nitrogen signaling may be a widely conserved phenomenon among various organisms.

Microarray analysis of the global gene expression profiles in *gaf1Δ* and wild-type cells under nitrogen-starved and unstarved conditions enabled us to search for a cluster of genes controlled by the action of Gaf1. Approximately 63% of the genes induced by the deletion of *gaf1^+^* under normal conditions (Subgroup B+G, Figure 4) overlap with those induced by nitrogen starvation in wild-type cells. In addition, many of the Subgroup B+G genes are identified to be induced during mating and sporulation [38]. Thus, it is likely that Gaf1 plays an important role not only in nitrogen signaling pathway but also in mating response. In accordance with the present result, recent microarray analysis using temperature-sensitive *tor2* mutants revealed that a total of 151 of 194 genes induced by the loss of Tor2 function are included in the list of roughly 1,000 genes found to be induced by nitrogen starvation in *S. pombe* [45]. This result pointed to an important role that Tor2 plays in nitrogen starvation and mating response. Interestingly, we also found that 13 genes among those induced by the deletion of *gaf1^+^* overlap with those induced by the loss of Tor2 function, suggesting that the two genes might be involve in the same signaling pathway activated by nitrogen starvation. Thus, it would be interesting to determine the relationship between *gaf1^+^* and *tor2^+^* genes.

Comparison of our microarray data with the genome-wide view of Ste11 target genes reported previously [40] enabled us to speculate that *ste11* ^+^ is a strong candidate for a direct transcriptional target of Gaf1 (Table 3). Especially, it is noteworthy that the genes involved in Ras/MAPK signaling pathway stimulated by pheromone such as *gpa1* ^+^, *ste4* ^+^, *spk1* ^+^, *ste11* ^+^, and *mei2* ^+^ are transcriptionally induced in response to the loss of *gaf1* ^+^ function under normal conditions. In addition, this finding is in agreement with the observation that deletion of *gaf1^+^* causes accelerated entrance of cells into meiotic cell cycle (Figure 2) and elevated mating and sporulation efficiency on exposure to nitrogen starvation (Table 2).

In accordant with the result of microarray analysis, cells of the *gaf1Δ ste11Δ* strain were completely defective in mating and sporulation (Table 2), indicating that *ste11* ^+^ is epistatic to *gaf1^+^*. Deletion of *gaf1^+^* not only increases the expression of *ste11^+^* in unstarved cells but also accelerates the induction of *ste11^+^* transcription in nitrogen-starved cells (Figure 5). In addition, the result of EMSA provides compelling evidence that Gaf1 binds to the canonical GATA motif (5′-HGATAR-3′) spanning from −371 to −366 in the promoter of *ste11^+^* to attenuate and delay its expression (Figure 5). Thus, it becomes evident that Gaf1 functions as a negative regulator of *ste11^+^* transcription, via direct interaction with the GATA motif in the *ste11^+^* promoter. The expression of *ste11^+^* is regulated directly by two transcription factors, Rst2 and Ste11, that bind to the upstream activating sequence (UASst; 5′-CCCCTC-3′) and the T-rich box (TR box; 5′-TTCTTTGTTY-3′) in the *ste11^+^* promoter, respectively [4]. No other proteins that either activate or repress the transcription of *ste11^+^* through direct binding to its promoter had been identified. Our research indicates that Gaf1 provides the prime example for negative regulation of *ste11^+^* transcription through direct binding to a *cis*-acting motif of its promoter.

**Figure 7:**
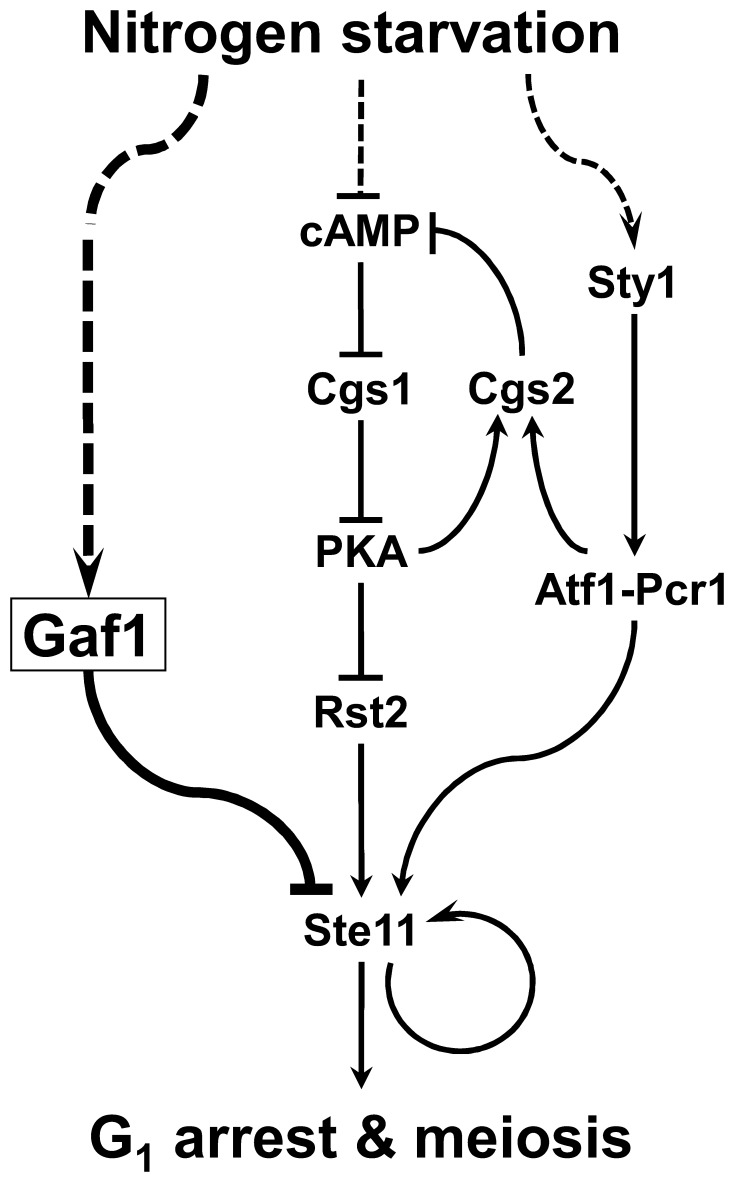
Schematic diagram showing the proposed function of Gaf1 in the nitrogen-signaling pathways in *S. pombe*. Nitrogen starvation causes induction of *gaf1^+^* expression, and Gaf1, in turn, represses the expression of *ste11^+^* via direct interaction with its promoter. It has been previously known that nitrogen starvation leads to the activation of Rst2 via the cAMP-dependent PKA pathway [14], [15] as well as Atf1-Pcr1 via the Sty1 MAPK pathway [3], [5], [6], [14]–[16], [47]–[49], consequently resulting in induction of *ste11^+^* expression. In addition, phosphodiesterase is most likely stimulated by PKA activity to create a feedback mechanism [50]. The pathway addressed in this study is shown in thick lines, and other paths previously determined are shown in thin lines. Activation and inhibition are indicated by arrows and crossing bars, respectively. Dotted lines indicate pathways remained to be fully determined.

Figure 7 shows a simplified view of the proposed role of Gaf1 in the nitrogen-signaling pathways governing the expression of *ste11^+^* and consequent sexual differentiation in *S. pombe* together with the cAMP-dependent PKA and stress-activated MAPK pathways determined previously. We found in the present study that nitrogen starvation causes induction of *gaf1^+^* expression, and Gaf1, in turn, represses the expression of *ste11^+^* via direct interaction with its promoter. Starvation of carbon or nitrogen source leads to decrease in the level of cAMP and subsequent drop of PKA activity, which consequently induces expression of *ste11^+^* through the activation of Rst2 that can bind to the promoter region of *ste11^+^* [4], [7], [46]. Nitrogen starvation also causes activation of the stress-activated MAPK pathway including Wis4, Win1, Wis1, and Sty1 [14], [15], leading to activation of Atf1 by phosphorylation [16], [47], [48]. Activated Atf1, in turn, forms a complex with another cAMP response element-binding protein Pcr1 to yield an Atf1-Pcr1 heterodimeric transcription factor, which is also required for expression of *ste11^+^* [5], [6], [49]. It has not yet been determined whether the Atf1-Pcr1 complex directly regulates the expression of *ste11^+^* or not. However, it has been reported that the Atf1-Pcr1 heterodimer directly activates the expression of *cgs2^+^* encoding a phosphodiesterase that has a major role in regulating the single cAMP-dependent PKA pathway [3]. In addition, phosphodiesterase is most likely stimulated by PKA activity to create a feedback mechanism [50]. Thus there exists a direct connection between the MAPK and PKA pathways mediated by the action of Atf1-Pcr1 complex. It remains to be determined whether the expression of *gaf1^+^* is subject to the control of either the cAMP-dependent PKA or the stress-activated MAPK pathway.

## Supporting Information

Table S1 **List of the genes up-regulated in unstarved (+N)** ***gaf1*** **Δ cells (Group −G).**
(PDF)

Table S2 **List of the genes up-regulated in nitrogen-starved wild-type (** ***gaf1*** **^+^) cells (Group −N).**
(PDF)

Table S3 **List of the genes up-regulated in nitrogen-starved (−N)** ***gaf1*** **Δ cells (Group −N/−G).**
(PDF)

Table S4 **List of the genes in Subgroup A.**
(PDF)

Table S5 **List of the genes in Subgroup B.**
(PDF)

Table S6 **List of the genes in Subgroup C.**
(PDF)

Table S7 **List of the genes in Subgroup D.**
(PDF)

Table S8 **List of the genes in Subgroup E.**
(PDF)

Table S9 **List of the genes in Subgroup F.**
(PDF)

Table S10 **List of the genes in Subgroup G.**
(PDF)
