# Supplementary material for: The Fission Yeast GATA Factor, Gaf1, Modulates Sexual Development via Direct Down-Regulation of ste11+ Expression in Response to Nitrogen Starvation
Source: PLoS One. 2012 Aug 10;7(8):e42409. doi: 10.1371/journal.pone.0042409 (PMC3416868; doi:10.1371/journal.pone.0042409)
Supplement: Table S1 — List of the genes up-regulated in unstarved (+N) gaf1 Δ cells (Group −G). (PDF) [file pone.0042409.s001.pdf]

Table S1. List of the genes up-regulated in unstarved (+N) *gaf1* Δ cells (Group -G)

*** 183 genes**

**Systematic**	**Gene name**	**Description (GeneDB)**	**Description (FunCat2)**	**Expression ratio (*gaf1* Δ, +N) / (WT, +N)**
SPAC1F8.02c		sequence orphan	sequence orphan	456.13
SPAC1F8.03c	*str3*	siderophore-iron transporter Str3	siderophore-iron transporter Str3 (PMID 12888492)	263.49
SPBC1683.09c	*frp1*	ferric-chelate reductase Frp1	ferric-chelate reductase Frp1	238.52
SPBC947.05c		ferric-chelate reductase (predicted)		53.02
SPAC4H3.12c		dubious	sequence orphan	23.87
SPAC1F7.08	*fio1*	iron transport multicopper oxidase Fio1	iron transport multicopper oxidase Fio1	16.51
SPAC1F7.07c	*fip1*	iron permease Fip1	iron permease Fip1	15.96
SPCC1620.04c	*mug55*	Cdc20/Fizzy family WD repeat protein	Cdc20/Fizzy family WD repeat protein	12.00
SPBCPT2R1.08c	*tlh2*	RecQ type DNA helicase Tlh1		10.69
SPAC4G8.15c			dubious	8.70
SPBPB21E7.04c		S-adenosylmethionine-dependent methyltransferase (predicted)	S-adenosylmethionine-dependent methyltransferase (predicted)	8.09
SPCC63.13		DNAJ domain protein	DNAJ domain protein	7.97
SPAC13F5.07c		term=sequence orphan; date=20080121	previusly annotated as dubious, may not be protein coding	7.11
SPAC23G3.03	*sib2*	ornithine N5 monooxygenase (predicted)	ornithine N5 monooxygenase (predicted)	6.79
SPBCPT2R1.06c		pseudogene		5.62
SPCC576.16c	*wtf22*	wtf element Wtf22		5.33
SPBC23G7.10c		NADH-dependent flavin oxidoreductase (predicted)		5.10
SPBPB21E7.05		sequence orphan	sequence orphan	4.94
SPBC839.06	*cta3*	P-type ATPase, calcium transporting Cta3	P-type ATPase, calcium transporting Cta3	4.70
SPAC8E11.06		sequence orphan	sequence orphan	4.46
SPAP27G11.08c	*meu32*	sequence orphan	sequence orphan	4.42
SPAC212.02		sequence orphan	sequence orphan	4.41
SPAC29E6.07		sequence orphan	sequence orphan	4.40
SPBC2F12.09c	*atf21*	transcription factor Atf21		4.39

SPAC13D6.01	*pof14*	F-box protein Pof14	F-box protein Pof14	4.28
SPBC106.02c	*srx1*	sulfiredoxin	sulphiredoxin (PMID 15824112)	4.27
SPAPB17E12.09		sequence orphan	sequence orphan	4.15
SPAC1F8.01	*ght3*	hexose transporter Ght3	hexose transporter Ght3 (PMID 10735857)	4.07
SPAC27D7.03c	*mei2*	RNA-binding protein involved in meiosis Mei2	RNA-binding protein involved in meiosis Mei2	4.07
SPCC757.02c		epimarase (predicted)	epimarase (predicted)	4.03
SPAC212.06c		pseudogene	pseudogene	3.91
SPCC191.10		sequence orphan	sequence orphan	3.80
SPAC4F10.17		conserved fungal protein	conserved fungal protein	3.74
SPAC1002.21			sequence orphan	3.67
SPBC359.05	*abc3*	ABC transporter Abc3	ABC transporter Abc3	3.60
SPAC1002.06c	*bqt2*	bouquet formation protein Bqt2	bouquet formation protein Bqt2	3.55
SPCC1393.10	*ctr4*	copper transporter complex subunit Ctr4	copper transporter complex subunit Ctr4 (PMID 11274192)	3.53
SPAC8C9.12c		iron ion transporter (predicted)	iron ion transporter (predicted)	3.48
SPBP4G3.03		PI31 proteasome regulator related		3.32
SPAC1A6.11		dubious	dubious	3.29
SPCC737.04		S. pombe specific UPF0300 family protein 6	S. pombe specific UPF0300 family protein 6	3.14
SPAC22F8.03c		sequence orphan	sequence orphan	3.12
SPCC1020.03		mitochondrial iron ion transporter	mitochondrial iron ion transporter	2.99
SPCC330.03c		NADPH-hemoprotein reductase	NADPH-hemoprotein reductase	2.97
SPBC1348.14c	*ght7*	hexose transporter Ght7	hexose transporter Ght7	2.81
SPBC887.16		dubious		2.80
SPBC1198.12	*mfr1*	fizzy-related protein Mfr1		2.79
SPAC23G3.02c	*sib1*	ferrichrome synthetase Sib1	ferrichrome synthetase Sib1	2.79
SPBC27B12.03c		lathosterol oxidase (predicted)		2.76
SPAC15E1.07c	*moa1*	meiotic cohesin complex associated protein Moa1	meiotic cohesin complex associated protein Moa1	2.75
SPAC869.05c		sulfate transporter (predicted)	sulfate transporter (predicted)	2.72
SPBC1711.14	*rec15*	meiotic recombination protein Rec15		2.69
SPBC32H8.06	*mug93*	TPR repeat protein, meiotically spliced		2.64
SPAC186.02c		hydroxyacid dehydrogenase (predicted)	hydroxyacid dehydrogenase (predicted)	2.59
SPAC9E9.17c		dubious	dubiuos	2.50
SPBC12C2.14c		dubious		2.45

SPBC16E9.01c	*php4*	CCAAT-binding factor complex subunit Php4		2.45
SPAC323.07c		MatE family transporter	MatE family transporter	2.43
SPBC4F6.09	*str1*	siderophore-iron transporter Str1		2.42
SPAC343.07	*mug28*	RNA-binding protein Mug28	RNA-binding protein Mug28	2.41
SPAC186.07c		hydroxyacid dehydrogenase (predicted)	hydroxyacid dehydrogenase (predicted)	2.38
SPBC8D2.19	*mde3*	serine/threonine protein kinase Mde3		2.37
SPAC1039.09	*isp5*	amino acid permease Isp5	amino acid permease Isp5	2.37
SPAC212.09c		pseudogene	pseudogene	2.35
SPAC17A5.11	*rec12*	endonuclease Rec12	endonuclease Rec12	2.35
SPCC622.21	*wtf12*	wtf element Wtf12	wtf element Wtf12	2.34
SPCC13B11.02c		sequence orphan	sequence orphan	2.30
SPAC9G1.09	*sid1*	PAK-related kinase Sid1	PAK-related kinase Sid1	2.27
SPAC32A11.01	*mug8*	conserved fungal protein	conserved fungal protein	2.27
SPAC869.03c		urea transporter (predicted)	urea transporter (predicted)	2.26
SPAC1F8.04c		hydrolase (predicted)	hydrolase (predicted)	2.21
SPAC212.07c		pseudogene	pseudogene	2.21
SPCC1442.11c		sequence orphan	sequence orphan	2.20
SPBC887.17		uracil permease (predicted)		2.20
SPCC548.02c	*wtf3*	wtf element Wtf3	wtf element Wtf3	2.19
SPAC1002.16c		nicotinic acid plasma membrane transporter (predicted)	nicotinic acid plasma membrane transporter (predicted)	2.18
SPAC977.13c		hydrolase		2.18
SPBC428.07	*meu6*	meiotic chromosome segregation protein Meu6	meiotic chromosome segregation protein Meu6	2.18
SPAC22F8.02c	*pvg5*	PvGal biosynthesis protein Pvg5	PvGal biosynthesis protein Pvg5	2.17
SPAC6G10.06		FAD-dependent amino acid oxidase (predicted)	amino acid oxidase (predicted)	2.16
SPAC5H10.07		sequence orphan	sequence orphan	2.16
SPAC4G9.07	*mug133*	S. pombe specific UPF0300 family protein 2	S. pombe specific UPF0300 family protein 2	2.16
SPBPJ4664.01	*dps1*	decaprenyl diphosphate synthase subunit Dps1		2.16
SPBC1271.06c	*mug96*	sequence orphan	sequence orphan	2.14
SPCC1223.02	*nmt1*	no message in thiamine Nmt1	no message in thiamine Nmt1	2.14
SPBC83.19c		sequence orphan		2.13
SPBC215.04	*git11*	heterotrimeric G protein gamma subunit Git11		2.11

SPBC1683.12		nicotinic acid plasma membrane transporter (predicted)	nicotinic acid plasma membrane transporter (predicted)	2.05
SPBC23G7.11		DNA-3-methyladenine glycosidase Mag2 (predicted)		2.01
SPAC1952.15c	*rec24*	meiotic recombination protein Rec24	meiotic recombination protein Rec24	2.00
SPAC6B12.16	*meu26*	conserved fungal protein	conserved fungal protein	1.99
SPBC216.02	*mcp5*	cortical anchoring factor for dynein Mcp5/Num1		1.99
SPCC338.02	*mug112*	sequence orphan	sequence orphan	1.97
SPBPB2B2.18		sequence orphan		1.97
SPAC750.01		pseudogene	pseudogene	1.96
SPCC417.12		carboxylesterase-lipase family (predicted)	carboxylesterase-lipase family (predicted)	1.95
SPAC31G5.09c	*spk1*	MAP kinase Spk1	MAP kinase Spk1	1.95
SPCC1183.10	*wtf10*	wtf element Wtf10	wtf element Wtf10	1.94
SPAC750.07c		S. pombe specific GPI anchored protein family 1	S. pombe specific GPI anchored protein family 1	1.93
SPAC11D3.17		zf-C2H2 type zinc finger protein	zinc finger protein	1.93
SPBC16G5.19		sequence orphan		1.92
SPBPB21E7.07	*aes1*	enhancer of RNA-mediated gene silencing	enhancer of RNA-mediated gene silencing (PMID 12034844)	1.92
SPAC343.20		sequence orphan	sequence orphan	1.91
SPCC1840.12		OPT oligopeptide transporter family	OPT oligopeptide transporter family	1.91
SPCC794.03		amino acid permease, unknown 13	amino acid permease, unknown 13	1.91
SPCC622.13c	*tti1*	Tel Two Interacting protein 1	conserved eukaryotic protein	1.91
SPAC24C9.15c	*spn5*	septin Spn5	septin Spn5	1.91
SPBC1271.07c		N-acetyltransferase (predicted)	N-acetyltransferase (predicted)	1.90
SPAC17A2.11		sequence orphan	sequence orphan	1.89
SPAC1751.04		sequence orphan	sequence orphan	1.86
SPAC227.13c	*isu1*	mitochondrial iron-sulfur cluster assembly scaffold protein Isu1	iron-sulfur cluster assembly scaffold protein Isu1	1.84
SPCC74.09	*mug24*	RNA-binding protein, rrm type	RNA-binding protein	1.82
SPACUNK4.15		2',3'-cyclic-nucleotide 3'-phosphodiesterase (predicted)	2',3'-cyclic-nucleotide 3'-phosphodiesterase (predicted)	1.81
SPAP27G11.16		sequence orphan	sequence orphan	1.80
SPBC23G7.13c		urea transporter (predicted)		1.80
SPAC14C4.08	*mug5*	meiotically upregulated gene Mug5	meiotically upregulated gene Mug5	1.80

SPAC25G10.04c	*rec10*	meiotic recombination protein Rec10	meiotic recombination protein Rec10 (PMID 7586030)	1.79
SPCC63.03		DNAJ domain protein, DNAJC11 family	DNAJ domain protein, DNAJC11 family	1.79
SPCC61.01c	*str2*	siderophore-iron transporter Str2		1.78
SPAPB18E9.03c			dubious	1.78
SPBC32C12.02	*ste11*	transcription factor Ste11		1.76
SPCC622.12c		NADP-specific glutamate dehydrogenase (predicted)	NADP-specific glutamate dehydrogenase (predicted)	1.76
SPAPB8E5.08		sequence orphan	sequence orphan	1.76
SPBC2G2.17c		beta-glucosidase Psu2 (predicted)		1.74
SPBC359.06	*mug14*	adducin	adducin	1.73
SPBC1683.11c		isocitrate lyase	isocitrate lyase	1.73
SPAC23D3.12		inorganic phosphate transporter (predicted)	inorganic phosphate transporter (predicted)	1.73
SPAC11E3.06	*map1*	MADS-box transcription factor Map1	MADS-box transcription factor Map1	1.72
SPBC19C2.04c	*ubp11*	ubiquitin C-terminal hydrolase Ubp11	ubiquitin C-terminal hydrolase Ubp11	1.72
SPBPB2B2.08		conserved fungal protein		1.71
SPBC24C6.06	*gpa1*	G-protein alpha subunit		1.70
SPAC22F8.04		triose phosphate transporter (predicted)	triose phosphate transporter (predicted)	1.69
SPAC1565.04c	*ste4*	adaptor protein Ste4	adaptor protein Ste4	1.69
SPBC29A10.12		HMG-box variant		1.69
SPAC15A10.10	*mde6*	Muskelin homolog	Muskelin homolog	1.68
SPAC1142.05	*ctr5*	copper transporter complex subunit Ctr5 (predicted)	copper transporter complex subunit Ctr5 (predicted)	1.67
SPBC12C2.03c		FAD binding protein (predicted)		1.66
SPAC26H5.09c		GFO/IDH/MocA family oxidoreductase	GFO/IDH/MocA family oxidoreductase	1.66
SPBC36.02c		spermidine family transporter (predicted)		1.65
SPBC2G2.09c	*crs1*	meiosis specific cyclin Crs1		1.65
SPAC222.15	*meu13*	Tat binding protein 1(TBP-1)-interacting protein (TBPIP) homolog (predicted)		1.64
SPBPJ4664.02		glycoprotein (predicted)		1.64
SPCPB1C11.01	*amt1*	ammonium transporter Amt1	ammonium transporter Amt1	1.63
SPCC965.12		dipeptidyl aminopeptidase (predicted)	dipeptidyl aminopeptidase (predicted)	1.63
SPAC3F10.10c	*map3*	pheromone M-factor receptor	pheromone M-factor receptor (PMID 8380233)	1.63
SPAC6C3.03c		sequence orphan	sequence orphan	1.62

SPBC19F8.06c	*meu22*	amino acid permease, unknown 11		1.62
SPBC660.06		conserved fungal protein	conserved fungal protein	1.61
SPBC19C7.05		cell wall organization protein (predicted)		1.61
SPBPB21E7.08		pseudogene	pseudogene	1.61
SPBC1921.04c		sequence orphan		1.59
SPCC645.12c		sequence orphan	sequence orphan	1.58
SPAC869.02c		nitric oxide dioxygenase (predicted)	nitric oxide dioxygenase (predicted)	1.57
SPAC890.02c	*alp7*	TACC homolog (predicted)	TACC homolog (predicted) (PMID 14742702)	1.57
SPAC25B8.02	*sds3*	Clr6 histone deacetylase complex subunit Sds3 (predicted)	histone deacetylase complex subunit Sds3 (predicted)	1.57
SPCC13B11.04c		glutathione-dependent formaldehyde dehydrogenase (predicted)		1.57
SPAC688.06c	*slx4*	structure-specific endonuclease subunit	structure-specific endonuclease subunit (PMID 14528010)	1.56
SPAC1399.01c		membrane transporter (predicted)	purine permease (predicted)	1.56
SPBC115.03		gfo/idh/mocA family oxidoreductase (predicted)	gfo/idh/mocA family oxidoreductase (predicted)mito	1.56
SPAC5H10.04		NADPH dehydrogenase (predicted)	NADPH dehydrogenase (predicted)	1.56
SPBC530.02		membrane transporter		1.55
SPCC162.04c	*wtf13*	wtf element Wtf13	wtf element Wtf13	1.55
SPBC1198.07c		mannan endo-1,6-alpha-mannosidase (predicted)	mannan endo-1,6-alpha-mannosidase (predicted)	1.55
SPAC1F8.05	*isp3*	sequence orphan	sequence orphan	1.55
SPAPB2B4.04c		P-type ATPase, calcium transporting Pmc1	P-type ATPase, calcium transporting Pmc1 (PMID 12707717)	1.54
SPAC27F1.10		sequence orphan	sequence orphan	1.54
SPCC584.16c		sequence orphan	sequence orphan	1.54
SPCC417.04		dubious	dubious	1.54
SPCC70.08c		methyltransferase (predicted)	methyltransferase (predicted)	1.53
SPAC3A12.18	*zwf1*	glucose-6-phosphate 1-dehydrogenase (predicted)		1.53
SPCC162.10	*ppk33*	serine/threonine protein kinase Ppk33 (predicted)	serine/threonine protein kinase Ppk33 (predicted)	1.53
SPAPB8E5.05	*mfm1*	M-factor precursor Mfm1	M-factor precursor Mfm1	1.53
SPBC119.14	*rti1*	Rad22 homolog Rti1		1.53

SPMIT.03		mitochondrial DNA binding endonuclease		1.52
SPCC126.14	*prp18*	U5 snRNP-associated protein Prp18	U5 snRNP-associated protein Prp18	1.52
SPBCPT2R1.02		sequence orphan		1.51
SPAC1039.08		serine acetyltransferase (predicted)	serine acetyltransferase (predicted)	1.51
SPCPB1C11.02		amino acid permease, unknown 16	amino acid permease, unknown 16	1.51
SPCC1322.03		TRP-like ion channel	TRP-like ion channel	1.50
SPCC1795.06	*map2*	P-factor	P-factor (PMID 8314086)	1.50
SPCC4G3.08	*psk1*	serine/threonine protein kinase Psk1	serine/threonine protein kinase Psk1	1.50
SPBC1711.01c	*matmi_1*	mating-type m-specific polypeptide mi		1.50
SPAC513.04		sequence orphan	sequence orphan	1.50
