# Supplementary material for: The Fission Yeast GATA Factor, Gaf1, Modulates Sexual Development via Direct Down-Regulation of ste11+ Expression in Response to Nitrogen Starvation
Source: PLoS One. 2012 Aug 10;7(8):e42409. doi: 10.1371/journal.pone.0042409 (PMC3416868; doi:10.1371/journal.pone.0042409)
Supplement: Table S2 — List of the genes up-regulated in nitrogen-starved wild-type ( gaf1 +) cells (Group −N). (PDF) [file pone.0042409.s002.pdf]

Table S2. List of the genes up-regulated in nitrogen-starved wild-type (*gaf1* $^{+}$) cells (Group -N)

*** 1,301 genes**

| Systematic | Gene name | Description (GeneDB) | Description (FunCat2) | Expression Ratio (WT, -N) / (WT, +N) |
|---|---|---|---|---|
| SPCC417.06c | *nmt1* | no message in thiamine Nmt1 | no message in thiamine Nmt1 | 1118.03 |
| SPAC589.07c | | urea transporter (predicted) | | 184.12 |
| SPCC1183.10 | | conserved fungal protein | conserved fungal protein | 165.16 |
| SPAC11H11.01 | | hydrolase (predicted) | hydrolase (predicted) | 107.88 |
| SPAC3C7.02c | *map2* | P-factor | P-factor (PMID 8314086) | 101.73 |
| SPCC126.02c | *ght3* | hexose transporter Ght3 | hexose transporter Ght3 (PMID 10735857) | 93.52 |
| SPCC417.10 | *mug14* | adducin | adducin | 82.76 |
| SPCC645.03c | *mel1* | alpha-galactosidase | alpha-galactosidase (PMID 15580593) | 76.27 |
| SPCC1529.01 | | sequence orphan | sequence orphan | 74.11 |
| SPCC622.06c | *ght1* | hexose transporter Ght1 | hexose transporter Ght1 (PMID 10735857) | 73.22 |
| SPAC32A11.03c | | HbrB family protein | HbrB family protein | 64.14 |
| SPAC1002.06c | | sequence orphan | sequence orphan | 59.80 |
| SPBP8B7.23 | | short chain dehydrogenase (predicted) | short chain dehydrogenase | 56.61 |
| SPAC637.03 | | pyruvate decarboxylase (predicted) | pyruvate decarboxylase (predicted) | 56.51 |
| SPBC31E1.04 | *mfm1* | M-factor precursor Mfm1 | M-factor precursor Mfm1 | 55.56 |
| SPBC19C7.10 | | conserved fungal protein | conserved fungal protein | 52.42 |
| SPAC26F1.04c | | sequence orphan | | 52.18 |
| SPBC337.07c | | succinate-semialdehyde dehydrogenase (predicted) | succinate-semialdehyde dehydrogenase (predicted) | 51.13 |
| SPAC24C9.15c | | cation binding protein (predicted) | cation binding protein (predicted) | 49.90 |
| SPBC20F10.03 | | epimarase (predicted) | epimarase (predicted) | 47.03 |
| SPBC146.02 | | phosphoketolase (predicted) | | 46.46 |
| SPAC3C7.05c | *ppk33* | serine/threonine protein kinase Ppk33 (predicted) | serine/threonine protein kinase Ppk33 (predicted) | 46.35 |
| SPBC17D11.01 | *mei2* | RNA-binding protein involved in meiosis Mei2 | RNA-binding protein involved in meiosis Mei2 | 43.74 |
| SPAC57A7.08 | *mug147* | sequence orphan | | 43.26 |
| SPBC16D10.06 | | sulfonate dioxygenase (predicted) | sulfonate dioxygenase (predicted) | 41.89 |
| SPCC1739.08c | | short chain dehydrogenase (predicted) | short chain dehydrogenase (predicted) | 41.51 |

| | | | | |
|---|---|---|---|---|
| SPBC14F5.10c | *ght4* | hexose transporter Ght4 | hexose transporter Ght4 (PMID 10735857) | 41.18 |
| SPAC11D3.10 | | rhomboid family protease | | 41.09 |
| SPBC17D1.07c | | PI31 proteasome regulator related | | 40.76 |
| SPAPB2B4.04c | | glucan 1,4-alpha-glucosidase (predicted) | glucan 1,4-alpha-glucosidase (predicted) | 40.20 |
| SPAC23C11.10 | *inv1* | beta-fructofuranosidase | beta-fructofuranosidase | 39.29 |
| SPBC1683.02 | *spk1* | MAP kinase Spk1 | MAP kinase Spk1 | 36.46 |
| SPAC31G5.21 | *isp5* | amino acid permease Isp5 | amino acid permease Isp5 | 33.67 |
| SPACUNK4.16c | | esterase/lipase (predicted) | esterase/lipase (predicted) | 33.62 |
| SPAC15A10.03c | *isp3* | sequence orphan | sequence orphan | 33.50 |
| SPCC1393.05 | | ThiJ domain protein | ThiJ domain protein | 33.31 |
| SPAC1F7.10 | | ThiJ domain protein | ThiJ domain protein | 33.27 |
| SPAC9G1.06c | | sequence orphan | sequence orphan | 32.13 |
| SPBC19C2.11c | *fbp1* | fructose-1,6-bisphosphatase Fbp1 | | 31.86 |
| SPBPB21E7.01c | | pseudo SPAC5H10.03 | pseudo SPAC5H10.03 | 31.49 |
| SPCC285.06c | *agl1* | alpha-glucosidase Agl1 | alpha-glucosidase Agl1 | 31.15 |
| SPBP4H10.10 | | short chain dehydrogenase (predicted) | short chain dehydrogenase (predicted) | 28.82 |
| SPCC364.05 | | sequence orphan | | 27.92 |
| SPBP19A11.02c | *eno102* | enolase (predicted) | enolase (predicted) | 26.65 |
| SPAC12B10.11 | | copper amine oxidase (predicted) | | 25.57 |
| SPBC1347.01c | | fumarate lyase superfamily | | 24.32 |
| SPCC1281.08 | | sequence orphan | sequence orphan | 24.12 |
| SPAC2G11.05c | *lsd90* | Lsd90 protein | | 22.97 |
| SPAC6F12.08c | | sequence orphan | | 22.95 |
| SPBC19G7.13 | *pol4* | DNA polymerase X family | DNA polymerase X family | 22.72 |
| SPBC8E4.03 | | uricase (predicted) | uricase (predicted) | 22.52 |
| SPBC365.08c | | MIP water channel (predicted) | MIP water channel | 22.08 |
| SPAC732.02c | | dubious | sequence orphan | 21.84 |
| SPAC9E9.15 | | mitochondrial iron-containing alcohol dehydrogenase family protein | glycerol dehydrogenase (Phlippen, Stevens, Wolf,Zimmermann manuscript in preparation) | 21.62 |
| SPAC24H6.11c | *shk2* | PAK-related kinase Shk2 | PAK-related kinase Shk2 (PMID 9660817) (PMID 9660818) | 20.72 |
| SPBC3E7.02c | *mug182* | YjeF family protein | YjeF family protein | 19.31 |
| SPCC777.05 | | DUF1761 family protein | DUF1761 family protein | 19.11 |
| SPBC337.12 | *mug114* | sequence orphan | sequence orphan | 18.64 |
| SPAC12B10.16c | | ThiJ domain protein | ThiJ domain protein | 18.51 |

| | | | | |
|---|---|---|---|---|
| SPAC15A10.16 | | conserved fungal protein | | 18.38 |
| SPBC16E9.11c | | UPF0103 family | UPF0103 family | 17.26 |
| SPCC965.07c | | glucose-6-phosphate 1-dehydrogenase (predicted) | glucose-6-phosphate 1-dehydrogenase (predicted) | 17.22 |
| SPCC1259.12c | | UDP-glucose 4-epimerase | | 16.93 |
| SPAC31A2.12 | | agmatinase (predicted) | agmatinase (predicted) | 16.87 |
| SPACUNK4.15 | | phosphoglycerate mutase family | phosphoglycerate mutase family | 16.35 |
| SPAC227.04 | | term=sequence orphan; date=20080121 | previusly annotated as dubious, may not be protein coding | 16.28 |
| SPAC22H10.07 | | yippee-like protein | yippee-like protein | 16.23 |
| SPCC162.06c | | membrane transporter (predicted) | purine permease (predicted) | 16.03 |
| SPCC330.11 | *meu31* | sequence orphan | sequence orphan | 15.76 |
| SPCC1223.13 | *gmh1* | alpha-1,2-galactosyltransferase Gmh1 (predicted) | alpha-1,2-galactosyltransferase Gmh1 | 15.73 |
| SPBC15D4.15 | *rsv1* | transcription factor Rsv1 (predicted) | | 15.71 |
| SPAC513.05 | | sequence orphan | sequence orphan | 15.60 |
| SPBC21C3.10c | | pseudogene | pseudogene | 15.21 |
| SPCC622.05 | | glucose-6-phosphate 1-dehydrogenase (predicted) | glucose-6-phosphate 1-dehydrogenase (predicted) | 15.14 |
| SPAC11H11.04 | *mug190* | C2 domain protein | C2 domain protein Tcb3 (predicted) | 15.06 |
| SPAC22H10.06c | *matmi_1* | mating-type m-specific polypeptide mi | | 15.00 |
| SPAC323.06c | *mug143* | sequence orphan | sequence orphan | 14.88 |
| SPCPB1C11.01 | | dubious | sequence orphan | 14.75 |
| SPBC21H7.06c | *urg1* | GTP cyclohydrolase II (predicted) | GTP cyclohydrolase (predicted) | 14.73 |
| SPAC19B12.07c | *rgs1* | regulator of G-protein signaling Rgs1 | regulator of G-protein signaling Rgs1 | 14.48 |
| SPAC57A7.09 | | pseudogene, similar to N-terminal of transmembrane channel | pseudogene, similar to N-terminal of transmembrane channel | 14.00 |
| SPCPB1C11.02 | | phosphoprotein phosphatase (predicted) | phosphoprotein phosphatase | 13.26 |
| SPBC32F12.09 | | membrane transporter | membrane transporter | 13.12 |
| SPBC685.05 | | FMN dependent dehydrogenase | FMN dependent dehydrogenase | 12.92 |
| SPAC17C9.12 | | protein kinase inhibitor (predicted) | protein kinase inhibitor (predicted) | 12.84 |
| SPAC3G6.05 | | DUF1770 family protein | DUF1770 family protein | 12.66 |
| SPAC24H6.01c | | human AMMECR1 homolog | human AMMECR1 homolog | 12.46 |
| SPBC36B7.09 | *dak2* | dihydroxyacetone kinase Dak2 | dihydroxyacetone kinase Dak2 (PMID 9804990) | 12.35 |
| SPAC23H4.14 | *zym1* | metallothionein | metallothionein (PMID 12050156) | 12.32 |
| SPBC1683.13c | | conserved fungal protein | | 12.30 |

| | | | | |
|---|---|---|---|---|
| SPAC2G11.03c | | allantoicase (predicted) | allantoicase (predicted) | 12.13 |
| SPBC15C4.06c | | galactose-1-phosphate uridylyltransferase (predicted) | | 12.08 |
| SPAC1F5.04c | | FAD-dependent amino acid oxidase (predicted) | amino acid oxidase (predicted) | 12.04 |
| SPAC630.04c | | methyltransferase (predicted) | methyltransferase (predicted) | 12.02 |
| SPAC1834.09 | | zinc finger protein zf-AN1 type | zinc finger protein zf-AN1 type | 11.92 |
| SPAPB24D3.10c | *rec24* | meiotic recombination protein Rec24 | meiotic recombination protein Rec24 | 11.82 |
| SPBP35G2.11c | *map1* | MADS-box transcription factor Map1 | MADS-box transcription factor Map1 | 11.77 |
| SPAC13G7.07 | *gpa1* | G-protein alpha subunit | | 11.54 |
| SPBC19F8.01c | *mug108* | sequence orphan | sequence orphan | 11.15 |
| SPBC3H7.08c | | sequence orphan | sequence orphan | 11.14 |
| SPBC1271.14 | *ste7* | meiotic suppressor protein Ste7 | meiotic suppressor protein Ste7 | 11.05 |
| SPBC31F10.08 | *mug158* | sulfatase modifying factor 1 related | | 11.04 |
| SPAC4G9.04c | *wtf22* | wtf element Wtf22 | | 11.02 |
| SPBC4C3.12 | *ste4* | adaptor protein Ste4 | adaptor protein Ste4 | 10.87 |
| SPBC3E7.05c | | conserved fungal protein | conserved fungal protein | 10.84 |
| SPAC11D3.16c | *rho5* | Rho family GTPase Rho5 | Rho family GTPase Rho5 | 10.67 |
| SPBC21.06c | *mug191* | alpha-1,6-mannanase (predicted) | alpha-1,6- mannanase (predicted) | 10.52 |
| SPBPB21E7.02c | *mug55* | Cdc20/Fizzy family WD repeat protein | Cdc20/Fizzy family WD repeat protein | 10.40 |
| SPCC594.06c | | potassium channel subunit (predicted) | potassium channel subunit (predicted) | 10.30 |
| SPAC1610.04 | *aif1* | apoptosis-inducing factor homolog Aif1 | apoptosis-inducing factor homolog Aif1 | 10.29 |
| SPCPJ732.02c | | pseudogene | | 10.23 |
| SPBC16C6.06 | | uridine ribohydrolase (predicted) | uridine ribohydrolase (predicted) | 10.11 |
| SPAPB1A10.10c | | glycerophosphodiester transporter | glycerophosphodiester transporter | 10.11 |
| SPAPB2B4.01c | | G-protein coupled receptor (predicted) | G-protein coupled receptor (predicted) | 10.00 |
| SPAC17A5.11 | | sequence orphan | sequence orphan | 9.85 |
| SPBC36.11 | *map4* | cell agglutination protein Map4 | | 9.74 |
| SPAC27F1.08 | *gti1* | gluconate transporter inducer Gti1 | gluconate transporter inducer Gti1 | 9.66 |
| SPBC1A4.04 | | sequence orphan | sequence orphan | 9.58 |
| SPAC17A2.11 | *mmf2* | homologous Pmf1p factor 1 | | 9.58 |
| SPAC1952.17c | *isp4* | OPT oligopeptide transporter family | | 9.49 |
| SPBC24C6.07 | | sequence orphan | sequence orphan | 9.40 |
| SPAC26A3.14c | | ureidoglycolate hydrolase (predicted) | ureidoglycolate hydrolase (predicted) | 9.40 |
| SPBC56F2.06 | | aldo/keto reductase, unknown biological role | | 9.24 |
| SPCC1020.05 | | hydrolase (inferred from context) | hydrolase (inferred from context) | 9.18 |
| SPCC1393.10 | | sequence orphan | sequence orphan | 8.92 |

| | | | | |
|---|---|---|---|---|
| SPAC167.08 | | OPT oligopeptide transporter family | OPT oligopeptide transporter family | 8.80 |
| SPBC21.07c | *isp7* | 2-OG-Fe(II) oxygenase superfamily protein | 2-OG-Fe(II) oxygenase superfamily protein | 8.46 |
| SPCC622.02 | | carbon-sulfur lyase (predicted) | carbon-sulfur lyase (predicted) | 8.45 |
| SPAC323.07c | | S-adenosylmethionine-dependent methyltransferase (predicted) | S-adenosylmethionine-dependent methyltransferase (predicted) | 8.44 |
| SPAC2F7.08c | *mfm3* | M-factor precursor Mfm3 | | 8.41 |
| SPBC1718.07c | *bsu1* | high-affinity import carrier for pyridoxine, pyridoxal, and pyridoxamine Bsu1 | high-affinity import carrier for pyridoxine,pyridoxal, and pyridoxamine Bsu1 | 8.40 |
| SPBC354.15 | | phosphoprotein phosphatase (predicted) | | 8.40 |
| SPAC15A10.05c | | conserved fungal protein | conserved fungal protein | 8.29 |
| SPCC777.13 | | NADH-dependent flavin oxidoreductase (predicted) | | 7.97 |
| SPAC17A2.01 | | sequence orphan | sequence orphan | 7.94 |
| SPCPB1C11.03 | | DNAJ domain protein | DNAJ domain protein | 7.81 |
| SPAC1687.12c | | riboflavin synthase | riboflavin synthase (PMID 14690539) | 7.78 |
| SPAC1635.01 | | tspO homolog | | 7.60 |
| SPCPB16A4.06c | | autophagy associated protein (predicted) | | 7.59 |
| SPAC750.04c | | membrane transporter (predicted) | membrane transporter (predicted) | 7.57 |
| SPCC757.13 | | amino acid permease, unknown 12 | | 7.57 |
| SPAC2E1P3.01 | | N-acetyltransferase (predicted) | N-acetyltransferase (predicted) | 7.55 |
| SPBC14C8.08c | *wtf3* | wtf element Wtf3 | wtf element Wtf3 | 7.50 |
| SPAC3A11.10c | | S. pombe specific UPF0321 family protein 3 | S. pombe specific UPF0321 family protein 3 | 7.42 |
| SPBC2F12.09c | *ste6* | guanyl-nucleotide exchange factor Ste6 | guanyl-nucleotide exchange factor Ste6 | 7.40 |
| SPAC144.17c | *tms1* | hexitol dehydrogenase (predicted) | hexitol dehydrogenase (predicted) | 7.40 |
| SPAC3A12.02 | *gna1* | glucosamine-phosphate N-acetyltransferase | glucosamine-phosphate N-acetyltransferase | 7.34 |
| SPAP27G11.08c | | conserved protein (fungal and plant) | conserved protein (fungal and plant) | 7.31 |
| SPAC13F5.03c | | nucleotide-sugar 4,6-dehydratase (predicted) | | 7.21 |
| SPBC725.06c | *aes1* | enhancer of RNA-mediated gene silencing | enhancer of RNA-mediated gene silencing (PMID 12034844) | 7.14 |
| SPCC4B3.17 | *ppr1* | L-azetidine-2-carboxylic acid acetyltransferase | L-azetidine-2-carboxylic acid acetyltransferase (PMID 12761200) | 7.09 |
| SPAC1A6.06c | *ish1* | LEA domain protein | | 6.93 |
| SPAC823.03 | | dipeptidyl aminopeptidase (predicted) | dipeptidyl aminopeptidase (predicted) | 6.91 |

| | | | | |
|---|---|---|---|---|
| SPBC29A3.03c | | short chain dehydrogenase (predicted) | short chain dehydrogenase (predicted) | 6.85 |
| SPBC21C3.11 | | pyrimidine 5'-nucleotidase (predicted) | pyrimidine 5'-nucleotidase (predicted) | 6.82 |
| SPBC1683.09c | | peptidase family C54 | peptidase family C54 | 6.81 |
| SPBC776.15c | | thioredoxin peroxidase | thioredoxin peroxidase | 6.80 |
| SPBC1711.01c | | hydrolase | | 6.80 |
| SPBC365.15 | | amidotransferase (predicted) | glutamine amidotransferase (predicted) | 6.78 |
| SPAC16E8.03 | | WD repeat protein | WD repeat protein | 6.72 |
| SPBC23E6.03c | | galactokinase Gal1 (predicted) | | 6.72 |
| SPAC57A10.08c | | copper amine oxidase (predicted) | copper amine oxidase (predicted) | 6.70 |
| SPAC1D4.11c | | FUN14 family protein | FUN14 family protein | 6.70 |
| SPAC29E6.07 | *wtf4* | wtf element Wtf4, pseudo | wtf element Wtf4, pseudo | 6.70 |
| SPBC691.05c | | | sequence orphan | 6.58 |
| SPAC23H3.15c | | sequence orphan | sequence orphan | 6.58 |
| SPBC106.02c | *map3* | pheromone M-factor receptor | pheromone M-factor receptor (PMID 8380233) | 6.53 |
| SPBC14F5.11c | | pyridoxal reductase (predicted) | pyridoxal reductase (predicted) | 6.42 |
| SPAC6F6.08c | *fus1* | formin Fus1 | formin Fus1 | 6.37 |
| SPAC1399.04c | *atg22* | autophagy associated protein Atg22 (predicted) | autophagy associated protein Atg22 (predicted) | 6.29 |
| SPBC16G5.16 | | DUF962 family protein | conserved protein (broad species distribution) | 6.27 |
| SPAC22H10.13 | | alpha,alpha-trehalose-phosphate synthase (predicted) | alpha,alpha-trehalose-phosphate synthase (predicted) | 6.21 |
| SPBC839.06 | | sequence orphan | sequence orphan | 6.17 |
| SPBC1652.01 | | serine protease (predicted) | serine protease (predicted) | 6.12 |
| SPBC1709.16c | | iron/ascorbate oxidoreductase family | | 6.07 |
| SPBC4B4.11 | | membrane transporter (predicted) | | 6.01 |
| SPAC19D5.07 | | conserved fungal protein | conserved fungal protein | 6.01 |
| SPCC576.16c | | DNA-3-methyladenine glycosidase Mag2 (predicted) | | 5.98 |
| SPCC1840.04 | | fungal cellulose binding domain protein | fungal cellulose binding domain protein | 5.98 |
| SPBC17F3.02 | | agmatinase 2 (predicted) | | 5.96 |
| SPAC688.13 | *rem1* | meiosis-specific cyclin Rem1 | | 5.94 |
| SPCC70.07c | | NAD-dependent glutamate dehydrogenase (predicted) | NAD-dependent glutamate dehydrogenase (predicted) | 5.90 |
| SPAC25H1.09 | *atg20* | sorting nexin Atg20 | sorting nexin Atg20 | 5.88 |

| | | | | |
|---|---|---|---|---|
| SPBC1683.06c | *ste11* | transcription factor Ste11 | | 5.87 |
| SPAC2E12.02 | *mug8* | conserved fungal protein | conserved fungal protein | 5.82 |
| SPBC1348.14c | *mug86* | acetate transporter (predicted) | acetate transporter (predicted) | 5.80 |
| SPCC550.09 | *cda1* | chitin deacetylase Cda1 | chitin deacetylase Cda1 | 5.72 |
| SPBC215.11c | | conserved eukaryotic protein | conserved eukaryotic protein | 5.71 |
| SPBC15D4.07c | | conserved fungal protein | conserved fungal protein | 5.63 |
| SPAC607.07c | | human aprataxin homolog | conserved eukaryotic protein | 5.62 |
| SPCC1393.02c | *wtf17* | wtf element Wtf17, pseudo | wtf element Wtf17, pseudo | 5.60 |
| SPCC965.05c | *isp6* | vacuolar serine protease Isp6 | vacuolar serine protease Isp6 | 5.54 |
| SPAC140.04 | *yak3* | aldose reductase YakC | | 5.54 |
| SPAC27E2.06c | | X-Pro dipeptidase (predicted) | | 5.49 |
| SPBC2D10.20 | *mug168* | sequence orphan | sequence orphan | 5.42 |
| SPAC31F12.01 | *hri1* | eIF2 alpha kinase Hri1 | eIF2 alpha kinase Hri1 | 5.40 |
| SPAC26F1.14c | | xylose and arabinose reductase (predicted) | xylose and arabinose reductase (predicted) | 5.39 |
| SPAC664.13 | *ran1* | serine/threonine protein kinase Ran1 | | 5.38 |
| SPBC20F10.10 | *cox3* | cytochrome c oxidase 3 | cytochrome c oxidase 3; similar to S. cerevisiae Q0275 | 5.38 |
| SPBC14C8.11c | *ucp10* | UBA/UAS domain protein Ucp10 | UBA/UAS domain protein Ucp10 | 5.37 |
| SPBC8D2.12c | | aminotransferase class-III, unknown specificty | aminotransferase class-III (predicted) | 5.35 |
| SPAC1006.09 | | glycerophosphoryl diester phosphodiesterase (predicted) | glycerophosphoryl diester phosphodiesterase (predicted) | 5.32 |
| SPCC126.04c | *str3* | siderophore-iron transporter Str3 | siderophore-iron transporter Str3 (PMID 12888492) | 5.31 |
| SPAC29B12.11c | | sequence orphan | sequence orphan | 5.30 |
| SPBC800.14c | | WD repeat protein Atg18 | WD repeat protein Atg18 | 5.30 |
| SPAC6B12.13 | | sequence orphan | sequence orphan | 5.28 |
| SPAC16E8.02 | | pseudogene | pseudogene | 5.24 |
| SPCC622.11 | *ayr1* | 1-acyldihydroxyacetone phosphate reductase (predicted) | 1-acyldihydroxyacetone phosphate reductase (predicted) | 5.22 |
| SPAC29B12.14c | | human MAWBP homolog | human MAWBP homolog | 5.22 |
| SPAP7G5.03 | | SPRY domain protein | SPRY domain protein | 5.21 |
| SPBC1198.06c | | phosphomethylpyrimidine kinase (predicted) | | 5.19 |
| SPAC8F11.05c | | NAD binding dehydrogenase family protein | NAD binding dehydrogenase family protein | 5.18 |

| | | | | |
|---|---|---|---|---|
| SPBC1773.08c | | phosphoprotein phosphatase (predicted) | phosphoprotein phosphatase (predicted) | 5.12 |
| SPAC25G10.07c | *ubp11* | ubiquitin C-terminal hydrolase Ubp11 | ubiquitin C-terminal hydrolase Ubp11 | 5.07 |
| SPBC32F12.01c | | AMP-binding dehydrogenase (predicted) | AMP-binding dehydrogenase (predicted) | 5.05 |
| SPAC1805.15c | | aldehyde dehydrogenase (predicted) | aldehyde dehydrogenase (predicted) | 5.05 |
| SPAC3H1.14 | | NADH/NADPH dependent indole-3-acetaldehyde reductase AKR3C2 | NADH/NADPH dependent indole-3-acetaldehyde reductase AKR3C2 | 5.03 |
| SPAC24B11.11c | | sequence orphan | sequence orphan | 5.00 |
| SPBP4H10.09 | | alpha-glucosidase | | 5.00 |
| SPBPB2B2.01 | | membrane transporter | membrane transporter | 4.93 |
| SPAC9G1.09 | *wtf18* | wtf element Wtf18 | wtf element Wtf18 | 4.91 |
| SPAC1B1.02c | | WD repeat protein, human WDR44 family | | 4.86 |
| SPBC3E7.01 | *rsv2* | transcription factor Rsv2 | | 4.86 |
| SPCC737.05 | *gst3* | glutathione S-transferase Gst3 | glutathione S-transferase (PMID 12151111) | 4.82 |
| SPAC31G5.12c | *srk1* | MAPK-activated protein kinase Srk1 | MAPK-activated protein kinase Srk1 | 4.81 |
| SPCC737.04 | | conserved fungal protein | conserved fungal protein | 4.78 |
| SPAC23C11.08 | | hydroxyacid dehydrogenase (predicted) | | 4.70 |
| SPAPB1A10.14 | *pyp2* | tyrosine phosphatase Pyp2 | tyrosine phosphatase Pyp2 | 4.69 |
| SPCC1393.12 | *mag1* | DNA-3-methyladenine glycosylase Mag1 | DNA-3-methyladenine glycosylase Mag1 | 4.67 |
| SPCC4G3.12c | | ubiquitin-protein ligase E3 (predicted) | | 4.64 |
| SPCC622.04 | | DUF423 protein | conserved protein (broad species distribution) | 4.61 |
| SPAC4G9.19 | | conserved fungal protein | conserved fungal protein | 4.60 |
| SPCC1620.09c | *bqt2* | bouquet formation protein Bqt2 | bouquet formation protein Bqt2 | 4.60 |
| SPBC24C6.09c | *exg3* | glucan 1,3-beta-glucosidase Exg3 | | 4.60 |
| SPAPB1A11.02 | | conserved fungal protein | conserved fungal protein | 4.56 |
| SPBP8B7.13 | | thiosulfate sulfurtransferase | | 4.55 |
| SPAC1039.07c | | hydantoin racemase family (predicted) | hydantoin racemase family (predicted) | 4.53 |
| SPBC1703.12 | *rad50* | DNA repair protein Rad50 | DNA repair protein Rad50 | 4.50 |
| SPBC660.09 | *rhp54* | Rad54 homolog Rhp54 | Rad54 homolog Rhp54 | 4.49 |
| SPCC736.13 | *meu32* | sequence orphan | sequence orphan | 4.48 |
| SPAC13G6.03 | | DUF1773 family protein 4 | DUF1773 family protein 4 | 4.48 |
| SPAC3H1.08c | | conserved protein (fungal and bacterial) | | 4.45 |
| SPCC1906.04 | | subtilisin related protein (predicted) | protease inhibitor (predicted) | 4.42 |

| | | | | |
|---|---|---|---|---|
| SPBC17D11.08 | | PI31 proteasome regulator related | | 4.40 |
| SPAC1399.01c | *ams1* | alpha-mannosidase (predicted) | alpha-mannosidase (predicted) | 4.40 |
| SPCC794.08 | *mug133* | S. pombe specific UPF0300 family protein 2 | S. pombe specific UPF0300 family protein 2 | 4.37 |
| SPCC74.03c | | conserved fungal protein | | 4.37 |
| SPAC11D3.11c | | short chain dehydrogenase (predicted) | short chain dehydrogenase | 4.36 |
| SPCC1183.11 | | conserved eukaryotic protein | | 4.32 |
| SPCC4G3.08 | *sst4* | sorting receptor for ubiquitinated membrane proteins, ESCRT 0 complex subunit Sst4 | sorting receptor for ubiquitinated membrane proteins (ISS) (PMID 12055639) | 4.29 |
| SPAC26F1.07 | *jmj2* | histone demethylase Jmj2 (predicted) | histone demethylase Jmj2 (predicted) | 4.29 |
| SPBC428.12c | *wtf21* | wtf element Wtf21 | wtf element Wtf21 | 4.27 |
| SPBC3E7.04c | | purine transporter (predicted) | purine transporter (predicted) | 4.26 |
| SPAC17C9.05c | *sut1* | alpha-glucoside transporter | alpha-glucoside transporter (PMID 11136464) | 4.23 |
| SPBC21C3.01c | | But2 family protein | S. pombe specific But2 family protein | 4.23 |
| SPBC23E6.01c | | zf-C2H2 type zinc finger protein | zinc finger protein | 4.23 |
| SPAC18G6.12c | | UPF0061 family protein | UPF0061 family protein | 4.20 |
| SPAC9.10 | | xylulose kinase (predicted) | xylulose kinase (predicted) | 4.20 |
| SPAC6F12.15c | *syj2* | inositol-polyphosphate 5-phosphatase (synaptojanin homolog 2) | | 4.17 |
| SPAC521.04c | *sst2* | human AMSH protein homolog | human amsh protein homolog | 4.16 |
| SPBC1683.01 | *git5* | heterotrimeric G protein beta subunit Git5 | | 4.15 |
| SPCC5E4.10c | *cgs1* | cAMP-dependent protein kinase regulatory subunit Cgs1 | cAMP-dependent protein kinase regulatory subunit Cgs1 | 4.15 |
| SPAC11D3.07c | *ctr6* | vacuolar copper transporter Ctr6 | | 4.15 |
| SPAC5D6.07c | | conserved protein | conserved protein | 4.14 |
| SPBC18H10.10c | | dubious | dubious | 4.13 |
| SPAC22G7.11c | | serine acetyltransferase (predicted) | serine acetyltransferase (predicted) | 4.12 |
| SPAC23A1.16c | | SNARE Vam7 (predicted) | SNARE Vam7 (predicted) | 4.11 |
| SPBC11B10.05c | *puc1* | cyclin Puc1 | | 4.11 |
| SPAC3A11.06 | *srw1* | CDK inhibitor Srw1 | CDK inhibitor Srw1 | 4.10 |
| SPCC1450.09c | *chr2* | chitin synthase regulatory factor (putative) Chr2 | chitin synthase regulatory factor (putative) Chr2 (PMID 15449309) | 4.10 |
| SPAC2C4.09 | | sulphate transporter (predicted) | sulphate transporter (predicted) | 4.09 |
| SPAC3G6.07 | | sequence orphan | | 4.08 |
| SPAC22E12.03c | | human RNF family homolog | human RNF family homolog | 4.07 |

| | | | | |
|---|---|---|---|---|
| SPAC5D6.08c | | LMBR1-like membrane protein | LMBR1-like membrane protein | 4.06 |
| SPAC186.03 | | aminotransferase class-III, unknown specificty | 4-aminobutyrate transaminase | 4.06 |
| SPAC513.04 | | conserved fungal protein | conserved fungal protein | 4.05 |
| SPCC338.02 | | pseudogene (predicted) | | 4.05 |
| SPAC23D3.14c | | sequence orphan | sequence orphan | 4.04 |
| SPBC8E4.04 | *mug185* | DNAJ domain protein Jjj family | DNAJ domain protein Jjj family | 4.04 |
| SPAC1556.01c | | sequence orphan | sequence orphan | 4.04 |
| SPAC4D7.01c | | MS ion channel protein 2 | MS ion channel protein 2 | 4.03 |
| SPBC14C8.09c | | acetyl-CoA hydrolase | acetyl-CoA hydrolase | 4.02 |
| SPAC2E12.03c | | conserved fungal protein | conserved fungal protein | 4.01 |
| SPAC29E6.01 | *gpi12* | pig-L (predicted) | pig-L | 4.01 |
| SPAC29B12.03 | | conjugation protein (predicted) | conjugation protein (predicted) | 4.00 |
| SPBC26H8.12 | | PSP1 family protein | | 3.99 |
| SPAC13C5.04 | | succinate-semialdehyde dehydrogenase (predicted) | succinate-semialdehyde dehydrogenase (predicted) | 3.99 |
| SPAC5H10.11 | *ppk35* | serine/threonine protein kinase Ppk35 | serine/threonine protein kinase Ppk35 | 3.93 |
| SPBC11B10.07c | | mitochondrial outer membrane protein (predicted) | | 3.92 |
| SPCC622.01c | | pseudogene | pseudogene | 3.91 |
| SPAC4G9.07 | | sequence orphan | sequence orphan | 3.90 |
| SPAC1F12.09 | | carbohydrate kinase (predicted) | carbohydrate kinase (predicted) | 3.90 |
| SPAC22H10.10 | *uga1* | 4-aminobutyrate aminotransferase (GABA transaminase) | 4-aminobutyrate aminotransferase (GABA transaminase) (predicted) | 3.89 |
| SPBC776.05 | *rev1* | deoxycytidyl transferase Rev1 (predicted) | deoxycytidyl transferase Rev1 (predicted) | 3.87 |
| SPCC1739.01 | *mug93* | TPR repeat protein, meiotically spliced | | 3.86 |
| SPAC821.04c | | CTLH domain | CTLH domain | 3.85 |
| SPCC24B10.07 | *apg3* | autophagy associated protein Apg3 (predicted) | autophagy associated protein Apg3 (predicted) | 3.85 |
| SPCC777.17c | | zinc finger protein, zf-C2H2 type/UBA domain protein | zinc finger protein | 3.84 |
| SPAC16.05c | | mitochondrial peptidase (predicted) | | 3.83 |
| SPCC191.01 | | NADH dehydrogenase | | 3.82 |
| SPAC4G9.13c | | F-box protein | F-box protein, unnamed | 3.82 |
| SPCC18B5.03 | *amt1* | ammonium transporter Amt1 | ammonium transporter Amt1 | 3.81 |
| SPAC23C4.16c | *atg13* | autophagy associated protein Atg13 (predicted) | autophagy associated protein Atg13 (predicted) | 3.80 |

| | | | | |
|---|---|---|---|---|
| SPBC32H8.06 | | human WW domain binding protein-2 ortholog | human WW domain binding protein-2 ortholog | 3.79 |
| SPAC15E1.02c | *mvp1* | sorting nexin Mvp1 | sorting nexin Mvp1 | 3.79 |
| SPCC70.05c | *meu10* | GPI anchored cell surface protein (predicted) | conserved fungal family | 3.78 |
| SPAC3G9.11c | | conserved fungal protein | conserved fungal protein | 3.78 |
| SPAP32A8.02 | | sequence orphan | sequence orphan | 3.78 |
| SPBPB21E7.04c | *mug63* | TLDc domain protein 1 | TLDc domain protein 1 | 3.77 |
| SPBC18E5.10 | | membrane transporter | | 3.77 |
| SPAC1093.04c | | gluconokinase | gluconokinase | 3.76 |
| SPCC74.09 | | membrane transporter | membrane transporter | 3.75 |
| SPAC644.06c | | P-type ATPase, calcium transporting Pmc1 | P-type ATPase, calcium transporting Pmc1 (PMID 12707717) | 3.75 |
| SPCC18.17c | | Usp (universal stress protein) family protein, implicated in meiotic chromosome segregation | Usp (universal stress protein) family protein,implicated in meiotic chromosome segregation | 3.74 |
| SPAC343.04c | *mfm2* | M-factor precursor Mfm2 | M-factor precursor Mfm2 | 3.72 |
| SPAC13G7.05 | | mitochondrial RNA-binding protein (predicted) | | 3.70 |
| SPCC965.12 | | DUF1772 family protein | DUF1772 family protein | 3.70 |
| SPAC30D11.01c | | sequence orphan | | 3.69 |
| SPAP8A3.12c | | amino acid permease, unknown 4 | amino acid permease, unknown 4 | 3.67 |
| SPBC1706.01 | *pka1* | cAMP-dependent protein kinase catalytic subunit Pka1 | cAMP-dependent protein kinase catalytic subunit Pka1 (PMID 8144551) | 3.67 |
| SPBC15D4.13c | *gos1* | SNARE Gos1 (predicted) | SNARE Gos1 | 3.66 |
| SPCC1450.06c | | bax inhibitor-like protein | bax inhibitor-like protein | 3.66 |
| SPAPB2B4.06 | | sequence orphan | sequence orphan | 3.65 |
| SPAC11D3.08c | | mitochondrial electron carrier (predicted) | mitochondrial electron carrier (predicted) | 3.64 |
| SPCC1494.09c | *mfr1* | fizzy-related protein Mfr1 | | 3.64 |
| SPAC977.10 | | alpha-1,2-mannosyltransferase (predicted) | alpha-1,2-mannosyltransferase (predicted) | 3.62 |
| SPAC1F8.04c | | phosphatidylinositol(3)-phosphate binding protein (predicted) | | 3.60 |
| SPBC9B6.03 | | vacuolar sorting protein Vps60 | vacuolar sorting protein Vps60 | 3.58 |
| SPCC63.13 | | UPF0031 family protein | conserved protein (broad species distribution) | 3.55 |
| SPCC1494.01 | *mmm1* | Mdm10/Mdm12/Mmm1 complex subunit Mmm1 (predicted) | | 3.54 |

| | | | | |
|---|---|---|---|---|
| SPCC970.01 | | ubiquitin-protein ligase E3 (predicted) | ubiquitin-protein ligase E3 (predicted) | 3.51 |
| SPCC794.02 | | conserved fungal protein | conserved fungal protein | 3.51 |
| SPBC1773.17c | *ubp8* | ubiquitin C-terminal hydrolase Ubp8 (predicted) | ubiquitin C-terminal hydrolase Ubp8 | 3.51 |
| SPBC3E7.06c | *idh2* | isocitrate dehydrogenase (NAD+) subunit 2 | isocitrate dehydrogenase (NAD+) subunit 2 | 3.50 |
| SPCC417.05c | | amino acid permease, unknown 16 | amino acid permease, unknown 16 | 3.50 |
| SPCC663.02 | | sequence orphan | | 3.50 |
| SPAC57A7.05 | | ubiquitin-protein ligase E3 (predicted) | | 3.49 |
| SPBC1A4.06c | | uncharacterised trans-sulfuration enzyme (predicted) | cystathionine gamma-synthase (predicted) | 3.48 |
| SPCC1840.06 | *mug112* | sequence orphan | sequence orphan | 3.48 |
| SPAC1565.04c | | alcohol dehydrogenase (predicted) | alcohol dehydrogenase (predicted) | 3.47 |
| SPBC12C2.08 | | sequence orphan | sequence orphan | 3.47 |
| SPBP8B7.08c | *gut2* | glycerol-3-phosphate dehydrogenase Gut2 | glycerol-3-phosphate dehydrogenase Gut2 | 3.47 |
| SPCC1450.08c | *ppk8* | serine/threonine protein kinase Ppk8 (predicted) | serine/threonine protein kinase Ppk8 (predicted) | 3.47 |
| SPAC750.07c | | adenine deaminase (predicted) | adenine deaminase (predicted) | 3.47 |
| SPAC23A1.17 | | vacuolar carboxypeptidase (predicted) | vacuolar carboxypeptidase (predicted) | 3.45 |
| SPBP23A10.09 | *cid13* | poly(A) polymerase Cid13 | poly(A) polymerase Cid13 (PMID 12062100) | 3.45 |
| SPBC25B2.08 | *mug99* | meiotically upregulated gene Mug99 | meiotically upregulated gene Mug99 | 3.44 |
| SPAC1F5.08c | *mug180* | esterase/lipase (predicted) | | 3.44 |
| SPCC622.21 | *wtf24* | wtf element Wtf24 | | 3.43 |
| SPAC13G6.05c | | proline specific permease (predicted) | proline specific permease (predicted) | 3.42 |
| SPBC16G5.19 | *atg8* | autophagy associated protein Atg8 (predicted) | | 3.42 |
| SPCC1739.03 | | mitochondrial ribosomal small subunit | | 3.41 |
| SPCC1919.06c | | Vid27 family protein | Vid27 family protein | 3.41 |
| SPAC17C9.16c | *pop1* | F-box/WD repeat protein Pop1 | | 3.41 |
| SPAC25B8.10 | | aldo/keto reductase involved in pentose catabolism (predicted) | | 3.39 |
| SPAC1D4.13 | *cta3* | P-type ATPase, calcium transporting Cta3 | P-type ATPase, calcium transporting Cta3 | 3.39 |
| SPAC3H1.06c | *ubc8* | ubiquitin conjugating enzyme Ubc8 | | 3.39 |
| SPAPB24D3.08c | *idh1* | isocitrate dehydrogenase (NAD+) subunit 1 Idh1 | isocitrate dehydrogenase (NAD+) subunit 1 Idh1 (PMID 10975257) | 3.38 |

| | | | | |
|---|---|---|---|---|
| SPBC725.05c | *wtf10* | wtf element Wtf10 | wtf element Wtf10 | 3.38 |
| SPBC1683.11c | *wtf14* | wtf element Wtf14 | wtf element Wtf14 | 3.38 |
| SPAC1952.11c | | zinc binding dehydrogenase | zinc binding dehydrogenase | 3.38 |
| SPAC4G8.10 | *ppk30* | Ark1/Prk1 family protein kinase Ppk30 | | 3.38 |
| SPBC1271.05c | | short chain dehydrogenase (predicted) | short chain dehydrogenase | 3.37 |
| SPBC1706.03 | | CIA30 family protein | CIA30 family protein | 3.37 |
| SPAC5D6.04 | *adl1* | DNA ligase (predicted) | DNA ligase (predicted) | 3.34 |
| SPAC19A8.12 | | sequence orphan | | 3.34 |
| SPBC29A10.09c | | vacuolar import and degradation protein Vid28 | vacuolar import and degradation protein Vid28 | 3.33 |
| SPAPB1A10.12c | | CAF1 family ribonuclease | | 3.33 |
| SPAC12B10.05 | *sst6* | ESCRT I complex subunit Vps23 | ESCRT I complex subunit Vps23 | 3.33 |
| SPBC365.12c | | steroid oxidoreductase superfamily protein | steroid oxidoreductase superfamily protein | 3.32 |
| SPAC2F3.05c | | malate dehydrogenase | malate dehydrogenase | 3.31 |
| SPAC1B9.02c | *sro1* | sequence orphan | sequence orphan | 3.30 |
| SPAC513.03 | | sequence orphan | | 3.29 |
| SPAC16A10.05c | *nbs1* | Mre11 complex subunit Nbs1 | | 3.29 |
| SPAC869.03c | | sequence orphan | sequence orphan | 3.29 |
| SPBC1921.07c | *wtf20* | wtf element Wtf20 | wtf element Wtf20 | 3.28 |
| SPBC337.15c | | guanine deaminase (predicted) | guanine deaminase (predicted) | 3.28 |
| SPBC13E7.11 | | WD repeat protein, human WDR68 family | | 3.27 |
| SPAC3C7.03c | *wtf25* | wtf element | wtf element | 3.26 |
| SPBC30B4.02c | *01-Oct* | mitochondrial intermediate peptidase Oct1 (predicted) | mitochondrial intermediate peptidase Oct1 (predicted) | 3.26 |
| SPAC6G9.16c | *ppk36* | serine/threonine protein kinase Ppk36 | serine/threonine protein kinase Ppk36 | 3.25 |
| SPBC1A4.01 | *cut2* | securin | | 3.25 |
| SPBC16A3.02c | | sequence orphan | sequence orphan | 3.25 |
| SPAC1F3.03 | | beclin family protein | | 3.25 |
| SPBC29B5.02c | | DNAJ domain protein DNAJB family | DNAJ domain protein DNAJB family | 3.23 |
| SPBC887.19 | *crk1* | cyclin-dependent kinase activating kinase Crk1 | | 3.23 |
| SPCC736.11 | | CDP-diacylglycerol-glycerol-3-phosphate3-phosphatidyltransferase | | 3.23 |
| SPAC9.08c | *Tf2-2* | retrotransposable element | | 3.23 |
| SPCC16C4.02c | | sequence orphan | sequence orphan | 3.22 |
| SPAC29A4.17c | | ubiquitin-protein ligase E3 (predicted) | ubiquitin-protein ligase E3 (predicted) | 3.21 |

| | | | | |
|---|---|---|---|---|
| SPBP8B7.28c | | conserved protein (fungal and plant) | conserved protein (fungal and plant) | 3.20 |
| SPBC36.12c | *etr1* | enoyl-[acyl-carrier protein] reductase | enoyl-[acyl-carrier protein] reductase | 3.19 |
| SPAC16E8.16 | *mok13* | alpha-1,3-glucan synthase Mok13 | | 3.19 |
| SPAPJ696.01c | | DUF726 family protein | DUF726 family protein | 3.19 |
| SPAC630.13c | | dihydrodiol dehydrogenase (predicted) | dihydrodiol dehydrogenase (predicted) | 3.19 |
| SPAC6G10.03c | | ubiquitin-protein ligase E3 (predicted) | | 3.18 |
| SPBC17D11.02c | | cystathionine beta-lyase (predicted) | cystathionine beta-lyase (predicted) | 3.17 |
| SPAC6G9.06c | | sequence orphan | sequence orphan | 3.17 |
| SPBC19C7.09c | | DUF580 family protein | DUF580 family protein | 3.16 |
| SPBC365.05c | *isa1* | iron-sulfur protein Isa1 | iron-sulfur protein Isa1 | 3.16 |
| SPAC15A10.01 | | protein phosphatase regulatory subunit Reg1 (predicted) | protein phosphatase regulatory subunit Reg1 (predicted) | 3.14 |
| SPAC3G9.02 | *pof9* | F-box protein Pof9 | | 3.14 |
| SPAC1783.06c | *gyp7* | GTPase activating protein Gyp7 (predicted) | GTPase activating protein Gyp7 (predicted) | 3.14 |
| SPBP4H10.12 | | S. pombe specific multicopy membrane protein family 1 | S. pombe specific multicopy membrane protein family 1 | 3.13 |
| SPAC19G12.09 | | zf-C3HC4 type zinc finger | | 3.12 |
| SPBC336.08 | | conserved fungal protein | conserved fungal protein | 3.12 |
| SPBC725.13c | *pxa1* | PXA domain protein Pxa1 | PXA domain protein | 3.11 |
| SPBC23G7.06c | | 5-formyltetrahydrofolate cyclo-ligase | | 3.11 |
| SPAC343.06c | | inosine-uridine preferring nucleoside hydrolase (predicted) | inosine-uridine preferring nucleoside hydrolase (predicted) | 3.10 |
| SPCC162.02c | | transthyretin (predicted) | transthyretin (predicted) | 3.10 |
| SPCC569.03 | | sequence orphan | | 3.10 |
| SPBPB7E8.02 | | phosphomethylpyrimidine kinase (predicted) | | 3.09 |
| SPBC725.03 | | proteasome regulatory particle, gankyrin (predicted) | gankyrin | 3.09 |
| SPAC1805.16c | *oma1* | metallopeptidase Oma1 (predicted) | metallopeptidase Oma1 (predicted) | 3.09 |
| SPAC4H3.04c | *png2* | ING family homolog Png2 | | 3.08 |
| SPCC1183.03c | | mitochondrial ATPase inhibitor (predicted) | mitochondrial ATPase inhibitor (predicted) | 3.07 |
| SPAC1002.05c | *eaf1* | RNA polymerase II transcription elongation factor SpEAF | RNA polymerase II transcription elongation factor SpEAF | 3.07 |
| SPAC1D4.03c | | L-asparaginase (predicted) | L-asparaginase (predicted) | 3.06 |
| SPAC17A2.15 | | ABC1 kinase family protein | | 3.06 |

| | | | | |
|---|---|---|---|---|
| SPAC4D7.02c | *sam50* | SAM complex subunit Sam50 (predicted) | SAM complex subunit Sam50 (predicted) | 3.03 |
| SPAC20G4.03c | *atf21* | transcription factor Atf21 | | 3.03 |
| SPAC17H9.19c | *hmt1* | ATP-binding cassette-type vacuolar membrane transporter Hmt1 | | 3.02 |
| SPBC32C12.02 | *nep2* | NEDD8 protease Nep2 | | 3.02 |
| SPCC970.11c | | conserved eukaryotic protein | | 3.02 |
| SPCC645.05c | *glo1* | glyoxalase I | | 3.02 |
| SPBC27B12.01c | *thi5* | transcription factor Thi5 | transcription factor Thi5 | 3.00 |
| SPCC622.07 | | phosphomethylpyrimidine kinase (predicted) | phosphomethylpyrimidine kinase (predicted) | 3.00 |
| SPAPB8E5.04c | | UbiE family methyltransferase (predicted) | UbiE family methyltransferase (predicted) | 3.00 |
| SPCC737.09c | | cysteine transporter (predicted) | cysteine transporter (predicted) | 3.00 |
| SPAC1687.22c | *mug135* | DUF1773 family protein 3 | DUF1773 family protein 3 | 2.99 |
| SPBP35G2.14 | *oca2* | serine/threonine protein kinase Oca2 (predicted) | serine/threonine protein kinase Oca2 (predicted) | 2.99 |
| SPCC63.03 | *fsv1* | SNARE Fsv1 | SNARE Fsv1 | 2.98 |
| SPBC3H7.13 | | sequence orphan | sequence orphan | 2.98 |
| SPAC16A10.06c | *rum1* | CDK inhibitor Rum1 | | 2.98 |
| SPBC12C2.10c | *mam1* | M-factor transporter Mam1 | | 2.97 |
| SPAC6B12.03c | | sequence orphan | | 2.97 |
| SPAC4G9.12 | | sequence orphan | | 2.97 |
| SPAC1B3.11c | *atm1* | ABC family iron transporter Atm1 | ABC family iron transporter Atm1 | 2.97 |
| SPAC23H3.03c | | GTPase activating protein (predicted) | GTPase activating protein (predicted) | 2.96 |
| SPBC20F10.07 | | Usp (universal stress protein) family protein | | 2.96 |
| SPAC25G10.01 | | CCR4-Not complex subunit Caf4/Mdv1 (predicted) | CCR4-Not complex subunit Caf4/Mdv1 (predicted) | 2.96 |
| SPBC405.05 | | aminotransferase class-III | 2,2-dialkylglycine decarboxylase (predicted) | 2.96 |
| SPAC1D4.12 | | NAD/NADH kinase (predicted) | NAD/NADH kinase | 2.95 |
| SPAC17C9.11c | *ell1* | RNA polymerase II transcription elongation factor SpELL | | 2.95 |
| SPCC569.06 | | MFS family transmembrane transporter Mfs1 | MFS family transmembrane transporter Mfs1 | 2.94 |
| SPCC285.09c | *pub2* | ubiquitin-protein ligase Pub2 | ubiquitin-protein ligase Pub2 | 2.94 |
| SPAC2G11.04 | *puf3* | RNA-binding protein Puf3 (predicted) | | 2.94 |

| | | | | |
|---|---|---|---|---|
| SPCC1235.04c | *moa1* | meiotic cohesin complex associated protein Moa1 | meiotic cohesin complex associated protein Moa1 | 2.94 |
| SPBC776.06c | | hypothetical protein | hypothetical protein | 2.94 |
| SPCC285.04 | *mkh1* | MEK kinase (MEKK) Mkh1 | MEK kinase (MEKK) Mkh1 (PMID 9199286) | 2.94 |
| SPBC19F8.07 | *gcn2* | eIF2 alpha kinase Gcn2 (predicted) | eIF2 alpha kinase Gcn2 (predicted) | 2.93 |
| SPAC4F8.01 | | conserved fungal protein | | 2.92 |
| SPBC1289.06c | | conserved protein (fungal and plant) | conserved protein (fungal and plant) | 2.92 |
| SPCC162.12 | *cuf2* | Cu metalloregulatory transcription factor Cuf2 | Cu metalloregulatory transcription factor Cuf2 | 2.92 |
| SPAC15E1.10 | | dubious | | 2.90 |
| SPCC1183.09c | | arrestin Aly1 related | arrestin Aly1 related | 2.90 |
| SPAC17A2.09c | | autophagy associated protein Atg24 (predicted) | autophagy associated protein Atg24 | 2.89 |
| SPAC3H5.11 | *wtf13* | wtf element Wtf13 | wtf element Wtf13 | 2.89 |
| SPAC6F12.03c | *phx1* | homeobox transcription factor Phx1 | homeobox transcription factor Phx1 | 2.89 |
| SPAP14E8.04 | | sequence orphan | sequence orphan | 2.88 |
| SPBC1348.12 | | dubious | dubious | 2.88 |
| SPBC1703.09 | | RNA-binding protein | RNA-binding protein | 2.88 |
| SPAC19A8.02 | *wtf5* | wtf element Wtf5 | wtf element Wtf5 | 2.87 |
| SPBC19F8.06c | *atg9* | autophagy associated protein Atg9 (predicted) | | 2.86 |
| SPAC16E8.09 | | sequence orphan | sequence orphan | 2.86 |
| SPAC29E6.09 | *arz1* | armadillo repeat containing, Zfs1 target number 1 | sequence orphan | 2.85 |
| SPBC947.15c | | sequence orphan | sequence orphan | 2.85 |
| SPBC16G5.07c | *msa1* | RNA-binding protein Msa1 | RNA-binding protein Msa1 | 2.84 |
| SPAC12G12.12 | *wtf16* | wtf element Wtf16 | wtf element Wtf16 | 2.84 |
| SPAC824.05 | *dma1* | mitotic spindle checkpoint protein Dma1 | mitotic spindle checkpoint protein Dma1 | 2.83 |
| SPBC1773.16c | | RNA-binding protein | RNA-binding protein | 2.83 |
| SPBC21C3.20c | | Lgl family protein | Lgl family protein | 2.83 |
| SPBC1289.15 | *pdh1* | DUF1751 family protein | DUF1751 family protein | 2.83 |
| SPAC513.02 | | DUF1212 family protein | DUF1212 family protein | 2.83 |
| SPBC1685.14c | | GFO/IDH/MocA family oxidoreductase | GFO/IDH/MocA family oxidoreductase | 2.83 |
| SPAC57A10.06 | *mug121* | sequence orphan | sequence orphan | 2.83 |
| SPAC4F8.10c | | zf-CCCH type zinc finger protein | zf-CCCH type zinc finger protein | 2.82 |

| | | | | |
|---|---|---|---|---|
| SPAC10F6.13c | | glutathione-dependent formaldehyde dehydrogenase (predicted) | glutathione-dependent formaldehyde dehydrogenase (predicted) | 2.81 |
| SPCC1840.09 | *nta1* | protein N-terminal amidase Nta1 (predicted) | | 2.81 |
| SPAC806.04c | | P-type ATPase | P-type ATPase (PMID 12707717) | 2.79 |
| SPAC24C9.14 | *rav1* | RAVE complex subunit Rav1 (predicted) | | 2.79 |
| SPAC23C4.07 | *spd1* | ribonucleotide reductase (RNR) inhibitor | ribonucleotide reductase (RNR) inhibitor | 2.78 |
| SPAC3C7.13c | *mug98* | sequence orphan | | 2.78 |
| SPCC645.02 | | amino acid permease, unknown 1 | amino acid permease, unknown 1 | 2.77 |
| SPAC19B12.08 | *xlf1* | xrcc4 like factor | xrcc4 like factor | 2.77 |
| SPBC1105.14 | *gpx1* | glutathione peroxidase Gpx1 | | 2.75 |
| SPAC23G3.03 | | R3H and G-patch domain, implicated in splicing | | 2.75 |
| SPAC24C9.08 | | dubious | dubiuos | 2.75 |
| SPAC5H10.02c | | S. pombe specific UPF0300 family protein 6 | S. pombe specific UPF0300 family protein 6 | 2.75 |
| SPAC22F3.05c | *git11* | heterotrimeric G protein gamma subunit Git11 | | 2.74 |
| SPAC21E11.04 | | GTPase activating protein | | 2.74 |
| SPBC1604.03c | | mitochondrial copper chaperone | mitochondrial copper ion transport protein | 2.73 |
| SPBC1703.08c | *clr1* | cryptic loci regulator Clr1 | | 2.73 |
| SPCC965.11c | *01-Sep* | fork head transcription factor Sep1 | | 2.72 |
| SPAC1527.02 | *coq7* | ubiquinone biosynthesis protein Coq7 | | 2.72 |
| SPBC31F10.10c | *alg13* | N-acetylglucosaminyldiphosphodolichol N-acetylglucosaminyltransferase Alg13 (predicted) | N-acetylglucosaminyldiphosphodolichol N-acetylglucosaminyltransferase Alg13 (predicted) | 2.72 |
| SPAC29A4.06c | | pseudo | | 2.72 |
| SPAC23A1.14c | *kin1* | microtubule affinity-regulating kinase Kin1 | | 2.72 |
| SPAC1002.17c | *frp1* | ferric-chelate reductase Frp1 | ferric-chelate reductase Frp1 | 2.72 |
| SPBC15D4.02 | | dubious | dubious | 2.72 |
| SPAC25B8.09 | | autophagy C terminal domain family protein | autophagy C terminal domain family protein | 2.71 |
| SPAC17G6.04c | | conserved fungal protein | | 2.71 |
| SPAC824.02 | | fasciclin domain protein (3) | fasciclin domain protein (3) | 2.71 |
| SPAC227.17c | | sequence orphan | sequence orphan | 2.70 |

| | | | | |
|---|---|---|---|---|
| SPAC222.13c | | aminopeptidase (predicted) | aspartyl aminopeptidase (predicted) | 2.70 |
| SPBC16E9.03c | | DUF544 family protein | DUF544 family protein | 2.69 |
| SPBC776.18c | *ppk19* | serine/threonine protein kinase Ppk19 | | 2.69 |
| SPCC794.01c | *nca2* | mitochondrial protein Nca2 (predicted) | mitochondrial protein Nca2 (predicted) | 2.69 |
| SPBPB2B2.18 | *tfg2* | transcription factor TFIIF complex beta subunit Tfg2 (predicted) | | 2.69 |
| SPAPB1A10.02 | *mug24* | RNA-binding protein, rrm type | RNA-binding protein | 2.69 |
| SPBC27B12.04c | *rec12* | endonuclease Rec12 | endonuclease Rec12 | 2.69 |
| SPBC8D2.01 | | mannan endo-1,6-alpha-mannosidase (predicted) | mannan endo-1,6-alpha-mannosidase (predicted) | 2.68 |
| SPCC188.08c | | Nudix family hydrolase | Nudix family hydrolase | 2.68 |
| SPAC3A11.14c | *ppk15* | serine/threonine protein kinase Ppk15 (predicted) | serine/threonine protein kinase Ppk15 (predicted) | 2.68 |
| SPCC1739.10 | | sequence orphan | sequence orphan | 2.67 |
| SPBC21D10.06c | | glutamate N-acetyltransferase (predicted) | glutamate N-acetyltransferase (predicted) | 2.67 |
| SPAC1002.12c | *rst2* | transcription factor Rst2 | transcription factor Rst2 | 2.67 |
| SPBC405.02c | | acyl-coA-sterol acyltransferase (predicted) | acyl-coA-sterol acyltransferase (predicted) | 2.67 |
| SPCC594.04c | | AAA family ATPase, unknown biological role | AAA family ATPase, unknown biological role | 2.66 |
| SPAC25B8.19c | | cardiolipin synthase/ hydrolase fusion protein (predicted) | cardiolipin synthase (predicted) | 2.66 |
| SPAC32A11.02c | | auxin family transmembrane transporter (predicted) | auxin family | 2.66 |
| SPBPB2B2.10c | *matmc_1* | mating-type m-specific polypeptide mc | | 2.65 |
| SPBC16H5.13 | *ppk34* | serine/threonine protein kinase Ppk34 | serine/threonine protein kinase Ppk34 | 2.65 |
| SPBC215.04 | *mug186* | sorting nexin Snx41 | | 2.65 |
| SPCC1020.03 | | TLDc domain protein 2 | | 2.64 |
| SPBC3B8.10c | | DUF1783 family protein | | 2.64 |
| SPAC458.04c | | beta-glucosidase Psu2 (predicted) | | 2.64 |
| SPAC1F7.12 | *mug161* | CwfJ family protein | CwfJ family protein | 2.64 |
| SPAC343.17c | *pkd2* | TRP-like ion channel | TRP-like ion channel (PMID 15537393) | 2.64 |
| SPAC186.06 | | phospholipase (predicted) | phospholipase | 2.63 |
| SPBC1604.18c | | calcium permease (predicted) | calcium permease (predicted) | 2.63 |
| SPBC354.13 | *hse1* | STAM like protein Hse1 | | 2.63 |
| SPBC582.10c | *wee1* | dual specificity protein kinase Wee1 | dual specificity protein kinase Wee1 | 2.63 |
| SPAC13C5.06c | | carboxyl methyl esterase | | 2.63 |

| | | | | |
|---|---|---|---|---|
| SPCC364.04c | | L-asparaginase (predicted) | L-asparaginase | 2.62 |
| SPBC27.03 | | membrane transporter | membrane transporter | 2.61 |
| SPCC1442.01 | | GPI inositol deacylase | GPI inositol deacylase | 2.61 |
| SPBC32H8.07 | | purine nucleoside phosphorylase (predicted) | purine nucleoside phosphorylase (predicted) | 2.61 |
| SPBC365.20c | | conserved fungal protein | conserved fungal protein | 2.61 |
| SPAC25A8.02 | *cds1* | replication checkpoint kinase Cds1 | replication checkpoint kinase Cds1 | 2.60 |
| SPAC343.03 | *psp3* | serine protease Psp3 (predicted) | serine protease Psp3 (predicted) | 2.60 |
| SPAC9.13c | | sequence orphan | sequence orphan | 2.60 |
| SPAC1B3.15c | | dubious | dubious | 2.60 |
| SPAC19E9.03 | | sequence orphan | sequence orphan | 2.59 |
| SPAC869.10c | | C2 domain protein | C2 domain protein | 2.59 |
| SPCC1919.01 | | sequence orphan | | 2.59 |
| SPAC4F10.16c | | SAGA complex subunit Sgf73 (predicted) | SAGA complex subunit Sgf73 (predicted) | 2.59 |
| SPBC29A10.02 | *rhp55* | RecA family ATPase Rhp55 | RecA family ATPase Rhp55 | 2.59 |
| SPAPB1A10.07c | *ght7* | hexose transporter Ght7 | hexose transporter Ght7 | 2.58 |
| SPAC2G11.13 | | amino acid permease, unknown 13 | amino acid permease, unknown 13 | 2.58 |
| SPBC16C6.04 | *wtf12* | wtf element Wtf12 | wtf element Wtf12 | 2.58 |
| SPBC3H7.03c | | SPX/EXS domain protein | SPX/EXS domain protein | 2.58 |
| SPBC30D10.03c | | ribonuclease II (RNB) family | ribonuclease II (RNB) family | 2.57 |
| SPAC19A8.03 | | nucleoside diphosphate-sugar hydrolase (predicted) | | 2.57 |
| SPAC15E1.07c | *aut12* | autophagy associated protein Aut12 (predicted) | autophagy associated protein Aut12 | 2.57 |
| SPBC8E4.05c | | transcription factor (predicted) | | 2.57 |
| SPAC11E3.14 | *klf1* | conserved fungal protein | conserved fungal protein | 2.56 |
| SPBC32H8.02c | | aromatic ring-opening dioxygenase (predicted) | | 2.56 |
| SPCC548.02c | | haloacid dehalogenase-like hydrolase | | 2.56 |
| SPAC14C4.01c | | conserved eukaryotic protein | | 2.55 |
| SPCC1739.04c | | methionine-tRNA ligase, mitochondrial | methionine-tRNA ligase | 2.55 |
| SPCC285.07c | | hydroxyacid dehydrogenase (predicted) | hydroxyacid dehydrogenase (predicted) | 2.55 |
| SPCC1223.02 | *ptc1* | protein phosphatase 2C Ptc1 | protein phosphatase 2C Ptc1 | 2.55 |
| SPBC3B9.10 | *zip1* | transcription factor Zip1 | transcription factor Zip1 | 2.55 |
| SPBC337.02c | *pcm2* | protein-L-isoaspartate O-methyltransferase (predicted) | protein-L-isoaspartate O-methyltransferase (predicted) | 2.55 |
| SPCC4B3.16 | *pep1* | sorting receptor for CPY | | 2.55 |

| | | | | |
|---|---|---|---|---|
| SPAC6F12.02 | | kinesin-like protein | | 2.54 |
| SPCC338.18 | *atf1* | transcription factor Atf1 | | 2.54 |
| SPBC1773.06c | *pms1* | MutL family mismatch-repair protein Pms1 | MutL family mismatch-repair protein Pms1 | 2.53 |
| SPAC922.03 | | human down-regulated in multiple cancers-1 homolog 2 | | 2.53 |
| SPAC25B8.08 | | ribonuclease II (RNB) family | | 2.53 |
| SPCC1795.01c | | pseudogene | | 2.53 |
| SPCC70.08c | | conserved fungal protein | conserved fungal protein | 2.53 |
| SPAC29B12.13 | | C2 domain protein | C2 domain protein | 2.52 |
| SPAC589.08c | | phospholipase (predicted) | phospholipase (predicted) | 2.52 |
| SPBC725.10 | *wtf9* | wtf element, Wtf2, pseudo | wtf element, Wtf2, pseudo | 2.52 |
| SPAC2C4.17c | *pof14* | F-box protein Pof14 | F-box protein Pof14 | 2.52 |
| SPBPB21E7.05 | *wtf1* | wtf element Wtf1, pseudo | | 2.51 |
| SPAC22G7.08 | *sfp1* | transcription factor Sfp1 (predicted) | transcription factor Sfp1 (predicted) | 2.51 |
| SPAPJ760.02c | | RNA-binding protein, rrm type | | 2.51 |
| SPBC215.03c | | pyruvate dehydrogenase protein x component | pyruvate dehydrogenase protein x component | 2.51 |
| SPAC16A10.08c | | mitochondrial hydrolase (predicted) | mitochondrial hydrolase | 2.51 |
| SPCC663.03 | | dubious | dubious | 2.50 |
| SPBC20F10.05 | *sib2* | ornithine N5 monooxygenase (predicted) | ornithine N5 monooxygenase (predicted) | 2.50 |
| SPAC17G6.12 | | zinc finger protein | | 2.50 |
| SPBC17A3.03c | | BRO1 domain protein | BRO1 domain protein | 2.50 |
| SPCP20C8.01c | *byr2* | MAP kinase kinase kinase Byr2 | | 2.50 |
| SPCPB16A4.05c | *mug179* | WD repeat protein Mug179 | WD repeat protein Mug179 | 2.50 |
| SPAC1687.09 | *kms1* | meiotic spindle pole body protein Kms1 | meiotic spindle pole body protein Kms1 | 2.50 |
| SPBC651.02 | | transcription factor (predicted) | transcription factor (predicted) | 2.49 |
| SPACUNK4.08 | | transcription factor (predicted) | transcription factor (predicted) | 2.49 |
| SPAC4G9.11c | *alp41* | ADP-ribosylation factor Alp41 | ADP-ribosylation factor Alp41 | 2.49 |
| SPAC2F3.16 | *sib1* | ferrichrome synthetase Sib1 | ferrichrome synthetase Sib1 | 2.49 |
| SPCC576.04 | *ubx4* | UBX domain protein Ubx4 (predicted) | | 2.48 |
| SPAPB15E9.02c | | conserved fungal protein | conserved fungal protein | 2.48 |
| SPBC887.15c | *uve1* | endonuclease Uve1 | | 2.48 |
| SPCC548.03c | | HVA22/TB2/DP1 family protein | | 2.48 |
| SPAC4D7.11 | *wtf2* | wtf element Wtf2, pseudo | | 2.48 |

| | | | | |
|---|---|---|---|---|
| SPAC20H4.11c | | abhydrolase family protein, unknown biological role | abhydrolase family protein, unknown biological role | 2.48 |
| SPCC1672.03c | | ubiquitin family, human C1ORF55 related | ubiquitin family, human C1ORF55 related | 2.48 |
| SPBC32F12.15 | | ubiquitin-like conjugating enzyme | | 2.48 |
| SPAC22F3.12c | *cox10* | protoheme IX farnesyltransferase | | 2.47 |
| SPBC23E6.09 | | hydroxyacylglutathione hydrolase (predicted) | hydroxyacylglutathione hydrolase (predicted) | 2.47 |
| SPAC1F8.03c | | MatE family transporter | MatE family transporter | 2.47 |
| SPAC212.06c | | glutamate-1-semialdehyde 2,1-aminomutaseaminotransferase (predicted) | glutamate-1-semialdehyde 2,1-aminomutaseaminotransferase (predicted) | 2.47 |
| SPAC2F7.06c | *rft1* | Man5GlcNac2-PP-Dol translocation protein Rft1 | | 2.46 |
| SPAC11D3.17 | *psk1* | serine/threonine protein kinase Psk1 | serine/threonine protein kinase Psk1 | 2.46 |
| SPAC1F8.05 | | tripeptidylpeptidase (predicted) | tripeptidylpeptidase (predicted) | 2.45 |
| SPAC13A11.04c | | dubious | sequence orphan | 2.45 |
| SPBC1709.14 | *ctt1* | catalase | catalase | 2.44 |
| SPBP8B7.26 | *med15* | mediator complex subunit Med15 (predicted) | mediator complex subunit Med15 (predicted) | 2.44 |
| SPAC1399.02 | | conserved fungal family | conserved fungal family | 2.44 |
| SPCC63.08c | | sterol binding ankyrin repeat protein | | 2.44 |
| SPCC1672.06c | | D-amino acid oxidase (predicted) | D-amino acid oxidase (predicted) | 2.43 |
| SPAC19G12.02c | | conserved fungal protein | | 2.43 |
| SPAC20H4.09 | *spc24* | spindle pole body protein Spc24 | | 2.43 |
| SPCC1672.04c | | sequence orphan | | 2.42 |
| SPAC823.09c | *dam1* | DASH complex subunit Dam1 | DASH complex subunit Dam1 | 2.42 |
| SPBC13E7.02 | *php2* | CCAAT-binding factor complex subunit Php2 | | 2.42 |
| SPCC320.09 | | NST UDP-galactose transporter (predicted) | NST UDP-galactose transporter | 2.42 |
| SPBC543.04 | | ubiquitin-protein ligase E3 (predicted) | ubiquitin-protein ligase E3 (predicted) | 2.42 |
| SPBC725.11c | *vps26* | retromer complex subunit Vps26 | retromer complex subunit Vps26 | 2.41 |
| SPAC4G8.11c | | cytochrome c heme lyase | | 2.41 |
| SPAC6B12.07c | | trans-aconitate 3-methyltransferase (predicted) | trans-aconitate 3-methyltransferase (predicted) | 2.41 |
| SPAC4H3.03c | *mug35* | sequence orphan | sequence orphan | 2.41 |
| SPBC1271.07c | *vps35* | retromer complex subunit Vps35 | retromer complex subunit Vps35 | 2.41 |

| | | | | |
|---|---|---|---|---|
| SPBC16A3.17c | | sulfate transporter (predicted) | sulfate transporter (predicted) | 2.41 |
| SPAC458.03 | *ght5* | hexose transporter Ght5 | hexose transporter Ght5 (PMID 10735857) | 2.40 |
| SPCC4G3.03 | | mitochondrial matrix protein import protein | | 2.40 |
| SPBC19F5.01c | | ubiquitin-protein ligase E3 (predicted) | ubiquitin-protein ligase E3 (predicted) | 2.40 |
| SPBC1271.08c | *pep12* | SNARE Pep12 | SNARE Pep12 | 2.40 |
| SPBC25H2.03 | | UDP-GlcNAc transferase associated protein Alg14 | UDP-GlcNAc transferase associated protein Alg14 | 2.40 |
| SPAPB24D3.04c | | | | 2.39 |
| SPAC22H12.03 | *cbf12* | CBF1/Su(H)/LAG-1 family transcription factor Cbf12 | transcription factor (predicted) | 2.39 |
| SPAC26H5.04 | | sequence orphan | sequence orphan | 2.39 |
| SPBC15D4.01c | *stn1* | telomere cap complex subunit Stn1 | | 2.39 |
| SPAC1B3.04c | *ubp9* | ubiquitin C-terminal hydrolase Ubp9 | ubiquitin C-terminal hydrolase Ubp9 | 2.38 |
| SPAC1296.04 | *alp21* | tubulin specific chaperone cofactor E | tubulin specific chaperone cofactor E | 2.38 |
| SPAC328.03 | | fructose-2,6-bisphosphate 2-phosphatase activity (predicted) | 6-phosphofructo-2-kinase (predicted) | 2.37 |
| SPAC8C9.16c | | RCC domain protein Ats1 (predicted) | RCC domain protein Ats1 (predicted) | 2.36 |
| SPCC61.03 | | 1-aminocyclopropane-1-carboxylate deaminase (predicted) | 1-aminocyclopropane-1-carboxylate deaminase (predicted) | 2.36 |
| SPAC23H3.12c | | peptide methionine sulfoxide reductase (predicted) | peptide methionine sulfoxide reductase (predicted) | 2.36 |
| SPAC10F6.11c | *ubp21* | ubiquitin C-terminal hydrolase Ubp21 | | 2.36 |
| SPAC14C4.10c | *wtf11* | wtf element Wtf11 | wtf element Wtf11 | 2.36 |
| SPCC1393.07c | | autophagy associated kinase activator (predicted) | kinase activator (predicted) | 2.36 |
| SPCC16A11.08 | *ssn6* | transcriptional corepressor Ssn6 | | 2.35 |
| SPMIT.08 | | heavy metal ion homeostasis protein (predicted) | heavy metal ion homeostasis protein (predicted) | 2.35 |
| SPBC1778.02 | | S. pombe specific GPI anchored protein family 1 | S. pombe specific GPI anchored protein family 1 | 2.35 |
| SPBC19F8.03c | | ubiquitin-protein ligase E3 (predicted) | | 2.35 |
| SPBC11G11.01 | | transcription related zf-ZZ type zinc finger protein | | 2.34 |
| SPAC1039.10 | | sequence orphan | sequence orphan | 2.34 |
| SPCC1827.07c | | alanine racemase (predicted) | alanine racemase (predicted) | 2.34 |
| SPBPB2B2.02 | *hsr1* | transcription factor Hsr1 | transcription factor Hsr1 | 2.34 |

| | | | | |
|---|---|---|---|---|
| SPAC24C9.07c | | mitochondrial NAD+ transporter (predicted) | mitochondrial NAD+ transporter | 2.33 |
| SPBC16C6.02c | | Ran GTPase binding protein (predicted) | Ran GTPase binding protein (predicted) | 2.33 |
| SPBC146.12 | | short chain dehydrogenase (predicted) | short chain dehydrogenase (predicted) | 2.33 |
| SPBC25B2.10 | | mitochondrial rhomboid protease | | 2.33 |
| SPAC922.07c | | haloacid dehalogenase-like hydrolase | haloacid dehalogenase-like hydrolase | 2.32 |
| SPAC977.04 | | human family 32A homolog | human family 32A homolog | 2.32 |
| SPBC725.02 | | nicotinamide riboside kinase (predicted) | nicotinamide riboside kinase | 2.32 |
| SPAC25B8.13c | *sec74* | guanyl-nucleotide exchange factor Sec74 | | 2.32 |
| SPAC1039.08 | | ATP-dependent DNA helicase Rhp16b (predicted) | ATP-dependent DNA helicase Rhp16b (predicted) | 2.32 |
| SPCC584.02 | *cyr1* | adenylate cyclase | | 2.32 |
| SPBC3H7.06c | | inositol metabolism protein Opi10 (predicted) | | 2.31 |
| SPAC3F10.10c | *pik3* | phosphatidylinositol 3-kinase Pik3 | phosphatidylinositol 3-kinase Pik3 | 2.31 |
| SPAC1565.03 | | phosphoprotein phosphatase (predicted) | | 2.31 |
| SPAPB18E9.02c | | xylose and arabinose reductase (predicted) | xylose and arabinose reductase (predicted) | 2.31 |
| SPAC16C9.01c | *wtf15* | wtf element Wtf15, pseudo | wtf element Wtf15, pseudo | 2.31 |
| SPBC691.01 | *mug130* | sequence orphan | sequence orphan | 2.31 |
| SPAC1039.11c | *cul1* | cullin 1 | cullin 1 | 2.31 |
| SPBC21C3.12c | | conserved eukaryotic protein | conserved eukaryotic protein | 2.30 |
| SPBC4B4.10c | *byr1* | MAP kinase kinase Byr1 | MAP kinase kinase Byr1 | 2.30 |
| SPCC663.17 | | dubious | dubious | 2.30 |
| SPCC31H12.06 | | conserved fungal protein | conserved fungal protein | 2.30 |
| SPBC660.08 | *vps3* | GTPase regulator Vps3 (predicted) | GTPase regulator Vps3 (predicted) | 2.30 |
| SPBPB21E7.07 | | sequence orphan | sequence orphan | 2.30 |
| SPAC1782.12c | | nicotinamidase (predicted) | | 2.29 |
| SPAC6G10.06 | | ubiquitin-protein ligase E3 (predicted) | | 2.29 |
| SPBPB2B2.12c | | prohibitin (predicted) | | 2.29 |
| SPBC887.16 | *pof13* | F-box protein Pof13 | F-box protein Pof13 | 2.28 |
| SPBC25B2.02c | *cut9* | anaphase-promoting complex subunit Cut9 | anaphase-promoting complex subunit Cut9 | 2.28 |
| SPAC3A11.09 | *atg2* | autophagy associated protein Mug36 | autophagy associated protein Mug36 | 2.28 |
| SPBC12C2.04 | | WD repeat protein, human IQWD1 family | | 2.28 |

| | | | | |
|---|---|---|---|---|
| SPBC713.07c | | transcription factor TFIIB | transcription factor TFIIB (PMID 12359329) | 2.28 |
| SPBC30D10.14 | | 2-oxoglutarate dehydrogenase (lipoamide) (e1 component of oxoglutarate dehydrogenase complex) (predicted) | | 2.28 |
| SPAC1F8.02c | | vacuolar polyphosphatase (predicted) | | 2.28 |
| SPCC550.10 | *mpr1* | response regulator phosphotransferase | | 2.27 |
| SPAC13D6.01 | *pmc3* | mediator complex subunit Pmc3 (pers. comm. Tomas Linder, from PMID 10625684) | mediator complex subunit Pmc3 (pers. comm. Tomas Linder, from PMID 10625684) | 2.27 |
| SPCC1442.05c | | sequence orphan | sequence orphan | 2.27 |
| SPAC521.03 | *mug2* | DUF1773 family protein 1 | DUF1773 family protein 1 | 2.27 |
| SPAC25B8.04c | *nup131* | nucleoporin Nup131 | | 2.27 |
| SPCC1442.04c | | membrane transporter | | 2.27 |
| SPBC336.12c | *rhp7* | Rad7 homolog Rhp7 | | 2.26 |
| SPBC27B12.05 | | zf-MYND type zinc finger protein | | 2.26 |
| SPAC8F11.07c | | nitrilase (predicted) | | 2.26 |
| SPAC23H4.01c | | arrestin/PY protein 1 | arrestin/PY protein 1 | 2.26 |
| SPAC11E3.05 | | membrane transporter (predicted) | | 2.25 |
| SPBC146.01 | *mug111* | sequence orphan | sequence orphan | 2.25 |
| SPAC16E8.17c | *sif1* | Sad1 interacting factor 1 | Sad1 interacting factor 1 | 2.25 |
| SPAC664.15 | *ntp1* | alpha,alpha-trehalase Ntp1 | alpha,alpha-trehalase Ntp1 | 2.25 |
| SPAC17G8.10c | *ppk24* | serine/threonine protein kinase Ppk24 | | 2.25 |
| SPAC977.13c | | peroxin Pex32 (predicted) | peroxin Pex32 (predicted) | 2.25 |
| SPAC513.06c | *mrp7* | mitochondrial ribosomal protein subunit L27 | mitochondrial ribosomal protein subunit L27 | 2.25 |
| SPBC56F2.05c | *urg2* | uracil phosphoribosyltransferase (predicted) | uracil phosphoribosyltransferase (predicted) | 2.25 |
| SPCC1682.11c | | dipeptidyl aminopeptidase (predicted) | dipeptidyl aminopeptidase (predicted) | 2.25 |
| SPCC417.11c | | GINS complex subunit Psf1 (predicted) | | 2.24 |
| SPAC1834.11c | *apc2* | anaphase-promoting complex subunit Apc2 | | 2.24 |
| SPMIT.04 | | transcription factor (predicted) | transcription factor (predicted) | 2.24 |
| SPCC24B10.14c | *mug51* | conserved fungal protein | conserved fungal protein | 2.24 |
| SPAC16A10.01 | *fap1* | L-pipecolate oxidase | L-pipecolate oxidase | 2.24 |
| SPBP8B7.18c | *vps1302* | chorein homolog | | 2.23 |
| SPCC417.07c | | thioesterase superfamily protein | | 2.23 |

| | | | | |
|---|---|---|---|---|
| SPAC1002.10c | *ned1* | lipin Ned1 | lipin | 2.23 |
| SPAC1565.07c | *ypt71* | GTPase Ypt71 | GTPase Ypt71 | 2.23 |
| SPBC713.06 | | hydrolase (predicted) | hydrolase (predicted) | 2.23 |
| SPAC140.01 | | AMP-activated protein kinase beta subunit (predicted) | AMP-activated protein kinase beta subunit (predicted) | 2.22 |
| SPBC19C2.04c | | S. pombe specific multicopy membrane protein family 1 | S. pombe specific multicopy membrane protein family 1 | 2.22 |
| SPBC1198.12 | *rhp16* | Rad16 homolog Rhp16 | Rad16 homolog Rhp16 | 2.22 |
| SPBC1711.12 | | conserved fungal protein | | 2.22 |
| SPBC646.12c | *gmh3* | alpha-1,2-galactosyltransferase Gmh3 | alpha-1,2-galactosyltransferase Gmh3 | 2.22 |
| SPAC7D4.05 | | trans-aconitate 3-methyltransferase (predicted) | trans-aconitate 3-methyltransferase (predicted) | 2.22 |
| SPAC2H10.02c | *pof11* | F-box protein Pof11 | F-box protein Pof11 | 2.22 |
| SPCC320.05 | *cit1* | citrate synthase (predicted) | citrate synthase | 2.22 |
| SPAC458.06 | | NAD/NADH kinase (predicted) | NAD/NADH kinase (predicted) | 2.21 |
| SPAC688.08 | *pku80* | Ku domain protein Pku80 | | 2.21 |
| SPAC144.08 | *mam3* | cell agglutination protein Mam3 | | 2.21 |
| SPBP35G2.06c | | DEP domain protein | | 2.21 |
| SPCC1450.12 | *gst2* | glutathione S-transferase Gst2 | glutathione S-transferase Gst2 | 2.21 |
| SPCC70.02c | *arb2* | argonaute binding protein 2 | argonaute binding protein 2 | 2.20 |
| SPMIT.05 | *ppk18* | serine/threonine protein kinase Ppk18 (predicted) | serine/threonine protein kinase Ppk18 (predicted) | 2.20 |
| SPAC29A4.12c | | DUF1776 family protein | DUF1776 family protein | 2.20 |
| SPBC1289.16c | | exocyst complex subunit Exo84 | exocyst complex subunit Exo84 | 2.20 |
| SPAC2F3.07c | | vacuolar protein involved in phosphoinositide metabolism | | 2.20 |
| SPAC644.08 | *mug66* | meiotically upregulated gene Mug66 | meiotically upregulated gene Mug66 | 2.19 |
| SPAC637.13c | | dipeptidyl aminopeptidase (predicted) | dipeptidyl aminopeptidase (predicted) | 2.19 |
| SPAC26H5.09c | | sequence orphan | sequence orphan | 2.19 |
| SPBC11B10.08 | | DUF89 family protein | DUF89 family protein | 2.18 |
| SPAC2E1P3.05c | *dnm1* | dynamin Dnm1 | dynamin Dnm1 | 2.18 |
| SPBC1685.01 | | esterase/lipase (predicted) | esterase/lipase (predicted) | 2.18 |
| SPCC622.03c | *cdc7* | serine/threonine protein kinase Cdc7 | | 2.18 |
| SPBC577.05c | *gpi18* | pig-V (predicted) | pig-V | 2.18 |
| SPCC74.04 | | | dubious | 2.17 |
| SPBC365.02c | | Niemann-Pick disease type C2 protein hE1 homolog | phosphatidylglycerol/phosphatidylinositol transfer protein (predicted) | 2.17 |
| SPAC589.04 | | pleckstrin homology domain protein | transcriptional coactivator (predicted) | 2.17 |

| | | | | |
|---|---|---|---|---|
| SPAC19G12.04 | *pas1* | cyclin Pas1 | cyclin Pas1 | 2.17 |
| SPBCPT2R1.06c | | leukotriene A-4 hydrolase (predicted) | leukotriene A-4 hydrolase (predicted) | 2.17 |
| SPBC3B9.06c | *gsk31* | serine/threonine protein kinase Gsk31 | | 2.17 |
| SPMIT.03 | *tco89* | TORC1 subunit Tco89 | sequence orphan | 2.17 |
| SPBC16A3.09c | | transcription factor (predicted) | transcription factor | 2.17 |
| SPCC162.10 | | cytoplasmic vesicle protein, Vid24 family | cytoplasmic vesicle protein, Vid24 family | 2.16 |
| SPCC757.03c | | nucleotide pyrophosphatase (predicted) | | 2.16 |
| SPBC1198.14c | *cmb1* | cytosine-mismatch binding protein 1 | cytosine-mismatch binding protein 1 | 2.16 |
| SPAC1952.15c | | plasma membrane protein involved in inositol lipid-mediated signaling | conserved fungal protein | 2.16 |
| SPBC1711.02 | *sid1* | PAK-related kinase Sid1 | PAK-related kinase Sid1 | 2.16 |
| SPAC23D3.11 | | amidase (predicted) | amidase (predicted) | 2.16 |
| SPCC794.04c | *mad2* | spindle checkpoint protein Mad2 | | 2.16 |
| SPCC132.03 | | manganese superoxide dismutase (AF069292) | manganese superoxide dismutase (AF069292) | 2.15 |
| SPBP35G2.10 | | human CCDC55 homolog | human CCDC55 homolog | 2.15 |
| SPBC354.11c | | sequence orphan | | 2.15 |
| SPBC1683.08 | | dihydrolipoamide S-succinyltransferase, e2 component of oxoglutarate dehydrogenase complex (predicted) | | 2.15 |
| SPBC800.13 | *mug74* | sequence orphan | | 2.15 |
| SPBC26H8.04c | | membrane transporter | membrane transporter | 2.15 |
| SPBC887.17 | *msp1* | mitochondrial GTPase Msp1 | | 2.15 |
| SPCPB16A4.02c | | SAGA complex subunit Spt20 (predicted) | SAGA complex subunit Spt20 (predicted) | 2.15 |
| SPAP27G11.05c | *pmp31* | plasma membrane proteolipid Pmp31 | plasma membrane proteolipid Pmp31 | 2.14 |
| SPAC4G8.03c | *gpa2* | heterotrimeric G protein alpha-2 subunit Gpa2 | heterotrimeric G protein alpha-2 subunit Gpa2 (PMID 1340462) | 2.14 |
| SPAC20G8.03 | *ggt2* | gamma-glutamyltranspeptidase Ggt2 | gamma-glutamyltranspeptidase Ggt2 | 2.14 |
| SPCC61.05 | | DUF221 family protein | DUF221 family protein | 2.14 |
| SPAC19B12.13 | | sequence orphan | sequence orphan | 2.14 |
| SPAC22H10.12c | | transcription factor (predicted) | | 2.14 |
| SPAPJ695.01c | *rec6* | meiotic recombination protein Rec6 | | 2.14 |
| SPBC19F8.04c | *vps16* | HOPS complex subunit Vps16 (predicted) | HOPS complex subunit Vps16 (predicted) | 2.13 |
| SPBC947.06c | | pseudogene | | 2.13 |
| SPAC13G6.15c | *fzo1* | mitochondrial fusion GTPase protein | | 2.13 |

| | | | | |
|---|---|---|---|---|
| SPAC1556.04c | *cgs2* | cAMP-specific phosphodiesterase Cgs2 | cAMP-specific phosphodiesterase Cgs2 | 2.13 |
| SPMIT.06 | | nucleoside 2-deoxyribosyltransferase (predicted) | nucleoside 2-deoxyribosyltransferase (predicted) | 2.13 |
| SPBC1709.11c | | ubiquitin-protein ligase E3 | ubiquitin-protein ligase E3 | 2.13 |
| SPBC660.05 | | succinate-CoA ligase (beta subunit) | succinate-CoA ligase (beta subunit) | 2.13 |
| SPBC83.03c | *ssm4* | p150-Glued | | 2.13 |
| SPCC1620.08 | | sequence orphan | sequence orphan | 2.12 |
| SPBP23A10.14c | *ypt7* | GTPase Ypt7 | | 2.12 |
| SPAC823.16c | | conserved protein (fungal and plant) | conserved protein (fungal and plant) | 2.12 |
| SPAC328.07c | | NADP-dependent oxidoreductase (predicted) | NADP-dependent oxidoreductase (predicted) | 2.12 |
| SPAC19G12.11 | *dak1* | dihydroxyacetone kinase Dak1 | dihydroxyacetone kinase Dak1 | 2.11 |
| SPAC139.05 | | DUF890 family protein | DUF890 family protein | 2.11 |
| SPAC2G11.09 | *mug122* | PX/PXA domain protein | PX/PXA domain protein | 2.11 |
| SPBC577.13 | *cid2* | caffeine induced death protein Cid2 | caffeine induced death protein Cid2 | 2.11 |
| SPBC609.01 | | C2 domain protein | C2 domain protein | 2.10 |
| SPBC2D10.17 | | vacuolar membrane zinc transporter (predicted) | vacuolar membrane zinc transporter (predicted) | 2.10 |
| SPAC22A12.01c | *meu8* | aldehyde dehydrogenase Meu8 (predicted) | betaine aldehyde dehydrogenase (predicted) | 2.10 |
| SPAC18G6.01c | *pkl1* | kinesin-like protein Pkl1 | kinesin-like protein Pkl1 | 2.10 |
| SPBC12C2.12c | | peptide N-glycanase (predicted) | | 2.10 |
| SPBC4.01 | *atg12* | autophagy associated protein Atg12 | autophagy associated protein Atg12 | 2.09 |
| SPAC1687.07 | *mam2* | pheromone p-factor receptor | pheromone p-factor receptor (PMID 1657593) | 2.09 |
| SPBC405.04c | *tas3* | RITS complex subunit 3 | | 2.09 |
| SPAC1296.06 | *scr1* | transcription factor Scr1 | | 2.09 |
| SPCC330.01c | *cdr1* | GIN4 family protein kinase Cdr1 | GIN4 family protein kinase Cdr1 | 2.09 |
| SPAC328.08c | | potassium ion/proton antiporter | potassium ion/proton antiporter | 2.08 |
| SPCC191.06 | | transcription factor, zf-fungal binuclear cluster type (predicted) | | 2.07 |
| SPBC1271.01c | | dubious | dubious | 2.07 |
| SPAC869.07c | *pof1* | F-box/WD repeat protein protein Pof1 | F-box protein Pof1 | 2.07 |
| SPCC576.01c | | transcription factor (predicted) | | 2.07 |
| SPAC959.05c | | mitochondrial intermembrane space protein sorting protein | | 2.06 |
| SPBC2A9.03 | *ucp8* | UBA/EH/EF hand domain protein Ucp8 | | 2.06 |

| | | | | |
|---|---|---|---|---|
| SPBC106.08c | *cmk1* | calcium/calmodulin-dependent protein kinase Cmk1 | calcium/calmodulin-dependent protein kinase Cmk1 | 2.06 |
| SPAPB1A11.03 | *nse2* | Smc5-6 complex non-SMC subunit 2 | Smc5-6 complex non-SMC subunit 2 (PMID 12966087) | 2.05 |
| SPBPB21E7.06 | *cox1* | cytochrome c oxidase 1 | cytochrome c oxidase 1; similar to S. cerevisiae Q0045 | 2.05 |
| SPAC869.01 | *pub3* | ubiquitin-protein ligase E3 | | 2.05 |
| SPAC144.16 | *thi2* | thiazole biosynthetic enzyme | | 2.05 |
| SPCC188.07 | | nicotinic acid plasma membrane transporter (predicted) | nicotinic acid plasma membrane transporter (predicted) | 2.05 |
| SPAC227.03c | *cwf16* | splicing factor (predicted) | | 2.05 |
| SPAC3A12.06c | | NAD/NADH kinase (predicted) | NAD/NADH kinase (predicted) | 2.05 |
| SPAC458.05 | | UPF0171 family protein | | 2.05 |
| SPCC1739.15 | *coq4* | ubiquinone biosynthesis protein Coq4 (predicted) | ubiquinone biosynthesis protein Coq4 | 2.04 |
| SPAC23D3.13c | | histone H4 variant | histone H4 variant | 2.04 |
| SPCP31B10.02 | | Der1-like (degradation in the ER) family | | 2.04 |
| SPCC330.04c | | MSP domain | MSP domain | 2.04 |
| SPAC1006.01 | *meu25* | sequence orphan | | 2.04 |
| SPAC6G10.10c | *apc11* | anaphase-promoting complex subunit Apc11 | anaphase-promoting complex subunit Apc11 | 2.03 |
| SPAC22E12.06c | *fis1* | mitochondrial fission protein Fis1 (predicted) | mitochondrial fission protein Fis1 (predicted) | 2.03 |
| SPBC16C6.03c | *gpi15* | pig-H (predicted) | | 2.03 |
| SPCC70.04c | *mug169* | sequence orphan | sequence orphan | 2.03 |
| SPAC23C4.18c | *dpm2* | dolichol-phosphate mannosyltransferase subunit 2 (predicted) | | 2.03 |
| SPAC3H5.04 | *mde2* | Mde2 protein | | 2.03 |
| SPBC1711.11 | | NAD dependent epimerase/dehydratase family protein | NAD dependent epimerase/dehydratase family protein | 2.03 |
| SPCC1682.15 | *cpp1* | protein farnesyltransferase beta subunit Cpp1 | protein farnesyltransferase beta subunit Cpp1 | 2.02 |
| SPBC646.15c | *fhl1* | fork head transcription factor Fhl1 | | 2.02 |
| SPBPB2B2.13 | | sequence orphan | | 2.02 |
| SPBC16A3.18 | | tRNA nucleotidyltransferase (predicted) | tRNA nucleotidyltransferase (predicted) | 2.02 |
| SPBC83.01 | *pzh1* | serine/threonine protein phosphatase Pzh1 | serine/threonine protein phosphatase Pzh1 | 2.02 |
| SPCC364.02c | | amino acid transporter (predicted) | amino acid transporter (predicted) | 2.02 |

| | | | | |
|---|---|---|---|---|
| SPAC1142.08 | | sequence orphan | sequence orphan | 2.02 |
| SPCC126.09 | | sequence orphan | | 2.01 |
| SPAC57A10.05c | *psf2* | GINS complex subunit Psf2 | | 2.01 |
| SPAC227.14 | | voltage-dependent anion-selective channel | voltage-dependent anion-selective channel | 2.01 |
| SPAC19B12.10 | | sphingolipid biosynthesis protein | sphingolipid biosynthesis protein | 2.01 |
| SPBC18H10.05 | | dubious | dubious | 2.01 |
| SPBC660.06 | *btn1* | battenin CLN3 family protein | battenin CLN3 family protein | 2.01 |
| SPCC1442.11c | *rep1* | MBF transcription factor complex subunit Rep1 | | 2.00 |
| SPCC1919.03c | *cdc10* | MBF transcription factor complex subunit Cdc10 | | 2.00 |
| SPBC409.12c | *gad8* | serine/threonine protein kinase Gad8 | serine/threonine protein kinase Gad8 (PMID 12805221) | 2.00 |
| SPBC28E12.06c | | nitrilase (predicted) | nitrilase (predicted) | 2.00 |
| SPBC25B2.03 | | membrane transporter (predicted) | membrane transporter (predicted) | 2.00 |
| SPAC1A6.11 | | sequence orphan | | 2.00 |
| SPBC1105.10 | | sequence orphan | sequence orphan | 2.00 |
| SPAC31G5.07 | *scn1* | TatD DNase family Scn1 | TatD DNase family Scn1 | 2.00 |
| SPCC18B5.11c | *win1* | MAP kinase kinase kinase Win1 | | 2.00 |
| SPBC1348.01 | | sequence orphan | sequence orphan | 1.99 |
| SPAC31G5.09c | *cbp3* | ubiquinol cytochrome-c reductase assembly protein Cbp3 | ubiquinol cytochrome-c reductase assembly protein Cbp3 | 1.99 |
| SPAC1952.09c | | sphingosine hydroxylase (predicted) | | 1.99 |
| SPCC757.04 | | pseudogene | | 1.99 |
| SPCC320.04c | *toc1* | Tor Complex Tor2 interacting protein 1 | | 1.98 |
| SPBC30D10.09c | | conserved fungal protein | conserved fungal protein | 1.98 |
| SPBC4B4.02c | *hem15* | ferrochelatase | ferrochelatase | 1.98 |
| SPBP4H10.17c | | conserved fungal protein | conserved fungal protein | 1.98 |
| SPAC1002.19 | *gpi7* | GPI anchor biosynthesis protein Gpi7 (predicted) | GPI anchor biosynthesis protein Gpi7 (predicted) | 1.97 |
| SPBC11C11.06c | *ccq1* | telomere maintenence protein | telomere maintenence protein | 1.97 |
| SPBC1D7.02c | *bis1* | stress response protein Bis1 | stress response protein Bis1 | 1.97 |
| SPBC216.03 | *tfb5* | transcription factor TFIIH complex subunit Tfb5 (predicted) | transcription factor TFIIH complex subunit Tfb5 (predicted) | 1.97 |
| SPCC1259.15c | *idp1* | isocitrate dehydrogenase Idp1 | isocitrate dehydrogenase Idp1 | 1.97 |

| | | | | |
|---|---|---|---|---|
| SPAPB1A11.01 | *asp1* | inositol hexakisphosphate kinase/inositol pyrophosphate synthase (predicted) | inositol hexakisphosphate kinase (predicted) | 1.97 |
| SPCC285.10c | *mug4* | sequence orphan | sequence orphan | 1.97 |
| SPBC543.05c | | transcription factor, zf-fungal binuclear cluster type (predicted) | transcription factor (predicted) | 1.97 |
| SPAC6G10.08 | *thp1* | uracil DNA N-glycosylase Thp1 | uracil DNA N-glycosylase Thp1 (PMID 12711670) | 1.97 |
| SPCC70.03c | *slu7* | splicing factor Slu7 | | 1.97 |
| SPAC328.04 | *cwc16* | complexed with Cdc5 protein Cwf16 | | 1.96 |
| SPCC1235.15 | *thi9* | thiamine transporter Thi9 | amino acid permease, unknown 2 | 1.96 |
| SPAPB8E5.10 | | membrane transporter | membrane transporter | 1.96 |
| SPAC13G7.13c | *mug184* | meiotically upregulated gene Mug184 | meiotically upregulated gene Mug184 | 1.96 |
| SPACUNK4.10 | | amino acid permease, unknown 15 | amino acid permease, unknown 15 | 1.96 |
| SPCC11E10.01 | *mug15* | sequence orphan | sequence orphan | 1.96 |
| SPAC3G6.09c | | aromatic aminotransferase (predicted) | aromatic aminotransferase (predicted) | 1.95 |
| SPBPB2B2.06c | *ypt4* | GTPase Ypt4 | GTPase Ypt4 | 1.95 |
| SPBC6B1.05c | | LEA domain protein | LEA domain protein | 1.95 |
| SPAC4H3.12c | | sequence orphan | | 1.95 |
| SPCC63.04 | *mug157* | conserved protein (fungal and bacterial) | conserved protein (fungal and bacterial) | 1.95 |
| SPAC144.10c | *mrp51* | mitochondrial ribosomal protein subunit L51-b (predicted) | mitochondrial ribosomal protein subunit L51-b (predicted) | 1.95 |
| SPAC29A4.11 | *mug67* | PPPDE peptidase family (predicted) | PPPDE peptidase family (predicted) | 1.95 |
| SPAC167.05 | *liz1* | pantothenate transporter | | 1.95 |
| SPCC320.03 | *prp16* | ATP-dependent RNA helicase Prp16 | | 1.95 |
| SPBC20F10.06 | | NLI interacting factor family | | 1.94 |
| SPCC1494.03 | *zrt1* | ZIP zinc transporter Zrt1 | | 1.94 |
| SPAC20G4.05c | *pvg5* | PvGal biosynthesis protein Pvg5 | PvGal biosynthesis protein Pvg5 | 1.94 |
| SPAC9E9.05 | *vps17* | retromer complex subunit Vps17 | retromer complex subunit Vps17 | 1.94 |
| SPBC36.10 | | conserved fungal protein | | 1.94 |
| SPCC965.09 | *hsf1* | transcription factor Hsf1 | transcription factor Hsf1 | 1.94 |
| SPBC530.05 | *grx3* | monothiol glutaredoxin Grx3 | monothiol glutaredoxin Grx3 | 1.94 |
| SPBC15D4.12c | *res2* | MBF transcription factor complex subunit Res2 | MBF transcription factor complex subunit Res2 | 1.93 |
| SPBC12C2.02c | | sequence orphan | sequence orphan | 1.93 |
| SPAP8A3.05 | *mde3* | serine/threonine protein kinase Mde3 | | 1.93 |
| SPAPYUG7.06 | *bgs2* | 1,3-beta-glucan synthase subunit Bgs2 | 1,3-beta-glucan synthase subunit Bgs2 | 1.92 |

| | | | | |
|---|---|---|---|---|
| SPBC16E9.17c | | RNA-binding protein | | 1.92 |
| SPBC21D10.09c | | WD repeat protein | | 1.92 |
| SPAC922.06 | *tsc1* | hamartin | hamartin | 1.92 |
| SPAC5D6.09c | | sequence orphan | | 1.92 |
| SPAC27D7.13c | *tbf1* | telomeric repeat binding factor Trf1 | | 1.91 |
| SPAC4G9.10 | *nep1* | NEDD8 protease Nep1 | | 1.91 |
| SPAC227.15 | | protein phosphatase inhibitor (predicted) | protein phosphatase inhibitor (predicted) | 1.91 |
| SPCC63.05 | *abc1* | ABC1 kinase family protein | | 1.91 |
| SPAC607.08c | *cip1* | RNA-binding protein Cip1 | | 1.91 |
| SPCC1183.05c | *csn1* | COP9/signalosome complex subunit Csn1 | | 1.91 |
| SPBC409.17c | | mitochondrial Rieske ISP assembly ATPase (predicted) | Rieske ISP assembly protein | 1.91 |
| SPBC32F12.08c | | MS ion channel protein 1 | | 1.91 |
| SPAC4D7.10c | *gnr1* | heterotrimeric G protein beta subunit Gnr1 | heterotrimeric G protein beta subunit Gnr1 | 1.91 |
| SPAC27E2.07 | *rga3* | GTPase activating protein Rga3 | GTPase activating protein Rga3 | 1.91 |
| SPAC1039.09 | *tfg1* | transcription factor TFIIF complex alpha subunit Tfg1 (predicted) | transcription factor TFIIF complex alpha subunit Tfg1 | 1.90 |
| SPAC1F3.09 | *tco1* | RTA1-like protein | RTA1-like protein | 1.90 |
| SPCC1020.10 | | membrane transporter | membrane transporter | 1.90 |
| SPBP4H10.16c | *btb1* | BTB/POZ domain protein Btb1 | BTB/POZ domain protein Btb1 | 1.90 |
| SPBC16D10.05 | *rad16* | DNA repair endonuclease XPF | DNA repair endonuclease XPF | 1.90 |
| SPCC757.02c | | peroxin Pex28/29 | peroxin Pex28/29 | 1.90 |
| SPAC25G10.03 | *tht2* | meiotically upregulated gene Mug22 | meiotically upregulated gene Mug22 | 1.90 |
| SPCC757.11c | | membrane transporter | membrane transporter | 1.89 |
| SPBC16E9.16c | | sequence orphan | | 1.89 |
| SPAC20G8.10c | | ubiquitin-protein ligase E3 (predicted) | | 1.89 |
| SPAC22A12.11 | | transcription factor (predicted) | | 1.89 |
| SPBP4H10.07 | *mok14* | alpha-1,3-glucan synthase Mok14 | alpha-1,3-glucan synthase Mok14 | 1.89 |
| SPBP35G2.12 | | transcription factor (predicted) | transcription factor (predicted) | 1.89 |
| SPBC2F12.05c | | sequence orphan | | 1.88 |
| SPBPB21E7.08 | | DUF1640 family protein | DUF1640 family protein | 1.88 |
| SPBC119.07 | *tip41* | TIP41-like type 2a phosphatase regulator Tip41 | TIP41-like type 2a phosphatase regulator Tip41 | 1.88 |

| | | | | |
|---|---|---|---|---|
| SPAC1F8.01 | *cox1102* | fusion cytochrome c oxidase assembly protein Cox1102, mitochondrial ribosomal protein Rsm2202 | | 1.88 |
| SPCC584.03c | | Mvp17/PMP22 family protein 1 | Mvp17/PMP22 family | 1.88 |
| SPBC18A7.01 | | conserved eukaryotic protein | conserved eukaryotic protein | 1.88 |
| SPAC22H12.05c | | RNA polymerase II associated Paf1 complex (predicted) | | 1.88 |
| SPAC144.13c | *tel2* | Tel2/Rad-5/Clk-2 family protein Tel2 | nuclear telomere cap complex subunit (predicted) | 1.88 |
| SPCC794.03 | | nuclease, XP-G family | nuclease, XP-G family | 1.88 |
| SPCC285.11 | | sequence orphan | | 1.87 |
| SPAC14C4.07 | | serine/threonine protein phosphatase (predicted) | | 1.87 |
| SPCC965.06 | | vacuolar sorting protein (predicted) | | 1.87 |
| SPCC70.10 | | iron sulfur cluster assembly protein (predicted) | | 1.87 |
| SPBC428.16c | | phospholipid scramblase | scramblase | 1.87 |
| SPAC6F12.12 | *cdd1* | cytidine deaminase Ccd1 | cytidine deaminase Pcd1 | 1.87 |
| SPBC1778.03c | *pcp1* | pericentrin Pcp1 | pericentrin Pcp1 | 1.87 |
| SPAC15F9.01c | | S. pombe specific DUF999 family protein 1 | S. pombe specific DUF999 family protein 1 | 1.87 |
| SPBC947.08c | | glycoprotein (predicted) | | 1.86 |
| SPAC5D6.06c | | inorganic pyrophosphatase | inorganic pyrophosphatase | 1.86 |
| SPAC23G3.02c | | mitochondrial iron ion transporter | mitochondrial iron ion transporter | 1.86 |
| SPCC63.14 | | tubulin specific chaperone cofactor C (predicted) | tubulin specific chaperone cofactor C (predicted) | 1.86 |
| SPAC25B8.18 | *sds23* | inducer of sexual development Sds23/Moc1 | | 1.86 |
| SPCC18B5.05c | | THIJ/PFPI family peptidase (predicted) | THIJ/PFPI family peptidase (predicted) | 1.86 |
| SPCC16C4.03 | | proline dehydrogenase | proline dehydrogenase | 1.86 |
| SPAC19A8.05c | | mitochondrial DNA binding endonuclease | | 1.86 |
| SPAC6B12.02c | | sequence orphan | sequence orphan | 1.86 |
| SPCC16A11.04 | | phosphatidylinositol-3-phosphatase (predicted) | phosphatidylinositol-3-phosphatase (predicted) | 1.86 |
| SPBC4F6.06 | | caspase | caspase | 1.85 |
| SPAP27G11.16 | *duo1* | DASH complex subunit Duo1 (predicted) | | 1.85 |
| SPAC10F6.04 | *aah2* | alpha-amylase homolog Aah2 | alpha-amylase homolog Aah2 | 1.85 |
| SPBC1289.11 | *mug30* | ubiquitin-protein ligase E3 | | 1.85 |

| | | | | |
|---|---|---|---|---|
| SPAC6B12.08 | *rap1* | telomere binding protein Rap1 | | 1.85 |
| SPAPB8E5.05 | | uracil permease (predicted) | | 1.85 |
| SPCC320.06 | | phosphatidylserine decarboxylase (predicted) | phosphatidylserine decarboxylase (predicted) | 1.85 |
| SPAC6C3.08 | *mus7* | DNA repair protein Mus7/Mms22 | DNA repair protein Mus7 | 1.85 |
| SPAC11D3.09 | | ubiquitin-protein ligase E3 (predicted) | | 1.85 |
| SPCC191.05c | *tim40* | TIM22 inner membrane protein import complex subunit Tim40 | TIM22 inner membrane protein import complex subunit Tim40 | 1.85 |
| SPACUNK12.02c | | isocitrate lyase | isocitrate lyase | 1.84 |
| SPAC1420.01c | | DUF59 family protein | DUF59 family protein | 1.84 |
| SPAC22F3.09c | | sequence orphan | | 1.84 |
| SPBC21B10.02 | | palmitoyltransferase (predicted) | | 1.84 |
| SPAC2F3.08 | *pmp1* | dual-specificity MAP kinase phosphatase Pmp1 | dual-specificity MAP kinase phosphatase Pmp1 | 1.83 |
| SPAC1142.03c | *mug65* | spore wall assembly protein (predicted) | spore wall assembly protein (predicted) | 1.83 |
| SPBC800.02 | *tsf1* | mitochondrial translation elongation factor EF-Ts Tsf1 | mitochondrial translation elongation factor EF-Ts Tsf1 (PMID 15695360) | 1.83 |
| SPBC1711.17 | *tra1* | phosphatidylinositol kinase-related protein Tra1 | | 1.83 |
| SPAC186.07c | | mitochondrial GTPase Guf1 (predicted) | mitochondrial GTPase Guf1 (predicted) | 1.83 |
| SPAC26A3.11 | | human GRASP protein homolog (predicted) | human GRASP protein homolog (predicted) | 1.83 |
| SPBC800.11 | | NADH pyrophosphatase (predicted) | | 1.83 |
| SPBC685.03 | *sre1* | sterol regulatory element binding protein Sre1 | | 1.83 |
| SPBC23G7.16 | | WD repeat protein, human WDR7 ortholog | | 1.83 |
| SPAC17A2.06c | *coq6* | monooxygenase Coq6 | | 1.82 |
| SPAC12G12.16c | | inositol phosphosphingolipid phospholipase C (predicted) | | 1.82 |
| SPBC902.05c | *sgt1* | SGT1 family transcriptional regulator Sgt1 | SGT1 family protein Sgt1 | 1.82 |
| SPAPYUK71.03c | | nifs homolog | nifs homolog | 1.82 |
| SPBC1773.05c | *whi5* | cell cycle transcriptional repressor Whi5 | cell cycle transcriptional repressor Whi5 | 1.82 |
| SPBC646.13 | | protein disulfide isomerase (predicted) | protein disulfide isomerase (predicted) | 1.82 |
| SPAC22A12.17c | | mitochondrial ribosomal protein subunit L9 (predicted) | mitochondrial ribosomal protein subunit L9 (predicted) | 1.82 |

| | | | | |
|---|---|---|---|---|
| SPAC56E4.02c | | aminopeptidase (predicted) | aminopeptidase (predicted) | 1.82 |
| SPBC11C11.03 | *atg15* | triacylglycerol lipase Atg15 (predicted) | triacylglycerol lipase Atg15 (predicted) | 1.81 |
| SPAC18B11.05 | | serine/threonine protein kinase (predicted) | serine/threonine protein kinase (predicted) | 1.81 |
| SPBC36B7.05c | | RNA-binding protein | RNA-binding protein | 1.81 |
| SPAC2E12.05 | | sequence orphan | sequence orphan | 1.81 |
| SPBC21B10.12 | *vps8* | WD repeat protein Vps8 | WD repeat protein Vps8 | 1.81 |
| SPBPB2B2.11 | | 6-phosphofructo-2-kinase (predicted) | 6-phosphofructo-2-kinase (predicted) | 1.81 |
| SPAC4H3.08 | *vps41* | vacuolar protein sorting-associated protein Vps41 | vacuolar protein sorting-associated protein Vps41 | 1.80 |
| SPCPJ732.03 | *isa2* | iron-sulfur protein Isa2 | | 1.80 |
| SPAC1F3.02c | | sequence orphan | sequence orphan | 1.80 |
| SPAC869.06c | | AP-1 accessory protein (predicted) | | 1.80 |
| SPAC12B10.13 | *coq9* | ubiquinone biosynthesis protein Coq9 (predicted) | ubiquinone biosynthesis protein Coq9 (predicted) | 1.79 |
| SPBC2D10.06 | | dubious | dubious | 1.79 |
| SPAC18B11.04 | *apc10* | anaphase-promoting complex subunit Apc10 | | 1.79 |
| SPAC13F5.07c | *snf5* | chromatin remodeling complex subunit Snf5 (predicted) | chromatin remodeling complex subunit Snf5 (predicted) | 1.79 |
| SPAC1782.03 | | ENTH domain protein (predicted) | conserved fungal protein | 1.79 |
| SPAC11E3.09 | | DUF1941 family protein | DUF1941 family protein | 1.79 |
| SPCC1919.04 | *set6* | histone lysine methyltransferase Set6 (predicted) | | 1.79 |
| SPAC26F1.11 | *ufd1* | Cdc48-Ufd1-Npl4 complex component Ufd1 (predicted) | | 1.79 |
| SPCC338.04 | *rhp14* | XP-A family homolog Rhp14 | XP-A family homolog Rhp14 | 1.78 |
| SPBC19C2.05 | | ATP-dependent RNA helicase, spliceosomal (predicted) | ATP-dependent RNA helicase, spliceosomal (predicted) | 1.78 |
| SPCC338.12 | | DUF1748 family protein | DUF1748 family protein | 1.78 |
| SPCC1620.04c | *cdc24* | DNA replication protein Cdc24 | DNA replication protein Cdc24 | 1.78 |
| SPBC12D12.04c | | conserved fungal protein | conserved fungal protein | 1.78 |
| SPCC1223.09 | *lig4* | DNA ligase Lig4 | DNA ligase Lig4 | 1.78 |
| SPCC548.07c | | phosphoinositide binding protein | phosphoinositide binding protein | 1.78 |
| SPAC1039.05c | *ubx2* | UBX domain protein Ubx2 | UBX domain protein Ubx2 | 1.78 |
| SPAC1834.08 | *mto1* | MT organizer Mto1 | MT organizer Mto1 | 1.78 |
| SPCC1235.14 | | inorganic phosphate transporter (predicted) | inorganic phosphate transporter (predicted) | 1.78 |

| | | | | |
|---|---|---|---|---|
| SPAPB17E12.09 | *mcp3* | sequence orphan | sequence orphan | 1.77 |
| SPAC824.07 | | inorganic anion exchanger (predicted) | | 1.77 |
| SPBC530.07c | | dubious | | 1.77 |
| SPAC2C4.15c | *cdc16* | two-component GAP Cdc16 | two-component GAP Cdc16 | 1.77 |
| SPAC25H1.03 | | phosphatase activator (predicted) | | 1.77 |
| SPAP7G5.06 | | pseudogene (predicted) | | 1.77 |
| SPAC212.07c | *pck2* | protein kinase C (PKC)-like Pck2 | | 1.77 |
| SPAC1805.10 | *ubc1* | ubiquitin conjugating enzyme Ubc1 | | 1.77 |
| SPBC713.02c | | transcription factor, zf-fungal binuclear cluster type (predicted) | | 1.77 |
| SPAC11E3.02c | | mitochondrial DNA binding endonuclease | | 1.76 |
| SPBC1773.03c | *rad15* | transcription factor TFIIH complex subunit Rad15 | transcription factor TFIIH complex subunit Rad15 | 1.76 |
| SPBC23G7.13c | | FAD synthetase | FAD synthetase | 1.76 |
| SPBC839.02 | *sec8* | exocyst complex subunit Sec8 | exocyst complex subunit Sec8 | 1.76 |
| SPAC11G7.03 | *itr2* | MFS myo-inositol transporter | MFS myo-inositol transporter | 1.76 |
| SPAC4F10.02 | | dienelactone hydrolase family | | 1.76 |
| SPAC27F1.05c | *bdp1* | transcription factor TFIIIB complex subunit Bdp1 (predicted) | transcription factor TFIIIB complex subunit Bdp1 (predicted) | 1.76 |
| SPAC22H10.02 | | conserved fungal protein | conserved fungal protein | 1.76 |
| SPAC1D4.02c | | sequence orphan | | 1.76 |
| SPAC10F6.14c | | IMP 5'-nucleotidase (predicted) | | 1.76 |
| SPAC1B3.20 | *pep3* | ubiquitin-protein ligase E3 (predicted) | ubiquitin-protein ligase E3 (predicted) | 1.76 |
| SPCC1322.08 | *meu15* | sequence orphan | sequence orphan | 1.75 |
| SPCC962.01 | | human hmmtag2 homolog | human hmmtag2 homolog | 1.75 |
| SPBC651.09c | *stg1* | SM22/transgelin-like actin modulating protein Stg1 | SM22/transgelin-like actin modulating protein Stg1 | 1.75 |
| SPAC2C4.07c | *brf1* | transcription factor TFIIIB complex subunit Brf1 | | 1.75 |
| SPAC11E3.06 | *spn7* | septin Spn7 | | 1.75 |
| SPBC800.07c | | human ZNF277P homolog | human ZNF277P homolog | 1.75 |
| SPBC17G9.13c | *rga6* | GTPase activating protein Rga6 | GTPase activating protein Rga6 | 1.75 |
| SPAC4G8.04 | *ago1* | argonaute | argonaute | 1.75 |
| SPBC26H8.01 | | GRAM domain protein | | 1.75 |
| SPAC6C3.04 | *wtf7* | wtf element Wtf7 | wtf element Wtf7 | 1.74 |
| SPCC1281.04 | | sequence orphan | sequence orphan | 1.74 |

| | | | | |
|---|---|---|---|---|
| SPBC21B10.11 | *pep7* | prevacuole/endosomal FYVE tethering component Pep7 (predicted) | prevacuole/endosomal FYVE tethering component Pep7 (predicted) | 1.74 |
| SPBP16F5.03c | *vps45* | vacuolar sorting protein Vps 45 (predicted) | vacuolar sorting protein Vps 45 | 1.74 |
| SPAC22F8.02c | | DuF1740 family protein | | 1.74 |
| SPAC23C4.12 | | sequence orphan | sequence orphan | 1.74 |
| SPBC1683.12 | *zfs1* | CCCH tandem zinc finger protein, human Tristetraprolin homolog Zfs1 | | 1.74 |
| SPBC21.02 | | synoviolin homolog | | 1.74 |
| SPBC106.13 | | alpha,alpha-trehalose-phosphate synthase (predicted) | alpha,alpha-trehalose-phosphate synthase (predicted) | 1.74 |
| SPCC14G10.04 | *cut7* | kinesin-like protein Cut7 | kinesin-like protein Cut7 | 1.74 |
| SPAC26F1.01 | | RNA-binding protein | | 1.74 |
| SPAC6F6.18c | | DUF1640 family protein | DUF1640 family protein | 1.73 |
| SPAC6C3.03c | | DNAJ domain protein, DNAJC11 family | DNAJ domain protein, DNAJC11 family | 1.73 |
| SPAC19D5.01 | *maf1* | repressor of RNA polymerase III Maf1 (predicted) | repressor of RNA polymerase III Maf1 (predicted) | 1.73 |
| SPCC4F11.02 | | conserved fungal protein | conserved fungal protein | 1.73 |
| SPBC359.06 | *bud6* | actin interacting protein 3 homolog Bud6 | | 1.73 |
| SPBC27B12.14 | *gdi1* | GDP dissociation inhibitor Gdi1 (predicted) | GDP dissociation inhibitor Gdi1 (predicted) | 1.73 |
| SPAC23C11.06c | | sterol binding ankyrin repeat protein | sterol binding ankyrin repeat protein | 1.73 |
| SPCC1223.12c | *fab1* | 1-phosphatidylinositol-3-phosphate 5-kinase Fab1 | 1-phosphatidylinositol-3-phosphate 5-kinase Fab1 (PMID 10567352) | 1.72 |
| SPBC1289.10c | *mug178* | mitochondrial ribosomal protein subunit L51-b | | 1.72 |
| SPBC6B1.02 | *sod22* | plasma membrane alkali metal cation/H+ antiporter Sod22 | plasma membrane alkali metal cation/H+ antiporter Sod22 | 1.72 |
| SPBC211.07c | | metallopeptidase | metallopeptidase | 1.72 |
| SPCC1795.07 | *spn5* | septin Spn5 | septin Spn5 | 1.72 |
| SPAC3H1.11 | | DNAJ domain protein Jac1 (predicted) | DNAJ domain protein Jac1 (predicted) | 1.72 |
| SPBC543.03c | | 6-phosphofructo-2-kinase | 6-phosphofructo-2-kinase | 1.72 |
| SPBC13G1.05 | *sod2* | CPA1 sodium ion/proton antiporter | CPA1 sodium ion/proton antiporter (PMID 8643524) | 1.72 |
| SPBC1271.09 | | ABC1 kinase family protein | ABC1 kinase family protein | 1.72 |
| SPAPJ691.02 | *tps1* | alpha,alpha-trehalose-phosphate synthase [UDP-forming] | alpha,alpha-trehalose-phosphate synthase [UDP-forming] | 1.72 |
| SPAC977.17 | | microfibrillar-associated protein family protein | microfibrillar-associated protein familt protein | 1.72 |

| | | | | |
|---|---|---|---|---|
| SPAC167.06c | *did4* | vacuolar sorting protein Did4 | | 1.71 |
| SPAC25H1.02 | *rsp1* | random septum position protein Rsp1 | | 1.71 |
| SPCC330.02 | | ubiquitin family protein, human UBTD1 homolog | ubiquitin family protein, human UBTD1 homolog | 1.71 |
| SPBP8B7.30c | | sulfate transporter (predicted) | | 1.71 |
| SPCC10H11.02 | | CPA1 sodium ion/proton antiporter | CPA1 sodium ion/proton antiporter | 1.71 |
| SPBC17A3.02 | | TAP42 family protein (predicted) | TAP42 family protein (predicted) | 1.71 |
| SPCP20C8.03 | *vam6* | guanyl-nucleotide exchange factor Vam6 | guanyl-nucleotide exchange factor Vma6 | 1.71 |
| SPAC630.07c | *vps13a* | chorein homolog | chorein homolog | 1.70 |
| SPAC1002.20 | | DUF408 family protein | DUF408 family protein | 1.70 |
| SPAC977.16c | *git3* | G-protein coupled receptor Git3 | G-protein coupled receptor Git3 | 1.70 |
| SPCC1494.08c | | non-specific DNA binding protein Spt2 (predicted) | non-specific DNA binding protein Spt2 (predicted) | 1.70 |
| SPBC1718.06 | | hydroxyacid dehydrogenase (predicted) | hydroxyacid dehydrogenase (predicted) | 1.70 |
| SPBC337.06c | | DUF747 family protein | | 1.70 |
| SPCC576.06c | *ubc11* | ubiquitin conjugating enzyme E2-C | ubiquitin conjugating enzyme E2-C (PMID 12724408) | 1.70 |
| SPAC24C9.06c | | sequence orphan | sequence orphan | 1.70 |
| SPBC2G2.07c | *pyp3* | protein-tyrosine phosphatase Pyp3 | protein-tyrosine phosphatase Pyp3 | 1.70 |
| SPAC167.07c | | sequence orphan | | 1.70 |
| SPAC27D7.03c | *gpi8* | pig-K | pig-K | 1.70 |
| SPCC4B3.01 | | DUF1752 family protein | DUF1752 family protein | 1.70 |
| SPBC337.16 | *spf38* | splicing factor Spf38 | splicing factor Spf38 | 1.70 |
| SPAC4A8.04 | | GTPase Gem1 | GTPase Gem1 (PMID 12482879) | 1.70 |
| SPAC4G9.14 | | 2',3'-cyclic-nucleotide 3'-phosphodiesterase (predicted) | 2',3'-cyclic-nucleotide 3'-phosphodiesterase (predicted) | 1.70 |
| SPAC18G6.09c | | chromosome segregation protein (predicted) | chromosome segregation protein (predicted) | 1.70 |
| SPCC1450.13c | *tom13* | mitochondrial TOM complex subunit Tom13 | | 1.70 |
| SPBC1921.04c | *ncs1* | related to neuronal calcium sensor Ncs1 | related to neuronal calcium sensor Ncs1 | 1.69 |
| SPAPB1E7.08c | *meu22* | amino acid permease, unknown 11 | | 1.69 |
| SPAC12B10.01c | *sck1* | serine/threonine protein kinase Sck1 | serine/threonine protein kinase Sck1 | 1.69 |
| SPCC1450.07c | | mannosyltransferase complex subunit (predicted) | mannosyltransferase complex subunit (predicted) | 1.69 |

| | | | | |
|---|---|---|---|---|
| SPAC186.02c | | ER membrane protein, BIG1 family (predicted) | ER membrane protein, BIG1 family (predicted) | 1.69 |
| SPBC15D4.11c | *swi2* | Swi5 complex subunit Swi2 | Swi5 complex subunit Swi2 | 1.69 |
| SPBP4H10.19c | | 26S proteasome regulator (predicted) | 26S proteasome regulator (predicted) | 1.69 |
| SPBC215.13 | *par2* | protein phosphatase regulatory subunit Par2 | protein phosphatase regulatory subunit Par2 | 1.69 |
| SPAC3H8.04 | | sequence orphan | | 1.69 |
| SPAC17C9.06 | | steroid reductase (predicted) | steroid reductase (predicted) | 1.69 |
| SPBC1604.01 | *cwf23* | DNAJ domain protein Cwf23 | | 1.69 |
| SPCC777.02 | *pmh1* | transcription factor TFIIH complex subunit Pmh1 | | 1.69 |
| SPBC23G7.11 | | human down-regulated in multiple cancers-1 homolog 1 | human down-regulated in multiple cancers-1 homolog 1 | 1.69 |
| SPCC895.08c | *pap1* | transcription factor Caf3 | transcription factor Caf3 | 1.69 |
| SPBP8B7.07c | | conserved fungal protein | | 1.69 |
| SPAC1F7.03 | | dubious | dubious | 1.69 |
| SPAC1F7.06 | | pseudogene | pseudogene | 1.68 |
| SPAC22H12.01c | | mitochondrial translation termination factor | | 1.68 |
| SPBP19A11.07c | *hhp2* | serine/threonine protein kinase Hhp2 | serine/threonine protein kinase Hhp2 (PMID 8026462) | 1.68 |
| SPAC1751.04 | | conserved fungal protein | | 1.68 |
| SPCC23B6.01c | | human CAND1/TIP120 ortholog | TATA binding protein interacting protein (predicted) | 1.68 |
| SPAPB2B4.07 | | FHA domain protein Far10 (predicted) | | 1.68 |
| SPBC19C7.04c | *pmd1* | leptomycin efflux transporter Pmd1 | leptomycin efflux transporter Pmd1 | 1.68 |
| SPBC947.10 | *mrp10* | mitochondrial ribosomal protein subunit Mrp10 | mitochondrial ribosomal protein subunit Mrp10 | 1.67 |
| SPBC14C8.01c | *atp10* | F1-F0 ATPase assembly protein (predicted) | F1-F0 ATPase assembly protein | 1.67 |
| SPAC2E1P3.02c | | transcription factor (predicted) | transcription factor (predicted) | 1.67 |
| SPBC106.03 | *hsp16* | heat shock protein Hsp16 | | 1.67 |
| SPCC1795.06 | *git1* | C2 domain protein Git1 | | 1.67 |
| SPAC29E6.05c | *myo2* | myosin II heavy chain | myosin II heavy chain | 1.67 |
| SPBC24C6.06 | *scd2* | scaffold protein Scd2 | scaffold protein Scd2 | 1.67 |
| SPCC622.15c | | SAGA complex subunit Sgf29 (predicted) | | 1.67 |
| SPBC29B5.01 | | human ZC3H3 homolog | | 1.67 |
| SPBC11C11.12 | *rec27* | meiotic recombination protein Rec27 | | 1.67 |

| | | | | |
|---|---|---|---|---|
| SPBP23A10.04 | | cytoskeletal signaling protein | cytoskeletal signaling protein | 1.67 |
| SPAC57A10.11c | *ure2* | urease | urease (PMID 9301025) | 1.66 |
| SPBP8B7.27 | | amidohydrolase | amidohydrolase | 1.66 |
| SPCC24B10.02c | *gap1* | GTPase activating protein Gap1 | | 1.66 |
| SPBC359.05 | *pin1* | peptidyl-prolyl cis-trans isomerase Pin1 | peptidyl-prolyl cis-trans isomerase Pin1 | 1.66 |
| SPAC17G6.08 | | | dubious | 1.66 |
| SPBC1347.11 | *pst1* | Clr6 histone deacetylase complex subunit Pst1 | | 1.66 |
| SPAC23E2.03c | | RanGTP-binding protein (predicted) | RanGTP-binding protein (predicted) | 1.66 |
| SPBC106.10 | | SAGA complex subunit (predicted) | | 1.66 |
| SPAC644.07 | *mad3* | mitotic spindle checkpoint protein Mad3 | mitotic spindle checkpoint protein Mad3 | 1.66 |
| SPBC27B12.08 | *ppk21* | serine/threonine protein kinase Ppk21 | | 1.66 |
| SPCC16A11.15c | *pcr1* | transcription factor Pcr1 | transcription factor Pcr1 | 1.65 |
| SPAC4F8.08 | *cwf15* | complexed with Cdc5 protein Cwf15 | | 1.65 |
| SPAC22A12.08c | *csn2* | COP9/signalosome complex subunit Csn2 | COP9/signalosome complex subunit Csn2 (PMID 11854407) | 1.65 |
| SPAC12G12.11c | *dot2* | EAP30 family protein Dot2 | | 1.65 |
| SPAC9E9.17c | | conserved fungal protein | | 1.65 |
| SPBC8D2.19 | *alo1* | D-arabinono-1,4-lactone oxidase | D-arabinono-1,4-lactone oxidase | 1.65 |
| SPBC119.09c | *apc5* | anaphase-promoting complex subunit Apc5 | anaphase-promoting complex subunit Apc5 (PMID 12477395) | 1.65 |
| SPCC790.02 | *pdt1* | Nramp family manganese ion transporter | Nramp family manganese ion transporter | 1.65 |
| SPAC14C4.03 | *tea4* | tip elongation aberrant protein Tea4 | tip elongation aberrant protein Tea4 | 1.65 |
| SPCC24B10.03 | | guanyl-nucleotide exchange factor (predicted) | guanyl-nucleotide exchange factor | 1.65 |
| SPAC1783.07c | | urea transporter (predicted) | urea transporter (predicted) | 1.65 |
| SPCC1259.09c | *pku70* | Ku domain protein Pku70 | Ku domain protein Pku70 | 1.65 |
| SPAC212.04c | | TRAPP complex subunit Bet3 (predicted) | TRAPP complex subunit Bet3 (predicted) | 1.64 |
| SPAC20G4.02c | *cdc14* | SIN component Cdc14 | | 1.64 |
| SPAC22F8.05 | | cyclin pho85 family | | 1.64 |
| SPBC19C2.09 | *zds1* | zds family protein Zds1 | zds family protein Zds1 | 1.64 |
| SPAC11H11.02c | | GTPase regulator (predicted) | | 1.64 |
| SPAC27D7.11c | | dubious | | 1.64 |

| | | | | |
|---|---|---|---|---|
| SPCC1322.14c | *cho1* | phosphatidyl-N-methylethanolamine N-methyltransferase (predicted) | | 1.64 |
| SPBC609.03 | | calreticulin/calnexin homolog | | 1.64 |
| SPAC56F8.15 | | frataxin homolog | frataxin homolog | 1.64 |
| SPCC1919.14c | | leucine carboxyl methyltransferase (predicted) | | 1.63 |
| SPAC17G6.02c | | succinate-CoA ligase alpha subunit | succinate-CoA ligase (alpha subunit) | 1.63 |
| SPAC19G12.03 | | dubious | sequence orphan | 1.63 |
| SPBC11C11.01 | | 5-amino-6-(5-phosphoribosylamino) uracil reductase | | 1.63 |
| SPAC10F6.06 | | NADPH cytochrome reductase (predicted) | NADPH cytochrome reductase | 1.63 |
| SPAC869.09 | *tps2* | trehalose-phosphate synthase Tps2 (predicted) | trehalose-phosphate synthase Tps2 (predicted) | 1.63 |
| SPBC1685.05 | | sequence orphan | sequence orphan | 1.63 |
| SPAC20G8.02 | *mit1* | SHREC complex subunit Mit1 | | 1.63 |
| SPBP4G3.03 | *cdc12* | formin Cdc12 | formin Cdc12 | 1.63 |
| SPAC139.03 | | RNA-binding protein, G-patch type (predicted) | RNA-binding protein (predicted) | 1.63 |
| SPCC1322.05c | | HEAT repeat protein, unknown biological role | HEAT repeat protein, unknown biological role | 1.63 |
| SPBC2G2.01c | *mug162* | sequence orphan | sequence orphan | 1.62 |
| SPAC23A1.18c | | serine protease (predicted) | | 1.62 |
| SPCC1223.10c | | sequence orphan | | 1.62 |
| SPCP31B10.06 | *oar2* | 3-oxoacyl-[acyl-carrier-protein] reductase Oar2 (predicted) | 3-oxoacyl-[acyl-carrier-protein] reductase Oar2 (predicted) | 1.62 |
| SPBP18G5.03 | | transcription factor, zf-fungal binuclear cluster type (predicted) | | 1.62 |
| SPBC1706.02c | *cdt2* | WD repeat protein Cdt2 | WD repeat protein Cdt2 | 1.62 |
| SPCC1235.08c | | carboxypeptidase (predicted) | | 1.62 |
| SPBC2D10.18 | | ubiquitin-protein ligase E3 (predicted) | ubiquitin-protein ligase E3 (predicted) | 1.61 |
| SPAP19A11.05c | | sequence orphan | sequence orphan | 1.61 |
| SPAC1006.04c | *ppk31* | serine/threonine protein kinase Ppk31 (predicted) | | 1.61 |
| SPBC21C3.03 | *spn6* | septin Spn6 | septin Spn6 | 1.61 |
| SPBC26H8.05c | | conserved fungal protein | conserved fungal protein | 1.61 |
| SPBC428.10 | *git7* | SGT1-like protein Git7 | | 1.60 |
| SPAC1952.13 | *lvs1* | beige protein homolog | | 1.60 |

| | | | | |
|---|---|---|---|---|
| SPCC191.10 | | zf-FYVE type zinc finger protein | | 1.60 |
| SPAC1F5.09c | *exg2* | glucan 1,3-beta-glucosidase Exg2 | glucan 1,3-beta-glucosidase Exg2 | 1.60 |
| SPBC32F12.03c | | DUF1769 family protein | | 1.60 |
| SPAC17G8.12 | *cut4* | anaphase-promoting complex subunit Apc1 | anaphase-promoting complex subunit Apc1 | 1.60 |
| SPAC644.11c | *csx1* | RNA-binding protein Csx1 | RNA-binding protein Csx1 | 1.60 |
| SPBC20F10.02c | *sid2* | Sid2p-Mob1p kinase complex subunit Sid2 | Sid2p-Mob1p kinase complex | 1.60 |
| SPCC1753.02c | *uba5* | NEDD8 activating enzyme (predicted) | NEDD8 activating enzyme (predicted) | 1.60 |
| SPAC21E11.03c | *mug33* | conserved fungal protein | conserved fungal protein | 1.60 |
| SPCC191.04c | *ctr5* | copper transporter complex subunit Ctr5 (predicted) | copper transporter complex subunit Ctr5 (predicted) | 1.60 |
| SPAC4G8.12c | *sxa2* | serine carboxypeptidase Sxa2 | serine carboxypeptidase Sxa2 | 1.59 |
| SPBC106.09 | *pvg2* | galactose residue biosynthesis protein Pvg2 | galactose residue biosynthesis protein Pvg2 | 1.59 |
| SPAC1F3.10c | *sec18* | secretory pathway protein Sec18 (predicted) | secretory pathway protein Sec18 (predicted) | 1.59 |
| SPBC3H7.02 | *otu1* | ubiquitin-specific protease (predicted) | ubiquitin-specific protease (predicted) | 1.59 |
| SPACUNK4.17 | | sequence orphan | sequence orphan | 1.59 |
| SPBC887.18c | *jmj1* | Jmj1 protein | Jmj1 protein | 1.59 |
| SPBC2D10.05 | *ctr4* | copper transporter complex subunit Ctr4 | copper transporter complex subunit Ctr4 (PMID 11274192) | 1.59 |
| SPCC1919.10c | *vti1* | SNARE Vti1 | | 1.59 |
| SPBC1198.13c | *tfa2* | transcription factor TFIIE beta subunit Tfa2 | transcription factor TFIIE beta subunit Tfa2 | 1.59 |
| SPAC23H3.04 | *arg3* | ornithine carbamoyltransferase Arg3 | ornithine carbamoyltransferase Arg3 | 1.59 |
| SPBC19C7.03 | | CASP family protein | CASP family protein | 1.59 |
| SPAC8C9.03 | | spermidine family transporter (predicted) | | 1.59 |
| SPBC3E7.08c | *tmp1* | thymidylate kinase Tmp1 | thymidylate kinase Tmp1 | 1.58 |
| SPAC24C9.13c | | steroid dehydrogenase (predicted) | steroid dehydrogenase (predicted) | 1.58 |
| SPAC869.08 | *vip1* | RNA-binding protein Vip1 | RNA-binding protein Vip1 | 1.58 |
| SPBPJ4664.03 | *snf22* | ATP-dependent DNA helicase Snf22 | ATP-dependent DNA helicase Snf22 | 1.58 |
| SPCC162.03 | *rad4* | BRCT domain protein Rad4 | BRCT domain protein Rad4 | 1.58 |
| SPAC4F10.17 | | histone promoter control protein Hpc2 (predicted) | | 1.58 |
| SPAC20H4.02 | *mek1* | Cds1/Rad53/Chk2 family protein kinase Mek1 | Cds1/Rad53/Chk2 family protein kinase Mek1 (PMID 12482912) | 1.58 |
| SPAC1B3.06c | | B13958 domain | B13958 domain | 1.58 |

| | | | | |
|---|---|---|---|---|
| SPBC649.03 | *gtr2* | Gtr1/RagA G protein Gtr2 | Gtr1/RagA G protein Gtr2 | 1.58 |
| SPAC3A11.05c | | conserved protein (fungal and plant) | | 1.58 |
| SPAC23C4.05c | | nitrogen permease regulator family | nitrogen permease regulator family | 1.58 |
| SPAC186.04c | *spo5* | meiotic RNA-binding protein 1 | meiotic RNA-binding protein 1 | 1.58 |
| SPBC713.08 | *sdh2* | succinate dehydrogenase (ubiquinone) iron-sulfur protein subunit | succinate dehydrogenase (ubiquinone) iron-sulfur protein subunit | 1.57 |
| SPBC1D7.05 | | ski complex interacting GTPase (predicted) | ski complex subunit Ski7 (predicted) | 1.57 |
| SPCC132.04c | *bet1* | SNARE Bet1 | SNARE Bet1 | 1.57 |
| SPBC1778.10c | | nuclease | | 1.57 |
| SPAC630.05 | *dad1* | DASH complex subunit Dad1 | DASH complex subunit Dad1 | 1.57 |
| SPBC660.07 | | membrane bound O-acyltransferase, MBOAT (predicted) | | 1.57 |
| SPAC7D4.09c | *mak1* | histidine kinase Mak1 | histidine kinase Mak1 | 1.57 |
| SPAC688.04c | *nak1* | PAK-related kinase Nak1 | | 1.57 |
| SPCC1672.08c | *srx1* | sulfiredoxin | sulphiredoxin (PMID 15824112) | 1.57 |
| SPBP18G5.02 | | clathrin binding protein | | 1.57 |
| SPAC1039.06 | *cob1* | cytochrome b, Cob1 | cytochrome b; respiratory chain complex III; similar to S. cerevisiae Q0105 | 1.57 |
| SPCC757.07c | | Pex16 family peroxisome import protein | | 1.57 |
| SPAC31G5.15 | *lkh1* | dual specificity protein kinase Lkh1 | dual specificity protein kinase Lkh1 | 1.56 |
| SPAC1486.01 | *dga1* | diacylglycerol O-acyltransferase | | 1.56 |
| SPCC1919.12c | | uracil phosphoribosyltransferase (predicted) | uracil phosphoribosyltransferase (predicted) | 1.56 |
| SPBC1734.08 | | mitochondrial ribosomal protein subunit S37 (predicted) | mitochondrial ribosomal protein subunit S37 (predicted) | 1.56 |
| SPBC31F10.02 | *cyk3* | cytokinesis protein Cyk3 | cytokinesis protein Cyk3 | 1.56 |
| SPAC607.09c | | alpha-glucosidase | alpha-glucosidase | 1.56 |
| SPCC16A11.01 | | mitochondrial Mvp17/PMP22 family protein 2 | Mvp17/PMP22 family | 1.56 |
| SPBC1773.09c | *pso2* | DNA 5' exonuclease (predicted) | DNA 5' exonuclease (predicted) | 1.56 |
| SPAC1952.04c | *ste20* | sterility protein Ste20 | | 1.56 |
| SPAC3A12.08 | *rhb1* | Rheb GTPase Rhb1 | Rheb GTPase Rhb1 | 1.56 |
| SPCC1235.06 | *scd1* | RhoGEF Scd1 | RhoGEF Scd1 | 1.56 |
| SPAC1002.21 | *mde5* | alpha-amylase homolog Mde5 | alpha-amylase homolog Mde5 | 1.56 |
| SPAC22E12.15 | *tsc2* | tuberin | tuberin | 1.55 |
| SPAC17A2.07c | | sodium/calcium exchanger (predicted) | sodium/calcium exchanger (predicted) | 1.55 |

| | | | | |
|---|---|---|---|---|
| SPAP27G11.12 | *aar2* | U5 snRNP-associated protein Aar2 | U5 snRNP-associated protein Aar2 | 1.55 |
| SPCC188.12 | *vps32* | vacuolar sorting protein Vps32 | vacuolar sorting protein Vps32 | 1.55 |
| SPAC12D12.09 | *gwt1* | pig-W | pig-W | 1.55 |
| SPBC1198.01 | *yam8* | calcium transport protein | calcium transport protein | 1.55 |
| SPBC83.12 | *dcp2* | mRNA decapping complex subunit Dcp2 | mRNA decapping complex subunit Dcp2 | 1.55 |
| SPBC1709.09 | *abc3* | ABC transporter Abc3 | ABC transporter Abc3 | 1.55 |
| SPCC191.11 | | pseudogene | pseudogene | 1.55 |
| SPAC1142.05 | | cleavage and polyadenylation specificity factor (predicted) | cleavage and polyadenylation specificity factor (predicted) | 1.55 |
| SPAC105.01c | | WIP homolog | WIP homolog | 1.55 |
| SPAC4F10.07c | *alp4* | gamma tubulin complex Spc97/GCP2 subunit Alp4 | | 1.54 |
| SPCC1840.12 | | dubious | dubious | 1.54 |
| SPCC11E10.02c | *uap2* | U2 snRNP-associated protein Uap2 | | 1.54 |
| SPBC1105.13c | *ssp2* | serine/threonine protein kinase Ssp2 | serine/threonine protein kinase Ssp2 | 1.54 |
| SPAC1782.01 | | DUF1741 family protein | | 1.54 |
| SPBC2G2.17c | | membrane transporter | | 1.54 |
| SPAC56E4.06c | *mug77* | autophagy associated protein Atg5 (predicted) | | 1.54 |
| SPAPB17E12.04c | | tyrosine-tRNA ligase | tyrosine-tRNA ligase | 1.54 |
| SPBC16D10.04c | *ubp22* | ubiquitin C-terminal hydrolase Ubp22 | ubiquitin C-terminal hydrolase Ubp22 | 1.54 |
| SPAC11D3.01c | | hydroxyacid dehydrogenase (predicted) | hydroxyacid dehydrogenase (predicted) | 1.54 |
| SPAC23H3.13c | *cwf24* | zf-C3HC4 type (RING finger)/GCN5-related N acetyltransferase fusion protein | | 1.54 |
| SPCC830.02 | | dubious | dubious | 1.53 |
| SPAC24B11.05 | *dna2* | DNA replication endonuclease-helicase Dna2 | | 1.53 |
| SPCC306.08c | | DUF953 family protein | | 1.53 |
| SPBC23G7.10c | | mitochondrial DNA binding protein (predicted) | | 1.53 |
| SPBC1718.01 | | aconitate hydratase | aconitate hydratase | 1.53 |
| SPBC651.05c | | mitochondrial membrane protein complex assembly protein | | 1.53 |
| SPAC2E1P3.04 | | 2-methylbutyraldehyde reductase (predicted) | 2-methylbutyraldehyde reductase (predicted) | 1.53 |
| SPCC736.05 | | conserved eukaryotic protein | | 1.53 |

| | | | | |
|---|---|---|---|---|
| SPBC1289.02c | | sequence orphan | | 1.53 |
| SPAC1142.07c | | calcipressin | | 1.53 |
| SPCC1450.01c | | urease accessory protein UREG (predicted) | urease accessory protein UREG (predicted) | 1.53 |
| SPCC18.09c | *ndc80* | spindle pole body protein Ndc80 | | 1.53 |
| SPAC15A10.06 | | oxysterol binding protein (predicted) | oxysterol binding protein (predicted) | 1.53 |
| SPAC32A11.01 | *myo52* | myosin type V | myosin type V | 1.52 |
| SPBP8B7.24c | *rad13* | DNA repair nuclease Rad13 | | 1.52 |
| SPBC215.07c | | WD repeat protein, human WDR70 family | WD repeat protein, human WDR70 family | 1.52 |
| SPBC83.05 | *atp5* | F0-ATPase delta subunit | F0-ATPase delta subunit | 1.52 |
| SPAP11E10.02c | | chromosome segregation protein | chromosome segregation protein | 1.52 |
| SPBC29A10.04 | | conjugation protein (predicted) | conserved fungal protein | 1.52 |
| SPAC27D7.08c | *hrr1* | Helicase Required for RNAi-mediated heterochromatin assembly Hrr1 | Helicase Required for RNAi-mediated heterochromatin assembly Hrr1 | 1.52 |
| SPAC6F6.12 | *amt3* | ammonium transporter Amt3 | ammonium transporter Amt3 | 1.52 |
| SPBC6B1.09c | | PWWP domain protein | | 1.52 |
| SPAC22F3.13 | | mitochondrial pyruvate dehydrogenase (lipoamide) kinase | pyruvate dehydrogenase (lipoamide) kinase | 1.51 |
| SPAC1296.03c | | CDC50 domain protein | | 1.51 |
| SPAC31G5.18c | | proteasome component | proteasome component | 1.51 |
| SPBC3B9.17 | *sft2* | Golgi transport protein Sft2 (predicted) | Golgi transport protein Sft2 (predicted) | 1.51 |
| SPAC6F6.16c | | sequence orphan | | 1.51 |
| SPAC1F7.09c | | spindle pole body interacting protein (predicted) | | 1.51 |
| SPBC215.10 | | mitochondrial splicing suppressor (predicted) | mitochondrial splicing suppressor (predicted) | 1.51 |
| SPBC13E7.10c | *gpi17* | pig-S | pig-S | 1.51 |
| SPBC1773.13 | *mes1* | meiosis II protein Mes1 | meiosis II protein Mes1 | 1.51 |
| SPBC713.09 | *snx12* | sorting nexin Snx12 (predicted) | sorting nexin Snx12 (predicted) | 1.51 |
| SPAC17G6.03 | *sec71* | Sec7 domain | Sec7 domain | 1.51 |
| SPCC1223.03c | | metaxin 1 | metaxin 1 | 1.51 |
| SPCC970.09 | | NAD binding dehydrogenase family protein | | 1.51 |
| SPAC23C4.13 | *psm1* | mitotic cohesin complex subunit Psm1 | | 1.51 |
| SPAC688.03c | | ORMDL family protein | | 1.51 |
| SPAC1751.01c | | sequence orphan | | 1.50 |

| | | | | |
|---|---|---|---|---|
| SPMIT.01 | | S. pombe specific DUF999 protein family 5 | S. pombe specific DUF999 protein family 5 | 1.50 |
| SPAC959.09c | *vtc4* | vacuolar transporter chaperone (VTC) complex subunit (predicted) | vacuolar transporter chaperone (VTC) complex subunit (predicted) | 1.50 |
| SPBC1539.02 | | aspartate aminotransferase (predicted) | aspartate aminotransferase (predicted) | 1.50 |
| SPBC1773.02c | *app1* | App1 protein | App1 protein | 1.50 |
| SPCC306.06c | *pho2* | 4-nitrophenylphosphatase | | 1.50 |
| SPCC162.04c | *srb8* | mediator complex subunit Srb8 | mediator complex subunit Srb8 (PMID 12738880) | 1.50 |
| SPBC31E1.01c | *php3* | CCAAT-binding factor complex subunit Php3 | CCAAT-binding factor complex subunit Php3 (PMID 8223474) | 1.50 |
| SPCC1620.14c | | sequence orphan | sequence orphan | 1.50 |
