# Supplementary material for: The Fission Yeast GATA Factor, Gaf1, Modulates Sexual Development via Direct Down-Regulation of ste11+ Expression in Response to Nitrogen Starvation
Source: PLoS One. 2012 Aug 10;7(8):e42409. doi: 10.1371/journal.pone.0042409 (PMC3416868; doi:10.1371/journal.pone.0042409)
Supplement: Table S3 — List of the genes up-regulated in nitrogen-starved (−N) gaf1 Δ cells (Group −N/−G). (PDF) [file pone.0042409.s003.pdf]

Table S3. List of the genes up-regulated in nitrogen-starved (-N) *gaf1* Δ cells (Group -N/-G)

*** 1,418 genes**

**Systematic**	**Gene name**	**Description (GeneDB)**	**Description (FunCat2)**	**Expression Ratio (*gaf1Δ*, -N) / (*gaf1Δ*, +N)**
SPCC1223.02	*nmt1*	no message in thiamine Nmt1	no message in thiamine Nmt1	705.95
SPMIT.09	*atp8*	F0-ATPase subunit 8		148.00
SPAC1F7.06		ThiJ domain protein	ThiJ domain protein	113.60
SPAC6B12.03c		HbrB family protein	HbrB family protein	97.09
SPCC548.07c	*ght1*	hexose transporter Ght1	hexose transporter Ght1 (PMID [illegible]	91.65
SPCC548.08c	*ght2*	hexose transporter Ght2	hexose transporter Ght1 (PMID 10735858)	92.65
SPCC1795.06	*map2*	P-factor	P-factor (PMID 8314086)	66.21
SPAC4H3.03c		glucan 1,4-alpha-glucosidase (predicted)	glucan 1,4-alpha-glucosidase (predicted)	65.44
SPCC191.11	*inv1*	beta-fructofuranosidase	beta-fructofuranosidase	61.31
SPAC1F5.09c	*shk2*	PAK-related kinase Shk2	PAK-related kinase Shk2 (PMID 9660817) (PMID 9660818)	53.30
SPAC3G9.11c		pyruvate decarboxylase (predicted)	pyruvate decarboxylase (predicted)	51.07
SPBC359.06	*mug14*	adducin	adducin	45.98
SPAC2F7.06c	*pol4*	DNA polymerase X family	DNA polymerase X family	45.09
SPAPB24D3.10c	*agl1*	alpha-glucosidase Agl1	alpha-glucosidase Agl1	43.09
SPAC186.04c		pseudogene, similar to N-terminal of transmembrane channel	pseudogene, similar to N-terminal of transmembrane channel	39.34
SPAC869.06c		cation binding protein (predicted)	cation binding protein (predicted)	38.85
SPAC29A4.12c	*mug108*	sequence orphan	sequence orphan	38.79
SPCC1739.08c		short chain dehydrogenase (predicted)	short chain dehydrogenase	38.29
SPCC794.01c		glucose-6-phosphate 1-dehydrogenase (predicted)	glucose-6-phosphate 1-dehydrogenase (predicted)	38.16
SPAC869.07c	*mel1*	alpha-galactosidase	alpha-galactosidase (PMID 15580593)	37.51
SPAPB8E5.05	*mfm1*	M-factor precursor Mfm1	M-factor precursor Mfm1	37.23
SPCC548.03c	*wtf4*	wtf element Wtf4, pseudo	wtf element Wtf4, pseudo	34.45
SPBC16E9.16c	*lsd90*	Lsd90 protein		33.93
SPBP4H10.09	*rsv1*	transcription factor Rsv1 (predicted)		33.11
SPBP4H10.10		rhomboid family protease		31.83

SPBC21D10.06c	*map4*	cell agglutination protein Map4		30.89
SPAC4F8.08	*mug114*	sequence orphan	sequence orphan	30.11
SPCC162.10	*ppk33*	serine/threonine protein kinase Ppk33 (predicted)	serine/threonine protein kinase Ppk33 (predicted)	30.03
SPAC1F8.05	*isp3*	sequence orphan	sequence orphan	26.64
SPAC1F8.01	*ght3*	hexose transporter Ght3	hexose transporter Ght3 (PMID 10735857)	25.29
SPBC1198.14c	*fbp1*	fructose-1,6-bisphosphatase Fbp1		24.82
SPAC31G5.09c	*spk1*	MAP kinase Spk1	MAP kinase Spk1	23.90
SPAC1002.19	*urg1*	GTP cyclohydrolase II (predicted)	GTP cyclohydrolase (predicted)	23.25
SPAC13F5.03c		mitochondrial iron-containing alcohol dehydrogenase family protein	glycerol dehydrogenase (Phlippen, Stevens, Wolf,Zimmermann manuscript in [illegible]	22.05
SPAC23H3.15c		sequence orphan		21.53
SPBC1683.08	*ght4*	hexose transporter Ght4	hexose transporter Ght4 (PMID	20.06
SPBC56F2.06	*mug147*	sequence orphan		19.90
SPAC4H3.04c		UPF0103 family	UPF0103 family	19.52
SPBC146.02		sequence orphan		19.49
SPAPJ691.02		yippee-like protein	yippee-like protein	18.77
SPAC22F3.12c	*rgs1*	regulator of G-protein signaling Rgs1	regulator of G-protein signaling Rgs1	17.46
SPCC794.04c		membrane transporter	membrane transporter	17.07
SPAC513.03	*mfm2*	M-factor precursor Mfm2	M-factor precursor Mfm2	16.93
SPCC1442.01	*ste6*	guanyl-nucleotide exchange factor Ste6	guanyl-nucleotide exchange factor Ste6	15.76
SPAC1093.06c	*dhc1*	dynein heavy chain		15.66
SPAC23E2.03c	*ste7*	meiotic suppressor protein Ste7	meiotic suppressor protein Ste7	15.28
SPAC1A6.06c	*meu31*	sequence orphan	sequence orphan	14.15
SPAC1F8.04c		hydrolase (predicted)	hydrolase (predicted)	13.74
SPCPB16A4.06c		sequence orphan	sequence orphan	13.20
SPBC1289.16c		copper amine oxidase (predicted)		13.15
SPCC338.18		sequence orphan	sequence orphan	12.11
SPAC1751.01c	*gti1*	gluconate transporter inducer Gti1	gluconate transporter inducer Gti1	12.06
SPAC17A2.01	*bsu1*	high-affinity import carrier for pyridoxine, pyridoxal, and pyridoxamine Bsu1	high-affinity import carrier for pyridoxine,pyridoxal, and pyridoxamine Bsu1	12.05
SPAPJ695.01c		S. pombe specific UPF0321 family protein 3	S. pombe specific UPF0321 family protein 3	11.10
SPBC660.05		conserved fungal protein	conserved fungal protein	11.09
SPAC22G7.11c		conserved fungal protein	conserved fungal protein	10.52
SPCC1020.01c	*pma2*	P-type proton ATPase Pma2		10.26

SPAC688.03c		human AMMECR1 homolog	human AMMECR1 homolog	10.25
SPCC757.03c		ThiJ domain protein	ThiJ domain protein	10.23
SPBC1348.14c	*ght7*	hexose transporter Ght7	hexose transporter Ght7	10.19
SPAC15A10.05c	*mug182*	YjeF family protein	YjeF family protein	10.16
SPAC458.04c		sequence orphan	sequence orphan	9.91
SPAC20H4.11c	*rho5*	Rho family GTPase Rho5	Rho family GTPase Rho5	9.76
SPCC1223.09		uricase (predicted)	uricase (predicted)	9.67
SPBPB2B2.10c		galactose-1-phosphate uridylyltransferase (predicted)		9.67
SPAC27D7.03c	*mei2*	RNA-binding protein involved in meiosis Mei2	RNA-binding protein involved in meiosis Mei2	9.30
SPBC725.10		tspO homolog		9.22
SPCC70.04c		sequence orphan	sequence orphan	9.02
SPAC513.04		sequence orphan	sequence orphan	8.96
SPAC20G4.02c	*fus1*	formin Fus1	formin Fus1	8.79
SPAC20G4.03c	*hri1*	eIF2 alpha kinase Hri1	eIF2 alpha kinase Hri1	8.74
SPAC15E1.02c		DUF1761 family protein	DUF1761 family protein	8.63
SPBPB2B2.06c		phosphoprotein phosphatase (predicted)		8.61
SPAC14C4.01c		DUF1770 family protein	DUF1770 family protein	8.58
SPAC167.06c	*mug143*	sequence orphan	sequence orphan	8.53
SPAC27D7.13c	*ssm4*	p150-Glued		8.45
SPAC977.16c	*dak2*	dihydroxyacetone kinase Dak2	dihydroxyacetone kinase Dak2 (PMID 9804990)	8.29
SPCC1393.12		sequence orphan	sequence orphan	8.24
SPCP31B10.06	*mug190*	C2 domain protein	C2 domain protein Tcb3 (predicted)	8.07
SPBC1604.01	*mug158*	sulfatase modifying factor 1 related		7.76
SPCC162.02c		AMP-binding dehydrogenase (predicted)	AMP-binding dehydrogenase (predicted)	7.72
SPAC139.05		succinate-semialdehyde dehydrogenase (predicted)	succinate-semialdehyde dehydrogenase (predicted)	7.67
SPAC5D6.09c	*mug86*	acetate transporter (predicted)	acetate transporter (predicted)	7.61
SPAC977.17		MIP water channel (predicted)	MIP water channel	7.59
SPAC22F8.05		alpha,alpha-trehalose-phosphate synthase (predicted)	alpha,alpha-trehalose-phosphate synthase (predicted)	7.48
SPCC191.01		sequence orphan	sequence orphan	7.39
SPBC24C6.06	*gpa1*	G-protein alpha subunit		7.32
SPAC3F10.10c	*map3*	pheromone M-factor receptor	pheromone M-factor receptor (PMID 8380233)	7.13

SPCC162.03		short chain dehydrogenase (predicted)	short chain dehydrogenase (predicted)	7.05
SPAC589.07c		WD repeat protein Atg18	WD repeat protein Atg18	7.00
SPAC22H10.13	*zym1*	metallothionein	metallothionein (PMID 12050156)	6.96
SPAC1565.03		sequence orphan	sequence orphan	6.94
SPAC3C7.13c		glucose-6-phosphate 1-dehydrogenase (predicted)	glucose-6-phosphate 1-dehydrogenase (predicted)	6.90
SPBC19C2.05	*ran1*	serine/threonine protein kinase Ran1		6.88
SPCC191.10		sequence orphan	sequence orphan	6.79
SPCC1020.05		phosphoprotein phosphatase (predicted)	phosphoprotein phosphatase (predicted)	6.74
SPAC1399.01c		membrane transporter (predicted)	purine permease (predicted)	6.74
SPBC29A10.14	*rec8*	meiotic cohesin complex subunit Rec8		6.74
SPBC3H7.08c		conserved fungal protein		6.49
SPBC32H8.07	*git5*	heterotrimeric G protein beta subunit Git5		6.45
SPBC1271.05c		zinc finger protein zf-AN1 type	zinc finger protein zf-AN1 type	6.24
SPAC11E3.06	*map1*	MADS-box transcription factor Map1	MADS-box transcription factor Map1	6.14
SPBPB7E8.02		PSP1 family protein		6.08
SPAP7G5.03		conjugation protein (predicted)	conjugation protein (predicted)	6.03
SPBPB21E7.06		pseudogene	pseudogene	6.00
SPBC215.11c		aldo/keto reductase, unknown biological role		5.99
SPCC4G3.03		WD repeat protein	WD repeat protein	5.92
SPAC4F10.17		conserved fungal protein	conserved fungal protein	5.84
SPAC23H3.04		conserved fungal protein	conserved fungal protein	5.82
SPAC637.03		conserved fungal protein	conserved fungal protein	5.78
SPBC24C6.09c		phosphoketolase (predicted)		5.69
SPCC24B10.14c	*xlf1*	xrcc4 like factor	xrcc4 like factor	5.69
SPBPB2B2.12c		UDP-glucose 4-epimerase		5.67
SPAC5H10.11	*gmh1*	alpha-1,2-galactosyltransferase Gmh1 (predicted)	alpha-1,2-galactosyltransferase Gmh1	5.64
SPCC1393.07c	*mug4*	sequence orphan	sequence orphan	5.57
SPAC1952.15c	*rec24*	meiotic recombination protein Rec24	meiotic recombination protein Rec24	5.57
SPMIT.04	*cox3*	cytochrome c oxidase 3	cytochrome c oxidase 3; similar to S. cerevisiae Q0275	5.52
SPAC30D11.01c		alpha-glucosidase		5.48
SPBC577.05c	*rec27*	meiotic recombination protein Rec27		5.42
SPAC1565.04c	*ste4*	adaptor protein Ste4	adaptor protein Ste4	5.41

SPAC1F7.09c		allantoicase (predicted)	allantoicase (predicted)	5.39
SPBC1685.05		serine protease (predicted)	serine protease (predicted)	5.35
SPAC3G6.07		dubious	sequence orphan	5.31
SPBC365.12c	*ish1*	LEA domain protein		5.30
SPBC725.03		conserved fungal protein		5.29
SPAC869.09		conserved fungal protein	conserved fungal protein	5.26
SPAC15F9.01c		sequence orphan	sequence orphan	5.22
SPAC27F1.05c		aminotransferase class-III, unknown specificty	4-aminobutyrate transaminase	5.19
SPAC19D5.01	*pyp2*	tyrosine phosphatase Pyp2	tyrosine phosphatase Pyp2	5.15
SPBC839.06	*cta3*	P-type ATPase, calcium transporting Cta3	P-type ATPase, calcium transporting Cta3	5.13
SPCC895.08c		conserved fungal protein	conserved fungal protein	5.06
SPAC17A2.15		dubious	dubious	4.99
SPBC1773.05c	*tms1*	hexitol dehydrogenase (predicted)	hexitol dehydrogenase (predicted)	4.93
SPCC74.09	*mug24*	RNA-binding protein, rrm type	RNA-binding protein	4.90
SPCC1223.03c	*gut2*	glycerol-3-phosphate dehydrogenase Gut2	glycerol-3-phosphate dehydrogenase Gut2	4.90
SPAC4D7.02c		glycerophosphoryl diester phosphodiesterase (predicted)	glycerophosphoryl diester phosphodiesterase (predicted)	4.89
SPAC29E6.07		sequence orphan	sequence orphan	4.86
SPCC285.07c	*wtf18*	wtf element Wtf18	wtf element Wtf18	4.83
SPBC106.13		conserved eukaryotic protein	conserved eukaryotic protein	4.83
SPCC1235.13	*ght6*	hexose transporter Ght6	hexose transporter Ght6 (PMID 10735857)	4.81
SPAC1805.15c	*pub2*	ubiquitin-protein ligase Pub2	ubiquitin-protein ligase Pub2	4.73
SPBC1711.11		autophagy associated protein (predicted)		4.72
SPCC830.02	*wtf24*	wtf element Wtf24		4.70
SPBC16E9.17c	*rem1*	meiosis-specific cyclin Rem1		4.69
SPAC3C7.05c	*mug191*	alpha-1,6-mannanase (predicted)	alpha-1,6- mannanase (predicted)	4.67
SPBC29A10.02	*spo5*	meiotic RNA-binding protein 1	meiotic RNA-binding protein 1	4.66
SPBC1347.01c	*rev1*	deoxycytidyl transferase Rev1 (predicted)	deoxycytidyl transferase Rev1 (predicted)	4.65
SPAC17H9.19c	*cdt2*	WD repeat protein Cdt2	WD repeat protein Cdt2	4.63
SPAC869.08	*pcm2*	protein-L-isoaspartate O-methyltransferase (predicted)	protein-L-isoaspartate O-methyltransferase (predicted)	4.62
SPBC6B1.02	*ppk30*	Ark1/Prk1 family protein kinase Ppk30		4.61

SPAC6G10.06		FAD-dependent amino acid oxidase (predicted)	amino acid oxidase (predicted)	4.60
SPAC16E8.03	*gna1*	glucosamine-phosphate N-acetyltransferase	glucosamine-phosphate N-acetyltransferase	4.59
SPAC23C11.06c		hydrolase (inferred from context)	hydrolase (inferred from context)	4.57
SPAC2C4.17c		MS ion channel protein 2	MS ion channel protein 2	4.57
SPBPB2B2.13		galactokinase Gal1 (predicted)		4.54
SPAC4A8.04	*isp6*	vacuolar serine protease Isp6	vacuolar serine protease Isp6	4.51
SPBC23G7.11		DNA-3-methyladenine glycosidase Mag2 (predicted)		4.51
SPCPB1C11.02		amino acid permease, unknown 16	amino acid permease, unknown 16	4.50
SPAC1002.20		sequence orphan	sequence orphan	4.48
SPAC31G5.07		conjugation protein (predicted)	conserved fungal protein	4.45
SPAC18G6.01c		conserved fungal protein	conserved fungal protein	4.45
SPBC530.07c		phosphomethylpyrimidine kinase (predicted)		4.45
SPCC1450.08c	*wtf16*	wtf element Wtf16	wtf element Wtf16	4.43
SPCC794.02	*wtf5*	wtf element Wtf5	wtf element Wtf5	4.42
SPAC57A7.05		conserved protein (fungal and plant)	conserved protein (fungal and plant)	4.42
SPAC2G11.13	*atg22*	autophagy associated protein Atg22 (predicted)	autophagy associated protein Atg22 (predicted)	4.38
SPAC19B12.08		peptidase family C54	peptidase family C54	4.38
SPCC576.01c		sulfonate dioxygenase (predicted)	sulfonate dioxygenase (predicted)	4.37
SPAC212.08c		GPI anchored protein (predicted)	GPI anchored protein (predicted)	4.31
SPAC9E9.17c		dubious	dubiuos	4.30
SPCC1739.15	*wtf21*	wtf element Wtf21	wtf element Wtf21	4.28
SPBC1718.02	*hop1*	linear element associated protein Hop1		4.26
SPAC32A11.01	*mug8*	conserved fungal protein	conserved fungal protein	4.25
SPCC1529.01		membrane transporter		4.25
SPAC15E1.10		PI31 proteasome regulator related		4.21
SPAC1002.05c	*jmj2*	histone demethylase Jmj2 (predicted)	histone demethylase Jmj2 (predicted)	4.18
SPAPB1A10.14		F-box protein	F-box protein, unnamed	4.17
SPBC1348.13		pseudogene	pseudogene	4.17
SPBC106.10	*pka1*	cAMP-dependent protein kinase catalytic subunit Pka1	cAMP-dependent protein kinase catalytic subunit Pka1 (PMID 8144551)	4.13
SPCC285.06c	*wtf17*	wtf element Wtf17, pseudo	wtf element Wtf17, pseudo	4.12
SPBC15C4.06c		ubiquitin-protein ligase E3 (predicted)		4.12
SPAC11E3.14		conserved protein	conserved protein	4.10

SPAC57A7.09		human RNF family homolog	human RNF family homolog	4.10
SPBC18H10.05		WD repeat protein, human WDR44 family		4.08
SPMIT.06		mitochondrial DNA binding endonuclease		4.08
SPBC354.08c		DUF221 family protein	DUF221 family protein	4.07
SPBC1105.14	*rsv2*	transcription factor Rsv2		4.06
SPAC3A11.06	*mvp1*	sorting nexin Mvp1	sorting nexin Mvp1	4.05
SPCC1322.08	*srk1*	MAPK-activated protein kinase Srk1	MAPK-activated protein kinase Srk1	4.03
SPBC1773.02c		thioredoxin peroxidase	thioredoxin peroxidase	4.02
SPCC417.06c	*ppk35*	serine/threonine protein kinase Ppk35	serine/threonine protein kinase Ppk35	4.00
SPAC8C9.16c	*mug63*	TLDc domain protein 1	TLDc domain protein 1	3.97
SPAC2F3.08	*sut1*	alpha-glucoside transporter	alpha-glucoside transporter (PMID 11136464)	3.95
SPCC338.12		subtilisin related protein (predicted)	protease inhibitor (predicted)	3.95
SPAC15A10.03c	*rhp54*	Rad54 homolog Rhp54	Rad54 homolog Rhp54	3.94
SPCC622.11		LMBR1-like membrane protein	LMBR1-like membrane protein	3.93
SPAC6B12.08	*mug185*	DNAJ domain protein Jjj family	DNAJ domain protein Jjj family	3.93
SPAC16E8.02		DUF962 family protein	conserved protein (broad species distribution)	3.92
SPCC4B3.01		thiosulfate sulfurtransferase		3.89
SPCC1223.12c	*meu10*	GPI anchored cell surface protein (predicted)	conserved fungal family	3.86
SPBC216.03		conserved fungal protein		3.86
SPCC16A11.08	*atg20*	sorting nexin Atg20	sorting nexin Atg20	3.85
SPCC1281.08	*wtf11*	wtf element Wtf11	wtf element Wtf11	3.85
SPCC320.03		transcription factor (predicted)	transcription factor (predicted)	3.85
SPCC285.10c		SPRY domain protein	SPRY domain protein	3.84
SPCC162.06c		vacuolar sorting protein Vps60	vacuolar sorting protein Vps60	3.84
SPBC19C2.04c	*ubp11*	ubiquitin C-terminal hydrolase Ubp11	ubiquitin C-terminal hydrolase Ubp11	3.84
SPAC5D6.10c	*mug116*	sequence orphan	sequence orphan	3.83
SPBC800.14c		DUF1772 family protein	DUF1772 family protein	3.82
SPBC36.11		sequence orphan		3.82
SPBC19F5.01c	*puc1*	cyclin Puc1		3.80
SPAC2E12.03c		G-protein coupled receptor (predicted)	G-protein coupled receptor (predicted)	3.79
SPBC713.06	*adl1*	DNA ligase (predicted)	DNA ligase (predicted)	3.79
SPMIT.01	*cox1*	cytochrome c oxidase 1	cytochrome c oxidase 1; similar to S. cerevisiae Q0045	3.78

SPCC285.11	*ucp10*	UBA/UAS domain protein Ucp10	UBA/UAS domain protein Ucp10	3.78
SPBC23G7.10c		NADH-dependent flavin oxidoreductase (predicted)		3.77
SPAC750.07c		S. pombe specific GPI anchored protein family 1	S. pombe specific GPI anchored protein family 1	3.77
SPAPB1E7.08c		membrane transporter	membrane transporter	3.77
SPAC1039.09	*isp5*	amino acid permease Isp5	amino acid permease Isp5	3.77
SPCC16A11.15c		sequence orphan	sequence orphan	3.76
SPBC685.03		sequence orphan		3.76
SPBC32H8.02c	*nep2*	NEDD8 protease Nep2		3.74
SPBC776.05		membrane transporter (predicted)		3.72
SPBC19C2.09	*sre1*	sterol regulatory element binding protein Sre1		3.72
SPBP4H10.12		conserved protein (fungal and bacterial)		3.70
SPAC1F7.12	*yak3*	aldose reductase YakC		3.69
SPAC29A4.17c		FUN14 family protein	FUN14 family protein	3.68
SPAC29B12.13		carbon-sulfur lyase (predicted)	carbon-sulfur lyase (predicted)	3.67
SPAC1782.12c		DUF423 protein	conserved protein (broad species distribution)	3.67
SPCC285.04		transthyretin (predicted)	transthyretin (predicted)	3.66
SPBC16A3.02c		mitochondrial peptidase (predicted)		3.66
SPCC663.17	*wtf15*	wtf element Wtf15, pseudo	wtf element Wtf15, pseudo	3.64
SPAC19B12.10	*sst2*	human AMSH protein homolog	human amsh protein homolog	3.60
SPBC23G7.06c		conserved eukaryotic protein		3.60
SPAPB2B4.06		conserved fungal protein	conserved fungal protein	3.58
SPBP8B7.18c		phosphomethylpyrimidine kinase (predicted)		3.57
SPBC1D7.02c	*scr1*	transcription factor Scr1		3.56
SPAC22G7.08	*ppk8*	serine/threonine protein kinase Ppk8 (predicted)	serine/threonine protein kinase Ppk8 (predicted)	3.56
SPBC32H8.06	*mug93*	TPR repeat protein, meiotically spliced		3.56
SPAC4G9.12		gluconokinase	gluconokinase	3.56
SPAPB1A11.03		FMN dependent dehydrogenase	FMN dependent dehydrogenase	3.54
SPBC21B10.12	*rec6*	meiotic recombination protein Rec6		3.53
SPAC4H3.08		short chain dehydrogenase (predicted)	short chain dehydrogenase (predicted)	3.53
SPBPB2B2.11		nucleotide-sugar 4,6-dehydratase (predicted)		3.52
SPCC622.03c		dubious	dubious	3.51

SPCC1906.04	*wtf20*	wtf element Wtf20	wtf element Wtf20	3.51
SPAC19G12.09		NADH/NADPH dependent indole-3-acetaldehyde reductase AKR3C2	NADH/NADPH dependent indole-3-acetaldehyde reductase AKR3C2	3.49
SPAC25B8.13c	*isp7*	2-OG-Fe(II) oxygenase superfamily protein	2-OG-Fe(II) oxygenase superfamily protein	3.47
SPAC2G11.05c		BRO1 domain protein	BRO1 domain protein	3.47
SPBC8E4.05c		fumarate lyase superfamily		3.46
SPAC823.16c	*mug179*	WD repeat protein Mug179	WD repeat protein Mug179	3.46
SPBC1718.01	*pop1*	F-box/WD repeat protein Pop1		3.42
SPBC1271.01c	*pof13*	F-box protein Pof13	F-box protein Pof13	3.42
SPAPB8E5.10		sequence orphan	sequence orphan	3.42
SPAC513.02		phosphoglycerate mutase family	phosphoglycerate mutase family	3.41
SPCC1020.10	*oca2*	serine/threonine protein kinase Oca2 (predicted)	serine/threonine protein kinase Oca2 (predicted)	3.40
SPAC227.15		protein phosphatase regulatory subunit Reg1 (predicted)	protein phosphatase regulatory subunit Reg1 (predicted)	3.40
SPAC6C3.07	*mug68*	sequence orphan	sequence orphan	3.39
SPAC13G7.13c	*msa1*	RNA-binding protein Msa1	RNA-binding protein Msa1	3.39
SPBC3H7.06c	*pof9*	F-box protein Pof9		3.38
SPBP8B7.30c	*thi5*	transcription factor Thi5	transcription factor Thi5	3.37
SPCC1682.11c		DUF580 family protein	DUF580 family protein	3.36
SPBC25B2.08		sequence orphan		3.36
SPBC2D10.06	*rep1*	MBF transcription factor complex subunit Rep1		3.36
SPAPB18E9.04c		sequence orphan	sequence orphan	3.36
SPAC25B8.08		conserved fungal family	conserved fungal family	3.35
SPAC6G9.16c		sequence orphan	sequence orphan	3.34
SPAC144.13c	*srw1*	CDK inhibitor Srw1	CDK inhibitor Srw1	3.33
SPAC8C9.03	*cgs1*	cAMP-dependent protein kinase regulatory subunit Cgs1	cAMP-dependent protein kinase regulatory subunit Cgs1	3.31
SPBC1685.14c		Vid27 family protein	Vid27 family protein	3.31
SPAC23D3.05c		alcohol dehydrogenase pseudogene	alcohol dehydrogenase pseudogene	3.30
SPCC548.02c	*wtf3*	wtf element Wtf3	wtf element Wtf3	3.30
SPAC18G6.09c		sequence orphan	sequence orphan	3.30
SPAC4G8.12c		alpha-1,2-mannosyltransferase (predicted)	alpha-1,2-mannosyltransferase (predicted)	3.30
SPAC26F1.01	*sec74*	guanyl-nucleotide exchange factor Sec74		3.28
SPAC12B10.13		CTLH domain	CTLH domain	3.27

SPAC26F1.14c	*aif1*	apoptosis-inducing factor homolog Aif1	apoptosis-inducing factor homolog Aif1	3.26
SPBC1348.12		zinc finger protein		3.26
SPBC1703.12	*ubp9*	ubiquitin C-terminal hydrolase Ubp9	ubiquitin C-terminal hydrolase Ubp9	3.26
SPBC428.10		sequence orphan	sequence orphan	3.25
SPAC821.04c	*cid13*	poly(A) polymerase Cid13	poly(A) polymerase Cid13 (PMID 12062100)	3.25
SPAC19A8.05c	*sst4*	sorting receptor for ubiquitinated membrane proteins, ESCRT 0 complex subunit Sst4	sorting receptor for ubiquitinated membrane proteins (ISS) (PMID 12055639)	3.25
SPAC607.08c		DUF726 family protein	DUF726 family protein	3.25
SPAC26H5.04		vacuolar import and degradation protein Vid28	vacuolar import and degradation protein Vid28	3.25
SPCC18B5.03	*wee1*	dual specificity protein kinase Wee1	dual specificity protein kinase Wee1	3.24
SPBC21.07c	*ppk24*	serine/threonine protein kinase Ppk24		3.23
SPAC4G8.04		GTPase activating protein (predicted)	GTPase activating protein (predicted)	3.23
SPCC622.04		dubious	dubious	3.23
SPBC25B2.02c	*mam1*	M-factor transporter Mam1		3.22
SPAC32A11.02c		conserved fungal protein	conserved fungal protein	3.21
SPAC513.05	*ams1*	alpha-mannosidase (predicted)	alpha-mannosidase (predicted)	3.21
SPAC20G4.05c		UPF0061 family protein	UPF0061 family protein	3.20
SPCC63.08c	*ppk36*	serine/threonine protein kinase Ppk36	serine/threonine protein kinase Ppk36	3.19
SPBC577.13	*syj2*	inositol-polyphosphate 5-phosphatase (synaptojanin homolog 2)		3.19
SPCC594.06c		SNARE Vam7 (predicted)	SNARE Vam7 (predicted)	3.18
SPBC23E6.09	*ssn6*	transcriptional corepressor Ssn6		3.18
SPBC14C8.05c	*meu17*	glucan-alpha-1,4-glucosidase		3.18
SPAC1002.17c	*urg2*	uracil phosphoribosyltransferase (predicted)	uracil phosphoribosyltransferase (predicted)	3.18
SPAC3A11.10c		dipeptidyl aminopeptidase (predicted)	dipeptidyl aminopeptidase (predicted)	3.17
SPBC839.02		arrestin Aly1 related	arrestin Aly1 related	3.16
SPBC119.07	*ppk19*	serine/threonine protein kinase Ppk19		3.15
SPBC31F10.10c		zf-MYND type zinc finger protein		3.14
SPBP8B7.04	*mug45*	sequence orphan		3.14
SPAC23D3.11	*ayr1*	1-acyldihydroxyacetone phosphate reductase (predicted)	1-acyldihydroxyacetone phosphate reductase (predicted)	3.14
SPCC1235.14	*ght5*	hexose transporter Ght5	hexose transporter Ght5 (PMID 10735857)	3.14

SPAC2F3.05c		xylose and arabinose reductase (predicted)	xylose and arabinose reductase (predicted)	3.13
SPMIT.05	*cob1*	cytochrome b, Cob1	cytochrome b; respiratory chain complex III; similar to S. cerevisiae Q0105	3.13
SPAC2E1P3.05c		fungal cellulose binding domain protein	fungal cellulose binding domain protein	3.13
SPBC3B8.10c		NLI interacting factor family		3.13
SPAC4F10.07c	*atg13*	autophagy associated protein Atg13 (predicted)	autophagy associated protein Atg13 (predicted)	3.13
SPCC1450.16c		triacylglycerol lipase	triacylglycerol lipase	3.12
SPBC14C8.01c	*cut2*	securin		3.12
SPAC6G10.03c		abhydrolase family protein, unknown biological role	abhydrolase family protein, unknown biological role	3.11
SPAC13F5.07c		term=sequence orphan; date=20080121	previusly annotated as dubious, may not be protein coding	3.10
SPBC32C12.02	*ste11*	transcription factor Ste11		3.10
SPCC364.05	*vps3*	GTPase regulator Vps3 (predicted)	GTPase regulator Vps3 (predicted)	3.09
SPAC1D4.11c	*lkh1*	dual specificity protein kinase Lkh1	dual specificity protein kinase Lkh1	3.09
SPAC25H1.03	*mug66*	meiotically upregulated gene Mug66	meiotically upregulated gene Mug66	3.08
SPBP19A11.02c		sequence orphan		3.08
SPAC3A11.05c	*kms1*	meiotic spindle pole body protein Kms1	meiotic spindle pole body protein Kms1	3.08
SPBC1348.09		short chain dehydrogenase (predicted)		3.08
SPBC36B7.05c		phosphatidylinositol(3)-phosphate binding protein (predicted)		3.07
SPAC2E12.05	*wtf1*	wtf element Wtf1, pseudo		3.07
SPAC13C5.04		amidotransferase (predicted)	glutamine amidotransferase (predicted)	3.07
SPAC1786.04		sequence orphan	sequence orphan	3.06
SPBC19C7.10		transcription factor (predicted)		3.06
SPCPJ732.02c		xylulose kinase (predicted)	xylulose kinase (predicted)	3.06
SPBC609.03		WD repeat protein, human IQWD1 family		3.06
SPAC27F1.10		sequence orphan	sequence orphan	3.05
SPBC9B6.03		zf-FYVE type zinc finger protein		3.05
SPAC2F3.07c		sequence orphan	sequence orphan	3.05
SPAC688.04c	*gst3*	glutathione S-transferase Gst3	glutathione S-transferase (PMID 12151111)	3.04
SPBC409.12c	*stn1*	telomere cap complex subunit Stn1		3.04

SPAC23A1.14c			uncharacterised trans-sulfuration enzyme (predicted)	cystathionine gamma-synthase (predicted)	3.04
SPCC576.04			bax inhibitor-like protein	bax inhibitor-like protein	3.04
SPAC1556.01c	*rad50*		DNA repair protein Rad50	DNA repair protein Rad50	3.04
SPBC4C3.12		*39692*	fork head transcription factor Sep1		3.04
SPBC725.11c	*php2*		CCAAT-binding factor complex subunit Php2		3.03
SPBC18A7.01			X-Pro dipeptidase (predicted)		3.03
SPAC13C5.03	*tht1*		nuclear membrane protein involved in karyogamy	nuclear membrane protein involved in karyogamy (PMID 9442101)	3.01
SPBC2D10.17	*clr1*		cryptic loci regulator Clr1		3.01
SPCC794.03			amino acid permease, unknown 13	amino acid permease, unknown 13	3.00
SPAC23C4.07	*tht2*		meiotically upregulated gene Mug22	meiotically upregulated gene Mug22	3.00
SPBP19A11.07c			human down-regulated in multiple cancers-1 homolog 2		2.99
SPBC6B1.09c	*nbs1*		Mre11 complex subunit Nbs1		2.99
SPCC1450.13c			riboflavin synthase	riboflavin synthase (PMID 14690539)	2.99
SPBC15D4.02			transcription factor, zf-fungal binuclear cluster type (predicted)		2.98
SPBC20F10.03			conserved eukaryotic protein		2.97
SPAC29B12.03	*spd1*		ribonucleotide reductase (RNR) inhibitor	ribonucleotide reductase (RNR) inhibitor	2.97
SPAC13A11.04c	*ubp8*		ubiquitin C-terminal hydrolase Ubp8 (predicted)	ubiquitin C-terminal hydrolase Ubp8	2.96
SPAC1786.01c			triacylglycerol lipase	triacylglycerol lipase	2.96
SPBPB21E7.08			pseudogene	pseudogene	2.96
SPAC1687.22c	*puf3*		RNA-binding protein Puf3 (predicted)		2.95
SPAC25A8.02			sequence orphan	sequence orphan	2.95
SPAC24B11.05			pyrimidine 5'-nucleotidase (predicted)	pyrimidine 5'-nucleotidase (predicted)	2.95
SPCC970.11c	*wtf9*		wtf element, Wtf2, pseudo	wtf element, Wtf2, pseudo	2.94
SPBC1711.17	*prp16*		ATP-dependent RNA helicase Prp16		2.94
SPBC27B12.04c			conserved eukaryotic protein		2.94
SPCC576.16c	*wtf22*		wtf element Wtf22		2.94
SPCC4G3.12c			ubiquitin-protein ligase E3 (predicted)	ubiquitin-protein ligase E3 (predicted)	2.93
SPBC1685.13			non classical export pathway protein (predicted)	non classical export pathway protein (predicted)	2.93
SPBC29A10.09c			CAF1 family ribonuclease		2.93

SPAC630.05	*gyp7*	GTPase activating protein Gyp7 (predicted)	GTPase activating protein Gyp7 (predicted)	2.92
SPAPB24D3.04c	*mag1*	DNA-3-methyladenine glycosylase Mag1	DNA-3-methyladenine glycosylase Mag1	2.92
SPBC32F12.03c	*gpx1*	glutathione peroxidase Gpx1		2.92
SPAC12D12.09				2.92
SPAC1006.09	*win1*	MAP kinase kinase kinase Win1		2.92
SPAC27D7.11c		But2 family protein	S. pombe specific But2 family protein	2.92
SPAC212.07c		pseudogene	pseudogene	2.91
SPAC18G6.12c		hypothetical protein	hypothetical protein	2.90
SPBC2D10.05	*exg3*	glucan 1,3-beta-glucosidase Exg3		2.90
SPBC887.16		dubious		2.89
SPAC5D6.07c	*pxa1*	PXA domain protein Pxa1	PXA domain protein	2.89
SPBC19C7.03	*cyr1*	adenylate cyclase		2.89
SPBC32H8.11	*mei4*	meiotic forkhead transcription factor Mei4		2.88
SPBPB8B6.02c		urea transporter (predicted)	urea transporter (predicted)	2.88
SPAC4G9.19		DNAJ domain protein DNAJB family	DNAJ domain protein DNAJB family	2.88
SPAC4F10.16c		P-type ATPase	P-type ATPase (PMID 12707717)	2.87
SPCC188.12	*spn6*	septin Spn6	septin Spn6	2.87
SPBC17D11.08		WD repeat protein, human WDR68 family		2.87
SPBC215.10		haloacid dehalogenase-like hydrolase		2.87
SPCC736.13		short chain dehydrogenase (predicted)	short chain dehydrogenase	2.86
SPAC29B12.11c		human WW domain binding protein-2 ortholog	human WW domain binding protein-2 ortholog	2.86
SPAC17A2.09c	*csx1*	RNA-binding protein Csx1	RNA-binding protein Csx1	2.84
SPAC29A4.11	*rga3*	GTPase activating protein Rga3	GTPase activating protein Rga3	2.83
SPAC1250.02	*mug95*	sequence orphan	sequence orphan	2.83
SPAC5D6.06c		UDP-GlcNAc transferase associated protein Alg14	UDP-GlcNAc transferase associated protein Alg14	2.82
SPCC1902.01	*gaf1*	transcription factor Gaf1		2.82
SPAC1952.13	*ned1*	lipin Ned1	lipin	2.81
SPCC1223.10c	*eaf1*	RNA polymerase II transcription elongation factor SpEAF	RNA polymerase II transcription elongation factor SpEAF	2.81
SPAC4G9.07	*mug133*	S. pombe specific UPF0300 family protein 2	S. pombe specific UPF0300 family protein 2	2.80
SPBC21.02		TLDc domain protein 2		2.79
SPBC1271.08c		sequence orphan	sequence orphan	2.79

SPBC19F8.06c	*meu22*	amino acid permease, unknown 11		2.78
SPCC584.02	*cuf2*	Cu metalloregulatory transcription factor Cuf2	Cu metalloregulatory transcription factor Cuf2	2.78
SPMIT.08		mitochondrial ribosomal small subunit		2.78
SPBC32F12.09	*rum1*	CDK inhibitor Rum1		2.77
SPBC887.19	*rft1*	Man5GlcNac2-PP-Dol translocation protein Rft1		2.77
SPAC11H11.01	*sst6*	ESCRT I complex subunit Vps23	ESCRT I complex subunit Vps23	2.77
SPBC6B1.03c		Pal1 family protein		2.77
SPBC1198.13c	*tfg2*	transcription factor TFIIF complex beta subunit Tfg2 (predicted)		2.76
SPBC30B4.02c		R3H and G-patch domain, implicated in splicing		2.75
SPBC8E4.04		aldo/keto reductase involved in pentose catabolism (predicted)		2.75
SPCC306.08c		malate dehydrogenase	malate dehydrogenase	2.74
SPAC4F8.13c	*rng2*	IQGAP	IQGAP	2.74
SPCC417.05c	*chr2*	chitin synthase regulatory factor (putative) Chr2	chitin synthase regulatory factor (putative) Chr2 (PMID 15449309)	2.73
SPCC550.10	*meu8*	aldehyde dehydrogenase Meu8 (predicted)	betaine aldehyde dehydrogenase (predicted)	2.73
SPAC19G12.03	*cda1*	chitin deacetylase Cda1	chitin deacetylase Cda1	2.73
SPCC18.09c		human aprataxin homolog	conserved eukaryotic protein	2.72
SPCC594.04c		steroid oxidoreductase superfamily protein	steroid oxidoreductase superfamily protein	2.72
SPBC2G2.17c		beta-glucosidase Psu2 (predicted)		2.71
SPAC22H12.05c		fasciclin domain protein (3)	fasciclin domain protein (3)	2.71
SPAC186.03		L-asparaginase (predicted)	L-asparaginase	2.71
SPAC22A12.17c		short chain dehydrogenase (predicted)	short chain dehydrogenase (predicted)	2.70
SPBC405.02c		sequence orphan		2.70
SPAC17G6.03		phosphoprotein phosphatase (predicted)	phosphoprotein phosphatase	2.70
SPAC13A11.06		pyruvate decarboxylase (predicted)		2.69
SPAC1F3.02c	*mkh1*	MEK kinase (MEKK) Mkh1	MEK kinase (MEKK) Mkh1 (PMID 9199286)	2.69
SPCC622.02		dubious	dubious	2.69
SPAC57A10.08c		esterase/lipase (predicted)	esterase/lipase (predicted)	2.68
SPAC9E9.05		sequence orphan	sequence orphan	2.68
SPBC947.15c		NADH dehydrogenase		2.68

SPAC16A10.01		DUF1212 family protein	DUF1212 family protein	2.68
SPBC83.01	*ucp8*	UBA/EH/EF hand domain protein Ucp8		2.68
SPBC19C2.11c		mitochondrial outer membrane protein (predicted)		2.67
SPBC1652.01		conserved fungal protein		2.67
SPAC11H11.04	*mam2*	pheromone p-factor receptor	pheromone p-factor receptor (PMID 1657593)	2.67
SPAC11E3.05		ubiquitin-protein ligase E3	ubiquitin-protein ligase E3	2.67
SPAC25H1.09	*mde5*	alpha-amylase homolog Mde5	alpha-amylase homolog Mde5	2.66
SPCC1672.06c	*asp1*	inositol hexakisphosphate kinase/inositol pyrophosphate synthase (predicted)	inositol hexakisphosphate kinase (predicted)	2.66
SPCC330.04c	*mug135*	DUF1773 family protein 3	DUF1773 family protein 3	2.66
SPBP23A10.04	*apc2*	anaphase-promoting complex subunit Apc2		2.66
SPBC29A3.03c		ubiquitin-protein ligase E3 (predicted)		2.65
SPBC3E7.04c		pseudo		2.65
SPBC609.01		ribonuclease II (RNB) family		2.65
SPAC1F5.08c	*yam8*	calcium transport protein	calcium transport protein	2.65
SPBC15D4.07c	*atg9*	autophagy associated protein Atg9 (predicted)		2.65
SPCC31H12.06	*mug111*	sequence orphan	sequence orphan	2.63
SPAC25B8.19c		transcription factor, zf-fungal binuclear cluster type (predicted)		2.62
SPBC21.06c	*cdc7*	serine/threonine protein kinase Cdc7		2.62
SPAC29E6.09		sequence orphan	sequence orphan	2.62
SPAC19A8.02		pleckstrin homology domain protein	transcriptional coactivator (predicted)	2.62
SPAC13C5.06c	*mug121*	sequence orphan	sequence orphan	2.62
SPCC132.03		sequence orphan	sequence orphan	2.62
SPBC1709.16c		aromatic ring-opening dioxygenase (predicted)		2.61
SPCPJ732.03	*meu15*	sequence orphan	sequence orphan	2.61
SPBC32F12.01c		inositol phosphosphingolipid phospholipase C (predicted)		2.61
SPAC11E3.02c		C2 domain protein	C2 domain protein	2.61
SPBP18G5.02		CDP-diacylglycerol-glycerol-3-phosphate3-phosphatidyltransferase		2.60
SPCC1494.03	*arz1*	armadillo repeat containing, Zfs1 target number 1	sequence orphan	2.60

SPBP8B7.24c	*atg8*	autophagy associated protein Atg8 (predicted)		2.60
SPCC622.15c		sequence orphan	sequence orphan	2.60
SPAPB2B4.01c	*gpi12*	pig-L (predicted)	pig-L	2.60
SPBC29B5.01	*atf1*	transcription factor Atf1		2.59
SPAC167.05		Usp (universal stress protein) family protein, implicated in meiotic chromosome segregation	Usp (universal stress protein) family protein,implicated in meiotic chromosome segregation	2.59
SPBC16C6.02c	*vps1302*	chorein homolog		2.59
SPBP4G3.02	*pho1*	acid phosphatase Pho1		2.59
SPAC17C9.11c		zinc finger protein, zf-C2H2 type/UBA domain protein	zinc finger protein	2.58
SPBC2F12.05c		sterol binding ankyrin repeat protein		2.58
SPAC12B10.01c		ubiquitin-protein ligase E3 (predicted)	ubiquitin-protein ligase E3 (predicted)	2.58
SPCC4E9.01c	*rec11*	meiotic cohesin complex subunit Rec11		2.58
SPCC63.14		conserved fungal protein	conserved fungal protein	2.58
SPAC19A8.03		phosphatidylinositol-3-phosphatase (predicted)	phosphatidylinositol-3-phosphatase (predicted)	2.57
SPAC31G5.12c	*maf1*	repressor of RNA polymerase III Maf1 (predicted)	repressor of RNA polymerase III Maf1 (predicted)	2.57
SPAC32A11.03c	*phx1*	homeobox transcription factor Phx1	homeobox transcription factor Phx1	2.57
SPBC660.09	*mug168*	sequence orphan	sequence orphan	2.57
SPAC521.04c		calcium permease (predicted)	calcium permease (predicted)	2.57
SPBC1198.06c		mannan endo-1,6-alpha-mannosidase (predicted)	mannan endo-1,6-alpha-mannosidase (predicted)	2.56
SPBC31E1.04	*pep12*	SNARE Pep12	SNARE Pep12	2.56
SPCC132.04c		NAD-dependent glutamate dehydrogenase (predicted)	NAD-dependent glutamate dehydrogenase (predicted)	2.56
SPBC32H8.13c	*mok12*	alpha-1,3-glucan synthase Mok12		2.56
SPAC24C9.07c	*bgs2*	1,3-beta-glucan synthase subunit Bgs2	1,3-beta-glucan synthase subunit Bgs2	2.55
SPAC22H10.02		conserved fungal protein	conserved fungal protein	2.55
SPBC21B10.11	*dpm2*	dolichol-phosphate mannosyltransferase subunit 2 (predicted)		2.55
SPAC22F3.04	*mug62*	AMP binding enzyme (predicted)	AMP binding enzyme (predicted)	2.55
SPBC14F5.11c	*mug186*	sorting nexin Snx41		2.55
SPBC23E6.01c		RNA-binding protein, rrm type		2.54
SPBC16C6.04		sequence orphan		2.53

SPBC1778.02	*rap1*	telomere binding protein Rap1		2.53
SPBC56F2.05c		transcription factor (predicted)		2.53
SPAC4F10.02		aminopeptidase (predicted)	aspartyl aminopeptidase (predicted)	2.52
SPAC630.07c		sequence orphan	sequence orphan	2.52
SPBP23A10.14c	*ell1*	RNA polymerase II transcription elongation factor SpELL		2.52
SPCC162.12	*tco89*	TORC1 subunit Tco89	sequence orphan	2.52
SPBC25H2.03		vacuolar protein involved in phosphoinositide metabolism		2.51
SPBC16C6.03c		conserved fungal protein		2.51
SPBC530.05		transcription factor (predicted)		2.51
SPCC417.11c		glutamate-1-semialdehyde 2,1-aminomutaseaminotransferase (predicted)	glutamate-1-semialdehyde 2,1-aminomutaseaminotransferase (predicted)	2.51
SPACUNK4.08		dipeptidyl aminopeptidase (predicted)	dipeptidyl aminopeptidase (predicted)	2.50
SPBP4H10.07		ubiquitin-protein ligase E3 (predicted)		2.50
SPAPB1A10.07c		sphingolipid biosynthesis protein	sphingolipid biosynthesis protein	2.50
SPCC24B10.02c		NAD/NADH kinase (predicted)	NAD/NADH kinase	2.49
SPCC18B5.05c		phosphomethylpyrimidine kinase (predicted)	phosphomethylpyrimidine kinase (predicted)	2.49
SPBC21C3.20c	*git1*	C2 domain protein Git1		2.49
SPCC1739.01		zf-CCCH type zinc finger protein	zf-CCCH type zinc finger protein	2.49
SPBC336.12c	*cdc10*	MBF transcription factor complex subunit Cdc10		2.49
SPAC10F6.04		RCC domain protein Ats1 (predicted)	RCC domain protein Ats1 (predicted)	2.48
SPCC1442.04c		conserved fungal protein	conserved fungal protein	2.48
SPAC27E2.07	*pvg2*	galactose residue biosynthesis protein Pvg2	galactose residue biosynthesis protein Pvg2	2.48
SPAC11G7.03	*idh1*	isocitrate dehydrogenase (NAD+) subunit 1 Idh1	isocitrate dehydrogenase (NAD+) subunit 1 Idh1 (PMID 10975257)	2.48
SPAC5H10.02c		ThiJ domain protein	ThiJ domain protein	2.47
SPBC1711.02	*matmc_1*	mating-type m-specific polypeptide mc		2.47
SPBC691.05c		membrane transporter (predicted)		2.46
SPAC17C9.05c	*pmc3*	mediator complex subunit Pmc3 (pers. comm. Tomas Linder, from PMID 10625684)	mediator complex subunit Pmc3 (pers. comm. Tomas Linder, from PMID 10625684)	2.46
SPCC965.06		potassium channel subunit (predicted)	potassium channel subunit (predicted)	2.46
SPAC1D4.03c	*aut12*	autophagy associated protein Aut12 (predicted)	autophagy associated protein Aut12	2.46

SPAC6F12.03c	*fsv1*	SNARE Fsv1	SNARE Fsv1	2.45
SPBC947.10		ubiquitin-protein ligase E3 (predicted)		2.45
SPAC24C9.08		vacuolar carboxypeptidase (predicted)	vacuolar carboxypeptidase (predicted)	2.45
SPCC320.07c	*mde7*	RNA-binding protein Mde7	RNA-binding protein Mde7	2.45
SPAC17A2.11		sequence orphan	sequence orphan	2.44
SPAPB18E9.02c	*ppk18*	serine/threonine protein kinase Ppk18 (predicted)	serine/threonine protein kinase Ppk18 (predicted)	2.44
SPCC24B10.07	*gad8*	serine/threonine protein kinase Gad8	serine/threonine protein kinase Gad8 (PMID 12805221)	2.44
SPAC1556.04c	*cdd1*	cytidine deaminase Ccd1	cytidine deaminase Pcd1	2.43
SPCC737.09c	*hmt1*	ATP-binding cassette-type vacuolar membrane transporter Hmt1		2.43
SPCC191.04c		dubious	dubious	2.43
SPBC691.01		palmitoyltransferase (predicted)		2.43
SPBC31E1.01c	*atg2*	autophagy associated protein Mug36	autophagy associated protein Mug36	2.43
SPCC1281.04		pyridoxal reductase (predicted)	pyridoxal reductase (predicted)	2.43
SPBC36.10		mitochondrial intermembrane space protein sorting protein		2.42
SPAC11D3.08c		amino acid permease, unknown 1	amino acid permease, unknown 1	2.42
SPCC320.05		sulphate transporter (predicted)	sulphate transporter (predicted)	2.42
SPBC4F6.06	*kin1*	microtubule affinity-regulating kinase Kin1		2.42
SPBC12C2.12c	*glo1*	glyoxalase I		2.42
SPBC16A3.13	*meu7*	alpha-amylase homolog Aah4		2.42
SPBC16C6.06	*pep1*	sorting receptor for CPY		2.42
SPAC22F8.02c	*pvg5*	PvGal biosynthesis protein Pvg5	PvGal biosynthesis protein Pvg5	2.41
SPBC27B12.05		WD repeat protein		2.41
SPAC12G12.11c		DUF544 family protein	DUF544 family protein	2.41
SPAC17G8.10c	*dma1*	mitotic spindle checkpoint protein Dma1	mitotic spindle checkpoint protein Dma1	2.41
SPAC31A2.12		arrestin/PY protein 1	arrestin/PY protein 1	2.40
SPAC17A2.07c		sequence orphan	sequence orphan	2.40
SPCC1393.05		sequence orphan	sequence orphan	2.40
SPBC6B1.05c		ubiquitin-like conjugating enzyme		2.40
SPBC26H8.04c		DEP domain protein		2.40
SPCC162.04c	*wtf13*	wtf element Wtf13	wtf element Wtf13	2.39
SPAC1952.04c			dubious	2.39
SPAPB2B4.03	*cig2*	cyclin Cig2	cyclin Cig2	2.39

SPAC4A8.10			lipase (predicted)	lipase (predicted)	2.38
SPAC14C4.07			membrane transporter	membrane transporter	2.38
SPAC23D3.13c			guanyl-nucleotide exchange factor (predicted)	guanyl-nucleotide exchange factor	2.38
SPBC3B9.06c	*apg3*		autophagy associated protein Apg3 (predicted)	autophagy associated protein Apg3 (predicted)	2.38
SPAC1F3.10c		*39722*	mitochondrial intermediate peptidase Oct1 (predicted)	mitochondrial intermediate peptidase Oct1 (predicted)	2.38
SPCC1183.11			MS ion channel protein 1		2.38
SPAC22H10.10	*alp21*		tubulin specific chaperone cofactor E	tubulin specific chaperone cofactor E	2.38
SPAC17G8.12			sequence orphan	sequence orphan	2.37
SPAC1002.12c			succinate-semialdehyde dehydrogenase (predicted)	succinate-semialdehyde dehydrogenase (predicted)	2.37
SPCC736.09c			TRAX	TRAX (PMID 16043634)	2.37
SPAC1F7.10			hydantoin racemase family (predicted)	hydantoin racemase family (predicted)	2.37
SPAC1296.03c	*sxa2*		serine carboxypeptidase Sxa2	serine carboxypeptidase Sxa2	2.37
SPBC1198.01			glutathione-dependent formaldehyde dehydrogenase (predicted)	glutathione-dependent formaldehyde dehydrogenase (predicted)	2.37
SPBC14F5.10c			ubiquitin-protein ligase E3 (predicted)		2.36
SPBC725.05c			nucleotide pyrophosphatase (predicted)		2.36
SPAC328.04			AAA family ATPase, unknown biological role	AAA family ATPase, unknown biological role	2.36
SPCC338.17c	*rad21*		kleisin	kleisin	2.36
SPAC22E12.06c	*gmh3*		alpha-1,2-galactosyltransferase Gmh3	alpha-1,2-galactosyltransferase Gmh3	2.36
SPAC25B8.18			mitochondrial electron carrier (predicted)	mitochondrial electron carrier (predicted)	2.36
SPAC630.15	*mug177*		sequence orphan	sequence orphan	2.36
SPCC4G3.08	*psk1*		serine/threonine protein kinase Psk1	serine/threonine protein kinase Psk1	2.36
SPBC1709.11c	*png2*		ING family homolog Png2		2.35
SPBC902.05c	*idh2*		isocitrate dehydrogenase (NAD+) subunit 2	isocitrate dehydrogenase (NAD+) subunit 2	2.35
SPBC1604.18c			vacuolar sorting protein (predicted)		2.35
SPAPB15E9.02c			dubious	dubious	2.35
SPAC22H10.07	*scd2*		scaffold protein Scd2	scaffold protein Scd2	2.35
SPAC23H4.01c			sterol binding ankyrin repeat protein	sterol binding ankyrin repeat protein	2.34
SPCC663.02	*wtf14*		wtf element Wtf14	wtf element Wtf14	2.34
SPAC17A2.06c	*vps8*		WD repeat protein Vps8	WD repeat protein Vps8	2.34
SPBC1773.06c			alcohol dehydrogenase (predicted)	alcohol dehydrogenase (predicted)	2.34

SPAC4G8.03c		RNA-binding protein	RNA-binding protein	2.34
SPAC56E4.06c	*ggt2*	gamma-glutamyltranspeptidase Ggt2	gamma-glutamyltranspeptidase Ggt2	2.34
SPAC1F3.03		Lgl family protein	Lgl family protein	2.33
SPBC16E9.11c	*pub3*	ubiquitin-protein ligase E3		2.33
SPAC6G10.10c		human hmmtag2 homolog	human hmmtag2 homolog	2.33
SPAC2F3.16		ubiquitin-protein ligase E3 (predicted)	ubiquitin-protein ligase E3 (predicted)	2.33
SPAC4D7.10c		SAGA complex subunit Spt20 (predicted)	SAGA complex subunit Spt20 (predicted)	2.33
SPAC167.07c		ubiquitin-protein ligase E3 (predicted)	ubiquitin-protein ligase E3 (predicted)	2.32
SPCC1259.12c		Ran GTPase binding protein (predicted)	Ran GTPase binding protein (predicted)	2.32
SPAC1687.08		sequence orphan	sequence orphan	2.32
SPAC15A10.16	*bud6*	actin interacting protein 3 homolog Bud6		2.32
SPAP11E10.02c	*mam3*	cell agglutination protein Mam3		2.31
SPAC140.04		conserved fungal protein	conserved fungal protein	2.31
SPAC16.05c	*sfp1*	transcription factor Sfp1 (predicted)	transcription factor Sfp1 (predicted)	2.31
SPAC16A10.08c	*mug74*	sequence orphan		2.30
SPAP32A8.02		xylose and arabinose reductase (predicted)	xylose and arabinose reductase (predicted)	2.30
SPAC6F12.02	*rst2*	transcription factor Rst2	transcription factor Rst2	2.30
SPAC2E1P3.01		zinc binding dehydrogenase	zinc binding dehydrogenase	2.30
SPAC732.02c		fructose-2,6-bisphosphate 2-phosphatase activity (predicted)	6-phosphofructo-2-kinase (predicted)	2.30
SPCC188.07	*ccq1*	telomere maintenence protein	telomere maintenence protein	2.30
SPCC757.07c	*ctt1*	catalase	catalase	2.30
SPBC1685.01	*pmp1*	dual-specificity MAP kinase phosphatase Pmp1	dual-specificity MAP kinase phosphatase Pmp1	2.29
SPAC6F12.12	*par2*	protein phosphatase regulatory subunit Par2	protein phosphatase regulatory subunit Par2	2.29
SPAC9.13c	*cwf16*	splicing factor (predicted)		2.29
SPAPB2B4.04c		P-type ATPase, calcium transporting Pmc1	P-type ATPase, calcium transporting Pmc1 (PMID 12707717)	2.29
SPAPB8E5.04c		Niemann-Pick disease type C2 protein hE1 homolog	phosphatidylglycerol/phosphatidylinositol transfer protein (predicted)	2.29
SPAC13G6.12c	*chs1*	chitin synthase I		2.28
SPCC4F11.02	*ptc1*	protein phosphatase 2C Ptc1	protein phosphatase 2C Ptc1	2.28
SPAC3A12.08		conserved fungal protein	conserved fungal protein	2.28
SPBC11C11.12		pseudogene (predicted)		2.28

SPCC290.04	*ams2*	cell cycle regulated GATA-type transcription factor Ams2		2.28
SPAC664.15		CCR4-Not complex subunit Caf4/Mdv1 (predicted)	CCR4-Not complex subunit Caf4/Mdv1 (predicted)	2.28
SPBC887.15c		sphingosine hydroxylase (predicted)		2.27
SPCC965.05c	*thp1*	uracil DNA N-glycosylase Thp1	uracil DNA N-glycosylase Thp1 (PMID 12711670)	2.27
SPBC1271.09		glycerophosphodiester transporter	glycerophosphodiester transporter	2.27
SPBC337.15c	*coq7*	ubiquinone biosynthesis protein Coq7		2.27
SPCC18B5.11c	*cds1*	replication checkpoint kinase Cds1	replication checkpoint kinase Cds1	2.27
SPAC6F6.08c	*cdc16*	two-component GAP Cdc16	two-component GAP Cdc16	2.26
SPAC6C3.08		proteasome regulatory particle, gankyrin (predicted)	gankyrin	2.26
SPAC1687.07		conserved fungal protein	conserved fungal protein	2.26
SPAC3C7.03c	*rhp55*	RecA family ATPase Rhp55	RecA family ATPase Rhp55	2.26
SPAC29E6.01	*pof11*	F-box protein Pof11	F-box protein Pof11	2.26
SPCC569.06		S. pombe specific multicopy membrane protein family 1	S. pombe specific multicopy membrane protein family 1	2.26
SPCC1259.14c	*meu27*	S. pombe specific UPF0300 family protein 5	S. pombe specific UPF0300 family protein 5	2.25
SPCC4B3.16	*tip41*	TIP41-like type 2a phosphatase regulator Tip41	TIP41-like type 2a phosphatase regulator Tip41	2.25
SPAC22A12.11	*dak1*	dihydroxyacetone kinase Dak1	dihydroxyacetone kinase Dak1	2.25
SPAC22A12.01c	*pso2*	DNA 5' exonuclease (predicted)	DNA 5' exonuclease (predicted)	2.24
SPCC1919.01	*ppk34*	serine/threonine protein kinase Ppk34	serine/threonine protein kinase Ppk34	2.24
SPAC17A5.18c	*rec25*	meiotic recombination protein Rec25	meiotic recombination protein Rec25	2.24
SPBP23A10.09		GINS complex subunit Psf1 (predicted)		2.24
SPAC3C7.02c		protein kinase inhibitor (predicted)	protein kinase inhibitor (predicted)	2.24
SPBP35G2.14		RNA-binding protein		2.24
SPAC1142.03c	*swi2*	Swi5 complex subunit Swi2	Swi5 complex subunit Swi2	2.23
SPAP14E8.04	*oma1*	metallopeptidase Oma1 (predicted)	metallopeptidase Oma1 (predicted)	2.23
SPAC823.03	*ppk15*	serine/threonine protein kinase Ppk15 (predicted)	serine/threonine protein kinase Ppk15 (predicted)	2.23
SPBC17D1.07c		GTPase regulator (predicted)		2.23
SPCC126.04c		SAGA complex subunit Sgf73 (predicted)	SAGA complex subunit Sgf73 (predicted)	2.22
SPBC13E7.11		mitochondrial rhomboid protease		2.22
SPAC31G5.10	*eta2*	Myb family protein Eta2	Myb family transcriptional regulator Eta2	2.22

SPCC16A11.01		plasma membrane protein involved in inositol lipid-mediated signaling	conserved fungal protein	2.21
SPAC4D7.11		conserved fungal protein	conserved fungal protein	2.21
SPCC1494.01		iron/ascorbate oxidoreductase family		2.21
SPBC1773.08c		mannosyltransferase complex subunit (predicted)	mannosyltransferase complex subunit (predicted)	2.21
SPAC343.06c		phospholipid scramblase	scramblase	2.21
SPBC27B12.01c	*mmm1*	Mdm10/Mdm12/Mmm1 complex subunit Mmm1 (predicted)		2.21
SPCC1450.12		conserved fungal protein	conserved fungal protein	2.21
SPBC557.05		arrestin		2.21
SPAC22A12.02c	*mug103*	sequence orphan	sequence orphan	2.20
SPBC13E7.02	*cwf24*	zf-C3HC4 type (RING finger)/GCN5-related N acetyltransferase fusion protein		2.20
SPCC1919.03c		AMP-activated protein kinase beta subunit (predicted)	AMP-activated protein kinase beta subunit (predicted)	2.20
SPCC126.09		vacuolar membrane zinc transporter (predicted)	vacuolar membrane zinc transporter (predicted)	2.20
SPBC1D7.05	*byr2*	MAP kinase kinase kinase Byr2		2.20
SPBC17D11.01	*nep1*	NEDD8 protease Nep1		2.19
SPBC83.05		mitochondrial RNA-binding protein (predicted)		2.19
SPAC1834.09	*mug51*	conserved fungal protein	conserved fungal protein	2.19
SPAC25B8.03		phosphatidylserine decarboxylase	phosphatidylserine decarboxylase	2.19
SPAC977.04		pseudogene	pseudogene	2.18
SPAC2E1P3.02c	*amt3*	ammonium transporter Amt3	ammonium transporter Amt3	2.18
SPCC1919.06c	*wtf25*	wtf element	wtf element	2.18
SPCC1235.04c		FAD synthetase	FAD synthetase	2.18
SPAC1296.04	*mug65*	spore wall assembly protein (predicted)	spore wall assembly protein (predicted)	2.18
SPAC3H1.11	*hsr1*	transcription factor Hsr1	transcription factor Hsr1	2.17
SPAC4G9.13c	*vps26*	retromer complex subunit Vps26	retromer complex subunit Vps26	2.17
SPBC1105.10	*rav1*	RAVE complex subunit Rav1 (predicted)		2.17
SPCC1450.06c	*grx3*	monothiol glutaredoxin Grx3	monothiol glutaredoxin Grx3	2.17
SPAC26F1.04c	*etr1*	enoyl-[acyl-carrier protein] reductase	enoyl-[acyl-carrier protein] reductase	2.17
SPCC74.06	*mak3*	histidine kinase Mak3	histidine kinase Mak3 (PMID 11758939)	2.17
SPAC3C7.06c	*pit1*	serine/threonine protein kinase Pit1	serine/threonine protein kinase Pit1	2.17

SPBC19G7.13	*tbf1*	telomeric repeat binding factor Trf1		2.16
SPBC337.12		human ZC3H3 homolog		2.16
SPAC22F8.04		triose phosphate transporter (predicted)	triose phosphate transporter (predicted)	2.15
SPCC1235.06	*sif1*	Sad1 interacting factor 1	Sad1 interacting factor 1	2.15
SPBC20F10.06	*mad2*	spindle checkpoint protein Mad2		2.15
SPAC227.04		autophagy C terminal domain family protein	autophagy C terminal domain family protein	2.15
SPBC25B2.10		Usp (universal stress protein) family protein		2.15
SPAC23C4.16c	*atg15*	triacylglycerol lipase Atg15 (predicted)	triacylglycerol lipase Atg15 (predicted)	2.15
SPCC24B10.03		sequence orphan	sequence orphan	2.15
SPAC23H4.14	*vam6*	guanyl-nucleotide exchange factor Vam6	guanyl-nucleotide exchange factor Vma6	2.15
SPCC417.10		membrane transporter	membrane transporter	2.15
SPAPB17E12.09		sequence orphan	sequence orphan	2.15
SPCC622.01c		sequence orphan	sequence orphan	2.15
SPAC27D7.08c		DUF890 family protein	DUF890 family protein	2.14
SPCC757.11c		membrane transporter	membrane transporter	2.14
SPAC17G6.02c	*tco1*	RTA1-like protein	RTA1-like protein	2.14
SPBP16F5.03c	*tra1*	phosphatidylinositol kinase-related protein Tra1		2.14
SPAC20G8.02		phospholipase (predicted)	phospholipase	2.14
SPBC651.05c	*dot2*	EAP30 family protein Dot2		2.14
SPCC330.01c	*rhp16*	Rad16 homolog Rhp16	Rad16 homolog Rhp16	2.14
SPAC13G7.07	*arb2*	argonaute binding protein 2	argonaute binding protein 2	2.13
SPAC57A7.08	*pzh1*	serine/threonine protein phosphatase Pzh1	serine/threonine protein phosphatase Pzh1	2.13
SPAC23C4.05c		LEA domain protein	LEA domain protein	2.13
SPCC569.03		DUF1773 family protein 4	DUF1773 family protein 4	2.13
SPAP7G5.06		amino acid permease, unknown 4	amino acid permease, unknown 4	2.13
SPAC328.07c		heavy metal ion homeostasis protein (predicted)	heavy metal ion homeostasis protein (predicted)	2.13
SPBC725.02	*mpr1*	response regulator phosphotransferase		2.13
SPAC227.14		nicotinamide riboside kinase (predicted)	nicotinamide riboside kinase	2.13
SPBP35G2.11c		transcription related zf-ZZ type zinc finger protein		2.13
SPBC11C11.06c		sequence orphan		2.12

SPCC645.02		conserved protein (fungal and plant)	conserved protein (fungal and plant)	2.12
SPAC644.06c	*cdr1*	GIN4 family protein kinase Cdr1	GIN4 family protein kinase Cdr1	2.12
SPAC20G8.10c		beclin family protein		2.12
SPACUNK12.02c	*cmk1*	calcium/calmodulin-dependent protein kinase Cmk1	calcium/calmodulin-dependent protein kinase Cmk1	2.12
SPBC354.09c		Tre1 family protein (predicted)	Tre1 family protein (predicted)	2.12
SPBC14C8.08c		dubious		2.12
SPCC962.01		C2 domain protein	C2 domain protein	2.11
SPAC17A2.10c		sequence orphan	sequence orphan	2.11
SPBC336.07	*sfc3*	transcription factor TFIIIC complex subunit Sfc3		2.11
SPAC6F12.08c		exocyst complex subunit Exo84	exocyst complex subunit Exo84	2.11
SPBC354.05c	*sre2*	membrane-tethered transcription factor (predicted)	membrane-tethered transcription factor (predicted) (PMID 11790253)	2.11
SPAC5D6.08c	*mes1*	meiosis II protein Mes1	meiosis II protein Mes1	2.11
SPCC970.09	*sec8*	exocyst complex subunit Sec8	exocyst complex subunit Sec8	2.11
SPBC16A3.18	*cip1*	RNA-binding protein Cip1		2.11
SPBC1703.04	*mlh1*	MutL family protein Mlh1 (predicted)		2.10
SPBC800.13		histone H4 variant	histone H4 variant	2.10
SPBC16D10.05	*mok13*	alpha-1,3-glucan synthase Mok13		2.10
SPCC1235.08c	*pdh1*	DUF1751 family protein	DUF1751 family protein	2.10
SPCC61.05		S. pombe specific multicopy membrane protein family 1	S. pombe specific multicopy membrane protein family 1	2.10
SPBC365.20c		nicotinamidase (predicted)		2.09
SPAC227.03c		mitochondrial NAD+ transporter (predicted)	mitochondrial NAD+ transporter	2.09
SPAC458.05	*pik3*	phosphatidylinositol 3-kinase Pik3	phosphatidylinositol 3-kinase Pik3	2.09
SPBC13E7.10c	*brf1*	transcription factor TFIIIB complex subunit Brf1		2.09
SPAC27E2.09	*mak2*	histidine kinase Mak2	histidine kinase Mak2 (PMID 11758939)	2.09
SPBC365.05c	*slu7*	splicing factor Slu7		2.09
SPBP4H10.17c		carboxyl methyl esterase		2.09
SPAC14C4.10c		Nudix family hydrolase	Nudix family hydrolase	2.09
SPBC1773.03c		aminotransferase class-III, unknown specificty	aminotransferase class-III (predicted)	2.08
SPCC777.13	*vps35*	retromer complex subunit Vps35	retromer complex subunit Vps35	2.08
SPBC119.04	*mei3*	meiosis inducing protein Mei3		2.08

SPBC15D4.11c		sequence orphan		2.08
SPBP4H10.11c	*lcf2*	long-chain-fatty-acid-CoA ligase		2.08
SPBC2A9.03		conserved protein (fungal and plant)		2.08
SPAC8F11.05c	*mug130*	sequence orphan	sequence orphan	2.08
SPAC17G6.08	*pep7*	prevacuole/endosomal FYVE tethering component Pep7 (predicted)	prevacuole/endosomal FYVE tethering component Pep7 (predicted)	2.08
SPAPB1A10.10c	*ypt71*	GTPase Ypt71	GTPase Ypt71	2.08
SPAC6F6.12		autophagy associated protein Atg24 (predicted)	autophagy associated protein Atg24	2.08
SPAC11D3.16c		sequence orphan	sequence orphan	2.07
SPBPB21E7.01c	*eno102*	enolase (predicted)	enolase (predicted)	2.07
SPBC17F3.02	*nak1*	PAK-related kinase Nak1		2.07
SPAC1783.06c	*atg12*	autophagy associated protein Atg12	autophagy associated protein Atg12	2.07
SPAC1F7.03	*pkd2*	TRP-like ion channel	TRP-like ion channel (PMID 15537393)	2.07
SPCPB1C11.01	*amt1*	ammonium transporter Amt1	ammonium transporter Amt1	2.06
SPCC737.05		peroxin Pex28/29	peroxin Pex28/29	2.06
SPBC947.08c		histone promoter control protein Hpc2 (predicted)		2.06
SPBC1718.07c	*zfs1*	CCCH tandem zinc finger protein, human Tristetraprolin homolog Zfs1		2.06
SPAC26H5.09c		GFO/IDH/MocA family oxidoreductase	GFO/IDH/MocA family oxidoreductase	2.06
SPBC2G2.06c	*apl1*	AP-2 adaptor complex subunit Apl1 (predicted)		2.06
SPBP8B7.13		conserved fungal protein		2.06
SPCC550.09		peroxin Pex32 (predicted)	peroxin Pex32 (predicted)	2.06
SPCC576.06c		tyrosine-tRNA ligase	tyrosine-tRNA ligase	2.06
SPBC23E6.03c	*nta1*	protein N-terminal amidase Nta1 (predicted)		2.06
SPAC22H10.06c		dubious	dubious	2.06
SPAC18G6.10		chromosome segregation protein Heh1 (predicted)	chromosome segregation protein (predicted)	2.06
SPAC7D4.04	*taf1*	Taz1 interacting factor 1	Taz1 interacting factor 1	2.06
SPBC29B5.02c	*isp4*	OPT oligopeptide transporter family		2.05
SPBC11B10.05c	*rsp1*	random septum position protein Rsp1		2.05
SPBPJ4664.03	*mfm3*	M-factor precursor Mfm3		2.05
SPBC1E8.02		ubiquitin family protein, unknown		2.05
SPAPB1E7.05	*gde1*	glycerophosphoryl diester phosphodiesterase Gde1	glycerophosphoryl diester phosphodiesterase	2.05

SPAC25G10.01		RNA-binding protein	RNA-binding protein	2.05
SPAC56F8.13		dubious	dubious	2.05
SPBC21.05c	*ral2*	Ras guanyl-nucleotide exchange factor Ral2 (predicted)		2.05
SPAC1039.11c		alpha-glucosidase	alpha-glucosidase	2.05
SPBC11C11.01		RNA-binding protein		2.04
SPCC1672.04c		mitochondrial copper chaperone	mitochondrial copper ion transport protein	2.04
SPBC12D12.02c	*cdm1*	DNA polymerase delta subunit Cdm1		2.04
SPCC1183.09c	*pmp31*	plasma membrane proteolipid Pmp31	plasma membrane proteolipid Pmp31	2.04
SPCC1753.02c	*git3*	G-protein coupled receptor Git3	G-protein coupled receptor Git3	2.04
SPAC57A10.02	*cdr2*	GIN4 family protein kinase Cdr2	GIN4 family protein kinase Cdr2	2.04
SPBC16A3.17c		membrane transporter		2.04
SPCC330.11	*btb1*	BTB/POZ domain protein Btb1	BTB/POZ domain protein Btb1	2.04
SPAC1565.07c		human CAND1/TIP120 ortholog	TATA binding protein interacting protein (predicted)	2.04
SPBC2A9.07c		zf-PARP type zinc finger protein		2.03
SPCC70.02c		mitochondrial ATPase inhibitor (predicted)	mitochondrial ATPase inhibitor (predicted)	2.03
SPAC16A10.06c	*nse2*	Smc5-6 complex non-SMC subunit 2	Smc5-6 complex non-SMC subunit 2 (PMID 12966087)	2.03
SPCC736.05	*wtf7*	wtf element Wtf7	wtf element Wtf7	2.03
SPBC3B8.07c	*dsd1*	dihydroceramide delta-4 desaturase		2.03
SPAC6F6.16c		sequence orphan	sequence orphan	2.03
SPAC15A10.13	*ppk3*	serine/threonine protein kinase Ppk3	serine/threonine protein kinase Ppk3	2.03
SPBC365.08c		Der1-like (degradation in the ER) family		2.03
SPCC777.17c		mitochondrial ribosomal protein subunit L9 (predicted)	mitochondrial ribosomal protein subunit L9 (predicted)	2.03
SPCC10H11.02	*cwf23*	DNAJ domain protein Cwf23		2.03
SPCC16A11.04	*snx12*	sorting nexin Snx12 (predicted)	sorting nexin Snx12 (predicted)	2.03
SPAC9E9.15		CIA30 family protein	CIA30 family protein	2.03
SPBC1709.14		peptide N-glycanase (predicted)		2.03
SPAC1142.08	*fhl1*	fork head transcription factor Fhl1		2.02
SPCC1620.14c	*snf22*	ATP-dependent DNA helicase Snf22	ATP-dependent DNA helicase Snf22	2.02
SPAC13G7.05		acyl-coA-sterol acyltransferase (predicted)	acyl-coA-sterol acyltransferase (predicted)	2.02
SPAC1B1.02c		NAD/NADH kinase (predicted)	NAD/NADH kinase (predicted)	2.01

SPAC1D4.12	*rad15*	transcription factor TFIIH complex subunit Rad15	transcription factor TFIIH complex subunit Rad15	2.01
SPBC1289.10c		transcription factor (predicted)		2.01
SPBC713.02c	*ubp21*	ubiquitin C-terminal hydrolase Ubp21		2.01
SPBP35G2.06c	*nup131*	nucleoporin Nup131		2.01
SPBP8B7.26		sequence orphan		2.01
SPBC1773.09c	*mug184*	meiotically upregulated gene Mug184	meiotically upregulated gene Mug184	2.01
SPAC105.01c		potassium ion/proton antiporter	potassium ion/proton antiporter	2.00
SPBC354.11c		dubious	sequence orphan	2.00
SPBC23G7.16	*ctr6*	vacuolar copper transporter Ctr6		2.00
SPBC29A3.08	*pof4*	elongin-A, F-box protein Pof4		2.00
SPBC3H7.03c		2-oxoglutarate dehydrogenase (lipoamide) (e1 component of oxoglutarate dehydrogenase complex) (predicted)		2.00
SPAC57A10.05c	*pof1*	F-box/WD repeat protein protein Pof1	F-box protein Pof1	2.00
SPAC1F3.08c		dubious	dubious	2.00
SPAC21E11.03c	*pcr1*	transcription factor Pcr1	transcription factor Pcr1	2.00
SPBC14C8.09c		conserved fungal protein		2.00
SPBC543.05c		inorganic anion exchanger (predicted)		1.99
SPBP18G5.03	*toc1*	Tor Complex Tor2 interacting protein 1		1.99
SPAC2F3.10		GARP complex subunit Vps54 (predicted)	GARP complex subunit Vps54 (predicted)	1.99
SPCC1739.04c		sequence orphan	sequence orphan	1.99
SPBC211.07c	*ubc8*	ubiquitin conjugating enzyme Ubc8		1.99
SPAC9.10	*thi9*	thiamine transporter Thi9	amino acid permease, unknown 2	1.99
SPAC3H1.14		cytoplasmic vesicle protein, Vid24 family	cytoplasmic vesicle protein, Vid24 family	1.99
SPAC2G11.03c	*vps45*	vacuolar sorting protein Vps 45 (predicted)	vacuolar sorting protein Vps 45	1.99
SPAC1006.01	*psp3*	serine protease Psp3 (predicted)	serine protease Psp3 (predicted)	1.99
SPAC3H8.06	*aur1*	inositol phosphorylceramide synthase (predicted)	inositol phosphorylceramide synthase (predicted)	1.99
SPCC1322.05c		leukotriene A-4 hydrolase (predicted)	leukotriene A-4 hydrolase (predicted)	1.99
SPAC24B11.12c		P-type ATPase	P-type ATPase (PMID 12707717)	1.99
SPBC1683.13c		transcription factor (predicted)	transcription factor (predicted)	1.98
SPCC4F11.05		dubious	dubious	1.98
SPBC31F10.02		thioesterase superfamily protein		1.98

SPCC1827.07c		SPX/EXS domain protein	SPX/EXS domain protein	1.98
SPCC285.09c	*cgs2*	cAMP-specific phosphodiesterase Cgs2	cAMP-specific phosphodiesterase Cgs2	1.98
SPBC660.07	*ntp1*	alpha,alpha-trehalase Ntp1	alpha,alpha-trehalase Ntp1	1.98
SPAC22E12.11c	*set3*	histone lysine methyltransferase Set3	histone lysine methyltransferase Set3	1.98
SPAC2G11.04		RNA-binding protein, G-patch type (predicted)	RNA-binding protein (predicted)	1.98
SPAC23H3.13c	*gpa2*	heterotrimeric G protein alpha-2 subunit Gpa2	heterotrimeric G protein alpha-2 subunit Gpa2 (PMID 1340462)	1.97
SPAC13G7.03		up-frameshift suppressor3 family	up-frameshift suppressor3 family	1.97
SPBC215.13		sequence orphan		1.97
SPAC4G8.10	*gos1*	SNARE Gos1 (predicted)	SNARE Gos1	1.97
SPCC1183.05c	*lig4*	DNA ligase Lig4	DNA ligase Lig4	1.97
SPAC458.06		phosphoinositide binding protein	phosphoinositide binding protein	1.97
SPBC530.04	*mod5*	Tea1 anchoring protein Mod5		1.97
SPAC18B11.09c		N-acetyltransferase (predicted)	N-acetyltransferase (predicted)	1.97
SPAC19G12.04		ureidoglycolate hydrolase (predicted)	ureidoglycolate hydrolase (predicted)	1.96
SPBC21C3.11	*ubx4*	UBX domain protein Ubx4 (predicted)		1.96
SPAPB1A10.13		sequence orphan	sequence orphan	1.96
SPCC16C4.01	*sif2*	Sad1 interacting factor 2		1.96
SPCC1919.14c	*bdp1*	transcription factor TFIIIB complex subunit Bdp1 (predicted)	transcription factor TFIIIB complex subunit Bdp1 (predicted)	1.96
SPAC56F8.16	*esc1*	transcription factor Esc1 (predicted)	transcription factor Esc1 (predicted)	1.96
SPAC3A11.11c		pyridoxal reductase (predicted)	pyridoxal reductase (predicted)	1.96
SPAC8F11.10c	*pvg1*	pyruvyltransferase		1.96
SPBP8B7.07c	*set6*	histone lysine methyltransferase Set6 (predicted)		1.96
SPAC30D11.11		Haemolysin-III family protein	Haemolysin-III family protein	1.96
SPAC25G10.03	*zip1*	transcription factor Zip1	transcription factor Zip1	1.96
SPBC337.02c		pseudogene		1.96
SPBC26H8.01	*thi2*	thiazole biosynthetic enzyme		1.96
SPCC777.05	*gtr2*	Gtr1/RagA G protein Gtr2	Gtr1/RagA G protein Gtr2	1.96
SPBC1734.08	*hse1*	STAM like protein Hse1		1.95
SPCC11E10.03	*mug1*	dynactin complex subunit (predicted)	dynactin complex subunit (predicted)	1.95
SPAPB1A10.08		sequence orphan	sequence orphan	1.95
SPAC2C4.09		DUF1640 family protein	DUF1640 family protein	1.95
SPAC31A2.05c	*mis4*	cohesin loading factor Mis4	cohesin loading factor Mis4	1.95
SPCC622.05		dubious	dubious	1.95

SPAC17G6.12	*cul1*	cullin 1	cullin 1	1.95
SPBC21C3.03		ABC1 kinase family protein		1.94
SPAC1610.04	*mug99*	meiotically upregulated gene Mug99	meiotically upregulated gene Mug99	1.94
SPAC29B12.14c		purine transporter (predicted)	purine transporter (predicted)	1.94
SPBC3B9.10	*vti1*	SNARE Vti1		1.94
SPAC10F6.11c		autophagy associated kinase activator (predicted)	kinase activator (predicted)	1.94
SPBC8D2.01	*gsk31*	serine/threonine protein kinase Gsk31		1.94
SPCC417.07c	*mto1*	MT organizer Mto1	MT organizer Mto1	1.94
SPBC1718.04		glycerol-3-phosphate O-acyltransferase (predicted)		1.94
SPAC1687.14c		EF hand family protein, unknown role	EF hand family protein, unknown role	1.94
SPAC607.09c	*btn1*	battenin CLN3 family protein	battenin CLN3 family protein	1.93
SPBC1706.02c	*wtf2*	wtf element Wtf2, pseudo		1.93
SPAC22F3.05c	*alp41*	ADP-ribosylation factor Alp41	ADP-ribosylation factor Alp41	1.93
SPBC776.06c		spindle pole body interacting protein (predicted)		1.93
SPAC1565.01		conserved fungal protein	conserved fungal protein	1.93
SPAC2G11.09		DUF221 family protein	DUF221 family protein	1.93
SPAC13G7.02c	*ssa1*	heat shock protein Ssa1 (predicted)	heat shock protein Ssa1	1.93
SPBC27B12.11c		transcription factor (predicted)		1.93
SPCC622.21	*wtf12*	wtf element Wtf12	wtf element Wtf12	1.93
SPBC660.06		conserved fungal protein	conserved fungal protein	1.93
SPBC3E7.01	*fab1*	1-phosphatidylinositol-3-phosphate 5-kinase Fab1	1-phosphatidylinositol-3-phosphate 5-kinase Fab1 (PMID 10567352)	1.92
SPBC3B8.02	*php5*	CCAAT-binding factor complex subunit Php5		1.92
SPCC645.03c	*isa1*	iron-sulfur protein Isa1	iron-sulfur protein Isa1	1.92
SPAC23C4.13	*bet1*	SNARE Bet1	SNARE Bet1	1.92
SPAC1B3.20		sequence orphan	sequence orphan	1.92
SPAC1093.03		inositol polyphosphate phosphatase (predicted)	polyphosphoinositide phosphatase (predicted)	1.92
SPBC25H2.13c	*cdc20*	DNA polymerase epsilon catalytic subunit a Pol2		1.92
SPBC16D10.04c	*dna2*	DNA replication endonuclease-helicase Dna2		1.92
SPAC1952.17c		GTPase activating protein		1.92
SPBC409.09c	*mis13*	kinetochore protein Mis13	kinetochore protein Mis13	1.91

SPAPB2B4.07		ubiquitin family protein, human UBTD1 homolog	ubiquitin family protein, human UBTD1 homolog	1.91
SPMIT.11	*cox2*	cytochrome c oxidase 2	cytochrome c oxidase 2; similar to S. cerevisiae Q0250	1.91
SPAP19A11.05c	*mrp7*	mitochondrial ribosomal protein subunit L27	mitochondrial ribosomal protein subunit L27	1.91
SPCC63.04	*mok14*	alpha-1,3-glucan synthase Mok14	alpha-1,3-glucan synthase Mok14	1.91
SPAC27F1.08	*pdt1*	Nramp family manganese ion transporter	Nramp family manganese ion transporter	1.91
SPBC3H7.05c		sequence orphan		1.91
SPBC12C2.10c	*pst1*	Clr6 histone deacetylase complex subunit Pst1		1.91
SPAC17G6.04c	*cpp1*	protein farnesyltransferase beta subunit Cpp1	protein farnesyltransferase beta subunit Cpp1	1.91
SPAC2E12.02	*hsf1*	transcription factor Hsf1	transcription factor Hsf1	1.90
SPCC970.01	*rad16*	DNA repair endonuclease XPF	DNA repair endonuclease XPF	1.90
SPAC227.11c		sensor for misfolded ER glycoproteins Yos9 (predicted)	sensor for misfolded ER glycoproteins Yos9 (predicted)	1.90
SPAC9E9.12c	*ybt1*	ABC transporter Ybt1	ABC transporter Ybt1	1.90
SPCC1183.10	*wtf10*	wtf element Wtf10	wtf element Wtf10	1.90
SPACUNK4.10		hydroxyacid dehydrogenase (predicted)	hydroxyacid dehydrogenase (predicted)	1.90
SPAC25G10.09c		actin cortical patch component, with EF hand and WH2 motif (predicted)		1.90
SPCC61.03		UPF0031 family protein	conserved protein (broad species distribution)	1.90
SPCC1682.15	*mug122*	PX/PXA domain protein	PX/PXA domain protein	1.90
SPBC1105.15c	*htd2*	3-hydroxyacyl-ACP dehydratase Htd2 (predicted)		1.89
SPBC19C7.04c		conserved fungal protein		1.89
SPBC405.04c	*ypt7*	GTPase Ypt7		1.89
SPBC17D11.02c		synoviolin homolog		1.89
SPAC1783.07c	*pap1*	transcription factor Caf3	transcription factor Caf3	1.89
SPAC16E8.09	*scd1*	RhoGEF Scd1	RhoGEF Scd1	1.89
SPAPJ696.01c	*vps17*	retromer complex subunit Vps17	retromer complex subunit Vps17	1.89
SPCC622.07		dubious	dubious	1.89
SPAC10F6.09c	*psm3*	mitotic cohesin complex subunit Psm3	mitotic cohesin complex subunit Psm3	1.89
SPAC1952.09c		acetyl-CoA hydrolase	acetyl-CoA hydrolase	1.89
SPAC23A1.16c		DUF408 family protein	DUF408 family protein	1.89

SPAC4G9.14		mitochondrial Mvp17/PMP22 family protein 2	Mvp17/PMP22 family	1.88
SPAC16C9.01c		carbohydrate kinase (predicted)	carbohydrate kinase (predicted)	1.88
SPAC1006.04c	*mcp3*	sequence orphan	sequence orphan	1.88
SPAC1006.03c		human CCDC131 homolog	human CCDC131 homolog	1.88
SPBC1718.06	*msp1*	mitochondrial GTPase Msp1		1.88
SPAC9G1.06c	*cyk3*	cytokinesis protein Cyk3	cytokinesis protein Cyk3	1.87
SPAC458.03	*tel2*	Tel2/Rad-5/Clk-2 family protein Tel2	nuclear telomere cap complex subunit (predicted)	1.87
SPCC320.04c		GTPase Gem1	GTPase Gem1 (PMID 12482879)	1.87
SPBC2D10.13	*est1*	telomerase regulator Est1		1.87
SPBC15D4.13c		sequence orphan		1.87
SPAC19G12.01c	*cut20*	anaphase-promoting complex subunit Apc4	anaphase-promoting complex subunit Apc4	1.87
SPCC11E10.02c	*gpi8*	pig-K	pig-K	1.87
SPAC20H4.02		conserved fungal protein	conserved fungal protein	1.87
SPAC17A5.04c	*mde10*	spore wall assembly peptidase Mde10	spore wall assembly peptidase Mde10	1.87
SPAC343.03	*apc11*	anaphase-promoting complex subunit Apc11	anaphase-promoting complex subunit Apc11	1.87
SPBC12C2.09c		Haemolysin-III family protein		1.87
SPBC106.20	*exo70*	exocyst complex subunit Exo70 (predicted)		1.87
SPBC646.15c		Pex16 family peroxisome import protein		1.87
SPAC6F12.15c	*cut9*	anaphase-promoting complex subunit Cut9	anaphase-promoting complex subunit Cut9	1.87
SPAC11E3.08c	*nse6*	Smc5-6 complex non-SMC subunit Nse6	Smc5-6 complex non-SMC subunit Nse6	1.87
SPAC23A1.17		WIP homolog	WIP homolog	1.87
SPAC5D6.04		auxin family transmembrane transporter (predicted)	auxin family	1.87
SPAC4F10.08	*mug126*	sequence orphan	sequence orphan	1.86
SPAC31G5.18c		ubiquitin family, human C1ORF55 related	ubiquitin family, human C1ORF55 related	1.86
SPBC21B10.02		sequence orphan		1.86
SPAC22A12.08c		cardiolipin synthase/ hydrolase fusion protein (predicted)	cardiolipin synthase (predicted)	1.86
SPBC25H2.08c	*mrs2*	magnesium ion transporter Mrs2	magnesium ion transporter Mrs2	1.86
SPCC1742.01		sequence orphan		1.86
SPBC405.05		sequence orphan		1.86

SPCC550.08		N-acetyltransferase (predicted)	N-acetyltransferase (predicted)	1.86
SPAC23A1.18c	*mrp51*	mitochondrial ribosomal protein subunit L51-b (predicted)	mitochondrial ribosomal protein subunit L51-b (predicted)	1.86
SPBC651.02		nitrilase (predicted)		1.86
SPCC1919.10c	*myo52*	myosin type V	myosin type V	1.86
SPBC216.07c	*tor2*	phosphatidylinositol kinase Tor2		1.86
SPAC630.04c		sequence orphan	sequence orphan	1.85
SPBC24C6.08c		vesicle coat protein		1.85
SPBC28E12.06c	*lvs1*	beige protein homolog		1.85
SPBC8E4.02c		sequence orphan		1.85
SPBC16A3.09c	*ufd1*	Cdc48-Ufd1-Npl4 complex component Ufd1 (predicted)		1.85
SPBC1A4.06c		mitochondrial matrix protein import protein		1.85
SPAC11D3.18c		nicotinic acid plasma membrane transporter (predicted)	nicotinic acid plasma membrane transporter (predicted)	1.85
SPBC691.03c	*apl3*	AP-2 adaptor complex subunit Alp3 (predicted)		1.84
SPAC1556.06b			sequence orphan	1.84
SPAC824.05	*vps16*	HOPS complex subunit Vps16 (predicted)	HOPS complex subunit Vps16 (predicted)	1.84
SPAC12B10.16c	*mug157*	conserved protein (fungal and bacterial)	conserved protein (fungal and bacterial)	1.84
SPAC959.09c	*apc5*	anaphase-promoting complex subunit Apc5	anaphase-promoting complex subunit Apc5 (PMID 12477395)	1.84
SPAC12G12.09		sequence orphan	sequence orphan	1.84
SPAC19G12.08		fatty acid hydroxylase (predicted)	fatty acid hydroxylase (predicted)	1.84
SPAC19G12.11	*coq9*	ubiquinone biosynthesis protein Coq9 (predicted)	ubiquinone biosynthesis protein Coq9 (predicted)	1.84
SPBC29A3.14c	*trt1*	telomerase reverse transcriptase 1 protein Trt1		1.84
SPCC645.06c	*rgf3*	RhoGEF Rgf3	RhoGEF Rgf3	1.84
SPCC70.05c		serine/threonine protein kinase (predicted)	serine/threonine protein kinase (predicted)	1.84
SPAC6G9.06c	*pcp1*	pericentrin Pcp1	pericentrin Pcp1	1.83
SPBC336.08	*spc24*	spindle pole body protein Spc24		1.83
SPBC30D10.10c	*tor1*	phosphatidylinositol kinase Tor1		1.83
SPCC663.06c		short chain dehydrogenase (predicted)	short chain dehydrogenase (predicted)	1.83
SPAC222.13c		6-phosphofructo-2-kinase (predicted)	6-phosphofructo-2-kinase (predicted)	1.83
SPBC18H10.10c	*cwc16*	complexed with Cdc5 protein Cwf16		1.83

SPAC23C4.18c	*rad4*	BRCT domain protein Rad4	BRCT domain protein Rad4	1.83
SPBC32F12.15	*tfb5*	transcription factor TFIIH complex subunit Tfb5 (predicted)	transcription factor TFIIH complex subunit Tfb5 (predicted)	1.83
SPAC1527.02	*sft2*	Golgi transport protein Sft2 (predicted)	Golgi transport protein Sft2 (predicted)	1.83
SPCC1183.03c		frataxin homolog	frataxin homolog	1.83
SPCC970.06		cargo receptor for soluble proteins (predicted)	cargo receptor for soluble proteins (predicted)	1.83
SPBPJ4664.02		glycoprotein (predicted)		1.83
SPAC2C4.07c		ribonuclease II (RNB) family	ribonuclease II (RNB) family	1.83
SPCC1919.12c		aminopeptidase (predicted)	aminopeptidase (predicted)	1.83
SPAC12B10.08c		mitochondrial tRNA(Ile)-lysidine synthetase family (predicted)	tRNA(Ile)-lysidine synthetase family	1.83
SPCC970.04c	*mob2*	protein kinase activator Mob2	protein kinase activator Mob2	1.82
SPBC16G5.07c		prohibitin (predicted)		1.82
SPCPJ732.01	*vps5*	retromer complex subunit Vps5	retromer complex subunit Vps5	1.82
SPAC7D4.05		hydrolase (predicted)	hydrolase (predicted)	1.82
SPAC1805.10		sequence orphan	sequence orphan	1.82
SPAC1635.01		voltage-dependent anion-selective channel	voltage-dependent anion-selective channel	1.82
SPAC23C11.08	*php3*	CCAAT-binding factor complex subunit Php3	CCAAT-binding factor complex subunit Php3 (PMID 8223474)	1.82
SPBC15D4.01c		kinesin-like protein		1.82
SPAPB1A10.02		chromosome segregation protein (predicted)	chromosome segregation protein (predicted)	1.81
SPBC25B2.03		zf-C3HC4 type zinc finger		1.81
SPAC589.02c	*med13*	mediator complex subunit Srb9	mediator complex subunit Srb9	1.81
SPBC1198.12	*mfr1*	fizzy-related protein Mfr1		1.81
SPCC14G10.04		sequence orphan	sequence orphan	1.81
SPCC550.03c		Ski complex RNA helicase Ski2 (predicted)	RNA helicase involved in mRNA catabolism	1.81
SPBC660.08		sequence orphan	sequence orphan	1.81
SPAPYUG7.02c	*sin1*	stress activated MAP kinase interacting protein Sin1	stress activated MAP kinase interacting protein Sin1	1.80
SPBC3D6.10	*apn2*	AP-endonuclease Apn2		1.80
SPBC713.07c		vacuolar polyphosphatase (predicted)		1.80
SPBC21H7.06c		inositol metabolism protein Opi10 (predicted)		1.80
SPCC306.06c		ER membrane protein, BIG1 family (predicted)	ER membrane protein, BIG1 family (predicted)	1.80

SPCC23B6.03c	*tel1*	ATM checkpoint kinase	ATM checkpoint kinase	1.80
SPAC31G5.21		human family 32A homolog	human family 32A homolog	1.80
SPCC320.14		threo-3-hydroxyaspartate ammonia-lyase (predicted)	threo-3-hydroxyaspartate ammonia-lyase (predicted)	1.80
SPAC1565.06c	*spg1*	GTPase Spg1	GTPase Spg1	1.80
SPAC1687.02		CAAX prenyl protease (predicted)	CAAX prenyl protease (predicted)	1.80
SPAC29B12.08		sequence orphan	sequence orphan	1.80
SPCC188.10c		pseudo		1.80
SPAC17H9.20	*psc3*	mitotic cohesin complex, non-SMC subunit Psc3		1.80
SPBC713.08	*tom13*	mitochondrial TOM complex subunit Tom13		1.80
SPCC1919.04		sequence orphan	sequence orphan	1.80
SPAC1250.01	*snf21*	ATP-dependent DNA helicase Snf21	ATP-dependent DNA helicase Snf21	1.79
SPAC23C11.16	*plo1*	Polo kinase Plo1	Polo kinase Plo1	1.79
SPAC1D4.13	*byr1*	MAP kinase kinase Byr1	MAP kinase kinase Byr1	1.79
SPAC212.04c		S. pombe specific DUF999 family protein 1	S. pombe specific DUF999 family protein 1	1.79
SPAC23C4.12	*hhp2*	serine/threonine protein kinase Hhp2	serine/threonine protein kinase Hhp2 (PMID 8026462)	1.79
SPAC29A4.06c		human CCDC55 homolog	human CCDC55 homolog	1.79
SPAC17C9.12		MSP domain	MSP domain	1.78
SPAC3G6.09c	*tps2*	trehalose-phosphate synthase Tps2 (predicted)	trehalose-phosphate synthase Tps2 (predicted)	1.78
SPAC3H5.04	*aar2*	U5 snRNP-associated protein Aar2	U5 snRNP-associated protein Aar2	1.78
SPBC146.12	*coq6*	monooxygenase Coq6		1.78
SPAP8A3.12c		tripeptidylpeptidase (predicted)	tripeptidylpeptidase (predicted)	1.78
SPBC16G5.09		serine carboxypeptidase (predicted)		1.78
SPAC26H5.11		spore wall assembly protein (predicted)	spore wall assembly protein (predicted)	1.78
SPAC11E3.10		VanZ-like family protein	VanZ-like family protein	1.78
SPBC16A3.10		membrane bound O-acyltransferase, MBOAT (predicted)		1.78
SPCC736.11	*ago1*	argonaute	argonaute	1.78
SPCC1393.02c		non-specific DNA binding protein Spt2 (predicted)	non-specific DNA binding protein Spt2 (predicted)	1.78
SPBC26H8.05c		serine/threonine protein phosphatase (predicted)		1.78
SPAC607.07c		sequence orphan	sequence orphan	1.78

SPAC56E4.02c	*alg13*	N-acetylglucosaminyldiphosphodolichol N-acetylglucosaminyltransferase Alg13 (predicted)	N-acetylglucosaminyldiphosphodolichol N-acetylglucosaminyltransferase Alg13 (predicted)	1.78
SPAC23G3.09	*taf4*	transcription factor TFIID complex subunit Taf4 (predicted)	transcription factor TFIID complex subunit Taf4	1.77
SPCC1620.08		succinate-CoA ligase (beta subunit)	succinate-CoA ligase (beta subunit)	1.77
SPAC17C9.06	*sam50*	SAM complex subunit Sam50 (predicted)	SAM complex subunit Sam50 (predicted)	1.77
SPAC57A10.04	*mug10*	sequence orphan	sequence orphan	1.77
SPAC343.04c	*gnr1*	heterotrimeric G protein beta subunit Gnr1	heterotrimeric G protein beta subunit Gnr1	1.76
SPAC23D3.14c	*aah2*	alpha-amylase homolog Aah2	alpha-amylase homolog Aah2	1.76
SPBC1105.08		EMP70 family		1.76
SPAC4G9.04c		cleavage and polyadenylation specificity factor (predicted)	cleavage and polyadenylation specificity factor (predicted)	1.76
SPBC4C3.04c		guanyl-nucleotide exchange factor (predicted)		1.76
SPAC24H6.11c		sulfate transporter (predicted)	sulfate transporter (predicted)	1.76
SPBC20F10.10		cyclin pho85 family		1.76
SPBC337.16	*cho1*	phosphatidyl-N-methylethanolamine N-methyltransferase (predicted)		1.76
SPCC23B6.01c		oxysterol binding protein (predicted)	oxysterol binding protein (predicted)	1.76
SPAPYUK71.03c		C2 domain protein	C2 domain protein	1.76
SPCC1840.12		OPT oligopeptide transporter family	OPT oligopeptide transporter family	1.76
SPBC651.03c	*gyp10*	GTPase activating protein Gyp10		1.76
SPAC1782.03		microfibrillar-associated protein family protein	microfibrillar-associated protein familt protein	1.76
SPCC757.04		transcription factor (predicted)	transcription factor (predicted)	1.76
SPBC543.04		UPF0171 family protein		1.76
SPAPB1A11.01		membrane transporter	membrane transporter	1.76
SPAC1B3.15c		membrane transporter	membrane transporter	1.76
SPCC16C4.09	*sts5*	RNB-like protein	RNB-like protein	1.76
SPAC6B12.02c	*mus7*	DNA repair protein Mus7/Mms22	DNA repair protein Mus7	1.75
SPBC11G11.01	*fis1*	mitochondrial fission protein Fis1 (predicted)	mitochondrial fission protein Fis1 (predicted)	1.75
SPBC582.04c		sequence orphan	sequence orphan	1.75
SPAC144.05		ATP-dependent DNA helicase (predicted)	ATP-dependent DNA helicase	1.75

SPAC4A8.09c	*cwf21*	complexed with Cdc5 protein Cwf21	complexed with Cdc5 protein Cwf21 (PMID 11884590)	1.75
SPBC1289.15		glycoprotein (predicted)		1.75
SPAC1F5.04c	*cdc12*	formin Cdc12	formin Cdc12	1.75
SPCP20C8.03		pseudogene (predicted)		1.75
SPAC12B10.03		WD repeat protein, human WDR20 family	WD repeat protein, human WDR20 family	1.75
SPBC1709.12	*rid1*	GTPase binding protein Rid1		1.75
SPCC4B3.15	*mid1*	medial ring protein Mid1	medial ring protein Mid1	1.75
SPBC354.13	*rga6*	GTPase activating protein Rga6	GTPase activating protein Rga6	1.75
SPBP8B7.28c		sequence orphan		1.75
SPBC19F8.04c		nuclease		1.75
SPCC1223.13	*cbf12*	CBF1/Su(H)/LAG-1 family transcription factor Cbf12	transcription factor (predicted)	1.75
SPBC8E4.01c		inorganic phosphate transporter (predicted)		1.75
SPBC21D10.09c		ubiquitin-protein ligase E3 (predicted)		1.74
SPAC23H3.03c		nitrogen permease regulator family	nitrogen permease regulator family	1.74
SPBC776.10c	*cog6*	Golgi transport complex peripheral subunit Cog6 (predicted)		1.74
SPAC3C7.09	*set8*	lysine methyltransferase Set8 (predicted)	lysine methyltransferase Set8 (predicted)	1.74
SPBC29A10.04	*psm1*	mitotic cohesin complex subunit Psm1		1.74
SPCC330.02	*rhp7*	Rad7 homolog Rhp7		1.74
SPCC777.11		sequence orphan	sequence orphan	1.74
SPCC417.02	*dad5*	DASH complex subunit Dad5	DASH complex subunit Dad5	1.74
SPCC1494.08c		conserved fungal protein	conserved fungal protein	1.74
SPAC1420.01c		DUF1752 family protein	DUF1752 family protein	1.73
SPCC126.07c		human CTD-binding SR-like protein rA9 homolog	ubiquitin-protein ligase E3 (predicted)	1.73
SPBC12C2.02c	*ste20*	sterility protein Ste20		1.73
SPCC320.06		sequence orphan	sequence orphan	1.73
SPAC22E12.14c	*sck2*	serine/threonine protein kinase Sck2	serine/threonine protein kinase Sck2	1.73
SPBC27B12.12c		CorA family magnesium ion transporter (predicted)		1.73
SPBC16E9.03c		DUF1783 family protein		1.73
SPAC3A11.14c	*pkl1*	kinesin-like protein Pkl1	kinesin-like protein Pkl1	1.73
SPCP31B10.02		conserved eukaryotic protein	conserved eukaryotic protein	1.73

SPCC1620.09c	*tfg1*	transcription factor TFIIF complex alpha subunit Tfg1 (predicted)	transcription factor TFIIF complex alpha subunit Tfg1	1.73
SPCC548.05c		ubiquitin-protein ligase E3 (predicted)	ubiquitin-protein ligase E3 (predicted)	1.73
SPAC1039.05c	*klf1*	conserved fungal protein	conserved fungal protein	1.73
SPCPB16A4.02c		conserved fungal protein	conserved fungal protein	1.73
SPCC132.05c		trichothecene 3-O-acetyltransferase pseudogene		1.73
SPCC1494.09c		sequence orphan	sequence orphan	1.73
SPBC660.13c	*ssb1*	DNA replication factor A subunit Ssb1	DNA replication factor A subunit Ssb1 (PMID 9111307) (PMID 8702843)	1.73
SPBC16H5.13		WD repeat protein, human WDR7 ortholog		1.72
SPCC18.01c	*adg3*	beta-glucosidase Adg3 (predicted)	beta-glucosidase Adg3 (predicted)	1.72
SPBC21C3.01c	*vps13a*	chorein homolog	chorein homolog	1.72
SPBC887.18c		SAGA complex subunit (predicted)		1.72
SPBC12D12.04c	*pck2*	protein kinase C (PKC)-like Pck2		1.72
SPAC17D4.03c	*cis4*	cation diffusion family zinc membrane transporter Cis4	membrane transporter (predicted)	1.72
SPBC776.15c		dihydrolipoamide S-succinyltransferase, e2 component of oxoglutarate dehydrogenase complex (predicted)		1.72
SPBC36B7.09	*gcn2*	eIF2 alpha kinase Gcn2 (predicted)	eIF2 alpha kinase Gcn2 (predicted)	1.72
SPCC126.02c	*pku70*	Ku domain protein Pku70	Ku domain protein Pku70	1.72
SPAC2G11.14	*taf111*	transcription factor TFIID complex subunit Taf111	transcription factor TFIID complex subunit Taf111	1.72
SPBC21H7.03c		acid phosphatase (predicted)		1.72
SPAC23C4.11	*atp18*	F0-ATPase subunit J (predicted)	F-0 ATPase subunit J (predicted)	1.72
SPAC343.12	*rds1*	conserved fungal protein	conserved fungal protein	1.72
SPAC926.03	*rlc1*	myosin II regulatory light chain	myosin II regulatory light chain (PMID 11056543)	1.72
SPAC19G12.07c	*rsd1*	RNA-binding protein Rsd1	RNA-binding protein Rsd1	1.72
SPBC800.02	*whi5*	cell cycle transcriptional repressor Whi5	cell cycle transcriptional repressor Whi5	1.72
SPAC1687.12c	*coq4*	ubiquinone biosynthesis protein Coq4 (predicted)	ubiquinone biosynthesis protein Coq4	1.71
SPAC343.19		phosphatidylinositol 4-kinase Lsb6 (predicted)		1.71
SPBC23G7.08c	*rga7*	GTPase activating protein Rga7		1.71
SPBC17F3.01c	*rga5*	GTPase activating protein Rga5		1.71

SPAC630.09c	*mug58*	glycerate kinase (predicted)	glycerate kinase (predicted)	1.71
SPBC3D6.13c		protein disulfide isomerase (predicted)		1.71
SPBC685.05	*gpi15*	pig-H (predicted)		1.71
SPAC1296.01c		phosphoacetylglucosamine mutase (predicted)	phosphoacetylglucosamine mutase (predicted)	1.71
SPAC29B12.07	*sec16*	multidomain vesicle coat component Sec16	multidomain vesicle coat component Sec16	1.71
SPCC584.03c		RanGTP-binding protein (predicted)	RanGTP-binding protein (predicted)	1.71
SPAC11D3.01c		conserved fungal protein	conserved fungal protein	1.71
SPAC12G12.03	*cip2*	RNA-binding protein Cip2	RNA-binding protein Cip2	1.71
SPAC9G1.07		sequence orphan	sequence orphan	1.71
SPCC4E9.02	*cig1*	cyclin Cig1		1.71
SPAC1A6.08c	*mug125*	sequence orphan	sequence orphan	1.71
SPBP23A10.13	*orc4*	origin recognition complex subunit Orc4		1.70
SPBC30D10.09c		HVA22/TB2/DP1 family protein		1.70
SPCC1494.10		transcription factor (predicted)		1.70
SPAC3A12.17c	*cys12*	cysteine synthase Cys12	cysteine synthase Cys12	1.70
SPCC1183.01	*sec15*	exocyst complex subunit Sec15 (predicted)	exocyst complex subunit Sec15 (predicted)	1.70
SPAC821.13c		P-type ATPase		1.70
SPAC5H10.01		DUF1445 family protein	DUF1445 family protein	1.70
SPAC16E8.17c		succinate-CoA ligase alpha subunit	succinate-CoA ligase (alpha subunit)	1.70
SPAC3A12.06c		sodium/calcium exchanger (predicted)	sodium/calcium exchanger (predicted)	1.70
SPAC17C9.16c		MFS family transmembrane transporter Mfs1	MFS family transmembrane transporter Mfs1	1.70
SPAC688.10	*rev3*	DNA polymerase zeta catalytic subunit Rev3	DNA polymerase zeta catalytic subunit Rev3	1.70
SPBC1347.10	*cdc23*	MCM-associated protein Mcm10		1.70
SPAC17A5.11	*rec12*	endonuclease Rec12	endonuclease Rec12	1.70
SPBC543.03c	*pku80*	Ku domain protein Pku80		1.70
SPAC9.08c		steroid reductase (predicted)	steroid reductase (predicted)	1.70
SPCC364.04c		CASP family protein	CASP family protein	1.69
SPAC3G9.12	*peg1*	CLASP family microtubule-associated protein	CLASP family microtubule-associated protein	1.69
SPBC3H7.13		FHA domain protein Far10 (predicted)		1.69
SPBC11C11.03	*ndc80*	spindle pole body protein Ndc80		1.69
SPAC22F3.02	*atf31*	transcription factor Atf31	transcription factor Atf31	1.69
SPAC6F6.13c		DUF726 family protein	DUF726 family protein	1.69

SPBC23E6.02		ATP-dependent DNA helicase (predicted)		1.69
SPBC16G5.16		transcription factor, zf-fungal binuclear cluster type (predicted)		1.69
SPAC18B11.05	*gpi18*	pig-V (predicted)	pig-V	1.69
SPAC29B12.01	*ino80*	SNF2 family helicase Ino80	SNF2 family helicase Ino80	1.69
SPBC32F12.07c		ubiquitin-protein ligase E3 (predicted)		1.69
SPAC25G10.07c	*cut7*	kinesin-like protein Cut7	kinesin-like protein Cut7	1.69
SPCC970.08		inositol polyphosphate kinase (predicted)	inositol polyphosphate kinase (predicted)	1.69
SPAC7D4.09c		steroid dehydrogenase (predicted)	steroid dehydrogenase (predicted)	1.68
SPAC26A3.09c	*rga2*	GTPase activating protein Rga2	GTPase activating protein Rga2	1.68
SPAC1D4.02c		human GRASP protein homolog (predicted)	human GRASP protein homolog (predicted)	1.68
SPCC1739.10	*mug33*	conserved fungal protein	conserved fungal protein	1.68
SPAC922.07c		aldehyde dehydrogenase (predicted)	aldehyde dehydrogenase (predicted)	1.68
SPAC1639.02c	*trk2*	potassium ion transporter Trk2		1.68
SPCC1620.13		phosphoglycerate mutase family	phosphoglycerate mutase family	1.68
SPCC550.12	*arp6*	actin-like protein Arp6	actin-like protein Arp6	1.68
SPAC637.13c		cytoskeletal signaling protein	cytoskeletal signaling protein	1.68
SPBC29A3.17	*gef3*	RhoGEF Gef3		1.68
SPAC6B12.07c		ubiquitin-protein ligase E3 (predicted)	ubiquitin-protein ligase E3 (predicted)	1.68
SPBC20F10.05		DuF1740 family protein		1.68
SPBC3B8.04c		membrane transporter		1.68
SPAC31G5.15		phosphatidylserine decarboxylase (predicted)	phosphatidylserine decarboxylase (predicted)	1.68
SPBP35G2.12		nucleoside diphosphate-sugar hydrolase (predicted)		1.68
SPAC1834.11c	*sec18*	secretory pathway protein Sec18 (predicted)	secretory pathway protein Sec18 (predicted)	1.68
SPAC1687.09		ENTH domain protein (predicted)	conserved fungal protein	1.68
SPAC15A10.06		CPA1 sodium ion/proton antiporter	CPA1 sodium ion/proton antiporter	1.67
SPBC19F8.03c		clathrin binding protein		1.67
SPBC1706.03	*fzo1*	mitochondrial fusion GTPase protein		1.67
SPBC3E7.08c	*rad13*	DNA repair nuclease Rad13		1.67
SPBC1683.02		adenine deaminase (predicted)	adenine deaminase (predicted)	1.67
SPBC317.01	*mbx2*	MADS-box transcription factor Pvg4		1.67
SPCC417.03		sequence orphan	sequence orphan	1.67

SPAC1B9.02c	*sck1*	serine/threonine protein kinase Sck1	serine/threonine protein kinase Sck1	1.67
SPAC16E8.16		transcription factor TFIIB	transcription factor TFIIB (PMID 12359329)	1.67
SPBC1826.01c	*mot1*	TATA-binding protein associated factor Mot1	TATA-binding protein associated factor Mot1	1.67
SPAC22F3.09c	*res2*	MBF transcription factor complex subunit Res2	MBF transcription factor complex subunit Res2	1.67
SPCC338.08	*ctp1*	CtIP-related endonuclease	sequence orphan	1.67
SPAC13G6.03	*gpi7*	GPI anchor biosynthesis protein Gpi7 (predicted)	GPI anchor biosynthesis protein Gpi7 (predicted)	1.67
SPCC777.02		transcription factor (predicted)	transcription factor (predicted)	1.66
SPAC16A10.03c		zinc finger protein Pep5/Vps11 (predicted)	zinc finger protein Pep5/Vps11 (predicted)	1.66
SPAC57A10.06	*mug15*	sequence orphan	sequence orphan	1.66
SPBC28F2.12	*rpb1*	DNA-directed RNA polymerase II large subunit		1.66
SPBC146.01	*med15*	mediator complex subunit Med15 (predicted)	mediator complex subunit Med15 (predicted)	1.66
SPBC428.20c	*alp6*	gamma tubulin complex Spc98/GCP3 subunit Alp6		1.66
SPAC869.01		amidase (predicted)	amidase (predicted)	1.66
SPBC1539.02		sequence orphan		1.66
SPAPJ760.02c	*app1*	App1 protein	App1 protein	1.65
SPBC776.18c	*pmh1*	transcription factor TFIIH complex subunit Pmh1		1.65
SPAC6C3.06c		P-type ATPase, calcium transporting	P-type ATPase, calcium transporting (PMID 12707717)	1.65
SPBC29A3.01		heavy metal ATPase		1.65
SPCC1322.14c	*vtc4*	vacuolar transporter chaperone (VTC) complex subunit (predicted)	vacuolar transporter chaperone (VTC) complex subunit (predicted)	1.65
SPBC27B12.08		AP-1 accessory protein (predicted)		1.65
SPAC1039.06		alanine racemase (predicted)	alanine racemase (predicted)	1.65
SPBC21C3.12c		DUF953 family protein		1.65
SPBC649.05	*cut12*	spindle pole body protein Cut12	spindle pole body protein Cut12 (PMID 9531532)	1.65
SPAC1A6.07		sequence orphan	sequence orphan	1.65
SPAC4G9.05	*mpf1*	meiotic PUF family protein 1	meiotic PUF family protein 1	1.65
SPAC27E2.01		alpha-amylase homolog (predicted)	alpha-amylase homolog (predicted)	1.65
SPCC70.10		sequence orphan	sequence orphan	1.65

SPAC6C3.04	*cit1*	citrate synthase (predicted)	citrate synthase	1.65
SPCC1672.08c	*tfa2*	transcription factor TFIIE beta subunit Tfa2	transcription factor TFIIE beta subunit Tfa2	1.65
SPAC17A5.05c		conserved fungal protein	conserved fungal protein	1.65
SPAC14C4.04	*B22918-2*	hypothetical protein	hypothetical protein	1.65
SPCC1450.01c		pseudogene		1.65
SPAC644.08		haloacid dehalogenase-like hydrolase	haloacid dehalogenase-like hydrolase	1.65
SPAC144.17c		6-phosphofructo-2-kinase	6-phosphofructo-2-kinase	1.65
SPAC27E2.06c		methionine-tRNA ligase, mitochondrial	methionine-tRNA ligase	1.65
SPBC26H8.02c	*sec9*	SNAP-25 homologue, t-SNARE component Sec9		1.65
SPBCPT2R1.06c		pseudogene		1.64
SPAC1952.10c		conserved fungal protein	conserved fungal protein (predicted)	1.64
SPBC342.05	*crb2*	DNA repair protein RAD9 homolog, Rhp9		1.64
SPAC1783.02c	*vps66*	acyltransferase (predicted)	acyltransferase (predicted)	1.64
SPBC12C2.08	*dnm1*	dynamin Dnm1	dynamin Dnm1	1.64
SPBC16A3.19		histone acetyltransferase complex subunit Eaf7 (predicted)		1.64
SPAPB2C8.01		glycoprotein (predicted)	glycoprotein (predicted)	1.64
SPAC22H10.03c	*kap114*	karyopherin Kap14	karyopherin Kap14	1.64
SPAC11D3.10		nifs homolog	nifs homolog	1.64
SPBC1773.13		aromatic aminotransferase (predicted)	aromatic aminotransferase (predicted)	1.64
SPCC74.03c	*ssp2*	serine/threonine protein kinase Ssp2	serine/threonine protein kinase Ssp2	1.64
SPAC29E6.05c		peptide methionine sulfoxide reductase (predicted)	peptide methionine sulfoxide reductase (predicted)	1.64
SPAC2F7.08c	*snf5*	chromatin remodeling complex subunit Snf5 (predicted)	chromatin remodeling complex subunit Snf5 (predicted)	1.63
SPBP26C9.03c	*fet4*	iron/zinc ion transporter (predicted)	iron ion transporter (predicted)	1.63
SPBC21B10.13c		transcription factor, homeobox type (predicted)		1.63
SPAC2F3.14c		conserved fungal protein	conserved fungal protein	1.63
SPCC5E4.10c		sequence orphan	sequence orphan	1.63
SPAC30D11.09	*cwf19*	complexed with Cdc5 protein Cwf19	complexed with Cdc5 protein Cwf19 (PMID 11884590)	1.63
SPCC1183.04c	*pet127*	mitochondrial membrane protein Pet127	mitochondrial membrane protein Pet127	1.63
SPAC57A7.11	*mip1*	WD repeat protein Mip1	WD repeat protein Mip1	1.63
SPCC1795.01c	*mad3*	mitotic spindle checkpoint protein Mad3	mitotic spindle checkpoint protein Mad3	1.63

SPCC622.10c		exocyst complex subunit Sec5 (predicted)	exocyst complex subunit Sec5 (predicted)	1.63
SPAC222.11	*hem13*	coproporphyrinogen III oxidase (predicted)	coproporphyrinogen III oxidase (predicted)	1.62
SPAC1B3.04c		mitochondrial GTPase Guf1 (predicted)	mitochondrial GTPase Guf1 (predicted)	1.62
SPBC337.06c	*cwf15*	complexed with Cdc5 protein Cwf15		1.62
SPBC1921.06c	*pvg3*	beta-1,3-galactosyltransferase		1.62
SPAC4F8.10c	*stg1*	SM22/transgelin-like actin modulating protein Stg1	SM22/transgelin-like actin modulating protein Stg1	1.62
SPAC1327.01c		transcription factor, zf-fungal binuclear cluster type (predicted)	transcription factor (predicted)	1.62
SPBC106.09	*cut4*	anaphase-promoting complex subunit Apc1	anaphase-promoting complex subunit Apc1	1.62
SPBC30D10.03c		IMP 5'-nucleotidase (predicted)		1.62
SPCC736.04c	*gma12*	alpha-1,2-galactosyltransferase Gma12	alpha-1,2-galactosyltransferase Gma12 (PMID 95003210)	1.62
SPCC1919.02		pig-X	pig-X	1.62
SPBC31F10.13c	*hip1*	hira protein Hip1		1.62
SPBC25B2.11	*pof2*	F-box protein Pof2		1.62
SPAC20H4.09		ATP-dependent RNA helicase, spliceosomal (predicted)	ATP-dependent RNA helicase, spliceosomal (predicted)	1.62
SPBC17A3.03c		phosphoprotein phosphatase (predicted)		1.62
SPCC1620.07c		lunapark homolog	lunapark homolog	1.62
SPAC110.01	*ppk1*	serine/threonine protein kinase Ppk1 (predicted)	serine/threonine protein kinase Ppk1 (predicted)	1.62
SPBC215.01		GTPase activating protein		1.62
SPAC139.03		transcription factor, zf-fungal binuclear cluster type (predicted)	transcription factor (predicted)	1.61
SPBC18E5.10		iron sulfur cluster assembly protein (predicted)		1.61
SPMIT.10	*atp9*	F0-ATPase subunit 9	F0-ATPase subunit 9; similar to S. cerevisiae Q0130	1.61
SPBC18E5.14c		sequence orphan		1.61
SPAC26H5.05		IPT/TIG ankyrin repeat protein	IPT/TIG ankyrin repeat protein	1.61
SPCC18.17c		sequence orphan	sequence orphan	1.61
SPCC4G3.07c	*phf1*	PHD finger containing protein Phf1	PHD finger containing protein Phf1	1.61
SPBC428.12c		RNA-binding protein	RNA-binding protein	1.61
SPAC823.05c	*tlg2*	SNARE Tlg2 (predicted)	SNARE Tlg2	1.61

SPBC2G2.09c	*crs1*	meiosis specific cyclin Crs1		1.61
SPBC2D10.04		arrestin Aly1 related		1.61
SPAC30D11.13	*hus5*	SUMO conjugating enzyme Hus5	SUMO conjugating enzyme Hus5	1.61
SPBC800.11		inosine-uridine preferring nucleoside hydrolase (predicted)	inosine-uridine preferring nucleoside hydrolase (predicted)	1.61
SPAC1002.16c		nicotinic acid plasma membrane transporter (predicted)	nicotinic acid plasma membrane transporter (predicted)	1.61
SPAC1687.15	*gsk3*	serine/threonine protein kinase Gsk3	serine/threonine protein kinase Gsk3	1.61
SPBP4H10.16c		phosphatase activator (predicted)		1.61
SPAC12G12.15	*sif3*	Sad1 interacting factor 3	Sad1 interacting factor 3	1.61
SPBC2F12.08c	*ceg1*	mRNA guanylyltransferase Ceg1		1.61
SPAC15E1.04		thymidylate synthase (predicted)	thymidylate synthase (predicted)	1.61
SPBC1198.10c		asparagine-tRNA ligase Slm5	asparagine-tRNA ligase Slm5	1.61
SPAC1296.05c		cyclin L family cyclin	cyclin L family cyclin	1.61
SPAC1556.08c	*cbs2*	protein kinase activator (predicted)		1.61
SPBC16D10.06	*zrt1*	ZIP zinc transporter Zrt1		1.60
SPAC19B12.07c		human ZNF277P homolog	human ZNF277P homolog	1.60
SPAC19G12.02c	*pms1*	MutL family mismatch-repair protein Pms1	MutL family mismatch-repair protein Pms1	1.60
SPAC57A7.13		RNA-binding protein	RNA-binding protein	1.60
SPBC19C7.02	*ubr1*	N-end-recognizing protein Ubr1		1.60
SPBC11B10.06	*sws1*	SWIM domain containing-Srs2 interacting protein 1		1.60
SPAC3H1.12c	*snt2*	Lid2 complex subunit Snt2	Lid2 complex subunit Snt2 (PMID 12488447)	1.60
SPBC21C3.19		DUF1960 family protein		1.60
SPBC19C7.11		ClC chloride channel (predicted)		1.60
SPCC965.11c		amino acid transporter (predicted)	amino acid transporter (predicted)	1.60
SPAC823.09c		L-asparaginase (predicted)	L-asparaginase (predicted)	1.60
SPBC428.08c	*clr4*	histone H3 methyltransferase Clr4	histone H3 methyltransferase Clr4	1.60
SPBC365.02c	*cox10*	protoheme IX farnesyltransferase		1.60
SPCC790.02	*pep3*	ubiquitin-protein ligase E3 (predicted)	ubiquitin-protein ligase E3 (predicted)	1.60
SPAC222.15	*meu13*	Tat binding protein 1(TBP-1)-interacting protein (TBPIP) homolog (predicted)		1.59
SPAC959.05c		protein disulfide isomerase (predicted)	protein disulfide isomerase (predicted)	1.59
SPCC162.07	*ent1*	epsin	epsin	1.59
SPAC3H5.09c		conserved fungal protein	conserved fungal protein	1.59

SPBC3E7.12c	*chr1*	chitin synthase regulatory factor (putative) Chr1		1.59
SPBC685.02		conserved eukaryotic protein		1.59
SPAC4F8.01	*did4*	vacuolar sorting protein Did4		1.59
SPCC1450.09c		phospholipase (predicted)	phospholipase (predicted)	1.59
SPBC15D4.08c		dubious		1.59
SPAC186.02c		hydroxyacid dehydrogenase (predicted)	hydroxyacid dehydrogenase (predicted)	1.59
SPCP1E11.05c		sterol O-acyltransferase (predicted)	sterol O-acyltransferase (predicted)	1.59
SPAC1486.02c	*ucp14*	UBA domain protein Ucp14	UBA domain protein Ucp14	1.59
SPAC19A8.12	*dcp2*	mRNA decapping complex subunit Dcp2	mRNA decapping complex subunit Dcp2	1.59
SPAC23H3.02c	*ini1*	RING finger-like protein Ini1	RING finger-like protein Ini1	1.58
SPCC663.15c		conserved fungal protein	conserved fungal protein	1.58
SPAC2H10.01		transcription factor, zf-fungal binuclear cluster type (predicted)	transcription factor	1.58
SPCC1020.12c		xap-5-like protein	xap-5-like protein	1.58
SPCC777.03c		nifs homolog	nifs homolog	1.58
SPAPB24D3.03		agmatinase (predicted)	agmatinase (predicted)	1.58
SPCC1235.07	*fta7*	Sim4 and Mal2 associated (4 and 2 associated) protein 7	Sim4 and Mal2 associated (4 and 2 associated) protein 7 (PMID 16079914)	1.58
SPBC8D2.03c	*hhf2*	histone H4 h4.2		1.58
SPCC1259.15c	*ubc11*	ubiquitin conjugating enzyme E2-C	ubiquitin conjugating enzyme E2-C (PMID 12724408)	1.58
SPBC365.15	*alp4*	gamma tubulin complex Spc97/GCP2 subunit Alp4		1.58
SPBC32F12.08c	*duo1*	DASH complex subunit Duo1 (predicted)		1.58
SPAC17A2.05	*osm1*	fumerate reductase	fumerate reductase	1.58
SPBC56F2.01	*pof12*	F-box protein Pof12		1.58
SPBC83.03c	*tas3*	RITS complex subunit 3		1.58
SPBP35G2.09	*usp103*	U1 snRNP-associated protein Usp103 (predicted)		1.58
SPCC23B6.04c		sec14 cytosolic factor family	sec14 cytosolic factor family	1.58
SPBC725.13c	*psf2*	GINS complex subunit Psf2		1.58
SPAC26F1.07		2-methylbutyraldehyde reductase (predicted)	2-methylbutyraldehyde reductase (predicted)	1.58
SPAC25B8.11		transcription factor (predicted)	transcription factor	1.58
SPAC7D4.03c		conserved fungal family	conserved fungal family	1.58
SPAC22E12.03c		THIJ/PFPI family peptidase (predicted)	THIJ/PFPI family peptidase (predicted)	1.57

SPBP23A10.12		FRG1 family protein		1.57
SPAC589.11	*mug82*	mitochondrial translation release factor (predicted)	translation release factor (predicted)	1.57
SPAC3C7.07c		arginine-tRNA protein transferase (predicted)	arginine-tRNA protein transferase (predicted)	1.57
SPCC794.11c		ENTH domain protein Ent3	ENTH domain protein Ent3	1.57
SPBC800.03	*clr3*	histone deacetylase (class II) Clr3	histone deacetylase (class II) Clr3	1.57
SPCC16C4.02c		DUF1941 family protein	DUF1941 family protein	1.57
SPBC4C3.06		actin cytoskeletal protein Syp1		1.57
SPAC20H4.10	*ufd2*	ubiquitin-protein ligase E4 (predicted)	ubiquitin-protein ligase E4 (predicted)	1.57
SPAC24H6.06	*sld3*	DNA replication pre-initiation complex subunit Sld3	pre-initiation complex subunit Sld3 (PMID 12006645)	1.57
SPAC29E6.10c		kinetochore protein (predicted)	kinetochore protein (predicted)	1.57
SPCC895.04c	*ufe1*	SNARE Ufe1	SNARE Ufe1	1.57
SPBC15D4.12c	*mug98*	sequence orphan		1.56
SPAC1834.08	*mak1*	histidine kinase Mak1	histidine kinase Mak1	1.56
SPBC3F6.05	*rga1*	GTPase activating protein Rga1		1.56
SPAC22E12.19		histone deacetylase complex subunit (predicted)	histone deacetylase complex subunit (predicted)	1.56
SPAC4F8.11		WD repeat protein, human WDR24 family	WD repeat protein, human WDR24 family	1.56
SPCC1827.08c	*pof7*	F-box protein Pof7	F-box protein Pof7	1.56
SPCC24B10.16c		sequence orphan	sequence orphan	1.56
SPAC664.07c	*rad9*	checkpoint clamp complex protein Rad9	checkpoint clamp complex protein Rad9	1.56
SPAC1002.18	*urg3*	DUF1688 family protein	DUF1688 family protein	1.56
SPBC337.07c		carboxypeptidase (predicted)		1.56
SPBC1861.09	*ppk22*	serine/threonine protein kinase Ppk22 (predicted)		1.56
SPBC83.09c		GYF domain		1.56
SPAC3G9.07c	*hos2*	histone deacetylase (class I) Hos2	histone deacetylase (class I) Hos2	1.56
SPBC215.03c	*csn1*	COP9/signalosome complex subunit Csn1		1.56
SPCC576.05		nucear export factor	nucear export factor	1.56
SPAC589.08c	*dam1*	DASH complex subunit Dam1	DASH complex subunit Dam1	1.56
SPBC887.09c		leucine-rich repeat protein Sog2 (predicted)		1.56
SPAC57A10.09c		High-mobility group non-histone chromatin protein	High-mobility group non-histone chromatin protein	1.56

SPAC110.02	*pds5*	cohesin-associated protein Pds5	cohesin-associated protein Pds5	1.56
SPBC1348.11		membrane transporter	membrane transporter	1.55
SPCC4B3.04c	*nte1*	lysophospholipase	lysophospholipase	1.55
SPAC10F6.05c	*ubc6*	ubiquitin conjugating enzyme Ubc6	ubiquitin conjugating enzyme Ubc6	1.55
SPAC343.17c		WD repeat protein, human WDR70 family	WD repeat protein, human WDR70 family	1.55
SPBC1215.01	*shy1*	SURF-family protein Shy1	SURF-family protein Shy1	1.55
SPBC1A4.04		sequence orphan		1.55
SPAC3G6.05		Mvp17/PMP22 family protein 1	Mvp17/PMP22 family	1.55
SPAC22H10.11c				1.55
SPAC806.04c		DUF89 family protein	DUF89 family protein	1.54
SPCC663.03	*pmd1*	leptomycin efflux transporter Pmd1	leptomycin efflux transporter Pmd1	1.54
SPAPB1A10.12c	*alo1*	D-arabinono-1,4-lactone oxidase	D-arabinono-1,4-lactone oxidase	1.54
SPCC594.07c		sequence orphan	sequence orphan	1.54
SPAC24H6.09	*gef1*	RhoGEF Gef1	RhoGEF Gef1	1.54
SPCC584.05	*sec1*	SNARE binding protein Sec1	SNARE binding protein Sec1	1.54
SPAC13C5.07	*rad32*	Rad32 nuclease	Rad32 nuclease	1.54
SPCC16C4.05		RNase P and RNase MRP subunit (predicted)	RNase P and RNase MRP subunit (predicted)	1.54
SPBC106.03		DUF1776 family protein	DUF1776 family protein	1.54
SPCC757.13		membrane transporter (predicted)	membrane transporter (predicted)	1.54
SPCC1827.02c		cholinephosphate cytidylyltransferase (predicted)	cholinephosphate cytidylyltransferase (predicted)	1.54
SPCC1235.03		SMR and CUE domain protein	SMR and CUE domain protein	1.54
SPAC1F3.09	*mug161*	CwfJ family protein	CwfJ family protein	1.54
SPAC9G1.02	*wis4*	MAP kinase kinase kinase Wis4	MAP kinase kinase kinase Wis4	1.53
SPBC1105.13c		sequence orphan		1.53
SPAC17H9.18c		dubious	dubious	1.53
SPAC6G9.15c		sequence orphan	sequence orphan	1.53
SPBP4H10.19c		calreticulin/calnexin homolog		1.53
SPAP8A3.05		ski complex interacting GTPase (predicted)	ski complex subunit Ski7 (predicted)	1.53
SPBC1921.07c		SAGA complex subunit Sgf29 (predicted)		1.53
SPCC645.13		transcription elongation regulator	transcription elongation regulator	1.53
SPAC2G11.12	*rqh1*	RecQ type DNA helicase Rqh1	RecQ type DNA helicase Rqh1	1.53
SPBC2D10.18	*abc1*	ABC1 kinase family protein		1.53
SPAC630.11	*vps55*	vacuolar sorting protein Vps55 (predicted)	vacuolar sorting protein Vps55 (predicted)	1.52

SPAC2F3.15	*lsk1*	latrunculin sensitive kinase Lsk1	latrunculin sensitive kinase Lsk1 (PMID 15537703)	1.52
SPAC3H1.08c		DUF1640 family protein	DUF1640 family protein	1.52
SPAC1782.01		proteasome component	proteasome component	1.52
SPCC162.08c	*nup211*	nuclear pore complex associated protein	nuclear pore complex associated protein	1.52
SPCC1235.10c	*sec6*	exocyst complex subunit Sec6	exocyst complex subunit Sec6	1.52
SPBC1685.07c		amino acid transporter (predicted)	amino acid transporter (predicted)	1.52
SPAC1002.02	*mug31*	nucleoporin Pom34 (predicted)	nucleoporin Pom34 (predicted)	1.52
SPBC428.16c	*rhb1*	Rheb GTPase Rhb1	Rheb GTPase Rhb1	1.52
SPCC1840.01c	*mog1*	Ran GTPase binding protein Mog1		1.52
SPBC14F5.13c		alkaline phosphatase (predicted)		1.52
SPCC1442.17c		DUF292 family protein	DUF292 family protein	1.52
SPAC644.11c		mitochondrial pyruvate dehydrogenase (lipoamide) kinase	pyruvate dehydrogenase (lipoamide) kinase	1.52
SPAC20H4.06c		RNA-binding protein	RNA-binding protein	1.52
SPBC530.11c		transcription factor (predicted)		1.52
SPAC26A3.14c		DUF1748 family protein	DUF1748 family protein	1.52
SPCC622.06c		dubious	dubious	1.52
SPAC12G12.12		NST UDP-galactose transporter (predicted)	NST UDP-galactose transporter	1.52
SPCC364.02c	*bis1*	stress response protein Bis1	stress response protein Bis1	1.52
SPAC1565.02c		GTPase activating protein	GTPase activating protein	1.52
SPBC582.06c	*mcp6*	horsetail movement protein Hrs1/Mcp6	meiosis specific coiled-coil protein Mcp6	1.52
SPAC1A6.01c		human thyroid receptor interacting protein homolog	human thyroid receptor interacting protein homolog	1.51
SPBC2A9.02		NAD dependent epimerase/dehydratase family protein		1.51
SPAP27G11.14c		sequence orphan	sequence orphan	1.51
SPCC830.05c	*epl1*	histone acetyltransferase complex subunit Epl1 (predicted)	histone acetyltransferase complex subunit Epl1 (predicted)	1.51
SPCC553.01c		meiotic chromosome segregation protein		1.51
SPBC1734.07c		TRAPP complex subunit Trs85 (predicted)		1.51
SPCC1020.13c		phospholipase (predicted)	phospholipase (predicted)	1.51
SPBC56F2.11	*met6*	homoserine O-acetyltransferase		1.51
SPBC1711.12		serine protease (predicted)		1.51

SPBC409.03	*swi5*	Swi5 protein		1.51
SPBC16A3.01	*spn3*	septin Spn3		1.51
SPBC530.14c	*dsk1*	SR protein-specific kinase Dsk1		1.51
SPAC922.06		short chain dehydrogenase (predicted)	short chain dehydrogenase	1.51
SPAC3A11.09	*sod22*	plasma membrane alkali metal cation/H+ antiporter Sod22	plasma membrane alkali metal cation/H+ antiporter Sod22	1.51
SPAC1952.11c	*ure2*	urease	urease (PMID 9301025)	1.51
SPAC19B12.13	*cox1102*	fusion cytochrome c oxidase assembly protein Cox1102, mitochondrial ribosomal protein Rsm2202		1.51
SPCC663.01c	*ekc1*	protein phosphatase regulatory subunit Ekc1 (predicted)	protein phosphatase regulatory subunit Ekc1 (predicted)	1.51
SPBC11C11.11c		mitochondrial ATP-dependent DNA helicase Irc3 (predicted)		1.51
SPBC1778.10c	*ppk21*	serine/threonine protein kinase Ppk21		1.51
SPBP8B7.27	*mug30*	ubiquitin-protein ligase E3		1.51
SPBC3B9.15c	*scp1*	sterol regulatory element binding protein Scp1		1.51
SPAC1805.01c	*ppk6*	serine/threonine protein kinase Ppk6	serine/threonine protein kinase Ppk6	1.50
SPBC29A10.13	*atp7*	F0-ATPase subunit D		1.50
SPAC1399.04c		uracil phosphoribosyltransferase (predicted)	uracil phosphoribosyltransferase (predicted)	1.50
SPBC146.11c	*mug97*	meiotically upregulated gene Mug97		1.50
SPBP8B7.23		ubiquitin-protein ligase E3 (predicted)		1.50
SPAC16C9.07	*ppk5*	serine/threonine protein kinase Ppk5 (predicted)		1.50
SPBC577.06c		phosphatidylinositol kinase (predicted)		1.50
SPAC31A2.11c	*cuf1*	Cu metalloregulatory transcription factor Cuf1	Cu metalloregulatory transcription factor Cuf1 (PMID 10593913)	1.50
SPBP35G2.02		DUF1000 family protein		1.50
SPBC2D10.14c	*myo51*	myosin type V		1.50
