# Supplementary material for: The Fission Yeast GATA Factor, Gaf1, Modulates Sexual Development via Direct Down-Regulation of ste11+ Expression in Response to Nitrogen Starvation
Source: PLoS One. 2012 Aug 10;7(8):e42409. doi: 10.1371/journal.pone.0042409 (PMC3416868; doi:10.1371/journal.pone.0042409)
Supplement: Table S4 — List of the genes in Subgroup A. (PDF) [file pone.0042409.s004.pdf]

Table S4. List of the genes in Subgroup A

*** 61 genes**

Systematic	Gene name	Description (GeneDB)	Description (FunCat2)	Expression Ratio (gaf1Δ, +N) / (WT, +N)	(WT, -N) / (WT, +N)	(gaf1Δ, -N) / (gaf1Δ, +N)
SPBC947.05c		ferric-chelate reductase (predicted)		53.02	1.46	0.54
SPAC1F7.08	*fio1*	iron transport multicopper oxidase Fio1	iron transport multicopper oxidase Fio1	16.51	0.79	0.88
SPAC1F7.07c	*fip1*	iron permease Fip1	iron permease Fip1	15.96	0.99	0.95
SPBCPT2R1.08c	*tlh2*	RecQ type DNA helicase Tlh1		10.69	1.39	0.81
SPAC4G8.15c			dubious	8.70	0.60	0.59
SPAC8E11.06		sequence orphan	sequence orphan	4.46	1.41	0.59
SPAC212.02		sequence orphan	sequence orphan	4.41	1.43	0.03
SPAC8C9.12c		iron ion transporter (predicted)	iron ion transporter (predicted)	3.48	0.41	0.62
SPAC22F8.03c		sequence orphan	sequence orphan	3.12	1.00	0.36
SPCC330.03c		NADPH-hemoprotein reductase	NADPH-hemoprotein reductase	2.97	1.44	0.39
SPBC27B12.03c		lathosterol oxidase (predicted)		2.76	0.86	0.70
SPAC869.05c		sulfate transporter (predicted)	sulfate transporter (predicted)	2.72	1.19	0.88
SPBC1711.14	*rec15*	meiotic recombination protein Rec15		2.69	1.01	0.75
SPBC12C2.14c		dubious		2.45	0.82	0.89
SPBC16E9.01c	*php4*	CCAAT-binding factor complex subunit Php4		2.45	0.86	1.00
SPBC4F6.09	*str1*	siderophore-iron transporter Str1		2.42	1.42	1.09
SPAC343.07	*mug28*	RNA-binding protein Mug28	RNA-binding protein Mug28	2.41	0.19	0.83
SPAC212.09c		pseudogene	pseudogene	2.35	1.11	0.88
SPCC13B11.02c		sequence orphan	sequence orphan	2.30	0.97	0.26
SPBC428.07	*meu6*	meiotic chromosome segregation protein Meu6	meiotic chromosome segregation protein Meu6	2.18	0.67	0.61
SPAC5H10.07		sequence orphan	sequence orphan	2.16	0.89	0.24
SPBPJ4664.01	*dps1*	decaprenyl diphosphate synthase subunit Dps1		2.16	1.44	1.49
SPBC1271.06c	*mug96*	sequence orphan	sequence orphan	2.14	0.49	0.16
SPBC83.19c		sequence orphan		2.13	0.58	1.45
SPAC6B12.16	*meu26*	conserved fungal protein	conserved fungal protein	1.99	1.46	0.69
SPBC216.02	*mcp5*	cortical anchoring factor for dynein Mcp5/Num1		1.99	1.33	1.11

SPAC750.01		pseudogene	pseudogene	1.96	0.26	0.17
SPCC417.12		carboxylesterase-lipase family (predicted)	carboxylesterase-lipase family (predicted)	1.95	0.84	0.49
SPAC343.20		sequence orphan	sequence orphan	1.91	0.89	0.90
SPCC622.13c	*tti1*	Tel Two Interacting protein 1	conserved eukaryotic protein	1.91	1.21	0.76
SPAC227.13c	*isu1*	mitochondrial iron-sulfur cluster assembly scaffold protein Isu1	iron-sulfur cluster assembly scaffold protein Isu1	1.84	1.05	0.80
SPAC14C4.08	*mug5*	meiotically upregulated gene Mug5	meiotically upregulated gene Mug5	1.80	1.32	1.19
SPAC25G10.04c	*rec10*	meiotic recombination protein Rec10	meiotic recombination protein Rec10 (PMID 7586030)	1.79	0.65	1.45
SPCC61.01c	*str2*	siderophore-iron transporter Str2		1.78	1.43	1.17
SPAPB18E9.03c			dubious	1.78	0.77	0.35
SPCC622.12c		NADP-specific glutamate dehydrogenase (predicted)	NADP-specific glutamate dehydrogenase (predicted)	1.76	1.22	0.83
SPAPB8E5.08		sequence orphan	sequence orphan	1.76	0.35	0.46
SPAC23D3.12		inorganic phosphate transporter (predicted)	inorganic phosphate transporter (predicted)	1.73	1.00	1.34
SPBPB2B2.08		conserved fungal protein		1.71	0.37	0.35
SPBC29A10.12		HMG-box variant		1.69	0.51	0.48
SPAC15A10.10	*mde6*	Muskelin homolog	Muskelin homolog	1.68	0.98	0.90
SPBC12C2.03c		FAD binding protein (predicted)		1.66	0.61	1.27
SPBC36.02c		spermidine family transporter (predicted)		1.65	1.03	1.13
SPBC19C7.05		cell wall organization protein (predicted)		1.61	0.40	0.74
SPCC645.12c		sequence orphan	sequence orphan	1.58	1.03	0.59
SPAC869.02c		nitric oxide dioxygenase (predicted)	nitric oxide dioxygenase (predicted)	1.57	0.57	0.41
SPAC890.02c	*alp7*	TACC homolog (predicted)	TACC homolog (predicted) (PMID 14742702)	1.57	1.42	0.72
SPAC25B8.02	*sds3*	Clr6 histone deacetylase complex subunit Sds3 (predicted)	histone deacetylase complex subunit Sds3 (predicted)	1.57	0.96	1.04
SPCC13B11.04c		glutathione-dependent formaldehyde dehydrogenase (predicted)		1.57	1.36	1.49
SPAC688.06c	*slx4*	structure-specific endonuclease subunit	structure-specific endonuclease subunit (PMID 14528010)	1.56	1.38	1.27
SPBC115.03		gfo/idh/mocA family oxidoreductase (predicted)	gfo/idh/mocA family oxidoreductase (predicted)mito	1.56	0.51	0.69
SPAC5H10.04		NADPH dehydrogenase (predicted)	NADPH dehydrogenase (predicted)	1.56	0.65	0.80
SPBC530.02		membrane transporter		1.55	0.86	1.11
SPBC1198.07c		mannan endo-1,6-alpha-mannosidase (predicted)	mannan endo-1,6-alpha-mannosidase (predicted)	1.55	0.80	0.77

SPCC584.16c		sequence orphan	sequence orphan	1.54	1.20	1.02
SPCC417.04		dubious	dubious	1.54	1.41	1.41
SPAC3A12.18	*zwf1*	glucose-6-phosphate 1-dehydrogenase (predicted)		1.53	1.34	0.76
SPBC119.14	*rti1*	Rad22 homolog Rti1		1.53	0.90	1.24
SPCC126.14	*prp18*	U5 snRNP-associated protein Prp18	U5 snRNP-associated protein Prp18	1.52	1.37	1.16
SPBCPT2R1.02		sequence orphan		1.51	0.37	0.45
SPCC1322.03		TRP-like ion channel	TRP-like ion channel	1.50	1.06	1.25
