# Supplementary material for: The Fission Yeast GATA Factor, Gaf1, Modulates Sexual Development via Direct Down-Regulation of ste11+ Expression in Response to Nitrogen Starvation
Source: PLoS One. 2012 Aug 10;7(8):e42409. doi: 10.1371/journal.pone.0042409 (PMC3416868; doi:10.1371/journal.pone.0042409)
Supplement: Table S5 — List of the genes in Subgroup B. (PDF) [file pone.0042409.s005.pdf]

Table S5. List of the genes in Subgroup B

*** 56 genes**

Systematic	Gene name	Description (GeneDB)	Description (FunCat2)	Expression Ratio (gaf1Δ, +N) / (WT, +N)	Expression Ratio (WT, -N) / (WT, +N)	Expression Ratio (gaf1Δ, -N) / (gaf1Δ, +N)
SPAC1F8.02c		sequence orphan	sequence orphan	456.13	32.13	0.68
SPAC1F8.03c	*str3*	siderophore-iron transporter Str3	siderophore-iron transporter Str3 (PMID 12888492)	263.49	5.31	1.11
SPBC1683.09c	*frp1*	ferric-chelate reductase Frp1	ferric-chelate reductase Frp1	238.52	2.72	0.89
SPAC4H3.12c		dubious	sequence orphan	23.87	14.75	0.28
SPCC1620.04c	*mug55*	Cdc20/Fizzy family WD repeat protein	Cdc20/Fizzy family WD repeat protein	12.00	10.40	0.93
SPBPB21E7.04c		S-adenosylmethionine-dependent methyltransferase (predicted)	S-adenosylmethionine-dependent methyltransferase (predicted)	8.09	8.44	0.55
SPCC63.13		DNAJ domain protein	DNAJ domain protein	7.97	7.81	0.88
SPAC23G3.03	*sib2*	ornithine N5 monooxygenase (predicted)	ornithine N5 monooxygenase (predicted)	6.79	2.50	0.79
SPBPB21E7.05		sequence orphan	sequence orphan	4.94	3.29	0.43
SPAP27G11.08c	*meu32*	sequence orphan	sequence orphan	4.42	4.48	1.28
SPBC2F12.09c	*atf21*	transcription factor Atf21		4.39	3.03	0.17
SPAC13D6.01	*pof14*	F-box protein Pof14	F-box protein Pof14	4.28	2.52	1.34
SPBC106.02c	*srx1*	sulfiredoxin	sulphiredoxin (PMID 15824112)	4.27	1.57	1.24
SPCC757.02c		epimarase (predicted)	epimarase (predicted)	4.03	47.03	1.08
SPAC212.06c		pseudogene	pseudogene	3.91	1.68	0.49
SPAC1002.21			sequence orphan	3.67	6.58	0.89
SPBC359.05	*abc3*	ABC transporter Abc3	ABC transporter Abc3	3.60	1.55	0.86
SPAC1002.06c	*bqt2*	bouquet formation protein Bqt2	bouquet formation protein Bqt2	3.55	4.60	1.38
SPCC1393.10	*ctr4*	copper transporter complex subunit Ctr4	copper transporter complex subunit Ctr4 (PMID 11274192)	3.53	1.59	0.42
SPBP4G3.03		PI31 proteasome regulator related		3.32	40.76	1.35
SPAC1A6.11		dubious	dubious	3.29	2.30	0.66
SPCC737.04		S. pombe specific UPF0300 family protein 6	S. pombe specific UPF0300 family protein 6	3.14	2.75	1.23
SPCC1020.03		mitochondrial iron ion transporter	mitochondrial iron ion transporter	2.99	1.86	1.21
SPAC23G3.02c	*sib1*	ferrichrome synthetase Sib1	ferrichrome synthetase Sib1	2.79	2.49	1.28

SPAC15E1.07c	*moa1*	meiotic cohesin complex associated protein Moa1	meiotic cohesin complex associated protein Moa1	2.75	2.94	0.39
SPAC323.07c		MatE family transporter	MatE family transporter	2.43	2.47	1.32
SPAC186.07c		hydroxyacid dehydrogenase (predicted)	hydroxyacid dehydrogenase (predicted)	2.38	1.54	0.58
SPBC8D2.19	*mde3*	serine/threonine protein kinase Mde3		2.37	1.93	0.22
SPAC9G1.09	*sid1*	PAK-related kinase Sid1	PAK-related kinase Sid1	2.27	2.16	0.75
SPAC869.03c		urea transporter (predicted)	urea transporter (predicted)	2.26	1.65	0.47
SPCC1442.11c		sequence orphan	sequence orphan	2.20	2.30	1.11
SPBC887.17		uracil permease (predicted)		2.20	1.85	1.32
SPAC977.13c		hydrolase		2.18	6.80	1.37
SPBC215.04	*git11*	heterotrimeric G protein gamma subunit Git11		2.11	2.74	0.75
SPBC1683.12		nicotinic acid plasma membrane transporter (predicted)	nicotinic acid plasma membrane transporter (predicted)	2.05	2.05	1.39
SPCC338.02	*mug112*	sequence orphan	sequence orphan	1.97	3.48	0.51
SPBPB2B2.18		sequence orphan		1.97	3.29	1.22
SPAC11D3.17		zf-C2H2 type zinc finger protein	zinc finger protein	1.93	4.23	0.83
SPBC16G5.19		sequence orphan		1.92	2.00	0.43
SPBPB21E7.07	*aes1*	enhancer of RNA-mediated gene silencing	enhancer of RNA-mediated gene silencing (PMID 12034844)	1.92	7.14	1.10
SPAC24C9.15c	*spn5*	septin Spn5	septin Spn5	1.91	1.72	1.27
SPBC1271.07c		N-acetyltransferase (predicted)	N-acetyltransferase (predicted)	1.90	7.55	0.56
SPAC1751.04		sequence orphan	sequence orphan	1.86	2.59	0.62
SPACUNK4.15		2',3'-cyclic-nucleotide 3'-phosphodiesterase (predicted)	2',3'-cyclic-nucleotide 3'-phosphodiesterase (predicted)	1.81	1.70	1.35
SPAP27G11.16		sequence orphan	sequence orphan	1.80	2.14	0.98
SPBC23G7.13c		urea transporter (predicted)		1.80	184.12	0.51
SPCC63.03		DNAJ domain protein, DNAJC11 family	DNAJ domain protein, DNAJC11 family	1.79	1.73	0.85
SPBC1683.11c		isocitrate lyase	isocitrate lyase	1.73	1.84	1.24
SPAC1142.05	*ctr5*	copper transporter complex subunit Ctr5 (predicted)	copper transporter complex subunit Ctr5 (predicted)	1.67	1.60	0.98
SPCC965.12		dipeptidyl aminopeptidase (predicted)	dipeptidyl aminopeptidase (predicted)	1.63	2.25	0.70
SPAC6C3.03c		sequence orphan	sequence orphan	1.62	3.78	0.85
SPBC1921.04c		sequence orphan		1.59	3.10	1.28
SPCC70.08c		methyltransferase (predicted)	methyltransferase (predicted)	1.53	12.02	0.84
SPMIT.03		mitochondrial DNA binding endonuclease		1.52	1.76	1.03
SPAC1039.08		serine acetyltransferase (predicted)	serine acetyltransferase (predicted)	1.51	4.12	1.30
SPBC1711.01c	*matmi_1*	mating-type m-specific polypeptide mi		1.50	15.00	1.33
