# Supplementary material for: The Fission Yeast GATA Factor, Gaf1, Modulates Sexual Development via Direct Down-Regulation of ste11+ Expression in Response to Nitrogen Starvation
Source: PLoS One. 2012 Aug 10;7(8):e42409. doi: 10.1371/journal.pone.0042409 (PMC3416868; doi:10.1371/journal.pone.0042409)
Supplement: Table S6 — List of the genes in Subgroup C. (PDF) [file pone.0042409.s006.pdf]

Table S6. List of the genes in Subgroup C

*** 202 genes**

Systematic	Gene name	Description (GeneDB)	Description (FunCat2)	Expression Ratio (gaf1Δ, +N) / (WT, +N)	(WT, -N) / (WT, +N)	(gaf1Δ, -N) / (gaf1Δ, +N)
SPAC23C11.10		conserved eukaryotic protein	conserved eukaryotic protein	1.31	2.30	1.32
SPACUNK4.16c		alpha,alpha-trehalose-phosphate synthase (predicted)	alpha,alpha-trehalose-phosphate synthase (predicted)	1.01	1.74	1.41
SPAC12B10.11	*exg2*	glucan 1,3-beta-glucosidase Exg2	glucan 1,3-beta-glucosidase Exg2	1.03	1.60	1.15
SPBC8E4.03		agmatinase 2 (predicted)		1.06	5.96	0.90
SPBC3E7.02c	*hsp16*	heat shock protein Hsp16		1.08	1.67	1.35
SPCC965.07c	*gst2*	glutathione S-transferase Gst2	glutathione S-transferase Gst2	1.35	2.21	1.24
SPBC15D4.15	*pho2*	4-nitrophenylphosphatase		1.11	1.50	1.05
SPBC21C3.10c		5-amino-6-(5-phosphoribosylamino) uracil reductase		0.98	1.63	1.27
SPAC323.06c	*uba5*	NEDD8 activating enzyme (predicted)	NEDD8 activating enzyme (predicted)	1.06	1.60	1.44
SPAC24H6.01c		membrane bound O-acyltransferase, MBOAT (predicted)		0.91	1.57	1.41
SPBC19F8.01c	*spn7*	septin Spn7		1.09	1.75	1.08
SPBC1271.14		glutamate N-acetyltransferase (predicted)	glutamate N-acetyltransferase (predicted)	1.02	2.67	1.09
SPBC31F10.08	*mde2*	Mde2 protein		0.98	2.03	0.79
SPBC3E7.05c		conserved eukaryotic protein		0.87	1.53	1.46
SPBPB21E7.02c		pseudo SPAC5H10.03	pseudo SPAC5H10.03	1.13	31.49	0.98
SPBC24C6.07	*cdc14*	SIN component Cdc14		0.87	1.64	1.24
SPAC167.08	*Tf2-2*	retrotransposable element		0.84	3.23	1.23
SPBC354.15	*fap1*	L-pipecolate oxidase	L-pipecolate oxidase	0.94	2.24	1.27
SPCPB1C11.03		cysteine transporter (predicted)	cysteine transporter (predicted)	0.76	3.00	0.89
SPAC750.04c		dubious		0.95	1.64	0.10
SPAC3A12.02		inorganic pyrophosphatase	inorganic pyrophosphatase	1.31	1.86	1.15
SPBC725.06c	*ppk31*	serine/threonine protein kinase Ppk31 (predicted)		1.50	1.61	1.45
SPCC4B3.17	*cbp3*	ubiquinol cytochrome-c reductase assembly protein Cbp3	ubiquinol cytochrome-c reductase assembly protein Cbp3	0.95	1.99	1.37
SPBC4B4.11		conserved fungal protein		1.03	1.68	1.33

SPAC19D5.07	*uga1*	4-aminobutyrate aminotransferase (GABA transaminase)	4-aminobutyrate aminotransferase (GABA transaminase) (predicted)	0.92	3.89	1.11
SPCC1840.04		caspase	caspase	0.98	1.85	1.50
SPAC688.13	*scn1*	TatD DNase family Scn1	TatD DNase family Scn1	1.03	2.00	1.49
SPCC70.07c	*tmp1*	thymidylate kinase Tmp1	thymidylate kinase Tmp1	1.02	1.58	1.15
SPBC1683.06c		uridine ribohydrolase (predicted)	uridine ribohydrolase (predicted)	0.69	10.11	1.49
SPBC2D10.20	*ubc1*	ubiquitin conjugating enzyme Ubc1		1.01	1.77	1.40
SPAC31F12.01	*zds1*	zds family protein Zds1	zds family protein Zds1	0.97	1.64	1.46
SPAC664.13		sequence orphan	sequence orphan	0.92	1.74	1.22
SPBC14C8.11c		sequence orphan		1.43	1.89	1.16
SPBC8D2.12c		mitochondrial DNA binding protein (predicted)		0.98	1.53	1.43
SPAC6B12.13		protein phosphatase inhibitor (predicted)	protein phosphatase inhibitor (predicted)	1.02	1.91	1.24
SPAC24B11.11c	*sid2*	Sid2p-Mob1p kinase complex subunit Sid2	Sid2p-Mob1p kinase complex	0.84	1.60	1.49
SPBPB2B2.01		amino acid permease, unknown 12		1.43	7.57	0.03
SPAPB1A11.02		esterase/lipase (predicted)	esterase/lipase (predicted)	0.35	33.62	0.95
SPAC1039.07c		aminotransferase class-III	2,2-dialkylglycine decarboxylase (predicted)	1.12	2.96	1.36
SPCC794.08		HEAT repeat protein, unknown biological role	HEAT repeat protein, unknown biological role	0.85	1.63	1.49
SPAC11D3.11c		pseudogene		1.04	1.99	1.21
SPBC1683.01		inorganic phosphate transporter (predicted)	inorganic phosphate transporter (predicted)	0.72	1.78	1.32
SPAC11D3.07c		transcription factor (predicted)	transcription factor	1.04	2.17	1.46
SPAC4D7.01c	*sec71*	Sec7 domain	Sec7 domain	1.04	1.51	1.42
SPBC26H8.12		cytochrome c heme lyase		0.98	2.41	1.48
SPBC11B10.07c		CDC50 domain protein		0.96	1.51	1.40
SPAC1F12.09	*gpi17*	pig-S	pig-S	0.97	1.51	1.41
SPAC1093.04c		tRNA nucleotidyltransferase (predicted)	tRNA nucleotidyltransferase (predicted)	0.83	2.02	1.38
SPBC1706.01	*tea4*	tip elongation aberrant protein Tea4	tip elongation aberrant protein Tea4	0.91	1.65	1.43
SPAC977.10	*sod2*	CPA1 sodium ion/proton antiporter	CPA1 sodium ion/proton antiporter (PMID 8643524)	1.04	1.72	1.28
SPBC1773.17c		hydroxyacid dehydrogenase (predicted)		1.16	4.70	0.71
SPBC3E7.06c		membrane transporter		1.18	1.54	1.36
SPCC1840.06	*atp5*	F0-ATPase delta subunit	F0-ATPase delta subunit	0.91	1.52	1.36

SPBP8B7.08c		leucine carboxyl methyltransferase (predicted)		1.05	1.63	1.28
SPAC13G6.05c		TRAPP complex subunit Bet3 (predicted)	TRAPP complex subunit Bet3 (predicted)	0.93	1.64	1.49
SPCC1739.03	*hrr1*	Helicase Required for RNAi-mediated heterochromatin assembly Hrr1	Helicase Required for RNAi-mediated heterochromatin assembly Hrr1	1.17	1.52	1.33
SPAC25B8.10		trans-aconitate 3-methyltransferase (predicted)	trans-aconitate 3-methyltransferase (predicted)	1.14	2.22	1.44
SPAC3H1.06c		membrane transporter (predicted)	membrane transporter (predicted)	1.04	2.00	1.24
SPAPB24D3.08c		NADP-dependent oxidoreductase (predicted)	NADP-dependent oxidoreductase (predicted)	1.15	2.12	0.87
SPAC12B10.05		metallopeptidase	metallopeptidase	0.97	1.72	1.44
SPAC16A10.05c	*dad1*	DASH complex subunit Dad1	DASH complex subunit Dad1	0.97	1.57	1.05
SPBC1A4.01	*apc10*	anaphase-promoting complex subunit Apc10		1.17	1.79	1.02
SPBC36.12c	*git7*	SGT1-like protein Git7		1.02	1.60	1.14
SPAC630.13c	*tsc2*	tuberin	tuberin	0.93	1.55	1.26
SPBC19C7.09c	*uve1*	endonuclease Uve1		0.80	2.48	1.24
SPAC15A10.01	*atm1*	ABC family iron transporter Atm1	ABC family iron transporter Atm1	1.01	2.97	1.40
SPAC3G9.02	*oar2*	3-oxoacyl-[acyl-carrier-protein] reductase Oar2 (predicted)	3-oxoacyl-[acyl-carrier-protein] reductase Oar2 (predicted)	1.11	1.62	1.14
SPAC1805.16c		purine nucleoside phosphorylase (predicted)	purine nucleoside phosphorylase (predicted)	0.90	2.61	1.20
SPCC645.05c	*myo2*	myosin II heavy chain	myosin II heavy chain	1.06	1.67	1.35
SPAC1B3.11c	*ypt4*	GTPase Ypt4	GTPase Ypt4	1.00	1.95	1.34
SPBC20F10.07		GRAM domain protein		1.05	1.75	1.47
SPBC19F8.07	*crk1*	cyclin-dependent kinase activating kinase Crk1		1.33	3.23	1.48
SPBC1289.06c		sequence orphan		1.20	1.70	1.02
SPAC3H5.11		NAD/NADH kinase (predicted)	NAD/NADH kinase (predicted)	1.04	2.05	1.33
SPBC1703.09		sequence orphan		0.87	1.53	1.18
SPBC1773.16c		transcription factor (predicted)	transcription factor (predicted)	1.12	2.24	1.41
SPAC10F6.13c		aspartate aminotransferase (predicted)	aspartate aminotransferase (predicted)	1.07	1.50	0.73
SPCC1840.09		NAD dependent epimerase/dehydratase family protein	NAD dependent epimerase/dehydratase family protein	0.99	2.03	1.47
SPAC24C9.14	*otu1*	ubiquitin-specific protease (predicted)	ubiquitin-specific protease (predicted)	0.90	1.59	1.35
SPAC21E11.04	*ppr1*	L-azetidine-2-carboxylic acid acetyltransferase	L-azetidine-2-carboxylic acid acetyltransferase (PMID 12761200)	1.31	7.09	0.93

SPBC1604.03c		sequence orphan		0.92	1.51	1.33
SPBC1703.08c		5-formyltetrahydrofolate cyclo-ligase		1.35	3.11	0.84
SPAC25B8.09		trans-aconitate 3-methyltransferase (predicted)	trans-aconitate 3-methyltransferase (predicted)	1.11	2.41	1.23
SPAC824.02		GPI inositol deacylase	GPI inositol deacylase	1.05	2.61	1.01
SPAC227.17c		conserved protein (fungal and plant)	conserved protein (fungal and plant)	1.14	2.92	1.13
SPCC188.08c	*ubp22*	ubiquitin C-terminal hydrolase Ubp22	ubiquitin C-terminal hydrolase Ubp22	0.95	1.54	1.39
SPAC186.06		human MAWBP homolog	human MAWBP homolog	0.81	5.22	0.60
SPBC582.10c		ATP-dependent DNA helicase Rhp16b (predicted)	ATP-dependent DNA helicase Rhp16b (predicted)	1.06	2.32	1.30
SPBC27.03	*meu25*	sequence orphan		1.19	2.04	0.73
SPAC19E9.03	*pas1*	cyclin Pas1	cyclin Pas1	1.13	2.17	1.45
SPAC869.10c		proline specific permease (predicted)	proline specific permease (predicted)	0.48	3.42	0.74
SPAC922.03		1-aminocyclopropane-1-carboxylate deaminase (predicted)	1-aminocyclopropane-1-carboxylate deaminase (predicted)	1.04	2.36	1.42
SPCP20C8.01c		B13958 domain	B13958 domain	0.78	1.58	1.24
SPCPB16A4.05c		urease accessory protein UREG (predicted)	urease accessory protein UREG (predicted)	1.33	1.53	1.28
SPAC4G9.11c	*cmb1*	cytosine-mismatch binding protein 1	cytosine-mismatch binding protein 1	0.97	2.16	1.42
SPCC1672.03c		guanine deaminase (predicted)	guanine deaminase (predicted)	1.05	3.28	0.95
SPAC1399.02		membrane transporter	membrane transporter	1.03	1.89	1.14
SPCC320.09	*hem15*	ferrochelatase	ferrochelatase	0.96	1.98	1.25
SPAC4G8.11c	*atp10*	F1-F0 ATPase assembly protein (predicted)	F1-F0 ATPase assembly protein	1.04	1.67	1.02
SPAC22H12.03		mitochondrial hydrolase (predicted)	mitochondrial hydrolase	1.00	2.51	1.11
SPAC328.03	*tps1*	alpha,alpha-trehalose-phosphate synthase [UDP-forming]	alpha,alpha-trehalose-phosphate synthase [UDP-forming]	0.99	1.72	1.31
SPAC23H3.12c		conserved protein (fungal and plant)	conserved protein (fungal and plant)	0.87	2.12	1.23
SPAC1039.10	*mmf2*	homologous Pmf1p factor 1		1.42	9.58	1.10
SPBPB2B2.02	*mug180*	esterase/lipase (predicted)		1.39	3.44	0.70
SPBC4B4.10c	*mug77*	autophagy associated protein Atg5 (predicted)		1.05	1.54	1.26
SPBC12C2.04		NAD binding dehydrogenase family protein		0.70	1.51	1.48
SPBC30D10.14		dienelactone hydrolase family		0.91	1.76	1.36
SPCC1442.05c		conserved fungal protein	conserved fungal protein	0.86	1.73	1.44
SPAC521.03		short chain dehydrogenase (predicted)	short chain dehydrogenase (predicted)	0.88	2.33	1.07

SPAC25B8.04c		mitochondrial splicing suppressor (predicted)	mitochondrial splicing suppressor (predicted)	0.97	1.51	1.23
SPAC8F11.07c	*cdc24*	DNA replication protein Cdc24	DNA replication protein Cdc24	1.24	1.78	1.07
SPAC513.06c		dihydrodiol dehydrogenase (predicted)	dihydrodiol dehydrogenase (predicted)	0.83	3.19	1.16
SPAC1002.10c	*sgt1*	SGT1 family transcriptional regulator Sgt1	SGT1 family protein Sgt1	1.10	1.82	1.24
SPAC140.01	*sdh2*	succinate dehydrogenase (ubiquinone) iron-sulfur protein subunit	succinate dehydrogenase (ubiquinone) iron-sulfur protein subunit	1.05	1.57	1.39
SPBC646.12c	*gap1*	GTPase activating protein Gap1		1.02	1.66	1.40
SPAC2H10.02c		26S proteasome regulator (predicted)	26S proteasome regulator (predicted)	0.97	1.69	1.30
SPAC688.08	*srb8*	mediator complex subunit Srb8	mediator complex subunit Srb8 (PMID 12738880)	1.13	1.50	1.40
SPAC144.08		DNAJ domain protein Jac1 (predicted)	DNAJ domain protein Jac1 (predicted)	1.33	1.72	0.90
SPBC11B10.08		conserved fungal protein		1.06	1.69	1.25
SPCC74.04		amino acid permease, unknown 15	amino acid permease, unknown 15	1.02	1.96	1.04
SPAC589.04		metaxin 1	metaxin 1	0.87	1.51	1.39
SPBP35G2.10	*mit1*	SHREC complex subunit Mit1		0.89	1.63	1.39
SPAP27G11.05c	*vps41*	vacuolar protein sorting-associated protein Vps41	vacuolar protein sorting-associated protein Vps41	0.79	1.80	1.19
SPAC20G8.03	*itr2*	MFS myo-inositol transporter	MFS myo-inositol transporter	1.42	1.76	1.50
SPAC22H10.12c	*gdi1*	GDP dissociation inhibitor Gdi1 (predicted)	GDP dissociation inhibitor Gdi1 (predicted)	0.95	1.73	1.34
SPBC947.06c		spermidine family transporter (predicted)		1.37	1.59	1.12
SPAC13G6.15c		calcipressin		1.18	1.53	0.84
SPAC1296.06		NADPH cytochrome reductase (predicted)	NADPH cytochrome reductase	1.21	1.63	1.22
SPAC328.08c		tubulin specific chaperone cofactor C (predicted)	tubulin specific chaperone cofactor C (predicted)	0.90	1.86	1.42
SPCC191.06		sequence orphan	sequence orphan	0.77	2.85	1.12
SPBC106.08c	*mug2*	DUF1773 family protein 1	DUF1773 family protein 1	1.08	2.27	1.11
SPAC144.16		DUF59 family protein	DUF59 family protein	1.04	1.84	1.31
SPBC1348.01		S. pombe specific DUF999 protein family 5	S. pombe specific DUF999 protein family 5	1.14	1.50	1.20
SPBC4B4.02c	*nca2*	mitochondrial protein Nca2 (predicted)	mitochondrial protein Nca2 (predicted)	0.96	2.69	1.40
SPAC6G10.08	*idp1*	isocitrate dehydrogenase Idp1	isocitrate dehydrogenase Idp1	1.09	1.97	0.87
SPCC70.03c		proline dehydrogenase	proline dehydrogenase	0.98	1.86	1.27

SPCC1235.15	*dga1*	diacylglycerol O-acyltransferase		0.79	1.56	1.37
SPCC11E10.01		cystathionine beta-lyase (predicted)	cystathionine beta-lyase (predicted)	1.06	3.17	0.98
SPAC144.10c	*gwt1*	pig-W	pig-W	1.10	1.55	1.38
SPCC965.09		nitrilase (predicted)	nitrilase (predicted)	0.93	2.00	0.96
SPAPYUG7.06	*mug67*	PPPDE peptidase family (predicted)	PPPDE peptidase family (predicted)	1.04	1.95	1.31
SPAC4G9.10	*arg3*	ornithine carbamoyltransferase Arg3	ornithine carbamoyltransferase Arg3	0.97	1.59	0.61
SPCC63.05		TAP42 family protein (predicted)	TAP42 family protein (predicted)	1.05	1.71	1.17
SPBC409.17c		DUF1769 family protein		1.17	1.60	1.06
SPBC1778.03c		NADH pyrophosphatase (predicted)		0.83	1.83	1.18
SPCC16C4.03	*pin1*	peptidyl-prolyl cis-trans isomerase Pin1	peptidyl-prolyl cis-trans isomerase Pin1	0.86	1.66	1.31
SPBC1289.11	*spf38*	splicing factor Spf38	splicing factor Spf38	0.92	1.70	1.20
SPAC11D3.09		agmatinase (predicted)	agmatinase (predicted)	0.77	16.87	0.87
SPCC191.05c		nucleoside 2-deoxyribosyltransferase (predicted)	nucleoside 2-deoxyribosyltransferase (predicted)	1.02	2.13	0.70
SPAC26A3.11		amidohydrolase	amidohydrolase	0.93	1.66	1.25
SPAC12G12.16c		nuclease, XP-G family	nuclease, XP-G family	0.89	1.88	1.18
SPBC646.13	*sds23*	inducer of sexual development Sds23/Moc1		1.06	1.86	1.44
SPAC18B11.04	*ncs1*	related to neuronal calcium sensor Ncs1	related to neuronal calcium sensor Ncs1	1.35	1.69	1.42
SPAC11E3.09	*pyp3*	protein-tyrosine phosphatase Pyp3	protein-tyrosine phosphatase Pyp3	0.87	1.70	1.19
SPAC26F1.11		dubious	sequence orphan	0.95	1.63	1.17
SPCC338.04	*cid2*	caffeine induced death protein Cid2	caffeine induced death protein Cid2	1.17	2.11	1.36
SPAC824.07		hydroxyacylglutathione hydrolase (predicted)	hydroxyacylglutathione hydrolase (predicted)	0.96	2.47	1.20
SPAC2C4.15c	*ubx2*	UBX domain protein Ubx2	UBX domain protein Ubx2	1.12	1.78	1.04
SPAC10F6.14c		ABC1 kinase family protein	ABC1 kinase family protein	0.97	1.72	1.14
SPBC651.09c		RNA polymerase II associated Paf1 complex (predicted)		0.95	1.88	1.43
SPBC800.07c	*tsf1*	mitochondrial translation elongation factor EF-Ts Tsf1	mitochondrial translation elongation factor EF-Ts Tsf1 (PMID 15695360)	1.00	1.83	1.34
SPBC17G9.13c		sequence orphan		0.96	1.50	1.15
SPAC6F6.18c	*mug169*	sequence orphan	sequence orphan	1.37	2.03	1.22
SPBC27B12.14		mitochondrial membrane protein complex assembly protein		1.13	1.53	1.41
SPCC1795.07		mitochondrial ribosomal protein subunit S37 (predicted)	mitochondrial ribosomal protein subunit S37 (predicted)	1.13	1.56	1.06

SPBC13G1.05		DUF747 family protein		1.09	1.70	1.48
SPAC25H1.02	*jmj1*	Jmj1 protein	Jmj1 protein	1.02	1.59	0.91
SPBC17A3.02		conserved fungal protein		0.91	1.65	1.39
SPAC24C9.06c		aconitate hydratase	aconitate hydratase	1.12	1.53	1.24
SPBC2G2.07c	*mug178*	mitochondrial ribosomal protein subunit L51-b		1.00	1.72	1.45
SPCC1450.07c		D-amino acid oxidase (predicted)	D-amino acid oxidase (predicted)	0.90	2.43	0.95
SPAC3H8.04		chromosome segregation protein	chromosome segregation protein	0.84	1.52	1.49
SPAC22H12.01c	*mug35*	sequence orphan	sequence orphan	0.79	2.41	1.49
SPAC57A10.11c	*tim40*	TIM22 inner membrane protein import complex subunit Tim40	TIM22 inner membrane protein import complex subunit Tim40	1.10	1.85	1.31
SPBC1347.11	*sro1*	sequence orphan	sequence orphan	1.09	3.30	0.96
SPAC644.07		mitochondrial Rieske ISP assembly ATPase (predicted)	Rieske ISP assembly protein	1.00	1.91	1.45
SPBC119.09c		ORMDL family protein		1.10	1.51	1.37
SPAC14C4.03	*mek1*	Cds1/Rad53/Chk2 family protein kinase Mek1	Cds1/Rad53/Chk2 family protein kinase Mek1 (PMID 12482912)	1.39	1.58	1.38
SPCC1259.09c		pyruvate dehydrogenase protein x component	pyruvate dehydrogenase protein x component	1.14	2.51	1.11
SPAC11H11.02c	*mug162*	sequence orphan	sequence orphan	1.02	1.62	1.30
SPAC56F8.15		dubious	dubious	1.31	2.07	1.06
SPAC10F6.06	*vip1*	RNA-binding protein Vip1	RNA-binding protein Vip1	1.02	1.58	1.27
SPBC2G2.01c	*liz1*	pantothenate transporter		1.05	1.95	1.08
SPBC20F10.02c		DUF1741 family protein		0.82	1.54	1.19
SPBC3H7.02		sulfate transporter (predicted)		0.78	1.71	1.22
SPACUNK4.17		NAD binding dehydrogenase family protein	NAD binding dehydrogenase family protein	0.74	5.18	1.39
SPAC24C9.13c	*mrp10*	mitochondrial ribosomal protein subunit Mrp10	mitochondrial ribosomal protein subunit Mrp10	0.96	1.67	1.38
SPAC1B3.06c		UbiE family methyltransferase (predicted)	UbiE family methyltransferase (predicted)	1.14	3.00	1.21
SPBC649.03	*rhp14*	XP-A family homolog Rhp14	XP-A family homolog Rhp14	1.00	1.78	1.28
SPAC1486.01		manganese superoxide dismutase (AF069292)	manganese superoxide dismutase (AF069292)	0.93	2.15	1.49
SPAC22E12.15			dubious	1.33	1.66	1.08
SPAP27G11.12		human down-regulated in multiple cancers-1 homolog 1	human down-regulated in multiple cancers-1 homolog 1	0.87	1.69	1.19
SPBC83.12		sequence orphan		0.88	3.69	1.47

SPBC1709.09		mitochondrial translation termination factor		0.95	1.68	1.11
SPAPB17E12.04c	*csn2*	COP9/signalosome complex subunit Csn2	COP9/signalosome complex subunit Csn2 (PMID 11854407)	0.93	1.65	1.18
SPAC2E1P3.04		copper amine oxidase (predicted)	copper amine oxidase (predicted)	0.91	6.70	1.33
SPBC1289.02c	*uap2*	U2 snRNP-associated protein Uap2		1.18	1.54	1.23
SPAC1142.07c	*vps32*	vacuolar sorting protein Vps32	vacuolar sorting protein Vps32	0.94	1.55	1.50
SPBC215.07c		PWWP domain protein		0.82	1.52	1.46
SPAC22F3.13	*tsc1*	hamartin	hamartin	0.98	1.92	1.48
SPBC3B9.17	*isa2*	iron-sulfur protein Isa2		0.91	1.80	1.32
SPBC713.09		sequence orphan		0.90	1.76	1.37
