# Supplementary material for: The Fission Yeast GATA Factor, Gaf1, Modulates Sexual Development via Direct Down-Regulation of ste11+ Expression in Response to Nitrogen Starvation
Source: PLoS One. 2012 Aug 10;7(8):e42409. doi: 10.1371/journal.pone.0042409 (PMC3416868; doi:10.1371/journal.pone.0042409)
Supplement: Table S7 — List of the genes in Subgroup D. (PDF) [file pone.0042409.s007.pdf]

Table S7. List of the genes in Subgroup D

*** 983 genes**

Systematic	Gene name	Description (GeneDB)	Description (FunCat2)	Expression Ratio (gaf1Δ, +N) / (WT, +N)	(WT, -N) / (WT, +N)	(gaf1Δ, -N) / (gaf1Δ, +N)
SPCC417.06c	*ppk35*	serine/threonine protein kinase Ppk35	serine/threonine protein kinase Ppk35	1.19	3.93	4.00
SPAC589.07c		WD repeat protein Atg18	WD repeat protein Atg18	0.80	5.30	7.00
SPAC11H11.01	*sst6*	ESCRT I complex subunit Vps23	ESCRT I complex subunit Vps23	0.94	3.33	2.77
SPAC3C7.02c		protein kinase inhibitor (predicted)	protein kinase inhibitor (predicted)	1.02	12.84	2.24
SPCC126.02c	*pku70*	Ku domain protein Pku70	Ku domain protein Pku70	1.10	1.65	1.72
SPCC417.10		membrane transporter	membrane transporter	0.96	1.90	2.15
SPCC645.03c	*isa1*	iron-sulfur protein Isa1	iron-sulfur protein Isa1	1.09	3.16	1.92
SPCC1529.01		membrane transporter		0.70	2.27	4.25
SPCC622.06c		dubious	dubious	1.24	2.88	1.52
SPAC32A11.03c	*phx1*	homeobox transcription factor Phx1	homeobox transcription factor Phx1	0.84	2.89	2.57
SPBP8B7.23		ubiquitin-protein ligase E3 (predicted)		0.99	1.89	1.50
SPAC637.03		conserved fungal protein	conserved fungal protein	0.88	6.01	5.78
SPBC31E1.04	*pep12*	SNARE Pep12	SNARE Pep12	0.93	2.40	2.56
SPBC19C7.10		transcription factor (predicted)		0.90	2.57	3.06
SPAC26F1.04c	*etr1*	enoyl-[acyl-carrier protein] reductase	enoyl-[acyl-carrier protein] reductase	0.89	3.19	2.17
SPBC337.07c		carboxypeptidase (predicted)		0.89	1.62	1.56
SPBC20F10.03		conserved eukaryotic protein		0.79	3.02	2.97
SPBC146.02		sequence orphan		1.03	22.95	19.49
SPAC3C7.05c	*mug191*	alpha-1,6-mannanase (predicted)	alpha-1,6- mannanase (predicted)	0.91	10.52	4.67
SPBC17D11.01	*nep1*	NEDD8 protease Nep1		0.78	1.91	2.19
SPAC57A7.08	*pzh1*	serine/threonine protein phosphatase Pzh1	serine/threonine protein phosphatase Pzh1	0.86	2.02	2.13
SPBC16D10.06	*zrt1*	ZIP zinc transporter Zrt1		0.87	1.94	1.60
SPCC1739.08c		short chain dehydrogenase (predicted)	short chain dehydrogenase	1.22	56.61	38.29
SPBC14F5.10c		ubiquitin-protein ligase E3 (predicted)		1.13	3.18	2.36
SPAC11D3.10		nifs homolog	nifs homolog	1.41	1.82	1.64
SPBC17D1.07c		GTPase regulator (predicted)		0.86	1.64	2.23
SPBC1683.02		adenine deaminase (predicted)	adenine deaminase (predicted)	0.76	3.47	1.67
SPAC31G5.21		human family 32A homolog	human family 32A homolog	0.92	2.32	1.80

SPAC15A10.03c	*rhp54*	Rad54 homolog Rhp54	Rad54 homolog Rhp54	0.97	4.49	3.94
SPCC1393.05		sequence orphan	sequence orphan	0.83	1.86	2.40
SPAC1F7.10		hydantoin racemase family (predicted)	hydantoin racemase family (predicted)	1.29	4.53	2.37
SPAC9G1.06c	*cyk3*	cytokinesis protein Cyk3	cytokinesis protein Cyk3	0.92	1.56	1.87
SPBC19C2.11c		mitochondrial outer membrane protein (predicted)		0.99	3.92	2.67
SPBPB21E7.01c	*eno102*	enolase (predicted)	enolase (predicted)	0.43	26.65	2.07
SPCC285.06c	*wtf17*	wtf element Wtf17, pseudo	wtf element Wtf17, pseudo	1.34	5.60	4.12
SPBP4H10.10		rhomboid family protease		1.28	41.09	31.83
SPCC364.05	*vps3*	GTPase regulator Vps3 (predicted)	GTPase regulator Vps3 (predicted)	0.87	2.30	3.09
SPBP19A11.02c		sequence orphan		0.63	1.92	3.08
SPBC1347.01c	*rev1*	deoxycytidyl transferase Rev1 (predicted)	deoxycytidyl transferase Rev1 (predicted)	0.86	3.87	4.65
SPCC1281.08	*wtf11*	wtf element Wtf11	wtf element Wtf11	1.19	2.36	3.85
SPAC2G11.05c		BRO1 domain protein	BRO1 domain protein	1.06	2.50	3.47
SPAC6F12.08c		exocyst complex subunit Exo84	exocyst complex subunit Exo84	0.90	2.20	2.11
SPBC19G7.13	*tbf1*	telomeric repeat binding factor Trf1		0.94	1.91	2.16
SPBC365.08c		Der1-like (degradation in the ER) family		0.85	2.04	2.03
SPAC732.02c		fructose-2,6-bisphosphate 2-phosphatase activity (predicted)	6-phosphofructo-2-kinase (predicted)	0.98	2.37	2.30
SPAC9E9.15		CIA30 family protein	CIA30 family protein	0.84	3.37	2.03
SPAC24H6.11c		sulfate transporter (predicted)	sulfate transporter (predicted)	0.80	2.41	1.76
SPCC777.05	*gtr2*	Gtr1/RagA G protein Gtr2	Gtr1/RagA G protein Gtr2	0.88	1.58	1.96
SPBC337.12		human ZC3H3 homolog		0.89	1.67	2.16
SPAC12B10.16c	*mug157*	conserved protein (fungal and bacterial)	conserved protein (fungal and bacterial)	1.12	1.95	1.84
SPAC15A10.16	*bud6*	actin interacting protein 3 homolog Bud6		0.83	1.73	2.32
SPBC16E9.11c	*pub3*	ubiquitin-protein ligase E3		0.86	2.05	2.33
SPCC1259.12c		Ran GTPase binding protein (predicted)	Ran GTPase binding protein (predicted)	0.93	2.33	2.32
SPAC31A2.12		arrestin/PY protein 1	arrestin/PY protein 1	0.83	2.26	2.40
SPAC227.04		autophagy C terminal domain family protein	autophagy C terminal domain family protein	1.00	2.71	2.15
SPAC22H10.07	*scd2*	scaffold protein Scd2	scaffold protein Scd2	0.88	1.67	2.35
SPCC162.06c		vacuolar sorting protein Vps60	vacuolar sorting protein Vps60	0.99	3.58	3.84
SPCC330.11	*btb1*	BTB/POZ domain protein Btb1	BTB/POZ domain protein Btb1	0.90	1.90	2.04
SPCC1223.13	*cbf12*	CBF1/Su(H)/LAG-1 family transcription factor Cbf12	transcription factor (predicted)	0.65	2.39	1.75

SPAC513.05	*ams1*	alpha-mannosidase (predicted)	alpha-mannosidase (predicted)	0.93	4.40	3.21
SPCC622.05		dubious	dubious	0.93	2.72	1.95
SPAC11H11.04	*mam2*	pheromone p-factor receptor	pheromone p-factor receptor (PMID 1657593)	0.85	2.09	2.67
SPAC22H10.06c		dubious	dubious	0.43	1.54	2.06
SPBC21H7.06c		inositol metabolism protein Opi10 (predicted)		0.72	2.31	1.80
SPAC19B12.07c		human ZNF277P homolog	human ZNF277P homolog	0.98	1.75	1.60
SPAC57A7.09		human RNF family homolog	human RNF family homolog	0.88	4.07	4.10
SPBC32F12.09	*rum1*	CDK inhibitor Rum1		1.03	2.98	2.77
SPBC685.05	*gpi15*	pig-H (predicted)		0.73	2.03	1.71
SPAC17C9.12		MSP domain	MSP domain	0.94	2.04	1.78
SPAC3G6.05		Mvp17/PMP22 family protein 1	Mvp17/PMP22 family	0.79	1.88	1.55
SPBC36B7.09	*gcn2*	eIF2 alpha kinase Gcn2 (predicted)	eIF2 alpha kinase Gcn2 (predicted)	0.96	2.93	1.72
SPAC23H4.14	*vam6*	guanyl-nucleotide exchange factor Vam6	guanyl-nucleotide exchange factor Vma6	0.78	1.71	2.15
SPBC1683.13c		transcription factor (predicted)	transcription factor (predicted)	0.93	2.49	1.98
SPAC2G11.03c	*vps45*	vacuolar sorting protein Vps 45 (predicted)	vacuolar sorting protein Vps 45	0.83	1.74	1.99
SPBC15C4.06c		ubiquitin-protein ligase E3 (predicted)		1.11	4.64	4.12
SPAC1F5.04c	*cdc12*	formin Cdc12	formin Cdc12	0.89	1.63	1.75
SPAC630.04c		sequence orphan	sequence orphan	1.06	2.19	1.85
SPAC1834.09	*mug51*	conserved fungal protein	conserved fungal protein	0.73	2.24	2.19
SPAPB24D3.10c	*agl1*	alpha-glucosidase Agl1	alpha-glucosidase Agl1	0.72	31.15	43.09
SPBP35G2.11c		transcription related zf-ZZ type zinc finger protein		1.00	2.34	2.13
SPAC13G7.07	*arb2*	argonaute binding protein 2	argonaute binding protein 2	1.12	2.20	2.13
SPBC3H7.08c		conserved fungal protein		0.51	12.30	6.49
SPAC4G9.04c		cleavage and polyadenylation specificity factor (predicted)	cleavage and polyadenylation specificity factor (predicted)	0.87	1.55	1.76
SPBC4C3.12	*41153*	fork head transcription factor Sep1		0.86	2.72	3.04
SPAC11D3.16c		sequence orphan	sequence orphan	0.95	5.00	2.07
SPBC21.06c	*cdc7*	serine/threonine protein kinase Cdc7		0.73	2.18	2.62
SPCC594.06c		SNARE Vam7 (predicted)	SNARE Vam7 (predicted)	1.00	4.11	3.18
SPAC1610.04	*mug99*	meiotically upregulated gene Mug99	meiotically upregulated gene Mug99	1.03	3.44	1.94
SPCPJ732.02c		xylulose kinase (predicted)	xylulose kinase (predicted)	0.95	4.20	3.06
SPBC16C6.06	*pep1*	sorting receptor for CPY		1.09	2.55	2.42
SPAPB1A10.10c	*ypt71*	GTPase Ypt71	GTPase Ypt71	0.93	2.23	2.08

SPAPB2B4.01c	*gpi12*	pig-L (predicted)	pig-L	1.07	4.01	2.60
SPBC36.11		sequence orphan		0.88	3.34	3.82
SPAC27F1.08	*pdt1*	Nramp family manganese ion transporter	Nramp family manganese ion transporter	0.85	1.65	1.91
SPBC1A4.04		sequence orphan		0.91	1.88	1.55
SPAC1952.17c		GTPase activating protein		0.96	2.74	1.92
SPAC26A3.14c		DUF1748 family protein	DUF1748 family protein	0.98	1.78	1.52
SPBC56F2.06	*mug147*	sequence orphan		1.37	43.26	19.90
SPCC1020.05		phosphoprotein phosphatase (predicted)	phosphoprotein phosphatase (predicted)	0.99	5.12	6.74
SPBC21.07c	*ppk24*	serine/threonine protein kinase Ppk24		0.99	2.25	3.23
SPCC622.02		dubious	dubious	0.84	2.50	2.69
SPAC2F7.08c	*snf5*	chromatin remodeling complex subunit Snf5 (predicted)	chromatin remodeling complex subunit Snf5 (predicted)	1.11	1.79	1.63
SPBC1718.07c	*zfs1*	CCCH tandem zinc finger protein, human Tristetraprolin homolog Zfs1		0.96	1.74	2.06
SPAC15A10.05c	*mug182*	YjeF family protein	YjeF family protein	0.89	19.31	10.16
SPCC777.13	*vps35*	retromer complex subunit Vps35	retromer complex subunit Vps35	0.93	2.41	2.08
SPAC17A2.01	*bsu1*	high-affinity import carrier for pyridoxine, pyridoxal, and pyridoxamine Bsu1	high-affinity import carrier for pyridoxine,pyridoxal, and pyridoxamine Bsu1	0.98	8.40	12.05
SPAC1687.12c	*coq4*	ubiquinone biosynthesis protein Coq4 (predicted)	ubiquinone biosynthesis protein Coq4	1.06	2.04	1.71
SPAC1635.01		voltage-dependent anion-selective channel	voltage-dependent anion-selective channel	1.03	2.01	1.82
SPCPB16A4.06c		sequence orphan	sequence orphan	1.33	74.11	13.20
SPCC757.13		membrane transporter (predicted)	membrane transporter (predicted)	0.84	7.57	1.54
SPAC2E1P3.01		zinc binding dehydrogenase	zinc binding dehydrogenase	1.13	3.38	2.30
SPBC14C8.08c		dubious		0.86	1.77	2.12
SPAC3A11.10c		dipeptidyl aminopeptidase (predicted)	dipeptidyl aminopeptidase (predicted)	1.26	6.91	3.17
SPAC144.17c		6-phosphofructo-2-kinase	6-phosphofructo-2-kinase	0.90	1.72	1.65
SPAC13F5.03c		mitochondrial iron-containing alcohol dehydrogenase family protein	glycerol dehydrogenase (Phlippen, Stevens, Wolf,Zimmermann manuscript in preparation)	0.81	21.62	22.05
SPAC1A6.06c	*meu31*	sequence orphan	sequence orphan	0.95	15.76	14.15
SPAC823.03	*ppk15*	serine/threonine protein kinase Ppk15 (predicted)	serine/threonine protein kinase Ppk15 (predicted)	0.95	2.68	2.23
SPBC29A3.03c		ubiquitin-protein ligase E3 (predicted)		0.99	3.49	2.65

SPBC21C3.11	*ubx4*	UBX domain protein Ubx4 (predicted)		0.87	2.48	1.96
SPBC776.15c		dihydrolipoamide S-succinyltransferase, e2 component of oxoglutarate dehydrogenase complex (predicted)		0.90	2.15	1.72
SPBC365.15	*alp4*	gamma tubulin complex Spc97/GCP2 subunit Alp4		1.02	1.54	1.58
SPAC16E8.03	*gna1*	glucosamine-phosphate N-acetyltransferase	glucosamine-phosphate N-acetyltransferase	0.90	7.34	4.59
SPBC23E6.03c	*nta1*	protein N-terminal amidase Nta1 (predicted)		0.87	2.81	2.06
SPAC57A10.08c		esterase/lipase (predicted)	esterase/lipase (predicted)	0.90	2.18	2.68
SPAC1D4.11c	*lkh1*	dual specificity protein kinase Lkh1	dual specificity protein kinase Lkh1	0.76	1.56	3.09
SPBC691.05c		membrane transporter (predicted)		0.88	2.25	2.46
SPAC23H3.15c		sequence orphan		0.55	27.92	21.53
SPBC14F5.11c	*mug186*	sorting nexin Snx41		0.97	2.65	2.55
SPAC6F6.08c	*cdc16*	two-component GAP Cdc16	two-component GAP Cdc16	0.81	1.77	2.26
SPAC1399.04c		uracil phosphoribosyltransferase (predicted)	uracil phosphoribosyltransferase (predicted)	0.75	1.56	1.50
SPBC16G5.16		transcription factor, zf-fungal binuclear cluster type (predicted)		0.84	1.77	1.69
SPAC22H10.13	*zym1*	metallothionein	metallothionein (PMID 12050156)	0.82	12.32	6.96
SPBC1652.01		conserved fungal protein		1.10	2.71	2.67
SPBC1709.16c		aromatic ring-opening dioxygenase (predicted)		0.62	2.56	2.61
SPBC17F3.02	*nak1*	PAK-related kinase Nak1		0.90	1.57	2.07
SPAC25H1.09	*mde5*	alpha-amylase homolog Mde5	alpha-amylase homolog Mde5	0.53	1.56	2.66
SPAC2E12.02	*hsf1*	transcription factor Hsf1	transcription factor Hsf1	0.82	1.94	1.90
SPCC550.09		peroxin Pex32 (predicted)	peroxin Pex32 (predicted)	0.93	2.25	2.06
SPBC215.11c		aldo/keto reductase, unknown biological role		0.74	9.24	5.99
SPBC15D4.07c	*atg9*	autophagy associated protein Atg9 (predicted)		0.91	2.86	2.65
SPAC607.07c		sequence orphan	sequence orphan	0.81	1.74	1.78
SPCC1393.02c		non-specific DNA binding protein Spt2 (predicted)	non-specific DNA binding protein Spt2 (predicted)	0.87	1.70	1.78
SPCC965.05c	*thp1*	uracil DNA N-glycosylase Thp1	uracil DNA N-glycosylase Thp1 (PMID 12711670)	0.98	1.97	2.27
SPAC140.04		conserved fungal protein	conserved fungal protein	0.90	2.48	2.31
SPAC27E2.06c		methionine-tRNA ligase, mitochondrial	methionine-tRNA ligase	1.12	2.55	1.65

SPAC26F1.14c	*aif1*	apoptosis-inducing factor homolog Aif1	apoptosis-inducing factor homolog Aif1	1.05	10.29	3.26
SPBC20F10.10		cyclin pho85 family		0.75	1.64	1.76
SPAC1006.09	*win1*	MAP kinase kinase kinase Win1		0.80	2.00	2.92
SPCC126.04c		SAGA complex subunit Sgf73 (predicted)	SAGA complex subunit Sgf73 (predicted)	0.92	2.59	2.22
SPAC29B12.11c		human WW domain binding protein-2 ortholog	human WW domain binding protein-2 ortholog	1.14	3.79	2.86
SPBC800.14c		DUF1772 family protein	DUF1772 family protein	0.93	3.70	3.82
SPAC16E8.02		DUF962 family protein	conserved protein (broad species distribution)	0.98	6.27	3.92
SPCC622.11		LMBR1-like membrane protein	LMBR1-like membrane protein	0.83	4.06	3.93
SPAC29B12.14c		purine transporter (predicted)	purine transporter (predicted)	1.10	4.26	1.94
SPAP7G5.03		conjugation protein (predicted)	conjugation protein (predicted)	0.95	4.00	6.03
SPBC1198.06c		mannan endo-1,6-alpha-mannosidase (predicted)	mannan endo-1,6-alpha-mannosidase (predicted)	0.89	2.68	2.56
SPAC8F11.05c	*mug130*	sequence orphan	sequence orphan	1.26	2.31	2.08
SPBC1773.08c		mannosyltransferase complex subunit (predicted)	mannosyltransferase complex subunit (predicted)	0.93	1.69	2.21
SPAC25G10.07c	*cut7*	kinesin-like protein Cut7	kinesin-like protein Cut7	1.01	1.74	1.69
SPBC32F12.01c		inositol phosphosphingolipid phospholipase C (predicted)		1.01	1.82	2.61
SPAC1805.15c	*pub2*	ubiquitin-protein ligase Pub2	ubiquitin-protein ligase Pub2	0.64	2.94	4.73
SPAC3H1.14		cytoplasmic vesicle protein, Vid24 family	cytoplasmic vesicle protein, Vid24 family	0.89	2.16	1.99
SPBP4H10.09	*rsv1*	transcription factor Rsv1 (predicted)		0.63	15.71	33.11
SPAC1B1.02c		NAD/NADH kinase (predicted)	NAD/NADH kinase (predicted)	1.11	2.21	2.01
SPBC3E7.01	*fab1*	1-phosphatidylinositol-3-phosphate 5-kinase Fab1	1-phosphatidylinositol-3-phosphate 5-kinase Fab1 (PMID 10567352)	1.00	1.72	1.92
SPCC737.05		peroxin Pex28/29	peroxin Pex28/29	0.71	1.90	2.06
SPAC31G5.12c	*maf1*	repressor of RNA polymerase III Maf1 (predicted)	repressor of RNA polymerase III Maf1 (predicted)	0.85	1.73	2.57
SPAC23C11.08	*php3*	CCAAT-binding factor complex subunit Php3	CCAAT-binding factor complex subunit Php3 (PMID 8223474)	0.92	1.50	1.82
SPAPB1A10.14		F-box protein	F-box protein, unnamed	1.29	3.82	4.17
SPCC1393.12		sequence orphan	sequence orphan	0.79	8.92	8.24
SPCC4G3.12c		ubiquitin-protein ligase E3 (predicted)	ubiquitin-protein ligase E3 (predicted)	0.94	3.21	2.93
SPCC622.04		dubious	dubious	0.71	1.79	3.23
SPAC4G9.19		DNAJ domain protein DNAJB family	DNAJ domain protein DNAJB family	0.88	3.23	2.88

SPCC1620.09c	*tfg1*	transcription factor TFIIF complex alpha subunit Tfg1 (predicted)	transcription factor TFIIF complex alpha subunit Tfg1	0.97	1.90	1.73
SPBC24C6.09c		phosphoketolase (predicted)		0.87	46.46	5.69
SPBP8B7.13		conserved fungal protein		0.98	2.92	2.06
SPBC1703.12	*ubp9*	ubiquitin C-terminal hydrolase Ubp9	ubiquitin C-terminal hydrolase Ubp9	0.88	2.38	3.26
SPBC660.09	*mug168*	sequence orphan	sequence orphan	0.95	5.42	2.57
SPCC736.13		short chain dehydrogenase (predicted)	short chain dehydrogenase	1.24	4.36	2.86
SPAC13G6.03	*gpi7*	GPI anchor biosynthesis protein Gpi7 (predicted)	GPI anchor biosynthesis protein Gpi7 (predicted)	0.77	1.97	1.67
SPAC3H1.08c		DUF1640 family protein	DUF1640 family protein	0.95	1.73	1.52
SPCC1906.04	*wtf20*	wtf element Wtf20	wtf element Wtf20	0.92	3.28	3.51
SPBC17D11.08		WD repeat protein, human WDR68 family		0.99	3.27	2.87
SPCC74.03c	*ssp2*	serine/threonine protein kinase Ssp2	serine/threonine protein kinase Ssp2	0.98	1.54	1.64
SPCC1183.11		MS ion channel protein 1		0.74	1.91	2.38
SPAC26F1.07		2-methylbutyraldehyde reductase (predicted)	2-methylbutyraldehyde reductase (predicted)	0.83	1.53	1.58
SPBC428.12c		RNA-binding protein	RNA-binding protein	0.98	1.81	1.61
SPBC3E7.04c		pseudo		0.99	2.72	2.65
SPAC17C9.05c	*pmc3*	mediator complex subunit Pmc3 (pers. comm. Tomas Linder, from PMID 10625684)	mediator complex subunit Pmc3 (pers. comm. Tomas Linder, from PMID 10625684)	0.86	2.27	2.46
SPBC21C3.01c	*vps13a*	chorein homolog	chorein homolog	1.08	1.70	1.72
SPBC23E6.01c		RNA-binding protein, rrm type		0.94	2.51	2.54
SPAC18G6.12c		hypothetical protein	hypothetical protein	0.75	2.94	2.90
SPAC9.10	*thi9*	thiamine transporter Thi9	amino acid permease, unknown 2	0.98	1.96	1.99
SPAC6F12.15c	*cut9*	anaphase-promoting complex subunit Cut9	anaphase-promoting complex subunit Cut9	0.93	2.28	1.87
SPAC521.04c		calcium permease (predicted)	calcium permease (predicted)	0.86	2.63	2.57
SPCC5E4.10c		sequence orphan	sequence orphan	1.25	1.99	1.63
SPAC5D6.07c	*pxa1*	PXA domain protein Pxa1	PXA domain protein	0.86	3.11	2.89
SPBC18H10.10c	*cwc16*	complexed with Cdc5 protein Cwf16		0.97	1.96	1.83
SPAC22G7.11c		conserved fungal protein	conserved fungal protein	0.60	52.42	10.52
SPAC23A1.16c		DUF408 family protein	DUF408 family protein	1.16	1.70	1.89
SPBC11B10.05c	*rsp1*	random septum position protein Rsp1		0.92	1.71	2.05
SPAC3A11.06	*mvp1*	sorting nexin Mvp1	sorting nexin Mvp1	0.91	3.79	4.05
SPCC1450.09c		phospholipase (predicted)	phospholipase (predicted)	0.96	2.52	1.59
SPAC2C4.09		DUF1640 family protein	DUF1640 family protein	0.92	1.88	1.95

SPAC3G6.07		dubious	sequence orphan	0.61	21.84	5.31
SPAC22E12.03c		THIJ/PFPI family peptidase (predicted)	THIJ/PFPI family peptidase (predicted)	0.87	1.86	1.57
SPAC5D6.08c	*mes1*	meiosis II protein Mes1	meiosis II protein Mes1	1.00	1.51	2.11
SPAC186.03		L-asparaginase (predicted)	L-asparaginase	1.10	2.62	2.71
SPAC23D3.14c	*aah2*	alpha-amylase homolog Aah2	alpha-amylase homolog Aah2	1.24	1.85	1.76
SPBC8E4.04		aldo/keto reductase involved in pentose catabolism (predicted)		0.83	3.39	2.75
SPAC1556.01c	*rad50*	DNA repair protein Rad50	DNA repair protein Rad50	1.08	4.50	3.04
SPBC14C8.09c		conserved fungal protein		0.79	1.94	2.00
SPAC2E12.03c		G-protein coupled receptor (predicted)	G-protein coupled receptor (predicted)	0.81	10.00	3.79
SPAC29E6.01	*pof11*	F-box protein Pof11	F-box protein Pof11	1.04	2.22	2.26
SPAC29B12.03	*spd1*	ribonucleotide reductase (RNR) inhibitor	ribonucleotide reductase (RNR) inhibitor	0.85	2.78	2.97
SPAC13C5.04		amidotransferase (predicted)	glutamine amidotransferase (predicted)	0.91	6.78	3.07
SPAC5H10.11	*gmh1*	alpha-1,2-galactosyltransferase Gmh1 (predicted)	alpha-1,2-galactosyltransferase Gmh1	1.02	15.73	5.64
SPCC622.01c		sequence orphan	sequence orphan	0.91	1.59	2.15
SPAC22H10.10	*alp21*	tubulin specific chaperone cofactor E	tubulin specific chaperone cofactor E	1.05	2.38	2.38
SPBC776.05		membrane transporter (predicted)		0.95	6.01	3.72
SPCC1739.01		zf-CCCH type zinc finger protein	zf-CCCH type zinc finger protein	1.10	2.82	2.49
SPAC821.04c	*cid13*	poly(A) polymerase Cid13	poly(A) polymerase Cid13 (PMID 12062100)	1.22	3.45	3.25
SPCC24B10.07	*gad8*	serine/threonine protein kinase Gad8	serine/threonine protein kinase Gad8 (PMID 12805221)	0.89	2.00	2.44
SPCC777.17c		mitochondrial ribosomal protein subunit L9 (predicted)	mitochondrial ribosomal protein subunit L9 (predicted)	1.05	1.82	2.03
SPAC16.05c	*sfp1*	transcription factor Sfp1 (predicted)	transcription factor Sfp1 (predicted)	0.89	2.51	2.31
SPCC191.01		sequence orphan	sequence orphan	0.90	6.17	7.39
SPAC4G9.13c	*vps26*	retromer complex subunit Vps26	retromer complex subunit Vps26	1.07	2.41	2.17
SPCC18B5.03	*wee1*	dual specificity protein kinase Wee1	dual specificity protein kinase Wee1	0.94	2.63	3.24
SPAC23C4.16c	*atg15*	triacylglycerol lipase Atg15 (predicted)	triacylglycerol lipase Atg15 (predicted)	0.93	1.81	2.15
SPAC15E1.02c		DUF1761 family protein	DUF1761 family protein	0.61	19.11	8.63
SPCC70.05c		serine/threonine protein kinase (predicted)	serine/threonine protein kinase (predicted)	0.96	1.81	1.84
SPAC3G9.11c		pyruvate decarboxylase (predicted)	pyruvate decarboxylase (predicted)	0.87	56.51	51.07
SPAP32A8.02		xylose and arabinose reductase (predicted)	xylose and arabinose reductase (predicted)	0.99	2.31	2.30

SPBC18E5.10		iron sulfur cluster assembly protein (predicted)		0.95	1.87	1.61
SPAC644.06c	*cdr1*	GIN4 family protein kinase Cdr1	GIN4 family protein kinase Cdr1	0.83	2.09	2.12
SPCC18.17c		sequence orphan	sequence orphan	0.82	1.63	1.61
SPAC343.04c	*gnr1*	heterotrimeric G protein beta subunit Gnr1	heterotrimeric G protein beta subunit Gnr1	0.99	1.91	1.76
SPAC13G7.05		acyl-coA-sterol acyltransferase (predicted)	acyl-coA-sterol acyltransferase (predicted)	0.88	2.67	2.02
SPAC30D11.01c		alpha-glucosidase		1.07	5.00	5.48
SPAP8A3.12c		tripeptidylpeptidase (predicted)	tripeptidylpeptidase (predicted)	1.30	2.45	1.78
SPBC15D4.13c		sequence orphan		0.95	2.97	1.87
SPCC1450.06c	*grx3*	monothiol glutaredoxin Grx3	monothiol glutaredoxin Grx3	0.90	1.94	2.17
SPAPB2B4.06		conserved fungal protein	conserved fungal protein	1.02	4.56	3.58
SPAC11D3.08c		amino acid permease, unknown 1	amino acid permease, unknown 1	1.21	2.77	2.42
SPCC1494.09c		sequence orphan	sequence orphan	0.99	2.00	1.73
SPBC9B6.03		zf-FYVE type zinc finger protein		1.11	1.60	3.05
SPCC1494.01		iron/ascorbate oxidoreductase family		0.82	6.07	2.21
SPCC970.01	*rad16*	DNA repair endonuclease XPF	DNA repair endonuclease XPF	0.88	1.90	1.90
SPCC794.02	*wtf5*	wtf element Wtf5	wtf element Wtf5	1.06	2.87	4.42
SPCC417.05c	*chr2*	chitin synthase regulatory factor (putative) Chr2	chitin synthase regulatory factor (putative) Chr2 (PMID 15449309)	0.89	4.10	2.73
SPCC663.02	*wtf14*	wtf element Wtf14	wtf element Wtf14	1.03	3.38	2.34
SPAC57A7.05		conserved protein (fungal and plant)	conserved protein (fungal and plant)	0.70	7.31	4.42
SPBC1A4.06c		mitochondrial matrix protein import protein		1.06	2.40	1.85
SPBC12C2.08	*dnm1*	dynamin Dnm1	dynamin Dnm1	0.94	2.18	1.64
SPCC1450.08c	*wtf16*	wtf element Wtf16	wtf element Wtf16	0.92	2.84	4.43
SPAC23A1.17		WIP homolog	WIP homolog	0.98	1.55	1.87
SPBP23A10.09		GINS complex subunit Psf1 (predicted)		0.84	2.24	2.24
SPBC25B2.08		sequence orphan		0.69	4.08	3.36
SPAC1F5.08c	*yam8*	calcium transport protein	calcium transport protein	1.07	1.55	2.65
SPCC1919.06c	*wtf25*	wtf element	wtf element	1.12	3.26	2.18
SPAC17C9.16c		MFS family transmembrane transporter Mfs1	MFS family transmembrane transporter Mfs1	1.38	2.94	1.70
SPAC1D4.13	*byr1*	MAP kinase kinase Byr1	MAP kinase kinase Byr1	1.22	2.30	1.79
SPBC725.05c		nucleotide pyrophosphatase (predicted)		0.92	2.16	2.36
SPAC1952.11c	*ure2*	urease	urease (PMID 9301025)	0.88	1.66	1.51
SPAC4G8.10	*gos1*	SNARE Gos1 (predicted)	SNARE Gos1	1.21	3.66	1.97

SPBC1271.05c		zinc finger protein zf-AN1 type	zinc finger protein zf-AN1 type	1.49	11.92	6.24
SPBC1706.03	*fzo1*	mitochondrial fusion GTPase protein		0.97	2.13	1.67
SPAC5D6.04		auxin family transmembrane transporter (predicted)	auxin family	1.13	2.66	1.87
SPAC19A8.12	*dcp2*	mRNA decapping complex subunit Dcp2	mRNA decapping complex subunit Dcp2	0.92	1.55	1.59
SPBC29A10.09c		CAF1 family ribonuclease		1.02	3.33	2.93
SPAPB1A10.12c	*alo1*	D-arabinono-1,4-lactone oxidase	D-arabinono-1,4-lactone oxidase	1.07	1.65	1.54
SPBC365.12c	*ish1*	LEA domain protein		0.87	6.93	5.30
SPAC2F3.05c		xylose and arabinose reductase (predicted)	xylose and arabinose reductase (predicted)	0.62	5.39	3.13
SPAC1B9.02c	*sck1*	serine/threonine protein kinase Sck1	serine/threonine protein kinase Sck1	0.99	1.69	1.67
SPAC513.03	*mfm2*	M-factor precursor Mfm2	M-factor precursor Mfm2	0.82	3.72	16.93
SPBC1921.07c		SAGA complex subunit Sgf29 (predicted)		0.97	1.67	1.53
SPBC337.15c	*coq7*	ubiquinone biosynthesis protein Coq7		1.01	2.72	2.27
SPBC13E7.11		mitochondrial rhomboid protease		0.79	2.33	2.22
SPAC3C7.03c	*rhp55*	RecA family ATPase Rhp55	RecA family ATPase Rhp55	0.90	2.59	2.26
SPBC30B4.02c		R3H and G-patch domain, implicated in splicing		1.10	2.75	2.75
SPAC6G9.16c		sequence orphan	sequence orphan	0.86	5.30	3.34
SPBC16A3.02c		mitochondrial peptidase (predicted)		0.86	3.83	3.66
SPAC1F3.03		Lgl family protein	Lgl family protein	1.06	2.83	2.33
SPBC29B5.02c	*isp4*	OPT oligopeptide transporter family		0.65	9.49	2.05
SPBC887.19	*rft1*	Man5GlcNac2-PP-Dol translocation protein Rft1		1.00	2.46	2.77
SPCC736.11	*ago1*	argonaute	argonaute	0.94	1.75	1.78
SPAC9.08c		steroid reductase (predicted)	steroid reductase (predicted)	0.99	1.69	1.70
SPCC16C4.02c		DUF1941 family protein	DUF1941 family protein	1.14	1.79	1.57
SPAC29A4.17c		FUN14 family protein	FUN14 family protein	1.04	6.70	3.68
SPBP8B7.28c		sequence orphan		0.76	1.84	1.75
SPAC16E8.16		transcription factor TFIIB	transcription factor TFIIB (PMID 12359329)	0.99	2.28	1.67
SPAPJ696.01c	*vps17*	retromer complex subunit Vps17	retromer complex subunit Vps17	1.01	1.94	1.89
SPAC6G10.03c		abhydrolase family protein, unknown biological role	abhydrolase family protein, unknown biological role	0.91	2.48	3.11
SPBC17D11.02c		synoviolin homolog		0.83	1.74	1.89
SPAC6G9.06c	*pcp1*	pericentrin Pcp1	pericentrin Pcp1	0.76	1.87	1.83
SPBC365.05c	*slu7*	splicing factor Slu7		0.85	1.97	2.09

SPAC1783.06c	*atg12*	autophagy associated protein Atg12	autophagy associated protein Atg12	0.91	2.09	2.07
SPBP4H10.12		conserved protein (fungal and bacterial)		1.00	4.45	3.70
SPAC19G12.09		NADH/NADPH dependent indole-3-acetaldehyde reductase AKR3C2	NADH/NADPH dependent indole-3-acetaldehyde reductase AKR3C2	1.15	5.03	3.49
SPBC336.08	*spc24*	spindle pole body protein Spc24		0.90	2.43	1.83
SPBC725.13c	*psf2*	GINS complex subunit Psf2		0.80	2.01	1.58
SPBC23G7.06c		conserved eukaryotic protein		0.62	4.32	3.60
SPAC343.06c		phospholipid scramblase	scramblase	0.70	1.87	2.21
SPCC162.02c		AMP-binding dehydrogenase (predicted)	AMP-binding dehydrogenase (predicted)	0.96	5.05	7.72
SPCC569.03		DUF1773 family protein 4	DUF1773 family protein 4	0.65	4.48	2.13
SPBPB7E8.02		PSP1 family protein		1.03	3.99	6.08
SPBC725.03		conserved fungal protein		1.45	18.38	5.29
SPAC4H3.04c		UPF0103 family	UPF0103 family	0.49	17.26	19.52
SPCC1183.03c		frataxin homolog	frataxin homolog	0.82	1.64	1.83
SPAC1002.05c	*jmj2*	histone demethylase Jmj2 (predicted)	histone demethylase Jmj2 (predicted)	0.83	4.29	4.18
SPAC1D4.03c	*aut12*	autophagy associated protein Aut12 (predicted)	autophagy associated protein Aut12	0.88	2.57	2.46
SPAC17A2.15		dubious	dubious	0.51	1.53	4.99
SPAC4D7.02c		glycerophosphoryl diester phosphodiesterase (predicted)	glycerophosphoryl diester phosphodiesterase (predicted)	0.74	5.32	4.89
SPAC20G4.03c	*hri1*	eIF2 alpha kinase Hri1	eIF2 alpha kinase Hri1	0.92	5.40	8.74
SPAC17H9.19c	*cdt2*	WD repeat protein Cdt2	WD repeat protein Cdt2	1.09	1.62	4.63
SPCC970.11c	*wtf9*	wtf element, Wtf2, pseudo	wtf element, Wtf2, pseudo	1.02	2.52	2.94
SPBC27B12.01c	*mmm1*	Mdm10/Mdm12/Mmm1 complex subunit Mmm1 (predicted)		1.27	3.54	2.21
SPCC622.07		dubious	dubious	0.97	2.01	1.89
SPAPB8E5.04c		Niemann-Pick disease type C2 protein hE1 homolog	phosphatidylglycerol/phosphatidylinositol transfer protein (predicted)	0.92	2.17	2.29
SPCC737.09c	*hmt1*	ATP-binding cassette-type vacuolar membrane transporter Hmt1		1.21	3.02	2.43
SPAC1687.22c	*puf3*	RNA-binding protein Puf3 (predicted)		0.73	2.94	2.95
SPBP35G2.14		RNA-binding protein		0.88	1.74	2.24
SPBC3H7.13		FHA domain protein Far10 (predicted)		0.90	1.68	1.69
SPAC16A10.06c	*nse2*	Smc5-6 complex non-SMC subunit 2	Smc5-6 complex non-SMC subunit 2 (PMID 12966087)	0.92	2.05	2.03
SPBC12C2.10c	*pst1*	Clr6 histone deacetylase complex subunit Pst1		0.82	1.66	1.91

SPAC6B12.03c		HbrB family protein	HbrB family protein	0.52	64.14	97.09
SPAC4G9.12		gluconokinase	gluconokinase	1.13	3.76	3.56
SPAC23H3.03c		nitrogen permease regulator family	nitrogen permease regulator family	0.81	1.58	1.74
SPAC25G10.01		RNA-binding protein	RNA-binding protein	1.39	2.88	2.05
SPBC405.05		sequence orphan		1.01	2.01	1.86
SPAC1D4.12	*rad15*	transcription factor TFIIH complex subunit Rad15	transcription factor TFIIH complex subunit Rad15	0.83	1.76	2.01
SPAC17C9.11c		zinc finger protein, zf-C2H2 type/UBA domain protein	zinc finger protein	0.95	3.84	2.58
SPCC569.06		S. pombe specific multicopy membrane protein family 1	S. pombe specific multicopy membrane protein family 1	0.85	2.22	2.26
SPCC285.09c	*cgs2*	cAMP-specific phosphodiesterase Cgs2	cAMP-specific phosphodiesterase Cgs2	1.02	2.13	1.98
SPAC2G11.04		RNA-binding protein, G-patch type (predicted)	RNA-binding protein (predicted)	1.03	1.63	1.98
SPCC1235.04c		FAD synthetase	FAD synthetase	0.97	1.76	2.18
SPBC776.06c		spindle pole body interacting protein (predicted)		0.78	1.51	1.93
SPCC285.04		transthyretin (predicted)	transthyretin (predicted)	0.76	3.10	3.66
SPAC4F8.01	*did4*	vacuolar sorting protein Did4		0.90	1.71	1.59
SPCC162.12	*tco89*	TORC1 subunit Tco89	sequence orphan	0.86	2.17	2.52
SPAC15E1.10		PI31 proteasome regulator related		0.78	4.40	4.21
SPCC1183.09c	*pmp31*	plasma membrane proteolipid Pmp31	plasma membrane proteolipid Pmp31	1.04	2.14	2.04
SPAC17A2.09c	*csx1*	RNA-binding protein Csx1	RNA-binding protein Csx1	0.70	1.60	2.84
SPAC6F12.03c	*fsv1*	SNARE Fsv1	SNARE Fsv1	1.03	2.98	2.45
SPAP14E8.04	*oma1*	metallopeptidase Oma1 (predicted)	metallopeptidase Oma1 (predicted)	1.27	3.09	2.23
SPBC1348.12		zinc finger protein		1.25	2.50	3.26
SPAC19A8.02		pleckstrin homology domain protein	transcriptional coactivator (predicted)	0.90	2.17	2.62
SPAC16E8.09	*scd1*	RhoGEF Scd1	RhoGEF Scd1	0.83	1.56	1.89
SPAC29E6.09		sequence orphan	sequence orphan	0.90	3.22	2.62
SPBC947.15c		NADH dehydrogenase		0.93	3.82	2.68
SPBC16G5.07c		prohibitin (predicted)		0.97	2.29	1.82
SPAC12G12.12		NST UDP-galactose transporter (predicted)	NST UDP-galactose transporter	0.90	2.42	1.52
SPAC824.05	*vps16*	HOPS complex subunit Vps16 (predicted)	HOPS complex subunit Vps16 (predicted)	1.05	2.13	1.84
SPBC21C3.20c	*git1*	C2 domain protein Git1		0.80	1.67	2.49
SPBC1289.15		glycoprotein (predicted)		1.20	1.86	1.75

SPAC513.02		phosphoglycerate mutase family	phosphoglycerate mutase family	0.88	16.35	3.41
SPBC1685.14c		Vid27 family protein	Vid27 family protein	1.12	3.41	3.31
SPAC57A10.06	*mug15*	sequence orphan	sequence orphan	1.44	1.96	1.66
SPAC4F8.10c	*stg1*	SM22/transgelin-like actin modulating protein Stg1	SM22/transgelin-like actin modulating protein Stg1	0.94	1.75	1.62
SPAC806.04c		DUF89 family protein	DUF89 family protein	1.00	2.18	1.54
SPAC23C4.07	*tht2*	meiotically upregulated gene Mug22	meiotically upregulated gene Mug22	0.79	1.90	3.00
SPAC3C7.13c		glucose-6-phosphate 1-dehydrogenase (predicted)	glucose-6-phosphate 1-dehydrogenase (predicted)	0.77	17.22	6.90
SPCC645.02		conserved protein (fungal and plant)	conserved protein (fungal and plant)	1.00	3.20	2.12
SPAC19B12.08		peptidase family C54	peptidase family C54	1.31	6.81	4.38
SPBC1105.14	*rsv2*	transcription factor Rsv2		1.01	4.86	4.06
SPAC24C9.08		vacuolar carboxypeptidase (predicted)	vacuolar carboxypeptidase (predicted)	0.75	3.45	2.45
SPAC5H10.02c		ThiJ domain protein	ThiJ domain protein	1.31	33.31	2.47
SPAC22F3.05c	*alp41*	ADP-ribosylation factor Alp41	ADP-ribosylation factor Alp41	1.03	2.49	1.93
SPCC965.11c		amino acid transporter (predicted)	amino acid transporter (predicted)	0.87	2.02	1.60
SPAC1527.02	*sft2*	Golgi transport protein Sft2 (predicted)	Golgi transport protein Sft2 (predicted)	1.04	1.51	1.83
SPBC31F10.10c		zf-MYND type zinc finger protein		0.92	2.26	3.14
SPAC29A4.06c		human CCDC55 homolog	human CCDC55 homolog	0.92	2.15	1.79
SPAC23A1.14c		uncharacterised trans-sulfuration enzyme (predicted)	cystathionine gamma-synthase (predicted)	0.98	3.48	3.04
SPAC1002.17c	*urg2*	uracil phosphoribosyltransferase (predicted)	uracil phosphoribosyltransferase (predicted)	0.24	2.25	3.18
SPBC15D4.02		transcription factor, zf-fungal binuclear cluster type (predicted)		1.23	1.62	2.98
SPAC17G6.04c	*cpp1*	protein farnesyltransferase beta subunit Cpp1	protein farnesyltransferase beta subunit Cpp1	0.92	2.02	1.91
SPAC222.13c		6-phosphofructo-2-kinase (predicted)	6-phosphofructo-2-kinase (predicted)	0.98	1.81	1.83
SPBC16E9.03c		DUF1783 family protein		1.17	2.64	1.73
SPBC776.18c	*pmh1*	transcription factor TFIIH complex subunit Pmh1		0.84	1.69	1.65
SPCC794.01c		glucose-6-phosphate 1-dehydrogenase (predicted)	glucose-6-phosphate 1-dehydrogenase (predicted)	0.44	15.14	38.16
SPAPB1A10.02		chromosome segregation protein (predicted)	chromosome segregation protein (predicted)	0.99	1.70	1.81
SPBC27B12.04c		conserved eukaryotic protein		0.76	2.55	2.94
SPBC8D2.01	*gsk31*	serine/threonine protein kinase Gsk31		0.99	2.17	1.94
SPAC3A11.14c	*pkl1*	kinesin-like protein Pkl1	kinesin-like protein Pkl1	0.94	2.10	1.73

SPCC1739.10	*mug33*	conserved fungal protein	conserved fungal protein	1.07	1.60	1.68
SPBC21D10.06c	*map4*	cell agglutination protein Map4		0.84	9.74	30.89
SPAC1002.12c		succinate-semialdehyde dehydrogenase (predicted)	succinate-semialdehyde dehydrogenase (predicted)	1.04	3.99	2.37
SPBC405.02c		sequence orphan		0.96	1.87	2.70
SPCC594.04c		steroid oxidoreductase superfamily protein	steroid oxidoreductase superfamily protein	0.83	3.32	2.72
SPAC25B8.19c		transcription factor, zf-fungal binuclear cluster type (predicted)		1.03	2.07	2.62
SPAC32A11.02c		conserved fungal protein	conserved fungal protein	0.83	10.84	3.21
SPBPB2B2.10c		galactose-1-phosphate uridylyltransferase (predicted)		0.81	12.08	9.67
SPBC16H5.13		WD repeat protein, human WDR7 ortholog		0.99	1.83	1.72
SPBC3B8.10c		NLI interacting factor family		0.79	1.94	3.13
SPAC458.04c		sequence orphan	sequence orphan	0.62	9.40	9.91
SPAC1F7.12	*yak3*	aldose reductase YakC		0.95	5.54	3.69
SPAC343.17c		WD repeat protein, human WDR70 family	WD repeat protein, human WDR70 family	0.81	1.52	1.55
SPBC1604.18c		vacuolar sorting protein (predicted)		0.80	1.87	2.35
SPBC354.13	*rga6*	GTPase activating protein Rga6	GTPase activating protein Rga6	1.00	1.75	1.75
SPAC13C5.06c	*mug121*	sequence orphan	sequence orphan	0.74	2.83	2.62
SPCC364.04c		CASP family protein	CASP family protein	0.98	1.59	1.69
SPCC1442.01	*ste6*	guanyl-nucleotide exchange factor Ste6	guanyl-nucleotide exchange factor Ste6	0.98	7.40	15.76
SPBC32H8.07	*git5*	heterotrimeric G protein beta subunit Git5		0.79	4.15	6.45
SPBC365.20c		nicotinamidase (predicted)		1.09	2.29	2.09
SPAC25A8.02		sequence orphan	sequence orphan	0.96	3.25	2.95
SPAC343.03	*apc11*	anaphase-promoting complex subunit Apc11	anaphase-promoting complex subunit Apc11	1.12	2.03	1.87
SPAC9.13c	*cwf16*	splicing factor (predicted)		0.89	2.05	2.29
SPAC1B3.15c		membrane transporter	membrane transporter	0.85	1.96	1.76
SPCC1919.01	*ppk34*	serine/threonine protein kinase Ppk34	serine/threonine protein kinase Ppk34	0.86	2.65	2.24
SPAC4F10.16c		P-type ATPase	P-type ATPase (PMID 12707717)	0.63	2.79	2.87
SPBC29A10.02	*spo5*	meiotic RNA-binding protein 1	meiotic RNA-binding protein 1	0.88	1.58	4.66
SPAPB1A10.07c		sphingolipid biosynthesis protein	sphingolipid biosynthesis protein	0.90	2.01	2.50
SPAC2G11.13	*atg22*	autophagy associated protein Atg22 (predicted)	autophagy associated protein Atg22 (predicted)	1.02	6.29	4.38

SPBC16C6.04		sequence orphan		0.77	2.02	2.53
SPBC3H7.03c		2-oxoglutarate dehydrogenase (lipoamide) (e1 component of oxoglutarate dehydrogenase complex) (predicted)		0.94	2.28	2.00
SPBC30D10.03c		IMP 5'-nucleotidase (predicted)		1.03	1.76	1.62
SPAC19A8.03		phosphatidylinositol-3-phosphatase (predicted)	phosphatidylinositol-3-phosphatase (predicted)	0.78	1.86	2.57
SPBC8E4.05c		fumarate lyase superfamily		0.62	24.32	3.46
SPAC11E3.14		conserved protein	conserved protein	0.77	4.14	4.10
SPBC32H8.02c	*nep2*	NEDD8 protease Nep2		0.88	3.02	3.74
SPAC14C4.01c		DUF1770 family protein	DUF1770 family protein	1.17	12.66	8.58
SPCC1739.04c		sequence orphan	sequence orphan	0.88	2.12	1.99
SPCC285.07c	*wtf18*	wtf element Wtf18	wtf element Wtf18	1.03	4.91	4.83
SPBC3B9.10	*vti1*	SNARE Vti1		1.01	1.59	1.94
SPBC337.02c		pseudogene		0.88	2.13	1.96
SPCC4B3.16	*tip41*	TIP41-like type 2a phosphatase regulator Tip41	TIP41-like type 2a phosphatase regulator Tip41	0.98	1.88	2.25
SPAC6F12.02	*rst2*	transcription factor Rst2	transcription factor Rst2	1.09	2.67	2.30
SPCC338.18		sequence orphan	sequence orphan	0.89	15.60	12.11
SPBC1773.06c		alcohol dehydrogenase (predicted)	alcohol dehydrogenase (predicted)	0.73	3.47	2.34
SPAC25B8.08		conserved fungal family	conserved fungal family	0.81	2.44	3.35
SPCC1795.01c	*mad3*	mitotic spindle checkpoint protein Mad3	mitotic spindle checkpoint protein Mad3	0.80	1.66	1.63
SPAC29B12.13		carbon-sulfur lyase (predicted)	carbon-sulfur lyase (predicted)	1.10	8.45	3.67
SPAC589.08c	*dam1*	DASH complex subunit Dam1	DASH complex subunit Dam1	1.09	2.42	1.56
SPBC725.10		tspO homolog		0.59	7.60	9.22
SPAC2C4.17c		MS ion channel protein 2	MS ion channel protein 2	0.95	4.03	4.57
SPAC22G7.08	*ppk8*	serine/threonine protein kinase Ppk8 (predicted)	serine/threonine protein kinase Ppk8 (predicted)	0.67	3.47	3.56
SPAPJ760.02c	*app1*	App1 protein	App1 protein	0.84	1.50	1.65
SPBC215.03c	*csn1*	COP9/signalosome complex subunit Csn1		0.89	1.91	1.56
SPAC16A10.08c	*mug74*	sequence orphan		0.97	2.15	2.30
SPCC663.03	*pmd1*	leptomycin efflux transporter Pmd1	leptomycin efflux transporter Pmd1	1.39	1.68	1.54
SPBC20F10.05		DuF1740 family protein		0.87	1.74	1.68
SPAC17G6.12	*cul1*	cullin 1	cullin 1	1.02	2.31	1.95

SPBC17A3.03c		phosphoprotein phosphatase (predicted)		0.99	2.31	1.62
SPAC1687.09		ENTH domain protein (predicted)	conserved fungal protein	0.95	1.79	1.68
SPBC651.02		nitrilase (predicted)		1.11	2.26	1.86
SPACUNK4.08		dipeptidyl aminopeptidase (predicted)	dipeptidyl aminopeptidase (predicted)	0.97	2.19	2.50
SPAC2F3.16		ubiquitin-protein ligase E3 (predicted)	ubiquitin-protein ligase E3 (predicted)	1.09	3.51	2.33
SPCC576.04		bax inhibitor-like protein	bax inhibitor-like protein	1.03	3.66	3.04
SPAPB15E9.02c		dubious	dubious	0.79	1.69	2.35
SPBC887.15c		sphingosine hydroxylase (predicted)		1.14	1.99	2.27
SPCC548.03c	*wtf4*	wtf element Wtf4, pseudo	wtf element Wtf4, pseudo	0.17	6.70	34.45
SPAC4D7.11		conserved fungal protein	conserved fungal protein	1.07	1.98	2.21
SPAC20H4.11c	*rho5*	Rho family GTPase Rho5	Rho family GTPase Rho5	1.07	10.67	9.76
SPBC32F12.15	*tfb5*	transcription factor TFIIH complex subunit Tfb5 (predicted)	transcription factor TFIIH complex subunit Tfb5 (predicted)	1.22	1.97	1.83
SPAC22F3.12c	*rgs1*	regulator of G-protein signaling Rgs1	regulator of G-protein signaling Rgs1	1.20	14.48	17.46
SPBC23E6.09	*ssn6*	transcriptional corepressor Ssn6		0.70	2.35	3.18
SPAC2F7.06c	*pol4*	DNA polymerase X family	DNA polymerase X family	0.39	22.72	45.09
SPAC13A11.04c	*ubp8*	ubiquitin C-terminal hydrolase Ubp8 (predicted)	ubiquitin C-terminal hydrolase Ubp8	0.94	3.51	2.96
SPBC1709.14		peptide N-glycanase (predicted)		0.98	2.10	2.03
SPBP8B7.26		sequence orphan		0.90	2.15	2.01
SPCC63.08c	*ppk36*	serine/threonine protein kinase Ppk36	serine/threonine protein kinase Ppk36	0.85	3.25	3.19
SPCC1672.06c	*asp1*	inositol hexakisphosphate kinase/inositol pyrophosphate synthase (predicted)	inositol hexakisphosphate kinase (predicted)	0.87	1.97	2.66
SPAC19G12.02c	*pms1*	MutL family mismatch-repair protein Pms1	MutL family mismatch-repair protein Pms1	1.10	2.53	1.60
SPAC20H4.09		ATP-dependent RNA helicase, spliceosomal (predicted)	ATP-dependent RNA helicase, spliceosomal (predicted)	1.09	1.78	1.62
SPCC1672.04c		mitochondrial copper chaperone	mitochondrial copper ion transport protein	1.04	2.73	2.04
SPAC823.09c		L-asparaginase (predicted)	L-asparaginase (predicted)	1.35	3.06	1.60
SPBC13E7.02	*cwf24*	zf-C3HC4 type (RING finger)/GCN5-related N acetyltransferase fusion protein		0.60	1.54	2.20
SPBC543.04		UPF0171 family protein		0.76	2.05	1.76
SPBC725.11c	*php2*	CCAAT-binding factor complex subunit Php2		1.12	2.42	3.03

SPAC6B12.07c		ubiquitin-protein ligase E3 (predicted)	ubiquitin-protein ligase E3 (predicted)	0.78	1.61	1.68
SPAC4H3.03c		glucan 1,4-alpha-glucosidase (predicted)	glucan 1,4-alpha-glucosidase (predicted)	0.49	40.20	65.44
SPBC16A3.17c		membrane transporter		0.79	3.77	2.04
SPAC458.03	*tel2*	Tel2/Rad-5/Clk-2 family protein Tel2	nuclear telomere cap complex subunit (predicted)	0.78	1.88	1.87
SPCC4G3.03		WD repeat protein	WD repeat protein	0.87	6.72	5.92
SPBC19F5.01c	*puc1*	cyclin Puc1		0.71	4.11	3.80
SPBC1271.08c		sequence orphan	sequence orphan	1.41	24.12	2.79
SPBC25H2.03		vacuolar protein involved in phosphoinositide metabolism		1.00	2.20	2.51
SPAPB24D3.04c	*mag1*	DNA-3-methyladenine glycosylase Mag1	DNA-3-methyladenine glycosylase Mag1	0.89	4.67	2.92
SPAC26H5.04		vacuolar import and degradation protein Vid28	vacuolar import and degradation protein Vid28	0.71	3.33	3.25
SPBC15D4.01c		kinesin-like protein		1.14	2.54	1.82
SPAC1B3.04c		mitochondrial GTPase Guf1 (predicted)	mitochondrial GTPase Guf1 (predicted)	1.11	1.83	1.62
SPAC1296.04	*mug65*	spore wall assembly protein (predicted)	spore wall assembly protein (predicted)	0.79	1.83	2.18
SPAC8C9.16c	*mug63*	TLDc domain protein 1	TLDc domain protein 1	0.75	3.77	3.97
SPCC61.03		UPF0031 family protein	conserved protein (broad species distribution)	1.15	3.55	1.90
SPAC10F6.11c		autophagy associated kinase activator (predicted)	kinase activator (predicted)	0.99	2.36	1.94
SPAC14C4.10c		Nudix family hydrolase	Nudix family hydrolase	1.02	2.68	2.09
SPCC1393.07c	*mug4*	sequence orphan	sequence orphan	1.17	1.97	5.57
SPCC16A11.08	*atg20*	sorting nexin Atg20	sorting nexin Atg20	1.26	5.88	3.85
SPMIT.08		mitochondrial ribosomal small subunit		0.98	3.41	2.78
SPBC1778.02	*rap1*	telomere binding protein Rap1		0.79	1.85	2.53
SPBC19F8.03c		clathrin binding protein		1.10	1.57	1.67
SPBC11G11.01	*fis1*	mitochondrial fission protein Fis1 (predicted)	mitochondrial fission protein Fis1 (predicted)	1.15	2.03	1.75
SPCC1827.07c		SPX/EXS domain protein	SPX/EXS domain protein	1.05	2.58	1.98
SPAC24C9.07c	*bgs2*	1,3-beta-glucan synthase subunit Bgs2	1,3-beta-glucan synthase subunit Bgs2	1.01	1.92	2.55
SPBC16C6.02c	*vps1302*	chorein homolog		0.86	2.23	2.59
SPBC146.12	*coq6*	monooxygenase Coq6		0.92	1.82	1.78
SPBC25B2.10		Usp (universal stress protein) family protein		0.94	2.96	2.15

SPAC922.07c		aldehyde dehydrogenase (predicted)	aldehyde dehydrogenase (predicted)	0.81	5.05	1.68
SPAC977.04		pseudogene	pseudogene	0.72	1.55	2.18
SPBC725.02	*mpr1*	response regulator phosphotransferase		1.13	2.27	2.13
SPAC25B8.13c	*isp7*	2-OG-Fe(II) oxygenase superfamily protein	2-OG-Fe(II) oxygenase superfamily protein	0.54	8.46	3.47
SPCC584.02	*cuf2*	Cu metalloregulatory transcription factor Cuf2	Cu metalloregulatory transcription factor Cuf2	0.71	2.92	2.78
SPBC3H7.06c	*pof9*	F-box protein Pof9		1.24	3.14	3.38
SPAC1565.03		sequence orphan	sequence orphan	0.77	3.65	6.94
SPAPB18E9.02c	*ppk18*	serine/threonine protein kinase Ppk18 (predicted)	serine/threonine protein kinase Ppk18 (predicted)	0.88	2.20	2.44
SPAC16C9.01c		carbohydrate kinase (predicted)	carbohydrate kinase (predicted)	1.24	3.90	1.88
SPBC691.01		palmitoyltransferase (predicted)		0.77	1.84	2.43
SPAC1039.11c		alpha-glucosidase	alpha-glucosidase	1.11	1.56	2.05
SPBC21C3.12c		DUF953 family protein		0.96	1.53	1.65
SPCC663.17	*wtf15*	wtf element Wtf15, pseudo	wtf element Wtf15, pseudo	0.78	2.31	3.64
SPCC31H12.06	*mug111*	sequence orphan	sequence orphan	0.94	2.25	2.63
SPBC660.08		sequence orphan	sequence orphan	1.20	2.02	1.81
SPAC1782.12c		DUF423 protein	conserved protein (broad species distribution)	0.93	4.61	3.67
SPBPB2B2.12c		UDP-glucose 4-epimerase		1.33	16.93	5.67
SPBC25B2.02c	*mam1*	M-factor transporter Mam1		0.96	2.97	3.22
SPAC3A11.09	*sod22*	plasma membrane alkali metal cation/H+ antiporter Sod22	plasma membrane alkali metal cation/H+ antiporter Sod22	1.07	1.72	1.51
SPBC713.07c		vacuolar polyphosphatase (predicted)		0.82	2.28	1.80
SPCC550.10	*meu8*	aldehyde dehydrogenase Meu8 (predicted)	betaine aldehyde dehydrogenase (predicted)	0.91	2.10	2.73
SPCC1442.04c		conserved fungal protein	conserved fungal protein	0.88	1.78	2.48
SPBC336.12c	*cdc10*	MBF transcription factor complex subunit Cdc10		0.94	2.00	2.49
SPBC27B12.05		WD repeat protein		0.85	1.92	2.41
SPAC23H4.01c		sterol binding ankyrin repeat protein	sterol binding ankyrin repeat protein	0.92	1.73	2.34
SPAC11E3.05		ubiquitin-protein ligase E3	ubiquitin-protein ligase E3	0.76	2.13	2.67
SPBC146.01	*med15*	mediator complex subunit Med15 (predicted)	mediator complex subunit Med15 (predicted)	0.95	2.44	1.66
SPAC16E8.17c		succinate-CoA ligase alpha subunit	succinate-CoA ligase (alpha subunit)	1.02	1.63	1.70
SPAC664.15		CCR4-Not complex subunit Caf4/Mdv1 (predicted)	CCR4-Not complex subunit Caf4/Mdv1 (predicted)	0.84	2.96	2.28

SPAC17G8.10c	*dma1*	mitotic spindle checkpoint protein Dma1	mitotic spindle checkpoint protein Dma1	1.12	2.83	2.41
SPBC56F2.05c		transcription factor (predicted)		0.71	1.89	2.53
SPCC1682.11c		DUF580 family protein	DUF580 family protein	0.90	3.16	3.36
SPCC417.11c		glutamate-1-semialdehyde 2,1-aminomutaseaminotransferase (predicted)	glutamate-1-semialdehyde 2,1-aminomutaseaminotransferase (predicted)	1.43	2.47	2.51
SPAC1834.11c	*sec18*	secretory pathway protein Sec18 (predicted)	secretory pathway protein Sec18 (predicted)	0.94	1.59	1.68
SPMIT.04	*cox3*	cytochrome c oxidase 3	cytochrome c oxidase 3; similar to S. cerevisiae Q0275	0.96	5.38	5.52
SPCC24B10.14c	*xlf1*	xrcc4 like factor	xrcc4 like factor	0.58	2.77	5.69
SPAC16A10.01		DUF1212 family protein	DUF1212 family protein	1.11	2.83	2.68
SPBP8B7.18c		phosphomethylpyrimidine kinase (predicted)		1.04	3.09	3.57
SPCC417.07c	*mto1*	MT organizer Mto1	MT organizer Mto1	0.89	1.78	1.94
SPAC1565.07c		human CAND1/TIP120 ortholog	TATA binding protein interacting protein (predicted)	0.89	1.68	2.04
SPBC713.06	*adl1*	DNA ligase (predicted)	DNA ligase (predicted)	0.87	3.34	3.79
SPBC1711.12		serine protease (predicted)		0.94	1.62	1.51
SPAC7D4.05		hydrolase (predicted)	hydrolase (predicted)	1.07	2.23	1.82
SPCC320.05		sulphate transporter (predicted)	sulphate transporter (predicted)	1.00	4.09	2.42
SPAC458.06		phosphoinositide binding protein	phosphoinositide binding protein	0.95	1.78	1.97
SPBP35G2.06c	*nup131*	nucleoporin Nup131		0.79	2.27	2.01
SPCC1450.12		conserved fungal protein	conserved fungal protein	0.94	2.61	2.21
SPCC70.02c		mitochondrial ATPase inhibitor (predicted)	mitochondrial ATPase inhibitor (predicted)	0.95	3.07	2.03
SPMIT.05	*cob1*	cytochrome b, Cob1	cytochrome b; respiratory chain complex III; similar to S. cerevisiae Q0105	0.52	1.57	3.13
SPAC29A4.12c	*mug108*	sequence orphan	sequence orphan	0.95	11.15	38.79
SPBC1289.16c		copper amine oxidase (predicted)		0.67	25.57	13.15
SPAC2F3.07c		sequence orphan	sequence orphan	0.62	3.47	3.05
SPAC644.08		haloacid dehalogenase-like hydrolase	haloacid dehalogenase-like hydrolase	1.06	2.32	1.65
SPAC637.13c		cytoskeletal signaling protein	cytoskeletal signaling protein	0.86	1.67	1.68
SPAC2E1P3.05c		fungal cellulose binding domain protein	fungal cellulose binding domain protein	0.68	5.98	3.13
SPBC1685.01	*pmp1*	dual-specificity MAP kinase phosphatase Pmp1	dual-specificity MAP kinase phosphatase Pmp1	0.94	1.83	2.29

SPCC622.03c		dubious	dubious	1.21	4.13	3.51
SPBC577.05c	*rec27*	meiotic recombination protein Rec27		0.57	1.67	5.42
SPBC365.02c	*cox10*	protoheme IX farnesyltransferase		1.03	2.47	1.60
SPAC19G12.04		ureidoglycolate hydrolase (predicted)	ureidoglycolate hydrolase (predicted)	1.00	9.40	1.96
SPBC3B9.06c	*apg3*	autophagy associated protein Apg3 (predicted)	autophagy associated protein Apg3 (predicted)	1.06	3.85	2.38
SPBC16A3.09c	*ufd1*	Cdc48-Ufd1-Npl4 complex component Ufd1 (predicted)		0.70	1.79	1.85
SPCC757.03c		ThiJ domain protein	ThiJ domain protein	0.55	18.51	10.23
SPBC1198.14c	*fbp1*	fructose-1,6-bisphosphatase Fbp1		0.62	31.86	24.82
SPBC1711.02	*matmc_1*	mating-type m-specific polypeptide mc		0.80	2.65	2.47
SPAC23D3.11	*ayr1*	1-acyldihydroxyacetone phosphate reductase (predicted)	1-acyldihydroxyacetone phosphate reductase (predicted)	0.95	5.22	3.14
SPCC794.04c		membrane transporter	membrane transporter	0.71	13.12	17.07
SPCC132.03		sequence orphan	sequence orphan	1.16	4.04	2.62
SPBC354.11c		dubious	sequence orphan	1.40	2.45	2.00
SPBC1683.08	*ght4*	hexose transporter Ght4	hexose transporter Ght4 (PMID 10735857)	1.42	41.18	20.06
SPBC800.13		histone H4 variant	histone H4 variant	0.78	2.04	2.10
SPBC26H8.04c		DEP domain protein		1.00	2.21	2.40
SPCPB16A4.02c		conserved fungal protein	conserved fungal protein	1.02	1.61	1.73
SPAC4G8.03c		RNA-binding protein	RNA-binding protein	0.75	2.83	2.34
SPCC61.05		S. pombe specific multicopy membrane protein family 1	S. pombe specific multicopy membrane protein family 1	0.76	3.13	2.10
SPAC19B12.13	*cox1102*	fusion cytochrome c oxidase assembly protein Cox1102, mitochondrial ribosomal protein Rsm2202		1.06	1.88	1.51
SPAPJ695.01c		S. pombe specific UPF0321 family protein 3	S. pombe specific UPF0321 family protein 3	0.33	7.42	11.10
SPBC19F8.04c		nuclease		0.99	1.57	1.75
SPAC1556.04c	*cdd1*	cytidine deaminase Ccd1	cytidine deaminase Pcd1	0.92	1.87	2.43
SPMIT.06		mitochondrial DNA binding endonuclease		0.51	1.86	4.08
SPBC1709.11c	*png2*	ING family homolog Png2		0.98	3.08	2.35
SPBC660.05		conserved fungal protein	conserved fungal protein	0.74	8.29	11.09
SPBC83.03c	*tas3*	RITS complex subunit 3		0.95	2.09	1.58
SPCC1620.08		succinate-CoA ligase (beta subunit)	succinate-CoA ligase (beta subunit)	0.95	2.13	1.77

SPBP23A10.14c	*ell1*	RNA polymerase II transcription elongation factor SpELL		0.85	2.95	2.52
SPAC823.16c	*mug179*	WD repeat protein Mug179	WD repeat protein Mug179	0.81	2.50	3.46
SPAC328.07c		heavy metal ion homeostasis protein (predicted)	heavy metal ion homeostasis protein (predicted)	1.14	2.35	2.13
SPAC19G12.11	*coq9*	ubiquinone biosynthesis protein Coq9 (predicted)	ubiquinone biosynthesis protein Coq9 (predicted)	0.90	1.79	1.84
SPAC139.05		succinate-semialdehyde dehydrogenase (predicted)	succinate-semialdehyde dehydrogenase (predicted)	0.78	51.13	7.67
SPAC2G11.09		DUF221 family protein	DUF221 family protein	0.93	2.14	1.93
SPBC577.13	*syj2*	inositol-polyphosphate 5-phosphatase (synaptojanin homolog 2)		0.86	4.17	3.19
SPBC609.01		ribonuclease II (RNB) family		1.07	2.53	2.65
SPBC2D10.17	*clr1*	cryptic loci regulator Clr1		0.89	2.73	3.01
SPAC22A12.01c	*pso2*	DNA 5' exonuclease (predicted)	DNA 5' exonuclease (predicted)	0.81	1.56	2.24
SPAC18G6.01c		conserved fungal protein	conserved fungal protein	1.16	3.51	4.45
SPBC12C2.12c	*glo1*	glyoxalase I		1.09	3.02	2.42
SPBC4.01		sequence orphan		0.79	52.18	86.50
SPAC1687.07		conserved fungal protein	conserved fungal protein	1.11	3.12	2.26
SPBC405.04c	*ypt7*	GTPase Ypt7		0.94	2.12	1.89
SPCC330.01c	*rhp16*	Rad16 homolog Rhp16	Rad16 homolog Rhp16	0.93	2.22	2.14
SPBC1271.01c	*pof13*	F-box protein Pof13	F-box protein Pof13	0.64	2.28	3.42
SPAC869.07c	*mel1*	alpha-galactosidase	alpha-galactosidase (PMID 15580593)	1.13	76.27	37.51
SPCC576.01c		sulfonate dioxygenase (predicted)	sulfonate dioxygenase (predicted)	1.00	41.89	4.37
SPAC959.05c		protein disulfide isomerase (predicted)	protein disulfide isomerase (predicted)	0.93	1.82	1.59
SPBC2A9.03		conserved protein (fungal and plant)		0.83	1.58	2.08
SPAPB1A11.03		FMN dependent dehydrogenase	FMN dependent dehydrogenase	0.29	12.92	3.54
SPBPB21E7.06		pseudogene	pseudogene	0.52	15.21	6.00
SPAC869.01		amidase (predicted)	amidase (predicted)	1.28	2.16	1.66
SPCC188.07	*ccq1*	telomere maintenence protein	telomere maintenence protein	0.78	1.97	2.30
SPAC227.03c		mitochondrial NAD+ transporter (predicted)	mitochondrial NAD+ transporter	0.65	2.33	2.09
SPAC3A12.06c		sodium/calcium exchanger (predicted)	sodium/calcium exchanger (predicted)	0.90	1.55	1.70
SPAC458.05	*pik3*	phosphatidylinositol 3-kinase Pik3	phosphatidylinositol 3-kinase Pik3	0.97	2.31	2.09
SPCC1739.15	*wtf21*	wtf element Wtf21	wtf element Wtf21	1.04	4.27	4.28
SPAC23D3.13c		guanyl-nucleotide exchange factor (predicted)	guanyl-nucleotide exchange factor	0.91	1.65	2.38
SPCP31B10.02		conserved eukaryotic protein	conserved eukaryotic protein	0.96	1.88	1.73

SPCC330.04c	*mug135*	DUF1773 family protein 3	DUF1773 family protein 3	0.83	2.99	2.66
SPAC1006.01	*psp3*	serine protease Psp3 (predicted)	serine protease Psp3 (predicted)	1.12	2.60	1.99
SPAC6G10.10c		human hmmtag2 homolog	human hmmtag2 homolog	0.92	1.75	2.33
SPAC22E12.06c	*gmh3*	alpha-1,2-galactosyltransferase Gmh3	alpha-1,2-galactosyltransferase Gmh3	1.00	2.22	2.36
SPBC16C6.03c		conserved fungal protein		0.70	2.43	2.51
SPCC70.04c		sequence orphan	sequence orphan	1.45	7.94	9.02
SPAC23C4.18c	*rad4*	BRCT domain protein Rad4	BRCT domain protein Rad4	0.92	1.58	1.83
SPAC3H5.04	*aar2*	U5 snRNP-associated protein Aar2	U5 snRNP-associated protein Aar2	0.81	1.55	1.78
SPBC1711.11		autophagy associated protein (predicted)		1.09	7.59	4.72
SPCC1682.15	*mug122*	PX/PXA domain protein	PX/PXA domain protein	0.83	2.11	1.90
SPBC646.15c		Pex16 family peroxisome import protein		0.67	1.57	1.87
SPBPB2B2.13		galactokinase Gal1 (predicted)		0.98	6.72	4.54
SPBC16A3.18	*cip1*	RNA-binding protein Cip1		1.01	1.91	2.11
SPBC83.01	*ucp8*	UBA/EH/EF hand domain protein Ucp8		0.84	2.06	2.68
SPCC364.02c	*bis1*	stress response protein Bis1	stress response protein Bis1	0.93	1.97	1.52
SPAC1142.08	*fhl1*	fork head transcription factor Fhl1		1.15	2.02	2.02
SPCC126.09		vacuolar membrane zinc transporter (predicted)	vacuolar membrane zinc transporter (predicted)	1.08	2.10	2.20
SPAC57A10.05c	*pof1*	F-box/WD repeat protein protein Pof1	F-box protein Pof1	0.90	2.07	2.00
SPAC227.14		nicotinamide riboside kinase (predicted)	nicotinamide riboside kinase	0.98	2.32	2.13
SPAC19B12.10	*sst2*	human AMSH protein homolog	human amsh protein homolog	0.88	4.16	3.60
SPBC18H10.05		WD repeat protein, human WDR44 family		0.90	4.86	4.08
SPCC1919.03c		AMP-activated protein kinase beta subunit (predicted)	AMP-activated protein kinase beta subunit (predicted)	0.96	2.22	2.20
SPBC409.12c	*stn1*	telomere cap complex subunit Stn1		0.69	2.39	3.04
SPBC28E12.06c	*lvs1*	beige protein homolog		0.90	1.60	1.85
SPBC25B2.03		zf-C3HC4 type zinc finger		0.85	3.12	1.81
SPBC1105.10	*rav1*	RAVE complex subunit Rav1 (predicted)		1.06	2.79	2.17
SPAC31G5.07		conjugation protein (predicted)	conserved fungal protein	0.89	1.52	4.45
SPCC18B5.11c	*cds1*	replication checkpoint kinase Cds1	replication checkpoint kinase Cds1	0.91	2.60	2.27
SPAC1952.09c		acetyl-CoA hydrolase	acetyl-CoA hydrolase	0.80	4.02	1.89
SPCC757.04		transcription factor (predicted)	transcription factor (predicted)	0.79	1.67	1.76
SPCC320.04c		GTPase Gem1	GTPase Gem1 (PMID 12482879)	0.77	1.70	1.87
SPBC30D10.09c		HVA22/TB2/DP1 family protein		1.08	2.48	1.70
SPBP4H10.17c		carboxyl methyl esterase		0.86	2.63	2.09
SPAC1002.19	*urg1*	GTP cyclohydrolase II (predicted)	GTP cyclohydrolase (predicted)	0.38	14.73	23.25
SPBC11C11.06c		sequence orphan		0.77	1.95	2.12

SPBC1D7.02c	*scr1*	transcription factor Scr1		0.89	2.09	3.56
SPBC216.03		conserved fungal protein		0.78	4.37	3.86
SPCC1259.15c	*ubc11*	ubiquitin conjugating enzyme E2-C	ubiquitin conjugating enzyme E2-C (PMID 12724408)	0.96	1.70	1.58
SPAPB1A11.01		membrane transporter	membrane transporter	1.12	2.61	1.76
SPCC285.10c		SPRY domain protein	SPRY domain protein	0.82	5.21	3.84
SPBC543.05c		inorganic anion exchanger (predicted)		0.85	1.77	1.99
SPAC328.04		AAA family ATPase, unknown biological role	AAA family ATPase, unknown biological role	0.79	2.66	2.36
SPAPB8E5.10		sequence orphan	sequence orphan	0.84	2.34	3.42
SPAC13G7.13c	*msa1*	RNA-binding protein Msa1	RNA-binding protein Msa1	0.64	2.84	3.39
SPACUNK4.10		hydroxyacid dehydrogenase (predicted)	hydroxyacid dehydrogenase (predicted)	1.15	2.55	1.90
SPAC3G6.09c	*tps2*	trehalose-phosphate synthase Tps2 (predicted)	trehalose-phosphate synthase Tps2 (predicted)	0.91	1.63	1.78
SPBPB2B2.06c		phosphoprotein phosphatase (predicted)		1.10	8.40	8.61
SPBC6B1.05c		ubiquitin-like conjugating enzyme		1.03	2.48	2.40
SPCC63.04	*mok14*	alpha-1,3-glucan synthase Mok14	alpha-1,3-glucan synthase Mok14	0.91	1.89	1.91
SPAC29A4.11	*rga3*	GTPase activating protein Rga3	GTPase activating protein Rga3	0.73	1.91	2.83
SPAC167.05		Usp (universal stress protein) family protein, implicated in meiotic chromosome segregation	Usp (universal stress protein) family protein,implicated in meiotic chromosome segregation	1.02	3.74	2.59
SPCC320.03		transcription factor (predicted)	transcription factor (predicted)	0.52	2.49	3.85
SPBC20F10.06	*mad2*	spindle checkpoint protein Mad2		0.91	2.16	2.15
SPCC1494.03	*arz1*	armadillo repeat containing, Zfs1 target number 1	sequence orphan	0.75	2.85	2.60
SPAC20G4.05c		UPF0061 family protein	UPF0061 family protein	0.72	4.20	3.20
SPAC9E9.05		sequence orphan	sequence orphan	1.16	2.67	2.68
SPBC36.10		mitochondrial intermembrane space protein sorting protein		0.91	2.06	2.42
SPBC530.05		transcription factor (predicted)		0.74	2.07	2.51
SPBC15D4.12c	*mug98*	sequence orphan		1.21	2.78	1.56
SPBC12C2.02c	*ste20*	sterility protein Ste20		0.96	1.56	1.73
SPAP8A3.05		ski complex interacting GTPase (predicted)	ski complex subunit Ski7 (predicted)	0.98	1.57	1.53
SPBC16E9.17c	*rem1*	meiosis-specific cyclin Rem1		0.89	5.94	4.69
SPBC21D10.09c		ubiquitin-protein ligase E3 (predicted)		0.97	1.85	1.74
SPAC922.06		short chain dehydrogenase (predicted)	short chain dehydrogenase	1.24	3.37	1.51

SPAC5D6.09c	*mug86*	acetate transporter (predicted)	acetate transporter (predicted)	0.68	5.80	7.61
SPAC27D7.13c	*ssm4*	p150-Glued		0.53	2.13	8.45
SPAC227.15		protein phosphatase regulatory subunit Reg1 (predicted)	protein phosphatase regulatory subunit Reg1 (predicted)	0.96	3.14	3.40
SPAC607.08c		DUF726 family protein	DUF726 family protein	0.93	3.19	3.25
SPCC1183.05c	*lig4*	DNA ligase Lig4	DNA ligase Lig4	1.04	1.78	1.97
SPBC32F12.08c	*duo1*	DASH complex subunit Duo1 (predicted)		0.76	1.85	1.58
SPAC4D7.10c		SAGA complex subunit Spt20 (predicted)	SAGA complex subunit Spt20 (predicted)	0.68	2.15	2.33
SPAC27E2.07	*pvg2*	galactose residue biosynthesis protein Pvg2	galactose residue biosynthesis protein Pvg2	1.21	1.59	2.48
SPAC1F3.09	*mug161*	CwfJ family protein	CwfJ family protein	0.94	2.64	1.54
SPCC1020.10	*oca2*	serine/threonine protein kinase Oca2 (predicted)	serine/threonine protein kinase Oca2 (predicted)	0.62	2.99	3.40
SPBP4H10.16c		phosphatase activator (predicted)		1.20	1.77	1.61
SPBC16D10.05	*mok13*	alpha-1,3-glucan synthase Mok13		1.35	3.19	2.10
SPAC25G10.03	*zip1*	transcription factor Zip1	transcription factor Zip1	1.08	2.55	1.96
SPCC757.11c		membrane transporter	membrane transporter	0.97	3.75	2.14
SPBC16E9.16c	*lsd90*	Lsd90 protein		0.47	22.97	33.93
SPAC20G8.10c		beclin family protein		1.06	3.25	2.12
SPAC22A12.11	*dak1*	dihydroxyacetone kinase Dak1	dihydroxyacetone kinase Dak1	0.90	2.11	2.25
SPBP4H10.07		ubiquitin-protein ligase E3 (predicted)		0.99	2.29	2.50
SPBP35G2.12		nucleoside diphosphate-sugar hydrolase (predicted)		0.81	2.57	1.68
SPBC2F12.05c		sterol binding ankyrin repeat protein		0.95	2.44	2.58
SPBC119.07	*ppk19*	serine/threonine protein kinase Ppk19		0.90	2.69	3.15
SPCC584.03c		RanGTP-binding protein (predicted)	RanGTP-binding protein (predicted)	1.12	1.66	1.71
SPBC18A7.01		X-Pro dipeptidase (predicted)		1.11	5.49	3.03
SPAC22H12.05c		fasciclin domain protein (3)	fasciclin domain protein (3)	0.91	2.71	2.71
SPAC144.13c	*srw1*	CDK inhibitor Srw1	CDK inhibitor Srw1	1.38	4.10	3.33
SPCC285.11	*ucp10*	UBA/UAS domain protein Ucp10	UBA/UAS domain protein Ucp10	1.06	5.37	3.78
SPAC14C4.07		membrane transporter	membrane transporter	0.95	2.15	2.38
SPCC965.06		potassium channel subunit (predicted)	potassium channel subunit (predicted)	0.92	10.30	2.46
SPCC70.10		sequence orphan	sequence orphan	1.44	2.98	1.65
SPBC428.16c	*rhb1*	Rheb GTPase Rhb1	Rheb GTPase Rhb1	0.98	1.56	1.52
SPAC6F12.12	*par2*	protein phosphatase regulatory subunit Par2	protein phosphatase regulatory subunit Par2	0.87	1.69	2.29
SPAC15F9.01c		sequence orphan	sequence orphan	0.92	2.88	5.22

SPBC947.08c		histone promoter control protein Hpc2 (predicted)		0.84	1.58	2.06
SPAC5D6.06c		UDP-GlcNAc transferase associated protein Alg14	UDP-GlcNAc transferase associated protein Alg14	0.91	2.40	2.82
SPCC63.14		conserved fungal protein	conserved fungal protein	0.66	1.98	2.58
SPAC25B8.18		mitochondrial electron carrier (predicted)	mitochondrial electron carrier (predicted)	0.97	3.64	2.36
SPCC18B5.05c		phosphomethylpyrimidine kinase (predicted)	phosphomethylpyrimidine kinase (predicted)	1.28	3.00	2.49
SPAC19A8.05c	*sst4*	sorting receptor for ubiquitinated membrane proteins, ESCRT 0 complex subunit Sst4	sorting receptor for ubiquitinated membrane proteins (ISS) (PMID 12055639)	1.29	4.29	3.25
SPAC6B12.02c	*mus7*	DNA repair protein Mus7/Mms22	DNA repair protein Mus7	0.91	1.85	1.75
SPCC16A11.04	*snx12*	sorting nexin Snx12 (predicted)	sorting nexin Snx12 (predicted)	0.85	1.51	2.03
SPBC4F6.06	*kin1*	microtubule affinity-regulating kinase Kin1		0.92	2.72	2.42
SPAC10F6.04		RCC domain protein Ats1 (predicted)	RCC domain protein Ats1 (predicted)	0.86	2.36	2.48
SPAC6B12.08	*mug185*	DNAJ domain protein Jjj family	DNAJ domain protein Jjj family	0.77	4.04	3.93
SPCC320.06		sequence orphan	sequence orphan	1.12	2.86	1.73
SPAC6C3.08		proteasome regulatory particle, gankyrin (predicted)	gankyrin	1.34	3.09	2.26
SPACUNK12.02c	*cmk1*	calcium/calmodulin-dependent protein kinase Cmk1	calcium/calmodulin-dependent protein kinase Cmk1	1.16	2.06	2.12
SPAC1420.01c		DUF1752 family protein	DUF1752 family protein	0.96	1.70	1.73
SPAC22F3.09c	*res2*	MBF transcription factor complex subunit Res2	MBF transcription factor complex subunit Res2	1.18	1.93	1.67
SPBC21B10.02		sequence orphan		0.86	1.69	1.86
SPAC2F3.08	*sut1*	alpha-glucoside transporter	alpha-glucoside transporter (PMID 11136464)	0.93	4.23	3.95
SPAC1142.03c	*swi2*	Swi5 complex subunit Swi2	Swi5 complex subunit Swi2	0.90	1.69	2.23
SPBC800.02	*whi5*	cell cycle transcriptional repressor Whi5	cell cycle transcriptional repressor Whi5	1.18	1.82	1.72
SPBC1711.17	*prp16*	ATP-dependent RNA helicase Prp16		0.74	1.95	2.94
SPBC800.11		inosine-uridine preferring nucleoside hydrolase (predicted)	inosine-uridine preferring nucleoside hydrolase (predicted)	0.83	3.10	1.61
SPBC685.03		sequence orphan		1.09	2.97	3.76
SPBC23G7.16	*ctr6*	vacuolar copper transporter Ctr6		1.49	4.15	2.00
SPAC17A2.06c	*vps8*	WD repeat protein Vps8	WD repeat protein Vps8	0.83	1.81	2.34

SPBC902.05c	*idh2*	isocitrate dehydrogenase (NAD+) subunit 2	isocitrate dehydrogenase (NAD+) subunit 2	1.04	3.50	2.35
SPAPYUK71.03c		C2 domain protein	C2 domain protein	0.87	2.59	1.76
SPBC1773.05c	*tms1*	hexitol dehydrogenase (predicted)	hexitol dehydrogenase (predicted)	0.72	7.40	4.93
SPAC22A12.17c		short chain dehydrogenase (predicted)	short chain dehydrogenase (predicted)	1.06	28.82	2.70
SPAC56E4.02c	*alg13*	N-acetylglucosaminyldiphosphodolichol N-acetylglucosaminyltransferase Alg13 (predicted)	N-acetylglucosaminyldiphosphodolichol N-acetylglucosaminyltransferase Alg13 (predicted)	0.92	2.72	1.78
SPBC11C11.03	*ndc80*	spindle pole body protein Ndc80		0.99	1.53	1.69
SPAC18B11.05	*gpi18*	pig-V (predicted)	pig-V	0.90	2.18	1.69
SPBC36B7.05c		phosphatidylinositol(3)-phosphate binding protein (predicted)		0.88	3.60	3.07
SPAC2E12.05	*wtf1*	wtf element Wtf1, pseudo		0.99	2.51	3.07
SPBC21B10.12	*rec6*	meiotic recombination protein Rec6		1.04	2.14	3.53
SPBPB2B2.11		nucleotide-sugar 4,6-dehydratase (predicted)		1.03	7.21	3.52
SPAC4H3.08		short chain dehydrogenase (predicted)	short chain dehydrogenase (predicted)	0.60	41.51	3.53
SPCPJ732.03	*meu15*	sequence orphan	sequence orphan	1.20	1.75	2.61
SPAC1F3.02c	*mkh1*	MEK kinase (MEKK) Mkh1	MEK kinase (MEKK) Mkh1 (PMID 9199286)	0.97	2.94	2.69
SPAC869.06c		cation binding protein (predicted)	cation binding protein (predicted)	1.13	49.90	38.85
SPAC12B10.13		CTLH domain	CTLH domain	0.90	3.85	3.27
SPBC2D10.06	*rep1*	MBF transcription factor complex subunit Rep1		0.92	2.00	3.36
SPAC1782.03		microfibrillar-associated protein family protein	microfibrillar-associated protein familt protein	0.81	1.72	1.76
SPCC1919.04		sequence orphan	sequence orphan	0.96	1.93	1.80
SPBC19C2.05	*ran1*	serine/threonine protein kinase Ran1		1.18	5.38	6.88
SPCC338.12		subtilisin related protein (predicted)	protease inhibitor (predicted)	0.74	4.42	3.95
SPBC12D12.04c	*pck2*	protein kinase C (PKC)-like Pck2		0.82	1.77	1.72
SPCC1223.09		uricase (predicted)	uricase (predicted)	0.80	22.52	9.67
SPCC548.07c	*ght1*	hexose transporter Ght1	hexose transporter Ght1 (PMID 10735857)	0.83	73.22	91.65
SPAC1039.05c	*klf1*	conserved fungal protein	conserved fungal protein	1.05	2.56	1.73
SPAC1834.08	*mak1*	histidine kinase Mak1	histidine kinase Mak1	0.86	1.57	1.56
SPCC1235.14	*ght5*	hexose transporter Ght5	hexose transporter Ght5 (PMID 10735857)	0.77	2.40	3.14

SPBC530.07c		phosphomethylpyrimidine kinase (predicted)		1.38	5.19	4.45
SPAC25H1.03	*mug66*	meiotically upregulated gene Mug66	meiotically upregulated gene Mug66	0.72	2.19	3.08
SPAP7G5.06		amino acid permease, unknown 4	amino acid permease, unknown 4	0.66	3.67	2.13
SPAC1805.10		sequence orphan	sequence orphan	1.11	2.27	1.82
SPBC713.02c	*ubp21*	ubiquitin C-terminal hydrolase Ubp21		1.00	2.36	2.01
SPAC11E3.02c		C2 domain protein	C2 domain protein	0.94	2.52	2.61
SPBC1773.03c		aminotransferase class-III, unknown specificty	aminotransferase class-III (predicted)	0.93	5.35	2.08
SPBC839.02		arrestin Aly1 related	arrestin Aly1 related	0.85	2.90	3.16
SPAC11G7.03	*idh1*	isocitrate dehydrogenase (NAD+) subunit 1 Idh1	isocitrate dehydrogenase (NAD+) subunit 1 Idh1 (PMID 10975257)	1.03	3.38	2.48
SPAC4F10.02		aminopeptidase (predicted)	aspartyl aminopeptidase (predicted)	0.97	2.70	2.52
SPAC27F1.05c		aminotransferase class-III, unknown specificty	4-aminobutyrate transaminase	0.72	4.06	5.19
SPAC22H10.02		conserved fungal protein	conserved fungal protein	1.03	2.53	2.55
SPAC1D4.02c		human GRASP protein homolog (predicted)	human GRASP protein homolog (predicted)	1.01	1.83	1.68
SPAC1B3.20		sequence orphan	sequence orphan	1.05	2.70	1.92
SPCC1322.08	*srk1*	MAPK-activated protein kinase Srk1	MAPK-activated protein kinase Srk1	0.77	4.81	4.03
SPCC962.01		C2 domain protein	C2 domain protein	0.72	2.10	2.11
SPAC2C4.07c		ribonuclease II (RNB) family	ribonuclease II (RNB) family	1.03	2.57	1.83
SPAC4G8.04		GTPase activating protein (predicted)	GTPase activating protein (predicted)	0.88	2.96	3.23
SPBC26H8.01	*thi2*	thiazole biosynthetic enzyme		0.96	2.05	1.96
SPAC6C3.04	*cit1*	citrate synthase (predicted)	citrate synthase	1.15	2.22	1.65
SPCC1281.04		pyridoxal reductase (predicted)	pyridoxal reductase (predicted)	0.72	6.42	2.43
SPBC21B10.11	*dpm2*	dolichol-phosphate mannosyltransferase subunit 2 (predicted)		0.91	2.03	2.55
SPBP16F5.03c	*tra1*	phosphatidylinositol kinase-related protein Tra1		0.85	1.83	2.14
SPAC23C4.12	*hhp2*	serine/threonine protein kinase Hhp2	serine/threonine protein kinase Hhp2 (PMID 8026462)	1.02	1.68	1.79
SPBC21.02		TLDc domain protein 2		0.79	2.64	2.79
SPBC106.13		conserved eukaryotic protein	conserved eukaryotic protein	1.04	5.71	4.83
SPCC14G10.04		sequence orphan	sequence orphan	0.89	1.80	1.81
SPAC26F1.01	*sec74*	guanyl-nucleotide exchange factor Sec74		0.73	2.32	3.28
SPAC19D5.01	*pyp2*	tyrosine phosphatase Pyp2	tyrosine phosphatase Pyp2	0.48	4.69	5.15

SPCC4F11.02	*ptc1*	protein phosphatase 2C Ptc1	protein phosphatase 2C Ptc1	1.12	2.55	2.28
SPAC23C11.06c		hydrolase (inferred from context)	hydrolase (inferred from context)	1.04	9.18	4.57
SPCC1223.12c	*meu10*	GPI anchored cell surface protein (predicted)	conserved fungal family	0.71	3.78	3.86
SPBC1289.10c		transcription factor (predicted)		0.90	2.14	2.01
SPBC6B1.02	*ppk30*	Ark1/Prk1 family protein kinase Ppk30		0.78	3.38	4.61
SPBC211.07c	*ubc8*	ubiquitin conjugating enzyme Ubc8		1.14	3.39	1.99
SPAC3H1.11	*hsr1*	transcription factor Hsr1	transcription factor Hsr1	0.77	2.34	2.17
SPBC543.03c	*pku80*	Ku domain protein Pku80		1.05	2.21	1.70
SPBC1271.09		glycerophosphodiester transporter	glycerophosphodiester transporter	0.99	10.11	2.27
SPAPJ691.02		yippee-like protein	yippee-like protein	1.19	16.23	18.77
SPAC977.17		MIP water channel (predicted)	MIP water channel	1.18	22.08	7.59
SPAC167.06c	*mug143*	sequence orphan	sequence orphan	0.93	14.88	8.53
SPCC330.02	*rhp7*	Rad7 homolog Rhp7		1.05	2.26	1.74
SPBP8B7.30c	*thi5*	transcription factor Thi5	transcription factor Thi5	0.85	3.00	3.37
SPCC10H11.02	*cwf23*	DNAJ domain protein Cwf23		0.99	1.69	2.03
SPCP20C8.03		pseudogene (predicted)		0.81	4.05	1.75
SPAC630.07c		sequence orphan	sequence orphan	0.97	4.04	2.52
SPAC1002.20		sequence orphan	sequence orphan	0.90	6.58	4.48
SPAC977.16c	*dak2*	dihydroxyacetone kinase Dak2	dihydroxyacetone kinase Dak2 (PMID 9804990)	1.06	12.35	8.29
SPCC1494.08c		conserved fungal protein	conserved fungal protein	0.84	1.76	1.74
SPBC1718.06	*msp1*	mitochondrial GTPase Msp1		0.99	2.15	1.88
SPBC337.06c	*cwf15*	complexed with Cdc5 protein Cwf15		0.89	1.65	1.62
SPCC576.06c		tyrosine-tRNA ligase	tyrosine-tRNA ligase	0.79	1.54	2.06
SPAC167.07c		ubiquitin-protein ligase E3 (predicted)	ubiquitin-protein ligase E3 (predicted)	1.02	2.42	2.32
SPCC4B3.01		thiosulfate sulfurtransferase		0.93	4.55	3.89
SPBC337.16	*cho1*	phosphatidyl-N-methylethanolamine N-methyltransferase (predicted)		0.96	1.64	1.76
SPAC4A8.04	*isp6*	vacuolar serine protease Isp6	vacuolar serine protease Isp6	1.13	5.54	4.51
SPAC4G9.14		mitochondrial Mvp17/PMP22 family protein 2	Mvp17/PMP22 family	1.06	1.56	1.88
SPAC18G6.09c		sequence orphan	sequence orphan	1.15	1.61	3.30
SPCC1450.13c		riboflavin synthase	riboflavin synthase (PMID 14690539)	1.01	7.78	2.99
SPAPB1E7.08c		membrane transporter	membrane transporter	0.84	4.93	3.77
SPAC12B10.01c		ubiquitin-protein ligase E3 (predicted)	ubiquitin-protein ligase E3 (predicted)	0.87	2.40	2.58
SPBC15D4.11c		sequence orphan		1.17	3.50	2.08
SPBP4H10.19c		calreticulin/calnexin homolog		1.09	1.64	1.53

SPBC215.13		sequence orphan		0.94	2.42	1.97
SPAC17C9.06	*sam50*	SAM complex subunit Sam50 (predicted)	SAM complex subunit Sam50 (predicted)	1.16	3.03	1.77
SPBC1604.01	*mug158*	sulfatase modifying factor 1 related		0.93	11.04	7.76
SPCC777.02		transcription factor (predicted)	transcription factor (predicted)	0.99	1.89	1.66
SPCC895.08c		conserved fungal protein	conserved fungal protein	0.88	4.60	5.06
SPBP8B7.07c	*set6*	histone lysine methyltransferase Set6 (predicted)		0.88	1.79	1.96
SPAC1F7.03	*pkd2*	TRP-like ion channel	TRP-like ion channel (PMID 15537393)	1.03	2.64	2.07
SPAC1F7.06		ThiJ domain protein	ThiJ domain protein	0.23	33.27	113.60
SPBP19A11.07c		human down-regulated in multiple cancers-1 homolog 2		0.88	2.53	2.99
SPCC23B6.01c		oxysterol binding protein (predicted)	oxysterol binding protein (predicted)	0.97	1.53	1.76
SPAPB2B4.07		ubiquitin family protein, human UBTD1 homolog	ubiquitin family protein, human UBTD1 homolog	1.08	1.71	1.91
SPBC19C7.04c		conserved fungal protein		1.27	2.22	1.89
SPBC947.10		ubiquitin-protein ligase E3 (predicted)		0.91	2.35	2.45
SPBC14C8.01c	*cut2*	securin		1.31	3.25	3.12
SPAC2E1P3.02c	*amt3*	ammonium transporter Amt3	ammonium transporter Amt3	0.91	1.52	2.18
SPBC106.03		DUF1776 family protein	DUF1776 family protein	1.16	2.20	1.54
SPAC29E6.05c		peptide methionine sulfoxide reductase (predicted)	peptide methionine sulfoxide reductase (predicted)	1.01	2.36	1.64
SPCC622.15c		sequence orphan	sequence orphan	0.85	1.81	2.60
SPBC29B5.01	*atf1*	transcription factor Atf1		0.91	2.54	2.59
SPBC11C11.12		pseudogene (predicted)		0.83	1.77	2.28
SPBP23A10.04	*apc2*	anaphase-promoting complex subunit Apc2		0.99	2.24	2.66
SPBP8B7.27	*mug30*	ubiquitin-protein ligase E3		1.13	1.85	1.51
SPCC24B10.02c		NAD/NADH kinase (predicted)	NAD/NADH kinase	0.82	2.95	2.49
SPAC17G6.08	*pep7*	prevacuole/endosomal FYVE tethering component Pep7 (predicted)	prevacuole/endosomal FYVE tethering component Pep7 (predicted)	0.92	1.74	2.08
SPAC23E2.03c	*ste7*	meiotic suppressor protein Ste7	meiotic suppressor protein Ste7	1.19	11.05	15.28
SPBC106.10	*pka1*	cAMP-dependent protein kinase catalytic subunit Pka1	cAMP-dependent protein kinase catalytic subunit Pka1 (PMID 8144551)	1.02	3.67	4.13
SPBC27B12.08		AP-1 accessory protein (predicted)		1.09	1.80	1.65
SPCC16A11.15c		sequence orphan	sequence orphan	0.81	11.14	3.76
SPAC4F8.08	*mug114*	sequence orphan	sequence orphan	0.27	18.64	30.11

SPAC22A12.08c		cardiolipin synthase/ hydrolase fusion protein (predicted)	cardiolipin synthase (predicted)	1.07	2.66	1.86
SPAC12G12.11c		DUF544 family protein	DUF544 family protein	1.08	2.69	2.41
SPCC790.02	*pep3*	ubiquitin-protein ligase E3 (predicted)	ubiquitin-protein ligase E3 (predicted)	0.89	1.76	1.60
SPCC24B10.03		sequence orphan	sequence orphan	1.18	3.90	2.15
SPAC1783.07c	*pap1*	transcription factor Caf3	transcription factor Caf3	0.91	1.69	1.89
SPAC212.04c		S. pombe specific DUF999 family protein 1	S. pombe specific DUF999 family protein 1	0.83	1.87	1.79
SPAC20G4.02c	*fus1*	formin Fus1	formin Fus1	0.83	6.37	8.79
SPAC22F8.05		alpha,alpha-trehalose-phosphate synthase (predicted)	alpha,alpha-trehalose-phosphate synthase (predicted)	0.78	6.21	7.48
SPBC19C2.09	*sre1*	sterol regulatory element binding protein Sre1		0.76	1.83	3.72
SPAC27D7.11c		But2 family protein	S. pombe specific But2 family protein	0.71	4.23	2.92
SPCC1322.14c	*vtc4*	vacuolar transporter chaperone (VTC) complex subunit (predicted)	vacuolar transporter chaperone (VTC) complex subunit (predicted)	1.02	1.50	1.65
SPBC609.03		WD repeat protein, human IQWD1 family		0.78	2.28	3.06
SPCC1919.14c	*bdp1*	transcription factor TFIIIB complex subunit Bdp1 (predicted)	transcription factor TFIIIB complex subunit Bdp1 (predicted)	0.89	1.76	1.96
SPAC17G6.02c	*tco1*	RTA1-like protein	RTA1-like protein	1.10	1.90	2.14
SPAC19G12.03	*cda1*	chitin deacetylase Cda1	chitin deacetylase Cda1	0.95	5.72	2.73
SPBC11C11.01		RNA-binding protein		0.76	1.92	2.04
SPAC869.09		conserved fungal protein	conserved fungal protein	0.84	5.63	5.26
SPBC1685.05		serine protease (predicted)	serine protease (predicted)	0.77	6.12	5.35
SPAC20G8.02		phospholipase (predicted)	phospholipase	0.96	2.63	2.14
SPAC139.03		transcription factor, zf-fungal binuclear cluster type (predicted)	transcription factor (predicted)	0.93	1.97	1.61
SPCC1322.05c		leukotriene A-4 hydrolase (predicted)	leukotriene A-4 hydrolase (predicted)	0.92	2.17	1.99
SPAC23A1.18c	*mrp51*	mitochondrial ribosomal protein subunit L51-b (predicted)	mitochondrial ribosomal protein subunit L51-b (predicted)	1.03	1.95	1.86
SPCC1223.10c	*eaf1*	RNA polymerase II transcription elongation factor SpEAF	RNA polymerase II transcription elongation factor SpEAF	0.78	3.07	2.81
SPCP31B10.06	*mug190*	C2 domain protein	C2 domain protein Tcb3 (predicted)	1.02	15.06	8.07
SPBP18G5.03	*toc1*	Tor Complex Tor2 interacting protein 1		0.93	1.98	1.99
SPBC1706.02c	*wtf2*	wtf element Wtf2, pseudo		0.98	2.48	1.93
SPCC1235.08c	*pdh1*	DUF1751 family protein	DUF1751 family protein	0.95	2.83	2.10
SPBC2D10.18	*abc1*	ABC1 kinase family protein		1.24	1.91	1.53

SPAP19A11.05c	*mrp7*		mitochondrial ribosomal protein subunit L27	mitochondrial ribosomal protein subunit L27	0.85	2.25	1.91
SPAC1006.04c	*mcp3*		sequence orphan	sequence orphan	1.14	1.77	1.88
SPBC21C3.03			ABC1 kinase family protein		1.12	3.06	1.94
SPBC26H8.05c			serine/threonine protein phosphatase (predicted)		0.97	1.87	1.78
SPBC428.10			sequence orphan	sequence orphan	0.79	5.28	3.25
SPAC1952.13	*ned1*		lipin Ned1	lipin	0.90	2.23	2.81
SPAC1F5.09c	*shk2*		PAK-related kinase Shk2	PAK-related kinase Shk2 (PMID 9660817) (PMID 9660818)	1.27	20.72	53.30
SPBC32F12.03c	*gpx1*		glutathione peroxidase Gpx1		0.86	2.75	2.92
SPAC17G8.12			sequence orphan	sequence orphan	1.10	3.17	2.37
SPAC644.11c			mitochondrial pyruvate dehydrogenase (lipoamide) kinase	pyruvate dehydrogenase (lipoamide) kinase	0.93	1.51	1.52
SPCC1753.02c	*git3*		G-protein coupled receptor Git3	G-protein coupled receptor Git3	0.86	1.70	2.04
SPAC21E11.03c	*pcr1*		transcription factor Pcr1	transcription factor Pcr1	0.67	1.65	2.00
SPCC191.04c			dubious	dubious	1.30	2.60	2.43
SPAC4G8.12c			alpha-1,2-mannosyltransferase (predicted)	alpha-1,2-mannosyltransferase (predicted)	1.04	3.62	3.30
SPBC106.09	*cut4*		anaphase-promoting complex subunit Apc1	anaphase-promoting complex subunit Apc1	0.96	1.60	1.62
SPAC1F3.10c		*41183*	mitochondrial intermediate peptidase Oct1 (predicted)	mitochondrial intermediate peptidase Oct1 (predicted)	0.97	3.26	2.38
SPBC887.18c			SAGA complex subunit (predicted)		0.89	1.66	1.72
SPBC2D10.05	*exg3*		glucan 1,3-beta-glucosidase Exg3		0.85	4.60	2.90
SPCC1919.10c	*myo52*		myosin type V	myosin type V	0.90	1.52	1.86
SPBC1198.13c	*tfg2*		transcription factor TFIIF complex beta subunit Tfg2 (predicted)		0.91	2.69	2.76
SPAC23H3.04			conserved fungal protein	conserved fungal protein	0.74	4.78	5.82
SPBC19C7.03	*cyr1*		adenylate cyclase		0.83	2.32	2.89
SPAC8C9.03	*cgs1*		cAMP-dependent protein kinase regulatory subunit Cgs1	cAMP-dependent protein kinase regulatory subunit Cgs1	1.41	4.15	3.31
SPBC3E7.08c	*rad13*		DNA repair nuclease Rad13		0.87	1.52	1.67
SPAC869.08	*pcm2*		protein-L-isoaspartate O-methyltransferase (predicted)	protein-L-isoaspartate O-methyltransferase (predicted)	0.59	2.55	4.62
SPBPJ4664.03	*mfm3*		M-factor precursor Mfm3		1.48	8.41	2.05
SPCC162.03			short chain dehydrogenase (predicted)	short chain dehydrogenase (predicted)	0.95	6.85	7.05
SPAC20H4.02			conserved fungal protein	conserved fungal protein	0.94	2.30	1.87

SPAC3A11.05c	*kms1*	meiotic spindle pole body protein Kms1	meiotic spindle pole body protein Kms1	0.91	2.50	3.08
SPAC23C4.05c		LEA domain protein	LEA domain protein	0.91	1.95	2.13
SPAC186.04c		pseudogene, similar to N-terminal of transmembrane channel	pseudogene, similar to N-terminal of transmembrane channel	0.24	14.00	39.34
SPBC713.08	*tom13*	mitochondrial TOM complex subunit Tom13		1.02	1.70	1.80
SPBC1D7.05	*byr2*	MAP kinase kinase kinase Byr2		0.93	2.50	2.20
SPCC132.04c		NAD-dependent glutamate dehydrogenase (predicted)	NAD-dependent glutamate dehydrogenase (predicted)	1.36	5.90	2.56
SPBC1778.10c	*ppk21*	serine/threonine protein kinase Ppk21		0.74	1.66	1.51
SPAC630.05	*gyp7*	GTPase activating protein Gyp7 (predicted)	GTPase activating protein Gyp7 (predicted)	1.24	3.14	2.92
SPBC660.07	*ntp1*	alpha,alpha-trehalase Ntp1	alpha,alpha-trehalase Ntp1	0.95	2.25	1.98
SPAC7D4.09c		steroid dehydrogenase (predicted)	steroid dehydrogenase (predicted)	1.08	1.58	1.68
SPAC688.04c	*gst3*	glutathione S-transferase Gst3	glutathione S-transferase (PMID 12151111)	1.10	4.82	3.04
SPCC1672.08c	*tfa2*	transcription factor TFIIE beta subunit Tfa2	transcription factor TFIIE beta subunit Tfa2	0.85	1.59	1.65
SPBP18G5.02		CDP-diacylglycerol-glycerol-3-phosphate3-phosphatidyltransferase		1.20	3.23	2.60
SPAC1039.06		alanine racemase (predicted)	alanine racemase (predicted)	1.09	2.34	1.65
SPCC757.07c	*ctt1*	catalase	catalase	0.89	2.44	2.30
SPAC31G5.15		phosphatidylserine decarboxylase (predicted)	phosphatidylserine decarboxylase (predicted)	0.80	1.85	1.68
SPCC1919.12c		aminopeptidase (predicted)	aminopeptidase (predicted)	1.01	1.82	1.83
SPBC1734.08	*hse1*	STAM like protein Hse1		0.96	2.63	1.95
SPBC31F10.02		thioesterase superfamily protein		1.10	2.23	1.98
SPAC607.09c	*btn1*	battenin CLN3 family protein	battenin CLN3 family protein	0.92	2.01	1.93
SPCC16A11.01		plasma membrane protein involved in inositol lipid-mediated signaling	conserved fungal protein	0.88	2.16	2.21
SPBC1773.09c	*mug184*	meiotically upregulated gene Mug184	meiotically upregulated gene Mug184	1.02	1.96	2.01
SPAC1952.04c			dubious	1.13	2.17	2.39
SPAC3A12.08		conserved fungal protein	conserved fungal protein	1.11	4.05	2.28
SPCC1235.06	*sif1*	Sad1 interacting factor 1	Sad1 interacting factor 1	0.86	2.25	2.15
SPAC17A2.07c		sequence orphan	sequence orphan	0.78	2.39	2.40
SPCC188.12	*spn6*	septin Spn6	septin Spn6	1.18	1.61	2.87
SPAC12D12.09				0.73	2.39	2.92

SPBC1198.01		glutathione-dependent formaldehyde dehydrogenase (predicted)	glutathione-dependent formaldehyde dehydrogenase (predicted)	1.15	2.81	2.37
SPCC191.11	*inv1*	beta-fructofuranosidase	beta-fructofuranosidase	0.48	39.29	61.31
SPAC105.01c		potassium ion/proton antiporter	potassium ion/proton antiporter	0.91	2.08	2.00
SPAC4F10.07c	*atg13*	autophagy associated protein Atg13 (predicted)	autophagy associated protein Atg13 (predicted)	0.93	3.80	3.13
SPCC11E10.02c	*gpi8*	pig-K	pig-K	0.85	1.70	1.87
SPBC1105.13c		sequence orphan		1.04	1.62	1.53
SPAC1782.01		proteasome component	proteasome component	0.95	1.51	1.52
SPAC56E4.06c	*ggt2*	gamma-glutamyltranspeptidase Ggt2	gamma-glutamyltranspeptidase Ggt2	1.07	2.14	2.34
SPBC16D10.04c	*dna2*	DNA replication endonuclease-helicase Dna2		1.22	1.53	1.92
SPAC11D3.01c		conserved fungal protein	conserved fungal protein	0.96	4.01	1.71
SPAC23H3.13c	*gpa2*	heterotrimeric G protein alpha-2 subunit Gpa2	heterotrimeric G protein alpha-2 subunit Gpa2 (PMID 1340462)	1.30	2.14	1.97
SPCC830.02	*wtf24*	wtf element Wtf24		0.79	3.43	4.70
SPAC24B11.05		pyrimidine 5'-nucleotidase (predicted)	pyrimidine 5'-nucleotidase (predicted)	0.93	6.82	2.95
SPCC306.08c		malate dehydrogenase	malate dehydrogenase	0.98	3.31	2.74
SPBC1718.01	*pop1*	F-box/WD repeat protein Pop1		1.03	3.41	3.42
SPBC651.05c	*dot2*	EAP30 family protein Dot2		1.02	1.65	2.14
SPCC736.05	*wtf7*	wtf element Wtf7	wtf element Wtf7	1.00	1.74	2.03
SPCC1450.01c		pseudogene		1.18	2.53	1.65
SPCC18.09c		human aprataxin homolog	conserved eukaryotic protein	1.07	5.62	2.72
SPAC15A10.06		CPA1 sodium ion/proton antiporter	CPA1 sodium ion/proton antiporter	0.92	1.71	1.67
SPBP8B7.24c	*atg8*	autophagy associated protein Atg8 (predicted)		1.06	3.42	2.60
SPBC83.05		mitochondrial RNA-binding protein (predicted)		0.94	3.70	2.19
SPAP11E10.02c	*mam3*	cell agglutination protein Mam3		0.94	2.21	2.31
SPBC29A10.04	*psm1*	mitotic cohesin complex subunit Psm1		0.98	1.51	1.74
SPAC27D7.08c		DUF890 family protein	DUF890 family protein	0.82	2.11	2.14
SPAC6F6.12		autophagy associated protein Atg24 (predicted)	autophagy associated protein Atg24	1.02	2.89	2.08
SPBC6B1.09c	*nbs1*	Mre11 complex subunit Nbs1		0.99	3.29	2.99
SPAC1296.03c	*sxa2*	serine carboxypeptidase Sxa2	serine carboxypeptidase Sxa2	0.98	1.59	2.37
SPAC31G5.18c		ubiquitin family, human C1ORF55 related	ubiquitin family, human C1ORF55 related	0.96	2.48	1.86
SPAC6F6.16c		sequence orphan	sequence orphan	0.79	1.70	2.03

SPAC1F7.09c		allantoicase (predicted)	allantoicase (predicted)	1.28	12.13	5.39
SPBC215.10		haloacid dehalogenase-like hydrolase		1.09	2.56	2.87
SPBC13E7.10c	*brf1*	transcription factor TFIIIB complex subunit Brf1		0.98	1.75	2.09
SPBC1773.13		aromatic aminotransferase (predicted)	aromatic aminotransferase (predicted)	1.15	1.95	1.64
SPAC17G6.03		phosphoprotein phosphatase (predicted)	phosphoprotein phosphatase	0.76	13.26	2.70
SPCC1223.03c	*gut2*	glycerol-3-phosphate dehydrogenase Gut2	glycerol-3-phosphate dehydrogenase Gut2	0.54	3.47	4.90
SPCC970.09	*sec8*	exocyst complex subunit Sec8	exocyst complex subunit Sec8	0.96	1.76	2.11
SPAC23C4.13	*bet1*	SNARE Bet1	SNARE Bet1	0.95	1.57	1.92
SPAC688.03c		human AMMECR1 homolog	human AMMECR1 homolog	0.74	12.46	10.25
SPAC1751.01c	*gti1*	gluconate transporter inducer Gti1	gluconate transporter inducer Gti1	0.91	9.66	12.06
SPMIT.01	*cox1*	cytochrome c oxidase 1	cytochrome c oxidase 1; similar to S. cerevisiae Q0045	0.81	2.05	3.78
SPAC959.09c	*apc5*	anaphase-promoting complex subunit Apc5	anaphase-promoting complex subunit Apc5 (PMID 12477395)	0.93	1.65	1.84
SPBC1539.02		sequence orphan		1.07	2.59	1.66
SPBC1773.02c		thioredoxin peroxidase	thioredoxin peroxidase	1.00	6.80	4.02
SPCC306.06c		ER membrane protein, BIG1 family (predicted)	ER membrane protein, BIG1 family (predicted)	1.02	1.69	1.80
SPBC31E1.01c	*atg2*	autophagy associated protein Mug36	autophagy associated protein Mug36	0.85	2.28	2.43
SPCC1620.14c	*snf22*	ATP-dependent DNA helicase Snf22	ATP-dependent DNA helicase Snf22	0.85	1.58	2.02
