# Supplementary material for: The Fission Yeast GATA Factor, Gaf1, Modulates Sexual Development via Direct Down-Regulation of ste11+ Expression in Response to Nitrogen Starvation
Source: PLoS One. 2012 Aug 10;7(8):e42409. doi: 10.1371/journal.pone.0042409 (PMC3416868; doi:10.1371/journal.pone.0042409)
Supplement: Table S8 — List of the genes in Subgroup E. (PDF) [file pone.0042409.s008.pdf]

# Table S8. List of the genes in Subgroup E

*** 369 genes**

| Systematic | Gene name | Description (GeneDB) | Description (FunCat2) | Expression Ratio (gaf1Δ, +N) / (WT, +N) | Expression Ratio (WT, -N) / (WT, +N) | Expression Ratio (gaf1Δ, -N) / (gaf1Δ, +N) |
|---|---|---|---|---|---|---|
| SPMIT.09 | *atp8* | F0-ATPase subunit 8 | | 0.01 | 0.98 | 101.37 |
| SPAC1093.06c | *dhc1* | dynein heavy chain | | 0.30 | 1.47 | 15.66 |
| SPCC1020.01c | *pma2* | P-type proton ATPase Pma2 | | 0.43 | 1.26 | 10.26 |
| SPBC29A10.14 | *rec8* | meiotic cohesin complex subunit Rec8 | | 0.33 | 1.23 | 6.74 |
| SPCC1235.13 | *ght6* | hexose transporter Ght6 | hexose transporter Ght6 (PMID 10735857) | 0.68 | 1.32 | 4.81 |
| SPAC212.08c | | GPI anchored protein (predicted) | GPI anchored protein (predicted) | 1.04 | 1.32 | 4.31 |
| SPBC1718.02 | *hop1* | linear element associated protein Hop1 | | 0.90 | 1.17 | 4.26 |
| SPBC1348.13 | | pseudogene | pseudogene | 0.13 | 0.06 | 4.17 |
| SPBC354.08c | | DUF221 family protein | DUF221 family protein | 0.36 | 0.92 | 4.07 |
| SPAC5D6.10c | *mug116* | sequence orphan | sequence orphan | 0.26 | 0.67 | 3.83 |
| SPAC6C3.07 | *mug68* | sequence orphan | sequence orphan | 0.33 | 1.15 | 3.39 |
| SPAPB18E9.04c | | sequence orphan | sequence orphan | 1.05 | 0.80 | 3.36 |
| SPAC23D3.05c | | alcohol dehydrogenase pseudogene | alcohol dehydrogenase pseudogene | 0.51 | 0.44 | 3.30 |
| SPBC14C8.05c | *meu17* | glucan-alpha-1,4-glucosidase | | 1.39 | 1.37 | 3.18 |
| SPBP8B7.04 | *mug45* | sequence orphan | | 0.74 | 1.19 | 3.14 |
| SPCC1450.16c | | triacylglycerol lipase | triacylglycerol lipase | 0.67 | 1.13 | 3.12 |
| SPBC1348.09 | | short chain dehydrogenase (predicted) | | 0.94 | 1.43 | 3.08 |
| SPAC1786.04 | | sequence orphan | sequence orphan | 0.36 | 0.87 | 3.06 |
| SPAC13C5.03 | *tht1* | nuclear membrane protein involved in karyogamy | nuclear membrane protein involved in karyogamy (PMID 9442101) | 1.04 | 0.62 | 3.01 |
| SPAC1786.01c | | triacylglycerol lipase | triacylglycerol lipase | 0.66 | 0.89 | 2.96 |
| SPBC1685.13 | | non classical export pathway protein (predicted) | non classical export pathway protein (predicted) | 0.71 | 1.43 | 2.93 |
| SPBC32H8.11 | *mei4* | meiotic forkhead transcription factor Mei4 | | 0.83 | 1.24 | 2.88 |
| SPBPB8B6.02c | | urea transporter (predicted) | urea transporter (predicted) | 0.34 | 0.68 | 2.88 |
| SPAC1250.02 | *mug95* | sequence orphan | sequence orphan | 0.73 | 0.21 | 2.83 |
| SPCC1902.01 | *gaf1* | transcription factor Gaf1 | | 0.01 | 1.45 | 1.71 |
| SPBC6B1.03c | | Pal1 family protein | | 0.77 | 1.03 | 2.77 |
| SPAC4F8.13c | *rng2* | IQGAP | IQGAP | 0.76 | 1.44 | 2.74 |
| SPAC13A11.06 | | pyruvate decarboxylase (predicted) | | 1.27 | 1.14 | 2.69 |

| | | | | | | |
|---|---|---|---|---|---|---|
| SPBP4G3.02 | *pho1* | acid phosphatase Pho1 | | 1.41 | 1.48 | 2.59 |
| SPCC4E9.01c | *rec11* | meiotic cohesin complex subunit Rec11 | | 0.56 | 1.04 | 2.58 |
| SPBC32H8.13c | *mok12* | alpha-1,3-glucan synthase Mok12 | | 0.76 | 1.24 | 2.56 |
| SPAC22F3.04 | *mug62* | AMP binding enzyme (predicted) | AMP binding enzyme (predicted) | 0.59 | 0.10 | 2.55 |
| SPCC320.07c | *mde7* | RNA-binding protein Mde7 | RNA-binding protein Mde7 | 0.34 | 0.95 | 2.45 |
| SPBC16A3.13 | *meu7* | alpha-amylase homolog Aah4 | | 1.05 | 0.81 | 2.42 |
| SPAPB2B4.03 | *cig2* | cyclin Cig2 | cyclin Cig2 | 0.80 | 1.09 | 2.39 |
| SPAC4A8.10 | | lipase (predicted) | lipase (predicted) | 0.83 | 1.12 | 2.38 |
| SPCC736.09c | | TRAX | TRAX (PMID 16043634) | 0.51 | 0.95 | 2.37 |
| SPCC338.17c | *rad21* | kleisin | kleisin | 0.83 | 1.07 | 2.36 |
| SPAC630.15 | *mug177* | sequence orphan | sequence orphan | 0.53 | 0.68 | 2.36 |
| SPAC1687.08 | | sequence orphan | sequence orphan | 0.93 | 1.45 | 2.32 |
| SPAC13G6.12c | *chs1* | chitin synthase I | | 0.84 | 1.33 | 2.28 |
| SPCC290.04 | *ams2* | cell cycle regulated GATA-type transcription factor Ams2 | | 1.11 | 1.21 | 2.28 |
| SPCC1259.14c | *meu27* | S. pombe specific UPF0300 family protein 5 | S. pombe specific UPF0300 family protein 5 | 0.68 | 0.84 | 2.25 |
| SPAC17A5.18c | *rec25* | meiotic recombination protein Rec25 | meiotic recombination protein Rec25 | 1.03 | 1.32 | 2.24 |
| SPAC31G5.10 | *eta2* | Myb family protein Eta2 | Myb family transcriptional regulator Eta2 | 1.04 | 1.41 | 2.22 |
| SPBC557.05 | | arrestin | | 0.86 | 0.60 | 2.21 |
| SPAC22A12.02c | *mug103* | sequence orphan | sequence orphan | 0.36 | 1.13 | 2.20 |
| SPAC25B8.03 | | phosphatidylserine decarboxylase | phosphatidylserine decarboxylase | 1.24 | 1.21 | 2.19 |
| SPCC74.06 | *mak3* | histidine kinase Mak3 | histidine kinase Mak3 (PMID 11758939) | 0.67 | 1.43 | 2.17 |
| SPAC3C7.06c | *pit1* | serine/threonine protein kinase Pit1 | serine/threonine protein kinase Pit1 | 0.93 | 1.44 | 2.17 |
| SPBC354.09c | | Tre1 family protein (predicted) | Tre1 family protein (predicted) | 0.88 | 1.49 | 2.12 |
| SPAC17A2.10c | | sequence orphan | sequence orphan | 0.74 | 1.29 | 2.11 |
| SPBC336.07 | *sfc3* | transcription factor TFIIIC complex subunit Sfc3 | | 0.79 | 1.48 | 2.11 |
| SPBC354.05c | *sre2* | membrane-tethered transcription factor (predicted) | membrane-tethered transcription factor (predicted) (PMID 11790253) | 0.99 | 1.28 | 2.11 |
| SPBC1703.04 | *mlh1* | MutL family protein Mlh1 (predicted) | | 0.77 | 1.14 | 2.10 |
| SPAC27E2.09 | *mak2* | histidine kinase Mak2 | histidine kinase Mak2 (PMID 11758939) | 0.82 | 1.44 | 2.09 |
| SPBC119.04 | *mei3* | meiosis inducing protein Mei3 | | 1.12 | 1.33 | 2.08 |
| SPBP4H10.11c | *lcf2* | long-chain-fatty-acid-CoA ligase | | 0.75 | 1.02 | 2.08 |

| | | | | | | |
|---|---|---|---|---|---|---|
| SPBC2G2.06c | *apl1* | AP-2 adaptor complex subunit Apl1 (predicted) | | 0.78 | 1.46 | 2.06 |
| SPAC18G6.10 | | chromosome segregation protein Heh1 (predicted) | chromosome segregation protein (predicted) | 0.80 | 1.29 | 2.06 |
| SPAC7D4.04 | *taf1* | Taz1 interacting factor 1 | Taz1 interacting factor 1 | 0.86 | 1.48 | 2.06 |
| SPBC1E8.02 | | ubiquitin family protein, unknown | | 0.66 | 1.11 | 2.05 |
| SPAPB1E7.05 | *gde1* | glycerophosphoryl diester phosphodiesterase Gde1 | glycerophosphoryl diester phosphodiesterase | 0.84 | 1.33 | 2.05 |
| SPAC56F8.13 | | dubious | dubious | 0.49 | 0.44 | 2.05 |
| SPBC21.05c | *ral2* | Ras guanyl-nucleotide exchange factor Ral2 (predicted) | | 0.74 | 1.41 | 2.05 |
| SPBC12D12.02c | *cdm1* | DNA polymerase delta subunit Cdm1 | | 1.05 | 0.99 | 2.04 |
| SPAC57A10.02 | *cdr2* | GIN4 family protein kinase Cdr2 | GIN4 family protein kinase Cdr2 | 0.90 | 1.26 | 2.04 |
| SPBC2A9.07c | | zf-PARP type zinc finger protein | | 0.89 | 1.03 | 2.03 |
| SPBC3B8.07c | *dsd1* | dihydroceramide delta-4 desaturase | | 1.06 | 1.49 | 2.03 |
| SPAC15A10.13 | *ppk3* | serine/threonine protein kinase Ppk3 | serine/threonine protein kinase Ppk3 | 0.97 | 1.35 | 2.03 |
| SPBC29A3.08 | *pof4* | elongin-A, F-box protein Pof4 | | 0.86 | 1.19 | 2.00 |
| SPAC1F3.08c | | dubious | dubious | 0.57 | 0.20 | 2.00 |
| SPAC2F3.10 | | GARP complex subunit Vps54 (predicted) | GARP complex subunit Vps54 (predicted) | 0.82 | 1.43 | 1.99 |
| SPAC3H8.06 | *aur1* | inositol phosphorylceramide synthase (predicted) | inositol phosphorylceramide synthase (predicted) | 0.94 | 1.08 | 1.99 |
| SPAC24B11.12c | | P-type ATPase | P-type ATPase (PMID 12707717) | 0.81 | 1.47 | 1.99 |
| SPCC4F11.05 | | dubious | dubious | 0.98 | 0.69 | 1.98 |
| SPAC22E12.11c | *set3* | histone lysine methyltransferase Set3 | histone lysine methyltransferase Set3 | 0.92 | 1.30 | 1.98 |
| SPAC13G7.03 | | up-frameshift suppressor3 family | up-frameshift suppressor3 family | 0.55 | 0.99 | 1.97 |
| SPBC530.04 | *mod5* | Tea1 anchoring protein Mod5 | | 0.96 | 1.16 | 1.97 |
| SPAC18B11.09c | | N-acetyltransferase (predicted) | N-acetyltransferase (predicted) | 0.69 | 1.39 | 1.97 |
| SPAPB1A10.13 | | sequence orphan | sequence orphan | 0.89 | 1.18 | 1.96 |
| SPCC16C4.01 | *sif2* | Sad1 interacting factor 2 | | 0.57 | 0.98 | 1.96 |
| SPAC56F8.16 | *esc1* | transcription factor Esc1 (predicted) | transcription factor Esc1 (predicted) | 1.01 | 1.39 | 1.96 |
| SPAC3A11.11c | | pyridoxal reductase (predicted) | pyridoxal reductase (predicted) | 0.88 | 1.41 | 1.96 |
| SPAC8F11.10c | *pvg1* | pyruvyltransferase | | 1.27 | 1.39 | 1.96 |
| SPAC30D11.11 | | Haemolysin-III family protein | Haemolysin-III family protein | 0.74 | 0.53 | 1.96 |
| SPCC11E10.03 | *mug1* | dynactin complex subunit (predicted) | dynactin complex subunit (predicted) | 0.61 | 0.84 | 1.95 |
| SPAPB1A10.08 | | sequence orphan | sequence orphan | 1.03 | 1.36 | 1.95 |
| SPAC31A2.05c | *mis4* | cohesin loading factor Mis4 | cohesin loading factor Mis4 | 0.81 | 1.44 | 1.95 |

| | | | | | | |
|---|---|---|---|---|---|---|
| SPBC1718.04 | | glycerol-3-phosphate O-acyltransferase (predicted) | | 0.83 | 1.38 | 1.94 |
| SPAC1687.14c | | EF hand family protein, unknown role | EF hand family protein, unknown role | 0.96 | 1.43 | 1.94 |
| SPAC1565.01 | | conserved fungal protein | conserved fungal protein | 0.97 | 0.89 | 1.93 |
| SPAC13G7.02c | *ssa1* | heat shock protein Ssa1 (predicted) | heat shock protein Ssa1 | 0.82 | 1.43 | 1.93 |
| SPBC27B12.11c | | transcription factor (predicted) | | 1.21 | 1.25 | 1.93 |
| SPBC3B8.02 | *php5* | CCAAT-binding factor complex subunit Php5 | | 0.94 | 1.42 | 1.92 |
| SPAC1093.03 | | inositol polyphosphate phosphatase (predicted) | polyphosphoinositide phosphatase (predicted) | 0.87 | 1.46 | 1.92 |
| SPBC25H2.13c | *cdc20* | DNA polymerase epsilon catalytic subunit a Pol2 | | 0.97 | 1.17 | 1.92 |
| SPBC409.09c | *mis13* | kinetochore protein Mis13 | kinetochore protein Mis13 | 0.58 | 1.27 | 1.91 |
| SPMIT.11 | *cox2* | cytochrome c oxidase 2 | cytochrome c oxidase 2; similar to S. cerevisiae Q0250 | 0.95 | 1.47 | 1.91 |
| SPBC3H7.05c | | sequence orphan | | 1.13 | 1.20 | 1.91 |
| SPAC227.11c | | sensor for misfolded ER glycoproteins Yos9 (predicted) | sensor for misfolded ER glycoproteins Yos9 (predicted) | 0.93 | 1.48 | 1.90 |
| SPAC9E9.12c | *ybt1* | ABC transporter Ybt1 | ABC transporter Ybt1 | 0.75 | 1.27 | 1.90 |
| SPAC25G10.09c | | actin cortical patch component, with EF hand and WH2 motif (predicted) | | 1.00 | 1.44 | 1.90 |
| SPBC1105.15c | *htd2* | 3-hydroxyacyl-ACP dehydratase Htd2 (predicted) | | 0.84 | 1.34 | 1.89 |
| SPAC10F6.09c | *psm3* | mitotic cohesin complex subunit Psm3 | mitotic cohesin complex subunit Psm3 | 1.24 | 1.16 | 1.89 |
| SPAC1006.03c | | human CCDC131 homolog | human CCDC131 homolog | 0.84 | 1.17 | 1.88 |
| SPBC2D10.13 | *est1* | telomerase regulator Est1 | | 0.79 | 0.90 | 1.87 |
| SPAC19G12.01c | *cut20* | anaphase-promoting complex subunit Apc4 | anaphase-promoting complex subunit Apc4 | 0.85 | 1.15 | 1.87 |
| SPAC17A5.04c | *mde10* | spore wall assembly peptidase Mde10 | spore wall assembly peptidase Mde10 | 0.89 | 0.83 | 1.87 |
| SPBC12C2.09c | | Haemolysin-III family protein | | 0.89 | 1.13 | 1.87 |
| SPBC106.20 | *exo70* | exocyst complex subunit Exo70 (predicted) | | 0.82 | 1.44 | 1.87 |
| SPAC11E3.08c | *nse6* | Smc5-6 complex non-SMC subunit Nse6 | Smc5-6 complex non-SMC subunit Nse6 | 0.77 | 1.32 | 1.87 |
| SPAC4F10.08 | *mug126* | sequence orphan | sequence orphan | 0.65 | 1.29 | 1.86 |
| SPBC25H2.08c | *mrs2* | magnesium ion transporter Mrs2 | magnesium ion transporter Mrs2 | 0.78 | 1.27 | 1.86 |
| SPCC1742.01 | | sequence orphan | | 0.44 | 0.51 | 1.86 |
| SPCC550.08 | | N-acetyltransferase (predicted) | N-acetyltransferase (predicted) | 0.87 | 1.31 | 1.86 |
| SPBC216.07c | *tor2* | phosphatidylinositol kinase Tor2 | | 0.88 | 1.42 | 1.86 |

| | | | | | | |
|---|---|---|---|---|---|---|
| SPBC24C6.08c | | vesicle coat protein | | 0.78 | 1.12 | 1.85 |
| SPBC8E4.02c | | sequence orphan | | 0.72 | 1.35 | 1.85 |
| SPAC11D3.18c | | nicotinic acid plasma membrane transporter (predicted) | nicotinic acid plasma membrane transporter (predicted) | 0.61 | 1.42 | 1.85 |
| SPBC691.03c | *apl3* | AP-2 adaptor complex subunit Alp3 (predicted) | | 0.84 | 0.91 | 1.84 |
| SPAC1556.06b | | | sequence orphan | 1.29 | 0.91 | 1.84 |
| SPAC12G12.09 | | sequence orphan | sequence orphan | 0.82 | 1.40 | 1.84 |
| SPAC19G12.08 | | fatty acid hydroxylase (predicted) | fatty acid hydroxylase (predicted) | 0.87 | 0.68 | 1.84 |
| SPBC29A3.14c | *trt1* | telomerase reverse transcriptase 1 protein Trt1 | | 0.79 | 1.08 | 1.84 |
| SPCC645.06c | *rgf3* | RhoGEF Rgf3 | RhoGEF Rgf3 | 0.98 | 1.12 | 1.84 |
| SPBC30D10.10c | *tor1* | phosphatidylinositol kinase Tor1 | | 0.85 | 1.41 | 1.83 |
| SPCC663.06c | | short chain dehydrogenase (predicted) | short chain dehydrogenase (predicted) | 0.55 | 0.75 | 1.83 |
| SPCC970.06 | | cargo receptor for soluble proteins (predicted) | cargo receptor for soluble proteins (predicted) | 0.94 | 1.49 | 1.83 |
| SPAC12B10.08c | | mitochondrial tRNA(Ile)-lysidine synthetase family (predicted) | tRNA(Ile)-lysidine synthetase family | 0.98 | 1.33 | 1.83 |
| SPCC970.04c | *mob2* | protein kinase activator Mob2 | protein kinase activator Mob2 | 0.91 | 1.15 | 1.82 |
| SPCPJ732.01 | *vps5* | retromer complex subunit Vps5 | retromer complex subunit Vps5 | 0.83 | 1.48 | 1.82 |
| SPAC589.02c | *med13* | mediator complex subunit Srb9 | mediator complex subunit Srb9 | 0.93 | 1.21 | 1.81 |
| SPCC550.03c | | Ski complex RNA helicase Ski2 (predicted) | RNA helicase involved in mRNA catabolism | 0.83 | 1.44 | 1.81 |
| SPAPYUG7.02c | *sin1* | stress activated MAP kinase interacting protein Sin1 | stress activated MAP kinase interacting protein Sin1 | 0.88 | 1.41 | 1.80 |
| SPBC3D6.10 | *apn2* | AP-endonuclease Apn2 | | 0.89 | 1.43 | 1.80 |
| SPCC23B6.03c | *tel1* | ATM checkpoint kinase | ATM checkpoint kinase | 1.06 | 1.50 | 1.80 |
| SPCC320.14 | | threo-3-hydroxyaspartate ammonia-lyase (predicted) | threo-3-hydroxyaspartate ammonia-lyase (predicted) | 0.89 | 1.17 | 1.80 |
| SPAC1565.06c | *spg1* | GTPase Spg1 | GTPase Spg1 | 0.96 | 1.30 | 1.80 |
| SPAC1687.02 | | CAAX prenyl protease (predicted) | CAAX prenyl protease (predicted) | 1.01 | 1.49 | 1.80 |
| SPAC29B12.08 | | sequence orphan | sequence orphan | 0.80 | 0.45 | 1.80 |
| SPCC188.10c | | pseudo | | 1.22 | 0.52 | 1.80 |
| SPAC17H9.20 | *psc3* | mitotic cohesin complex, non-SMC subunit Psc3 | | 1.10 | 1.11 | 1.80 |
| SPAC1250.01 | *snf21* | ATP-dependent DNA helicase Snf21 | ATP-dependent DNA helicase Snf21 | 0.91 | 1.22 | 1.79 |
| SPAC23C11.16 | *plo1* | Polo kinase Plo1 | Polo kinase Plo1 | 0.94 | 1.29 | 1.79 |
| SPBC16G5.09 | | serine carboxypeptidase (predicted) | | 0.92 | 1.28 | 1.78 |

| | | | | | | |
|---|---|---|---|---|---|---|
| SPAC26H5.11 | | spore wall assembly protein (predicted) | spore wall assembly protein (predicted) | 0.67 | 1.08 | 1.78 |
| SPAC11E3.10 | | VanZ-like family protein | VanZ-like family protein | 0.87 | 1.23 | 1.78 |
| SPBC16A3.10 | | membrane bound O-acyltransferase, MBOAT (predicted) | | 0.86 | 1.40 | 1.78 |
| SPAC23G3.09 | *taf4* | transcription factor TFIID complex subunit Taf4 (predicted) | transcription factor TFIID complex subunit Taf4 | 0.75 | 1.36 | 1.77 |
| SPAC57A10.04 | *mug10* | sequence orphan | sequence orphan | 0.87 | 0.74 | 1.77 |
| SPBC1105.08 | | EMP70 family | | 0.82 | 1.17 | 1.76 |
| SPBC4C3.04c | | guanyl-nucleotide exchange factor (predicted) | | 0.99 | 1.28 | 1.76 |
| SPBC651.03c | *gyp10* | GTPase activating protein Gyp10 | | 1.04 | 1.48 | 1.76 |
| SPCC16C4.09 | *sts5* | RNB-like protein | RNB-like protein | 0.94 | 1.32 | 1.76 |
| SPBC582.04c | | sequence orphan | sequence orphan | 0.90 | 1.35 | 1.75 |
| SPAC144.05 | | ATP-dependent DNA helicase (predicted) | ATP-dependent DNA helicase | 0.85 | 1.15 | 1.75 |
| SPAC4A8.09c | *cwf21* | complexed with Cdc5 protein Cwf21 | complexed with Cdc5 protein Cwf21 (PMID 11884590) | 0.83 | 0.88 | 1.75 |
| SPAC12B10.03 | | WD repeat protein, human WDR20 family | WD repeat protein, human WDR20 family | 0.91 | 1.24 | 1.75 |
| SPBC1709.12 | *rid1* | GTPase binding protein Rid1 | | 1.02 | 0.69 | 1.75 |
| SPCC4B3.15 | *mid1* | medial ring protein Mid1 | medial ring protein Mid1 | 0.88 | 1.42 | 1.75 |
| SPBC8E4.01c | | inorganic phosphate transporter (predicted) | | 1.22 | 0.96 | 1.75 |
| SPBC776.10c | *cog6* | Golgi transport complex peripheral subunit Cog6 (predicted) | | 0.84 | 1.37 | 1.74 |
| SPAC3C7.09 | *set8* | lysine methyltransferase Set8 (predicted) | lysine methyltransferase Set8 (predicted) | 0.78 | 1.30 | 1.74 |
| SPCC777.11 | | sequence orphan | sequence orphan | 0.78 | 0.92 | 1.74 |
| SPCC417.02 | *dad5* | DASH complex subunit Dad5 | DASH complex subunit Dad5 | 0.49 | 0.11 | 1.74 |
| SPCC126.07c | | human CTD-binding SR-like protein rA9 homolog | ubiquitin-protein ligase E3 (predicted) | 0.73 | 1.21 | 1.73 |
| SPAC22E12.14c | *sck2* | serine/threonine protein kinase Sck2 | serine/threonine protein kinase Sck2 | 0.76 | 0.89 | 1.73 |
| SPBC27B12.12c | | CorA family magnesium ion transporter (predicted) | | 0.86 | 1.42 | 1.73 |
| SPCC548.05c | | ubiquitin-protein ligase E3 (predicted) | ubiquitin-protein ligase E3 (predicted) | 0.78 | 1.30 | 1.73 |
| SPCC132.05c | | trichothecene 3-O-acetyltransferase pseudogene | | 1.31 | 1.16 | 1.73 |

| | | | | | | |
|---|---|---|---|---|---|---|
| SPBC660.13c | *ssb1* | DNA replication factor A subunit Ssb1 | DNA replication factor A subunit Ssb1 (PMID 9111307) (PMID 8702843) | 0.96 | 1.13 | 1.73 |
| SPCC18.01c | *adg3* | beta-glucosidase Adg3 (predicted) | beta-glucosidase Adg3 (predicted) | 1.23 | 0.89 | 1.72 |
| SPAC17D4.03c | *cis4* | cation diffusion family zinc membrane transporter Cis4 | membrane transporter (predicted) | 0.88 | 1.36 | 1.72 |
| SPAC2G11.14 | *taf111* | transcription factor TFIID complex subunit Taf111 | transcription factor TFIID complex subunit Taf111 | 0.79 | 1.35 | 1.72 |
| SPBC21H7.03c | | acid phosphatase (predicted) | | 0.82 | 1.28 | 1.72 |
| SPAC23C4.11 | *atp18* | F0-ATPase subunit J (predicted) | F-0 ATPase subunit J (predicted) | 0.76 | 1.47 | 1.72 |
| SPAC343.12 | *rds1* | conserved fungal protein | conserved fungal protein | 0.71 | 1.30 | 1.72 |
| SPAC926.03 | *rlc1* | myosin II regulatory light chain | myosin II regulatory light chain (PMID 11056543) | 0.86 | 1.46 | 1.72 |
| SPAC19G12.07c | *rsd1* | RNA-binding protein Rsd1 | RNA-binding protein Rsd1 | 1.15 | 0.91 | 1.72 |
| SPAC343.19 | | phosphatidylinositol 4-kinase Lsb6 (predicted) | | 0.94 | 1.49 | 1.71 |
| SPBC23G7.08c | *rga7* | GTPase activating protein Rga7 | | 0.85 | 0.97 | 1.71 |
| SPBC17F3.01c | *rga5* | GTPase activating protein Rga5 | | 1.31 | 1.38 | 1.71 |
| SPAC630.09c | *mug58* | glycerate kinase (predicted) | glycerate kinase (predicted) | 0.80 | 1.16 | 1.71 |
| SPBC3D6.13c | | protein disulfide isomerase (predicted) | | 0.91 | 1.43 | 1.71 |
| SPAC1296.01c | | phosphoacetylglucosamine mutase (predicted) | phosphoacetylglucosamine mutase (predicted) | 0.89 | 1.46 | 1.71 |
| SPAC29B12.07 | *sec16* | multidomain vesicle coat component Sec16 | multidomain vesicle coat component Sec16 | 0.82 | 1.46 | 1.71 |
| SPAC12G12.03 | *cip2* | RNA-binding protein Cip2 | RNA-binding protein Cip2 | 1.03 | 1.24 | 1.71 |
| SPAC9G1.07 | | sequence orphan | sequence orphan | 0.90 | 1.36 | 1.71 |
| SPCC4E9.02 | *cig1* | cyclin Cig1 | | 0.88 | 0.65 | 1.71 |
| SPAC1A6.08c | *mug125* | sequence orphan | sequence orphan | 0.83 | 0.66 | 1.71 |
| SPBP23A10.13 | *orc4* | origin recognition complex subunit Orc4 | | 0.85 | 1.36 | 1.70 |
| SPCC1494.10 | | transcription factor (predicted) | | 0.91 | 1.18 | 1.70 |
| SPAC3A12.17c | *cys12* | cysteine synthase Cys12 | cysteine synthase Cys12 | 1.02 | 1.16 | 1.70 |
| SPCC1183.01 | *sec15* | exocyst complex subunit Sec15 (predicted) | exocyst complex subunit Sec15 (predicted) | 0.78 | 0.99 | 1.70 |
| SPAC821.13c | | P-type ATPase | | 1.07 | 1.30 | 1.70 |
| SPAC5H10.01 | | DUF1445 family protein | DUF1445 family protein | 0.87 | 1.14 | 1.70 |
| SPAC688.10 | *rev3* | DNA polymerase zeta catalytic subunit Rev3 | DNA polymerase zeta catalytic subunit Rev3 | 0.79 | 1.47 | 1.70 |
| SPBC1347.10 | *cdc23* | MCM-associated protein Mcm10 | | 0.89 | 1.47 | 1.70 |

| | | | | | | |
|---|---|---|---|---|---|---|
| SPAC3G9.12 | *peg1* | CLASP family microtubule-associated protein | CLASP family microtubule-associated protein | 0.88 | 1.14 | 1.69 |
| SPAC22F3.02 | *atf31* | transcription factor Atf31 | transcription factor Atf31 | 1.22 | 1.35 | 1.69 |
| SPAC6F6.13c | | DUF726 family protein | DUF726 family protein | 0.97 | 1.25 | 1.69 |
| SPBC23E6.02 | | ATP-dependent DNA helicase (predicted) | | 0.80 | 1.35 | 1.69 |
| SPAC29B12.01 | *ino80* | SNF2 family helicase Ino80 | SNF2 family helicase Ino80 | 0.83 | 1.43 | 1.69 |
| SPBC32F12.07c | | ubiquitin-protein ligase E3 (predicted) | | 0.86 | 1.43 | 1.69 |
| SPCC970.08 | | inositol polyphosphate kinase (predicted) | inositol polyphosphate kinase (predicted) | 0.84 | 1.14 | 1.69 |
| SPAC26A3.09c | *rga2* | GTPase activating protein Rga2 | GTPase activating protein Rga2 | 0.97 | 1.20 | 1.68 |
| SPAC1639.02c | *trk2* | potassium ion transporter Trk2 | | 0.98 | 1.18 | 1.68 |
| SPCC1620.13 | | phosphoglycerate mutase family | phosphoglycerate mutase family | 0.93 | 1.14 | 1.68 |
| SPCC550.12 | *arp6* | actin-like protein Arp6 | actin-like protein Arp6 | 0.78 | 0.98 | 1.68 |
| SPBC29A3.17 | *gef3* | RhoGEF Gef3 | | 0.76 | 0.96 | 1.68 |
| SPBC3B8.04c | | membrane transporter | | 1.01 | 1.33 | 1.68 |
| SPBC317.01 | *mbx2* | MADS-box transcription factor Pvg4 | | 0.39 | 1.16 | 1.67 |
| SPCC417.03 | | sequence orphan | sequence orphan | 0.73 | 0.82 | 1.67 |
| SPBC1826.01c | *mot1* | TATA-binding protein associated factor Mot1 | TATA-binding protein associated factor Mot1 | 0.88 | 1.20 | 1.67 |
| SPCC338.08 | *ctp1* | CtIP-related endonuclease | sequence orphan | 0.94 | 1.19 | 1.67 |
| SPAC16A10.03c | | zinc finger protein Pep5/Vps11 (predicted) | zinc finger protein Pep5/Vps11 (predicted) | 1.02 | 1.43 | 1.66 |
| SPBC28F2.12 | *rpb1* | DNA-directed RNA polymerase II large subunit | | 0.79 | 0.96 | 1.66 |
| SPBC428.20c | *alp6* | gamma tubulin complex Spc98/GCP3 subunit Alp6 | | 0.95 | 1.12 | 1.66 |
| SPAC6C3.06c | | P-type ATPase, calcium transporting | P-type ATPase, calcium transporting (PMID 12707717) | 0.92 | 1.34 | 1.65 |
| SPBC29A3.01 | | heavy metal ATPase | | 0.97 | 1.45 | 1.65 |
| SPBC649.05 | *cut12* | spindle pole body protein Cut12 | spindle pole body protein Cut12 (PMID 9531532) | 0.96 | 1.37 | 1.65 |
| SPAC1A6.07 | | sequence orphan | sequence orphan | 0.82 | 1.46 | 1.65 |
| SPAC4G9.05 | *mpf1* | meiotic PUF family protein 1 | meiotic PUF family protein 1 | 0.67 | 0.84 | 1.65 |
| SPAC27E2.01 | | alpha-amylase homolog (predicted) | alpha-amylase homolog (predicted) | 0.86 | 1.46 | 1.65 |
| SPAC17A5.05c | | conserved fungal protein | conserved fungal protein | 0.67 | 0.78 | 1.65 |
| SPAC14C4.04 | *B22918-2* | hypothetical protein | hypothetical protein | 0.88 | 1.42 | 1.65 |

| | | | | | | |
|---|---|---|---|---|---|---|
| SPBC26H8.02c | *sec9* | SNAP-25 homologue, t-SNARE component Sec9 | | 0.85 | 1.01 | 1.65 |
| SPAC1952.10c | | conserved fungal protein | conserved fungal protein (predicted) | 0.89 | 1.26 | 1.64 |
| SPBC342.05 | *crb2* | DNA repair protein RAD9 homolog, Rhp9 | | 0.89 | 1.40 | 1.64 |
| SPAC1783.02c | *vps66* | acyltransferase (predicted) | acyltransferase (predicted) | 0.95 | 1.33 | 1.64 |
| SPBC16A3.19 | | histone acetyltransferase complex subunit Eaf7 (predicted) | | 0.72 | 1.08 | 1.64 |
| SPAPB2C8.01 | | glycoprotein (predicted) | glycoprotein (predicted) | 0.84 | 1.31 | 1.64 |
| SPAC22H10.03c | *kap114* | karyopherin Kap14 | karyopherin Kap14 | 0.87 | 1.49 | 1.64 |
| SPBP26C9.03c | *fet4* | iron/zinc ion transporter (predicted) | iron ion transporter (predicted) | 0.88 | 1.46 | 1.63 |
| SPBC21B10.13c | | transcription factor, homeobox type (predicted) | | 0.86 | 0.96 | 1.63 |
| SPAC2F3.14c | | conserved fungal protein | conserved fungal protein | 0.70 | 0.91 | 1.63 |
| SPAC30D11.09 | *cwf19* | complexed with Cdc5 protein Cwf19 | complexed with Cdc5 protein Cwf19 (PMID 11884590) | 0.84 | 0.84 | 1.63 |
| SPCC1183.04c | *pet127* | mitochondrial membrane protein Pet127 | mitochondrial membrane protein Pet127 | 0.88 | 1.15 | 1.63 |
| SPAC57A7.11 | *mip1* | WD repeat protein Mip1 | WD repeat protein Mip1 | 0.72 | 1.08 | 1.63 |
| SPCC622.10c | | exocyst complex subunit Sec5 (predicted) | exocyst complex subunit Sec5 (predicted) | 0.78 | 1.05 | 1.63 |
| SPAC222.11 | *hem13* | coproporphyrinogen III oxidase (predicted) | coproporphyrinogen III oxidase (predicted) | 0.72 | 0.42 | 1.62 |
| SPBC1921.06c | *pvg3* | beta-1,3-galactosyltransferase | | 1.03 | 1.46 | 1.62 |
| SPAC1327.01c | | transcription factor, zf-fungal binuclear cluster type (predicted) | transcription factor (predicted) | 1.03 | 1.28 | 1.62 |
| SPCC736.04c | *gma12* | alpha-1,2-galactosyltransferase Gma12 | alpha-1,2-galactosyltransferase Gma12 (PMID 95003210) | 0.76 | 0.93 | 1.62 |
| SPCC1919.02 | | pig-X | pig-X | 1.07 | 1.48 | 1.62 |
| SPBC31F10.13c | *hip1* | hira protein Hip1 | | 0.82 | 1.44 | 1.62 |
| SPBC25B2.11 | *pof2* | F-box protein Pof2 | | 0.85 | 1.38 | 1.62 |
| SPCC1620.07c | | lunapark homolog | lunapark homolog | 1.21 | 1.46 | 1.62 |
| SPAC110.01 | *ppk1* | serine/threonine protein kinase Ppk1 (predicted) | serine/threonine protein kinase Ppk1 (predicted) | 0.99 | 1.46 | 1.62 |
| SPBC215.01 | | GTPase activating protein | | 0.70 | 1.25 | 1.62 |
| SPMIT.10 | *atp9* | F0-ATPase subunit 9 | F0-ATPase subunit 9; similar to S. cerevisiae Q0130 | 0.53 | 0.84 | 1.61 |
| SPBC18E5.14c | | sequence orphan | | 0.86 | 1.20 | 1.61 |
| SPAC26H5.05 | | IPT/TIG ankyrin repeat protein | IPT/TIG ankyrin repeat protein | 0.90 | 1.31 | 1.61 |
| SPCC4G3.07c | *phf1* | PHD finger containing protein Phf1 | PHD finger containing protein Phf1 | 0.91 | 1.22 | 1.61 |

| | | | | | | |
|---|---|---|---|---|---|---|
| SPAC823.05c | *tlg2* | SNARE Tlg2 (predicted) | SNARE Tlg2 | 0.84 | 1.24 | 1.61 |
| SPBC2D10.04 | | arrestin Aly1 related | | 0.87 | 1.25 | 1.61 |
| SPAC30D11.13 | *hus5* | SUMO conjugating enzyme Hus5 | SUMO conjugating enzyme Hus5 | 0.86 | 1.11 | 1.61 |
| SPAC1687.15 | *gsk3* | serine/threonine protein kinase Gsk3 | serine/threonine protein kinase Gsk3 | 0.90 | 1.38 | 1.61 |
| SPAC12G12.15 | *sif3* | Sad1 interacting factor 3 | Sad1 interacting factor 3 | 0.88 | 1.44 | 1.61 |
| SPBC2F12.08c | *ceg1* | mRNA guanylyltransferase Ceg1 | | 0.84 | 1.25 | 1.61 |
| SPAC15E1.04 | | thymidylate synthase (predicted) | thymidylate synthase (predicted) | 0.88 | 1.40 | 1.61 |
| SPBC1198.10c | | asparagine-tRNA ligase Slm5 | asparagine-tRNA ligase Slm5 | 0.90 | 1.26 | 1.61 |
| SPAC1296.05c | | cyclin L family cyclin | cyclin L family cyclin | 0.87 | 0.91 | 1.61 |
| SPAC1556.08c | *cbs2* | protein kinase activator (predicted) | | 0.91 | 1.37 | 1.61 |
| SPAC57A7.13 | | RNA-binding protein | RNA-binding protein | 0.94 | 1.47 | 1.60 |
| SPBC19C7.02 | *ubr1* | N-end-recognizing protein Ubr1 | | 0.77 | 1.28 | 1.60 |
| SPBC11B10.06 | *sws1* | SWIM domain containing-Srs2 interacting protein 1 | | 1.00 | 1.25 | 1.60 |
| SPAC3H1.12c | *snt2* | Lid2 complex subunit Snt2 | Lid2 complex subunit Snt2 (PMID 12488447) | 0.91 | 1.15 | 1.60 |
| SPBC21C3.19 | | DUF1960 family protein | | 0.66 | 1.41 | 1.60 |
| SPBC19C7.11 | | ClC chloride channel (predicted) | | 0.87 | 1.41 | 1.60 |
| SPBC428.08c | *clr4* | histone H3 methyltransferase Clr4 | histone H3 methyltransferase Clr4 | 0.88 | 1.41 | 1.60 |
| SPCC162.07 | *ent1* | epsin | epsin | 0.91 | 1.33 | 1.59 |
| SPAC3H5.09c | | conserved fungal protein | conserved fungal protein | 0.74 | 1.15 | 1.59 |
| SPBC3E7.12c | *chr1* | chitin synthase regulatory factor (putative) Chr1 | | 1.01 | 0.97 | 1.59 |
| SPBC685.02 | | conserved eukaryotic protein | | 1.08 | 1.43 | 1.59 |
| SPBC15D4.08c | | dubious | | 0.61 | 0.93 | 1.59 |
| SPCP1E11.05c | | sterol O-acyltransferase (predicted) | sterol O-acyltransferase (predicted) | 0.95 | 1.32 | 1.59 |
| SPAC1486.02c | *ucp14* | UBA domain protein Ucp14 | UBA domain protein Ucp14 | 0.84 | 1.32 | 1.59 |
| SPAC23H3.02c | *ini1* | RING finger-like protein Ini1 | RING finger-like protein Ini1 | 0.71 | 1.01 | 1.58 |
| SPCC663.15c | | conserved fungal protein | conserved fungal protein | 0.94 | 1.46 | 1.58 |
| SPAC2H10.01 | | transcription factor, zf-fungal binuclear cluster type (predicted) | transcription factor | 0.55 | 1.28 | 1.58 |
| SPCC1020.12c | | xap-5-like protein | xap-5-like protein | 0.90 | 1.49 | 1.58 |
| SPCC777.03c | | nifs homolog | nifs homolog | 0.72 | 0.95 | 1.58 |
| SPAPB24D3.03 | | agmatinase (predicted) | agmatinase (predicted) | 0.88 | 1.26 | 1.58 |
| SPCC1235.07 | *fta7* | Sim4 and Mal2 associated (4 and 2 associated) protein 7 | Sim4 and Mal2 associated (4 and 2 associated) protein 7 (PMID 16079914) | 0.99 | 0.88 | 1.58 |
| SPBC8D2.03c | *hhf2* | histone H4 h4.2 | | 1.00 | 0.87 | 1.58 |
| SPAC17A2.05 | *osm1* | fumerate reductase | fumerate reductase | 0.83 | 0.84 | 1.58 |

| | | | | | | |
|---|---|---|---|---|---|---|
| SPBC56F2.01 | *pof12* | F-box protein Pof12 | | 0.97 | 1.45 | 1.58 |
| SPBP35G2.09 | *usp103* | U1 snRNP-associated protein Usp103 (predicted) | | 0.92 | 1.02 | 1.58 |
| SPCC23B6.04c | | sec14 cytosolic factor family | sec14 cytosolic factor family | 1.01 | 1.35 | 1.58 |
| SPAC25B8.11 | | transcription factor (predicted) | transcription factor | 0.97 | 1.46 | 1.58 |
| SPAC7D4.03c | | conserved fungal family | conserved fungal family | 0.88 | 1.27 | 1.58 |
| SPBP23A10.12 | | FRG1 family protein | | 0.68 | 1.09 | 1.57 |
| SPAC589.11 | *mug82* | mitochondrial translation release factor (predicted) | translation release factor (predicted) | 0.75 | 1.13 | 1.57 |
| SPAC3C7.07c | | arginine-tRNA protein transferase (predicted) | arginine-tRNA protein transferase (predicted) | 0.99 | 1.49 | 1.57 |
| SPCC794.11c | | ENTH domain protein Ent3 | ENTH domain protein Ent3 | 0.99 | 1.32 | 1.57 |
| SPBC800.03 | *clr3* | histone deacetylase (class II) Clr3 | histone deacetylase (class II) Clr3 | 1.01 | 1.39 | 1.57 |
| SPBC4C3.06 | | actin cytoskeletal protein Syp1 | | 0.97 | 1.37 | 1.57 |
| SPAC20H4.10 | *ufd2* | ubiquitin-protein ligase E4 (predicted) | ubiquitin-protein ligase E4 (predicted) | 0.98 | 1.49 | 1.57 |
| SPAC24H6.06 | *sld3* | DNA replication pre-initiation complex subunit Sld3 | pre-initiation complex subunit Sld3 (PMID 12006645) | 0.91 | 1.33 | 1.57 |
| SPAC29E6.10c | | kinetochore protein (predicted) | kinetochore protein (predicted) | 0.81 | 1.14 | 1.57 |
| SPCC895.04c | *ufe1* | SNARE Ufe1 | SNARE Ufe1 | 0.90 | 1.42 | 1.57 |
| SPBC3F6.05 | *rga1* | GTPase activating protein Rga1 | | 1.19 | 1.37 | 1.56 |
| SPAC22E12.19 | | histone deacetylase complex subunit (predicted) | histone deacetylase complex subunit (predicted) | 0.93 | 0.89 | 1.56 |
| SPAC4F8.11 | | WD repeat protein, human WDR24 family | WD repeat protein, human WDR24 family | 0.82 | 1.43 | 1.56 |
| SPCC1827.08c | *pof7* | F-box protein Pof7 | F-box protein Pof7 | 0.79 | 1.28 | 1.56 |
| SPCC24B10.16c | | sequence orphan | sequence orphan | 0.85 | 1.50 | 1.56 |
| SPAC664.07c | *rad9* | checkpoint clamp complex protein Rad9 | checkpoint clamp complex protein Rad9 | 1.19 | 1.27 | 1.56 |
| SPAC1002.18 | *urg3* | DUF1688 family protein | DUF1688 family protein | 0.79 | 1.19 | 1.56 |
| SPBC1861.09 | *ppk22* | serine/threonine protein kinase Ppk22 (predicted) | | 0.83 | 1.21 | 1.56 |
| SPBC83.09c | | GYF domain | | 0.81 | 1.10 | 1.56 |
| SPAC3G9.07c | *hos2* | histone deacetylase (class I) Hos2 | histone deacetylase (class I) Hos2 | 0.82 | 0.97 | 1.56 |
| SPCC576.05 | | nucear export factor | nucear export factor | 0.77 | 1.26 | 1.56 |
| SPBC887.09c | | leucine-rich repeat protein Sog2 (predicted) | | 0.94 | 1.26 | 1.56 |
| SPAC57A10.09c | | High-mobility group non-histone chromatin protein | High-mobility group non-histone chromatin protein | 0.98 | 1.40 | 1.56 |

| | | | | | | |
|---|---|---|---|---|---|---|
| SPAC110.02 | *pds5* | cohesin-associated protein Pds5 | cohesin-associated protein Pds5 | 0.86 | 1.30 | 1.56 |
| SPBC1348.11 | | membrane transporter | membrane transporter | 0.42 | 0.48 | 1.55 |
| SPCC4B3.04c | *nte1* | lysophospholipase | lysophospholipase | 0.99 | 1.30 | 1.55 |
| SPAC10F6.05c | *ubc6* | ubiquitin conjugating enzyme Ubc6 | ubiquitin conjugating enzyme Ubc6 | 0.88 | 1.22 | 1.55 |
| SPBC1215.01 | *shy1* | SURF-family protein Shy1 | SURF-family protein Shy1 | 0.87 | 1.37 | 1.55 |
| SPAC22H10.11c | | | | 0.92 | 1.27 | 1.55 |
| SPCC594.07c | | sequence orphan | sequence orphan | 0.97 | 1.30 | 1.54 |
| SPAC24H6.09 | *gef1* | RhoGEF Gef1 | RhoGEF Gef1 | 0.90 | 1.16 | 1.54 |
| SPCC584.05 | *sec1* | SNARE binding protein Sec1 | SNARE binding protein Sec1 | 0.83 | 1.40 | 1.54 |
| SPAC13C5.07 | *rad32* | Rad32 nuclease | Rad32 nuclease | 0.96 | 1.13 | 1.54 |
| SPCC16C4.05 | | RNase P and RNase MRP subunit (predicted) | RNase P and RNase MRP subunit (predicted) | 0.74 | 0.83 | 1.54 |
| SPCC1827.02c | | cholinephosphate cytidylyltransferase (predicted) | cholinephosphate cytidylyltransferase (predicted) | 1.01 | 1.30 | 1.54 |
| SPCC1235.03 | | SMR and CUE domain protein | SMR and CUE domain protein | 0.65 | 1.31 | 1.54 |
| SPAC9G1.02 | *wis4* | MAP kinase kinase kinase Wis4 | MAP kinase kinase kinase Wis4 | 0.77 | 1.23 | 1.53 |
| SPAC17H9.18c | | dubious | dubious | 0.81 | 0.76 | 1.53 |
| SPAC6G9.15c | | sequence orphan | sequence orphan | 0.76 | 1.06 | 1.53 |
| SPCC645.13 | | transcription elongation regulator | transcription elongation regulator | 0.93 | 1.37 | 1.53 |
| SPAC2G11.12 | *rqh1* | RecQ type DNA helicase Rqh1 | RecQ type DNA helicase Rqh1 | 0.92 | 1.34 | 1.53 |
| SPAC630.11 | *vps55* | vacuolar sorting protein Vps55 (predicted) | vacuolar sorting protein Vps55 (predicted) | 0.85 | 1.24 | 1.52 |
| SPAC2F3.15 | *lsk1* | latrunculin sensitive kinase Lsk1 | latrunculin sensitive kinase Lsk1 (PMID 15537703) | 0.92 | 0.96 | 1.52 |
| SPCC162.08c | *nup211* | nuclear pore complex associated protein | nuclear pore complex associated protein | 0.87 | 1.38 | 1.52 |
| SPCC1235.10c | *sec6* | exocyst complex subunit Sec6 | exocyst complex subunit Sec6 | 1.07 | 1.44 | 1.52 |
| SPBC1685.07c | | amino acid transporter (predicted) | amino acid transporter (predicted) | 0.89 | 1.44 | 1.52 |
| SPAC1002.02 | *mug31* | nucleoporin Pom34 (predicted) | nucleoporin Pom34 (predicted) | 0.94 | 0.86 | 1.52 |
| SPCC1840.01c | *mog1* | Ran GTPase binding protein Mog1 | | 0.78 | 1.48 | 1.52 |
| SPBC14F5.13c | | alkaline phosphatase (predicted) | | 0.95 | 1.31 | 1.52 |
| SPCC1442.17c | | DUF292 family protein | DUF292 family protein | 0.95 | 1.03 | 1.52 |
| SPAC20H4.06c | | RNA-binding protein | RNA-binding protein | 0.79 | 1.34 | 1.52 |
| SPBC530.11c | | transcription factor (predicted) | | 0.81 | 1.23 | 1.52 |
| SPAC1565.02c | | GTPase activating protein | GTPase activating protein | 0.86 | 1.47 | 1.52 |
| SPBC582.06c | *mcp6* | horsetail movement protein Hrs1/Mcp6 | meiosis specific coiled-coil protein Mcp6 | 1.01 | 0.86 | 1.52 |

| | | | | | | |
|---|---|---|---|---|---|---|
| SPAC1A6.01c | | human thyroid receptor interacting protein homolog | human thyroid receptor interacting protein homolog | 0.89 | 1.23 | 1.51 |
| SPBC2A9.02 | | NAD dependent epimerase/dehydratase family protein | | 0.95 | 1.19 | 1.51 |
| SPAP27G11.14c | | sequence orphan | sequence orphan | 0.81 | 1.16 | 1.51 |
| SPCC830.05c | *epl1* | histone acetyltransferase complex subunit Epl1 (predicted) | histone acetyltransferase complex subunit Epl1 (predicted) | 0.77 | 1.17 | 1.51 |
| SPCC553.01c | | meiotic chromosome segregation protein | | 0.86 | 1.29 | 1.51 |
| SPBC1734.07c | | TRAPP complex subunit Trs85 (predicted) | | 0.74 | 0.93 | 1.51 |
| SPCC1020.13c | | phospholipase (predicted) | phospholipase (predicted) | 0.96 | 1.29 | 1.51 |
| SPBC56F2.11 | *met6* | homoserine O-acetyltransferase | | 0.88 | 1.22 | 1.51 |
| SPBC409.03 | *swi5* | Swi5 protein | | 0.75 | 1.02 | 1.51 |
| SPBC16A3.01 | *spn3* | septin Spn3 | | 0.89 | 1.20 | 1.51 |
| SPBC530.14c | *dsk1* | SR protein-specific kinase Dsk1 | | 0.91 | 1.06 | 1.51 |
| SPCC663.01c | *ekc1* | protein phosphatase regulatory subunit Ekc1 (predicted) | protein phosphatase regulatory subunit Ekc1 (predicted) | 1.03 | 1.44 | 1.51 |
| SPBC11C11.11c | | mitochondrial ATP-dependent DNA helicase Irc3 (predicted) | | 0.92 | 1.37 | 1.51 |
| SPBC3B9.15c | *scp1* | sterol regulatory element binding protein Scp1 | | 0.98 | 1.09 | 1.51 |
| SPAC1805.01c | *ppk6* | serine/threonine protein kinase Ppk6 | serine/threonine protein kinase Ppk6 | 0.89 | 1.08 | 1.50 |
| SPBC29A10.13 | *atp7* | F0-ATPase subunit D | | 0.83 | 1.41 | 1.50 |
| SPBC146.11c | *mug97* | meiotically upregulated gene Mug97 | | 0.66 | 0.96 | 1.50 |
| SPAC16C9.07 | *ppk5* | serine/threonine protein kinase Ppk5 (predicted) | | 0.87 | 1.43 | 1.50 |
| SPBC577.06c | | phosphatidylinositol kinase (predicted) | | 0.84 | 0.97 | 1.50 |
| SPAC31A2.11c | *cuf1* | Cu metalloregulatory transcription factor Cuf1 | Cu metalloregulatory transcription factor Cuf1 (PMID 10593913) | 0.92 | 1.24 | 1.50 |
| SPBP35G2.02 | | DUF1000 family protein | | 0.87 | 1.32 | 1.50 |
| SPBC2D10.14c | *myo51* | myosin type V | | 0.96 | 1.15 | 1.50 |
