# Supplementary material for: The Fission Yeast GATA Factor, Gaf1, Modulates Sexual Development via Direct Down-Regulation of ste11+ Expression in Response to Nitrogen Starvation
Source: PLoS One. 2012 Aug 10;7(8):e42409. doi: 10.1371/journal.pone.0042409 (PMC3416868; doi:10.1371/journal.pone.0042409)
Supplement: Table S9 — List of the genes in Subgroup F. (PDF) [file pone.0042409.s009.pdf]

Table S9. List of the genes in Subgroup F

*** 6 genes**

Systematic	Gene name	Description (GeneDB)	Description (FunCat2)	Expression Ratio (gaf1Δ, +N) / (WT, +N)	Expression Ratio (WT, -N) / (WT, +N)	Expression Ratio (gaf1Δ, -N) / (gaf1Δ, +N)
SPAC1002.16c		nicotinic acid plasma membrane transporter (predicted)	nicotinic acid plasma membrane transporter (predicted)	2.18	1.33	1.61
SPAC22F8.04		triose phosphate transporter (predicted)	triose phosphate transporter (predicted)	1.69	1.10	2.15
SPBC2G2.09c	*crs1*	meiosis specific cyclin Crs1		1.65	1.19	1.61
SPAC222.15	*meu13*	Tat binding protein 1(TBP-1)-interacting protein (TBPIP) homolog (predicted)		1.64	1.26	1.59
SPBPJ4664.02		glycoprotein (predicted)		1.64	1.26	1.83
SPAC27F1.10		sequence orphan	sequence orphan	1.54	1.31	3.05
