# Supplementary material for: The Fission Yeast GATA Factor, Gaf1, Modulates Sexual Development via Direct Down-Regulation of ste11+ Expression in Response to Nitrogen Starvation
Source: PLoS One. 2012 Aug 10;7(8):e42409. doi: 10.1371/journal.pone.0042409 (PMC3416868; doi:10.1371/journal.pone.0042409)
Supplement: Table S10 — List of the genes in Subgroup G. (PDF) [file pone.0042409.s010.pdf]

Table S10. List of the genes in Subgroup G

*** 60 genes**

Systematic	Gene name	Description (GeneDB)	Description (FunCat2)	Expression Ratio (gaf1Δ, +N) / (WT, +N)	(WT, -N) / (WT, +N)	(gaf1Δ, -N) / (gaf1Δ, +N)
SPAC13F5.07c		term=sequence orphan; date=20080121	previusly annotated as dubious, may not be protein coding	7.11	16.28	3.10
SPBCPT2R1.06c		pseudogene		5.62	10.23	1.64
SPCC576.16c	*wtf22*	wtf element Wtf22		5.33	11.02	2.94
SPBC23G7.10c		NADH-dependent flavin oxidoreductase (predicted)		5.10	7.97	3.77
SPBC839.06	*cta3*	P-type ATPase, calcium transporting Cta3	P-type ATPase, calcium transporting Cta3	4.70	3.39	5.13
SPAC29E6.07		sequence orphan	sequence orphan	4.40	2.60	4.86
SPAPB17E12.09		sequence orphan	sequence orphan	4.15	9.85	2.15
SPAC1F8.01	*ght3*	hexose transporter Ght3	hexose transporter Ght3 (PMID 10735857)	4.07	93.52	25.29
SPAC27D7.03c	*mei2*	RNA-binding protein involved in meiosis Mei2	RNA-binding protein involved in meiosis Mei2	4.07	43.74	9.30
SPCC191.10		sequence orphan	sequence orphan	3.80	59.80	6.79
SPAC4F10.17		conserved fungal protein	conserved fungal protein	3.74	165.16	5.84
SPBC1348.14c	*ght7*	hexose transporter Ght7	hexose transporter Ght7	2.81	2.58	10.19
SPBC887.16		dubious		2.80	2.90	2.89
SPBC1198.12	*mfr1*	fizzy-related protein Mfr1		2.79	3.64	1.81
SPBC32H8.06	*mug93*	TPR repeat protein, meiotically spliced		2.64	3.86	3.56
SPAC186.02c		hydroxyacid dehydrogenase (predicted)	hydroxyacid dehydrogenase (predicted)	2.59	1.70	1.59
SPAC9E9.17c		dubious	dubiuos	2.50	2.75	4.30
SPAC1039.09	*isp5*	amino acid permease Isp5	amino acid permease Isp5	2.37	33.67	3.77
SPAC17A5.11	*rec12*	endonuclease Rec12	endonuclease Rec12	2.35	2.69	1.70
SPCC622.21	*wtf12*	wtf element Wtf12	wtf element Wtf12	2.34	2.58	1.93
SPAC32A11.01	*mug8*	conserved fungal protein	conserved fungal protein	2.27	5.82	4.25
SPAC1F8.04c		hydrolase (predicted)	hydrolase (predicted)	2.21	107.88	13.74
SPAC212.07c		pseudogene	pseudogene	2.21	5.24	2.91
SPCC548.02c	*wtf3*	wtf element Wtf3	wtf element Wtf3	2.19	7.50	3.30

SPAC22F8.02c	*pvg5*	PvGal biosynthesis protein Pvg5	PvGal biosynthesis protein Pvg5	2.17	1.94	2.41
SPAC6G10.06		FAD-dependent amino acid oxidase (predicted)	amino acid oxidase (predicted)	2.16	12.04	4.60
SPAC4G9.07	*mug133*	S. pombe specific UPF0300 family protein 2	S. pombe specific UPF0300 family protein 2	2.16	4.37	2.80
SPCC1223.02	*nmt1*	no message in thiamine Nmt1	no message in thiamine Nmt1	2.14	1118.03	705.95
SPBC23G7.11		DNA-3-methyladenine glycosidase Mag2 (predicted)		2.01	5.98	4.51
SPAC1952.15c	*rec24*	meiotic recombination protein Rec24	meiotic recombination protein Rec24	2.00	11.82	5.57
SPAC31G5.09c	*spk1*	MAP kinase Spk1	MAP kinase Spk1	1.95	36.46	23.90
SPCC1183.10	*wtf10*	wtf element Wtf10	wtf element Wtf10	1.94	3.38	1.90
SPAC750.07c		S. pombe specific GPI anchored protein family 1	S. pombe specific GPI anchored protein family 1	1.93	2.35	3.77
SPCC1840.12		OPT oligopeptide transporter family	OPT oligopeptide transporter family	1.91	8.80	1.76
SPCC794.03		amino acid permease, unknown 13	amino acid permease, unknown 13	1.91	2.58	3.00
SPAC17A2.11		sequence orphan	sequence orphan	1.89	9.58	2.44
SPCC74.09	*mug24*	RNA-binding protein, rrm type	RNA-binding protein	1.82	2.69	4.90
SPBC32C12.02	*ste11*	transcription factor Ste11		1.76	5.87	3.10
SPBC2G2.17c		beta-glucosidase Psu2 (predicted)		1.74	2.64	2.71
SPBC359.06	*mug14*	adducin	adducin	1.73	82.76	45.98
SPAC11E3.06	*map1*	MADS-box transcription factor Map1	MADS-box transcription factor Map1	1.72	11.77	6.14
SPBC19C2.04c	*ubp11*	ubiquitin C-terminal hydrolase Ubp11	ubiquitin C-terminal hydrolase Ubp11	1.72	5.07	3.84
SPBC24C6.06	*gpa1*	G-protein alpha subunit		1.70	11.54	7.32
SPAC1565.04c	*ste4*	adaptor protein Ste4	adaptor protein Ste4	1.69	10.87	5.41
SPAC26H5.09c		GFO/IDH/MocA family oxidoreductase	GFO/IDH/MocA family oxidoreductase	1.66	2.83	2.06
SPCPB1C11.01	*amt1*	ammonium transporter Amt1	ammonium transporter Amt1	1.63	3.81	2.06
SPAC3F10.10c	*map3*	pheromone M-factor receptor	pheromone M-factor receptor (PMID 8380233)	1.63	6.53	7.13
SPBC19F8.06c	*meu22*	amino acid permease, unknown 11		1.62	1.69	2.78
SPBC660.06		conserved fungal protein	conserved fungal protein	1.61	3.78	1.93
SPBPB21E7.08		pseudogene	pseudogene	1.61	3.91	2.96
SPAC1399.01c		membrane transporter (predicted)	purine permease (predicted)	1.56	16.03	6.74
SPCC162.04c	*wtf13*	wtf element Wtf13	wtf element Wtf13	1.55	2.89	2.39
SPAC1F8.05	*isp3*	sequence orphan	sequence orphan	1.55	33.50	26.64
SPAPB2B4.04c		P-type ATPase, calcium transporting Pmc1	P-type ATPase, calcium transporting Pmc1 (PMID 12707717)	1.54	3.75	2.29
SPCC162.10	*ppk33*	serine/threonine protein kinase Ppk33 (predicted)	serine/threonine protein kinase Ppk33 (predicted)	1.53	46.35	30.03

SPAPB8E5.05	*mfm1*	M-factor precursor Mfm1	M-factor precursor Mfm1	1.53	55.56	37.23
SPCPB1C11.02		amino acid permease, unknown 16	amino acid permease, unknown 16	1.51	3.50	4.50
SPCC1795.06	*map2*	P-factor	P-factor (PMID 8314086)	1.50	101.73	66.21
SPCC4G3.08	*psk1*	serine/threonine protein kinase Psk1	serine/threonine protein kinase Psk1	1.50	2.46	2.36
SPAC513.04		sequence orphan	sequence orphan	1.50	1.50	8.96
